# Diagnostic potential of the amniotic fluid cells transcriptome in deciphering mendelian disease: a proof-of-concept

**DOI:** 10.1038/s41525-022-00347-4

**Published:** 2022-12-28

**Authors:** Mianne Lee, Anna K. Y. Kwong, Martin M. C. Chui, Jeffrey F. T. Chau, Christopher C. Y. Mak, Sandy L. K. Au, Hei Man Lo, Kelvin Y. K. Chan, Vicente A. Yépez, Julien Gagneur, Anita S. Y. Kan, Brian H. Y. Chung

**Affiliations:** 1 Department of Paediatrics and Adolescent Medicine, School of Clinical Medicine, Li Ka Shing Faculty of Medicine, The University of Hong Kong, Hong Kong, SAR China; 2 Department of Obstetrics and Gynaecology, School of Clinical Medicine, Li Ka Shing Faculty of Medicine, The University of Hong Kong, Hong Kong, SAR China; 3 Department of Obstetrics and Gynaecology, Queen Mary Hospital, Hong Kong, SAR China; 4 Prenatal Diagnostic Laboratory, Department of Obstetrics and Gynaecology, Tsan Yuk Hospital, Hong Kong, SAR China; 5 Department of Informatics, Technical University of Munich, Garching, Germany; 6 Institute of Human Genetics, School of Medicine, Technical University of Munich, Munich, Germany

**Keywords:** Molecular medicine, Paediatric research, Genetic testing

## Abstract

RNA sequencing (RNA-seq) is emerging in genetic diagnoses as it provides functional support for the interpretation of variants of uncertain significance. However, the use of amniotic fluid (AF) cells for RNA-seq has not yet been explored. Here, we examined the expression of clinically relevant genes in AF cells (*n* = 48) compared with whole blood and fibroblasts. The number of well-expressed genes in AF cells was comparable to that in fibroblasts and much higher than that in blood across different disease categories. We found AF cells RNA-seq feasible and beneficial in prenatal diagnosis (*n* = 4) as transcriptomic data elucidated the molecular consequence leading to the pathogenicity upgrade of variants in *CHD7* and *COL1A2* and revising the in silico prediction of a variant in *MYRF*. AF cells RNA-seq could become a reasonable choice for postnatal patients with advantages over fibroblasts and blood as it prevents invasive procedures.

## Introduction

Variants of uncertain significance (VUSs) account for a large proportion of the total variants and depending on the particular gene ranges from 40–64%^1–3^. The majority of VUSs are rare^4^; thus, their interpretation is challenging and can hinder prompt genetic diagnoses of Mendelian diseases. In practice, rare VUSs, particularly those in regulatory regions, will become more prevalent with the increased affordability and accessibility of whole exome sequencing (WES) and whole genome sequencing (WGS)^5^. A plethora of ongoing research in metabolomics, proteomics, methyl profiling, in vitro or in vivo functional studies, and transcriptome/RNA sequencing (RNA-seq) has attempted to address the complexities in interpreting VUS^6–9^. The ability of RNA-seq to simultaneously examine all expressed transcripts in a specific tissue places it the forefront of enhancing the pathogenic classification of variants^10^.

RNA-seq is emerging as a direct and useful tool for genetic diagnoses as it provides functional advantages and supports the interpretation of VUS. While WES and WGS have achieved a genetic diagnostic rate of 25–55% across a wide spectrum of Mendelian disorders^11–13^, RNA-seq, as a complementary diagnostic platform, is estimated to increase the diagnostic rate by 6–36%, with an average expected increase of 21%^6,14–21^. The variation in yield depends on the selection criteria, disease category, and tissues used. Recently, Yépez et al.^22^ developed a modular computational workflow, the “Detection of RNA Outliers Pipeline” (DROP), that integrates all fundamental RNA-seq analysis steps for robust clinical diagnoses. DROP was implemented in two recent studies with a diagnostic rate of 16%^18,21^; however, neither of these studies focused on prenatal diagnoses.

To date, there is limited RNA-seq data on amniotic fluid (AF) cells^23,24^. The Genotype-Tissue Expression (GTEx) consortium^25^, which catalogued tissue-specific gene expression levels for over 50 reference/non-disease tissue types^25^, lacks information regarding AF cells. Although previous studies have focused on RNA-seq in cell-free (cf) transcriptomes^23,26,27^, others have reported the disadvantages of cfRNA, including its ease of contamination with wild-type DNA from leukocyte lysis and short half-life. For optimal results, the use of specialized tubes with prompt processing time is recommended^28^. In addition, cfRNA is highly variable for transcripts with low concentrations; therefore, a standardized liquid biopsy workflow is required, which increases the difficulty of clinical implementation.

AF sampling via amniocentesis is the preferred diagnostic test when fetal anomalies are detected in ultrasounds examination. The relative ease of access to these samples in laboratories makes RNA from AF cells a more convenient choice than cfRNA. In addition, stored AF cells samples can be retrieved later for further investigation after birth. Chorionic villus sampling (CVS) admittedly have the same or even better advantages in terms of early accessibility and capacity for prompt decision-making; however, CVS is comparatively indirect as it originates from the placenta as opposed to amniotic cells, which come from the epiblast that eventually develops into the embryo^29^.

Arguably, whole blood can be considered the most routinely used sample type for RNA-seq postnatally, yet it is suggested that skin fibroblasts are preferable to whole blood for detecting RNA expression because they harbour a much higher number of well-expressed genes^18^. However, obtaining fibroblasts from skin biopsies is an invasive procedure, which makes AF cells a better option for RNA-seq in postnatal settings.

The embryonic origin of AF is similar to that of skin fibroblasts due to the constant bidirectional diffusion between the AF and fetus across the un-keratinized skin during early embryonic development^30^. Since the basic structure of the epidermis develops immediately after fertilization, AF contains all skin components until the fetal skin becomes fully keratinized at approximately 25 weeks of gestation^30–33^.

Given the similarities in the embryonic origin of AF and skin fibroblasts, we hypothesized that AF cells obtained during the second trimester (16–24 weeks) may express a large number of genes, similar to skin fibroblasts^18^. This study therefore compared the expression of clinically relevant genes in AF cells to that in blood and skin fibroblasts and investigated the possibility of using AF cells for RNA-seq in both prenatal and postnatal settings. We applied an outlier approach using DROP, in the prenatal setting with sufficient sample size for the accurate detection of splicing and expression outliers. This pioneer work explored the contribution of AF cells RNA-seq to molecular diagnostics.

## Results

### Demographics

We recruited 52 fetuses, comprising 23 females and 29 males. All 52 AF samples were obtained from pregnant women with a gestational age ranging from 16 weeks and 0 days to 21 weeks and 3 days between August 2020 and May 2021, except for one AF sample retrieved from a pregnancy in 2017. Forty-eight AF samples serve as the nondiseased controls and four AF samples were used for validation.

### RNA-seq quality control

The sequencing depth range was 106–177 million reads per sample (median: 121 million). On average, 94% of bases achieved a quality score of Q30. Over 12,500 genes passed quality filtering (Supplementary Fig. 1).

### Gene expression profiles for whole blood, fibroblasts, and AF cells

**Fig. 1:**
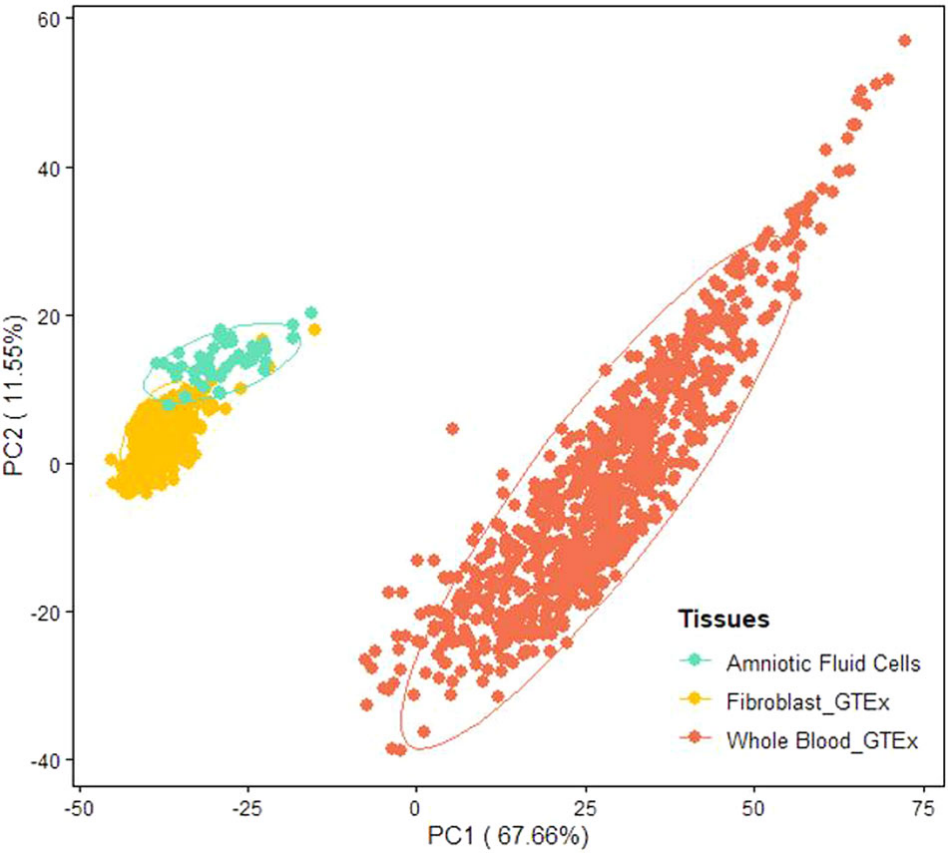
Principal component analysis (PCA) plot based on the expression profiles of GTEx whole blood (*n* = 504), GTEx fibroblasts (*n* = 755), and AF cells (*n* = 48). The top 1000 genes with the largest variance among GTEx whole blood, GTEx fibroblasts, and AF cells were used to generate this plot. The corresponding PCA plot with additional tissues (i.e. whole blood from 125 live participants, 803 GTEx muscle samples and 330 Induced pluripotent stem cells (iPSC) samples) is shown in Supplementary Fig. 6.

**Fig. 2:**
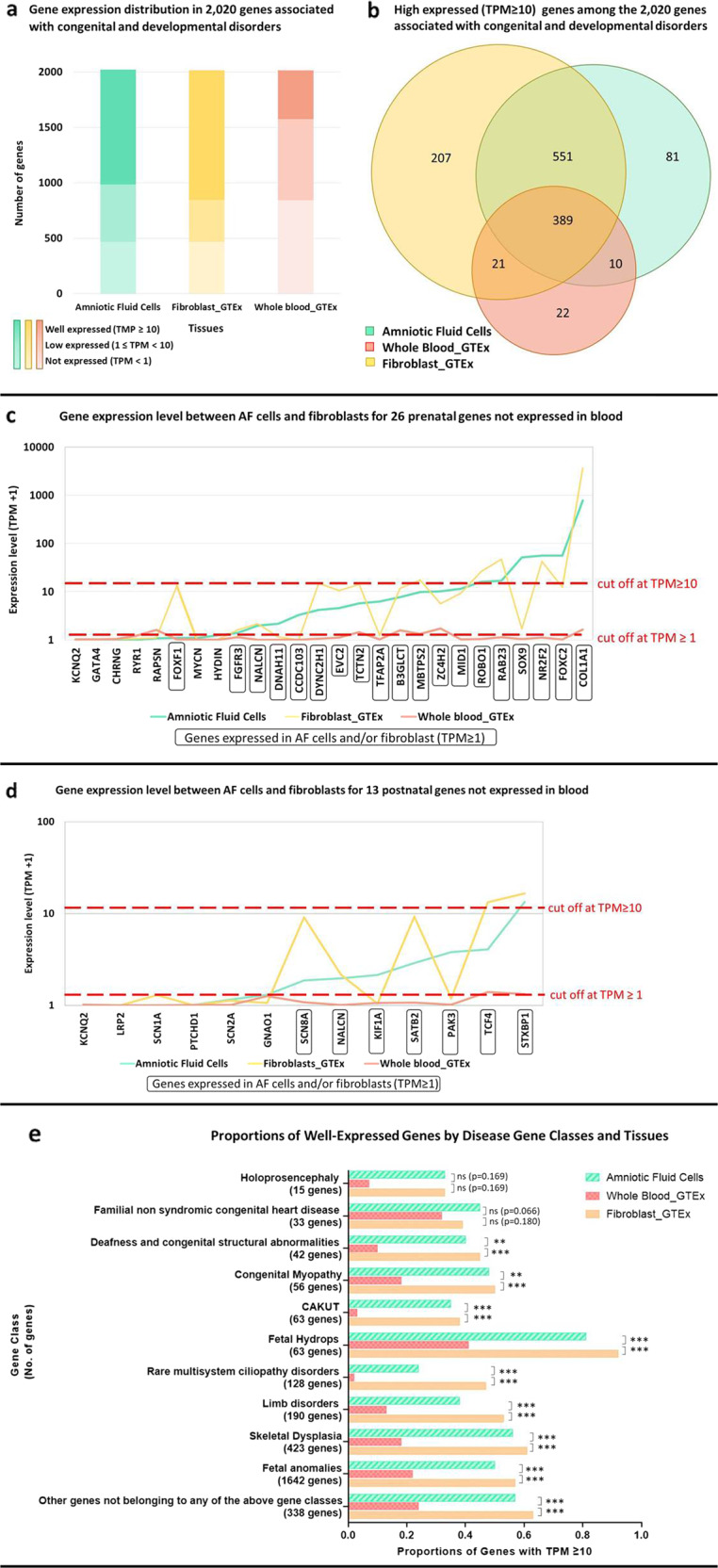
Gene expressions comparison among whole blood, fibroblasts and AF cells. **a** Number of well-expressed (median TPM ≥ 10), low-expressed (1 ≤ TMP < 10) and not expressed (median TPM < 1) genes among the 2,020 genes associated with congenital and developmental disorders on GTEx whole blood (*n* = 504), GTEx fibroblasts (*n* = 755), and AF cells (*n* = 48). **b** Venn diagram of genes well-expressed in whole blood, fibroblasts, and AF cells among the 2020 genes. **c** Gene expression in AF cells and fibroblasts of 26 genes not expressed in the whole blood. These genes were diagnostic or potentially clinically useful (i.e. presenting with a pathogenic/likely pathogenic/VUS variant) within the prenatal cohort of 610 fetuses in the PAGE study^34^. Red dotted line showing the cut off at median TPM ≥ 10. **d** Expression of 13 genes in AF cells and fibroblasts that were not expressed in the whole blood. These were recurrent diagnostic genes in the DDD study^35,36^. Red dotted line shows the cut off at median TPM ≥ 10. **e** Proportions of well-expressed genes in 11 gene classes across GTEx whole blood, GTEx fibroblasts, and AF cells. TPM transcript per million reads, AF amniotic fluid, ns not statistically significant; **p ≤ 0.01; ***p ≤ 0.00.

Principle component analysis (PCA) of the expression profiles in GTEx whole blood, GTEx fibroblasts, and our AF cells revealed three distinct clusters, with AF cells and fibroblasts exhibiting similar expression profiles (Fig. 1). Among the 2020 curated genes associated with congenital and developmental disorders (See Methods), more than half were well-expressed (median TPM ≥ 10) in both AF cells (51%) and fibroblasts (58%), whereas fewer than 500 genes (22%) were well-expressed in whole blood (Fig. 2a). AF cells and fibroblasts shared most of the well-expressed genes, with 81% (940/1168) overlap (Fig. 2b). Only 35% (410/1168) of the well-expressed genes in fibroblasts and 34% (399/1168) of those in AF cells were found in whole blood. Gene expression count matrices, as well as the privacy‑preserving count matrices of split and unsplit reads overlapping annotated splice sites from RNA‑seq, are available for download from the Zenodo repository without restrictions (https://zenodo.org/record/7079684#.YyLGoXZByUl).

Of the 2020 curated genes, 61 were considered diagnostic or potentially clinically useful (i.e., presenting with a pathogenic/likely pathogenic/VUS variant) within the prenatal cohort of 610 fetuses in the large-scale Prenatal Assessment of Genomes and Exomes (PAGE) study^34^. Of these 61 genes, 26 were not expressed (median TPM < 1) in whole blood, whereas over 50% could be detected in AF cells or fibroblasts (with median TPM ≥ 1), thereby further strengthening the feasibility of RNA-seq in AF cells for prenatal diagnoses (Fig. 2c). Four genes (*SOX9*, *TFAP2A*, *CCDC103*, and *DNAH11*) were expressed at a significantly higher level in AF cells than in fibroblasts.

Similarly, 54 out of the 2020 genes were recurrent diagnostic genes with potentially significant implications for postnatal genetic diagnosis according to the large-scale Deciphering Developmental Disorders (DDD) study^35,36^. Among them, 13 genes were not expressed (median TPM < 1) in whole blood, five and seven genes were expressed (median TPM ≥ 1) in fibroblasts and AF cells, respectively. Expression of *SCN8A*, *NALCN*, S*ATB2*, *TCF4* and *STXBP1* was detected in fibroblasts, whereas expression of *SCN8A*, *NALCN, KIF1A*, *PAK3*, *SATB2*, *TCF4*, and *STXBP1* was detected in AF cells (Fig. 2d). Notably, *STXBP1*, which contributed to six diagnoses in the DDD study^35,36^, was well expressed in both fibroblasts and AF cells. Supplementary Tables 3–5 summarize the expression levels of all 2,020 genes associated with congenital and developmental disorders.

Both GTEx fibroblasts and AF cells had a higher proportion of well-expressed genes across all 11 disease gene classes than GTEx whole blood (Fig. 2e). There were statistically significant *p*-values for all 11 gene classes except two, which was likely due to the smaller number of genes contained within those gene classes (15 and 30 genes). The majority of well-expressed genes had higher expression in terms of median and maximum TPM in fibroblasts and AF cells than in whole blood in all gene classes. Fibroblasts had the highest proportion of well-expressed genes across all classes, except for “familial nonsyndromic congenital heart disease”, in which AF cells had the highest proportion of well-expressed genes (though not significant).

A Gene Ontology (GO) enrichment analysis^37^ of 81 genes well-expressed in AF cells but not expressed or lowly expressed in whole blood and fibroblasts grouped 244 significant GO terms (FDR *q*-value < 0.1) into a network of 20 clusters (Supplementary Table 6). All 20 clusters contained terms related to early organ or system development and morphogenesis. The top three enriched clusters (*q*-value < 3.9 × 10^−4^) were represented by the GO terms (i) embryonic morphogenesis, (ii) cardiac chamber development, and (iii) sensory organ development.

Because GO analysis indicated potential enrichment of cardiac genes in AF cells, we further compared the overall expression of these genes (well-expressed and lowly expressed; *n* = 33) in whole blood, fibroblasts and AF cells. The overall number of expressed cardiac genes was comparable between fibroblasts (20/33) and AF cells (19/33), but was much higher in whole blood (11/33) (Supplementary Fig. 3a). Among the 33 cardiac genes, 45% (15/33) were considered as well-expressed in AF cells, followed by 39% (13/33) in fibroblasts and 21% (7/33) in whole blood (Supplementary Fig. 4a).

We performed a similar comparison with 423 skeletal genes as skeletal anomalies constitute one of the top phenotypes with the greatest proportions of diagnostic genetic variant during prenatal WES as represented by several large-scale prenatal cohort studies^34,38,39^. Moreover, it is one of the common phenotype with a genetic diagnosis among previous postnatal WES studies^40,41^. The overall number of well-expressed and lowly expressed skeletal genes was comparable in fibroblasts (339/423) and AF cells (337/423), but was much higher than that in whole blood (236/423) (Supplementary Fig. 3b). Fibroblasts, AF cells and blood had 61% (259/423), 50% (210/423), and 18% (78/423) well-expressed skeletal genes (Supplementary Fig. 4b).

### Use of AF cells RNA-seq in prenatal diagnoses

#### Family 1

**Fig. 3:**
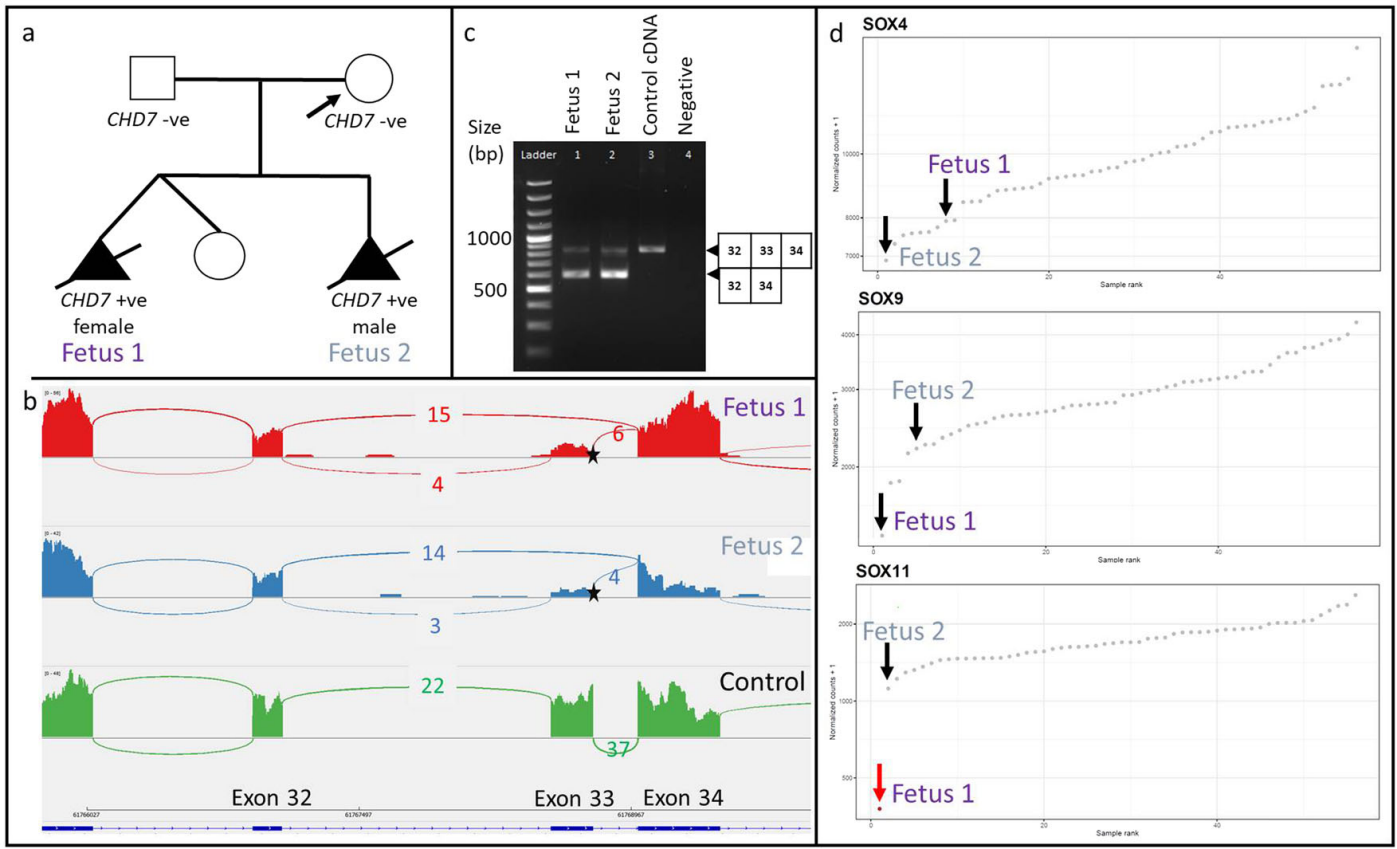
Family 1 with the *CHD7* splicing variant NM_017780.4: c.7164 + 1 G > A. **a** Pedigree for family 1. **b** Sashimi plot from integrative genome viewer (IGV) showing the skipping of exon 33 with no alternative splice site in intron 33 for both affected fetuses in comparison with a control. Only one (upper) line extended from exon 32 to 34. The location of the variant is shown with a star. **c** RT-PCR results with primer pairs spanning exons 32 to 34 showing the expected size of 827 bp. The expected size for the exon 33 deletion = 600 bp, demonstrating a heterozygous skipping of exon 33 in fetuses 1 and 2. The square boxes on the right indicate the exon number. **d** Expression rank plots for three genes of the SOX family. The X-axis represents all 52 AF cells, ranked in ascending order of the normalized read counts in the corresponding gene. The Y-axis represents the normalized read counts. NM_017780.4:c.7164 + 1 G > A in *CHD7* could be affecting the expression of downstream genes such as *SOX4*, *SOX9* and *SOX11*, as these genes were expressed less in fetuses 1 and 2 when compared to all 52 samples. The red dot/arrow indicates aberrant expression of that *SOX11* (FDR ≤ 0.05) in fetus 1.

The fetus of a 34-year-old Chinese woman was suspected to have a double aortic arch and left subclavian artery upon ultrasonography at 16 weeks of pregnancy. During the previous dichorionic diamniotic twin pregnancy of the patient, one fetus had had a double aortic arch and cleft lip with selective fetocide (Fig. 3a). Trio WES for index pregnancy revealed a *de novo* heterozygous splicing variant NM_017780.4:c.7164 + 1 G > A in *CHD7* (MIM 608892). The same variant had also been identified in the previously affected fetus by Sanger sequencing, in which further tests later confirmed that the variant had been caused by paternal germline mosaicism. *CHD7* is associated with CHARGE syndrome (MIM 214800). According to the American College of Medical Genetics (ACMG) Variant Interpretation guidelines, this splicing variant is classified as likely pathogenic (PVS1_Moderate, PS2, and PM2). Although the variant has been reported in the literature, the molecular consequences of either skipping exon 33 or creating an alternative splice site within intron 33 remain uncertain^42^.

DROP revealed 11 aberrant splicing (AS) outliers for each fetus (Supplementary Table 7). *CHD7* was detected in both affected fetuses as a splicing outlier (|Δψ| of 0.62 in fetus 1 and 0.66 in fetus 2 [FDR ≤ 0.05]). Our RNA-seq data revealed that exon 33 was skipped with no alternative splice site in intron 33 (Fig. 3b). This indicated the synthesis of a shortened, partially or non-functional *CHD7*. These findings were confirmed by RT-PCR using primer pairs spanning exons 32–34 (Fig. 3c). Integrating the recently updated guidelines for clinical interpretation of variant pathogenicity using RNA-seq data from Smirnov et al.^43^, PS3 evidence can be used after AF cells RNA-seq to upgrade the variant from likely pathogenic to pathogenic.

DROP revealed two aberrant expression (AE) outliers for fetus 1 and one expression outlier for fetus 2 (Supplementary Table 8); however, only *SOX11* was clinically relevant in fetus 1. The expression of *CHD7* was low with a median TPM value of 4.5 for fetus 1 and 2.4 for fetus 2 (Supplementary Fig. 5a). Because *CHD7* encodes an ATP-dependent chromatin modifier involved in transcription regulation during the early stages of development of various tissues^44^, the splice site variant could possibly lead to a loss of function in *CHD7* as a chromatin remodeler and gene transcription regulator. Consistent with previous findings that *CHD7* and downstream genes are the primary causes of CHARGE syndrome^45,46^, our RNA-seq data showed that the expression of *SOX4*, *SOX9*, and *SOX11* in both fetuses was among the lowest of the whole cohort (Fig. 3d). Different SOX family members are regulated by CHD7^47^. *SOX4* and *SOX11* are known direct targets of *CHD7*^48^, and the CHD7/SOX9 signalling pathway is essential for neural crest cell (NCC) specification and migration^49^. A recent transcriptomic study by Yan et al. (2020) also showed that *CHD7* fine-tunes the expression of a gene network that is critical for cardiac NCC development^50^, which is consistent with the hypothesis that the clinical features of CHARGE may result from the abnormal development of NCCs. This would explain the abnormal cardiac phenotype of the patient. However, further knockout functional studies are needed to validate the exact mechanisms underlying the double aortic arch.

#### Family 2

The fetus of a 36-year-old Chinese woman exhibited the coarctation of the aorta, a hypoplastic left heart, and a diaphragmatic hernia upon ultrasonography during the pregnancy. The family opted for medical termination post-mortem, which revealed a bilateral congenital diaphragmatic hernia, pulmonary hypoplasia, double outlet right ventricle, ventricular septal defect, and ambiguous external genitalia. Trio WES revealed a *de novo* heterozygous *MYRF* (MIM 608329) splicing variant in NM_001127392.1:c.2014-1 G > A, that was associated with cardiac-urogenital syndrome (MIM 618280) and inherited in an autosomal dominant manner. According to the ACMG Variant Interpretation Guidelines, this splicing variant was classified as pathogenic (PVS1, PS2, and PM2) and has not been previously reported; hence, its molecular mechanisms remain unknown. An in silico tool predicted disruption of reading frame leading to nonsense-mediated decay (NMD).

**Fig. 4:**
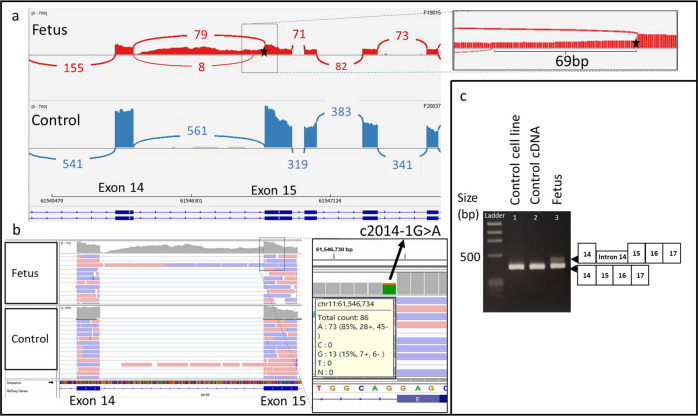
Family 2 with the *MYRF* splicing variant. **a** Sashimi plot, and **b** IGV profile showing an in frame 69-bp intron 14 retention, and a complete intron 14 retention, activated by NM_001127392.1:c.2014-1 G > A. This was not observed in another AF cell control. The splicing variant was also absent in gnomADv2.1.1. The location of the variant has been indicated with a star. **c** RT-PCR with primer pairs spanning exons 14 to 17 with an expected size of 364 bp for the wildtype and 433 bp for the transcript with partial intron retention. The squared boxes on the right indicate the exon number.

DROP revealed seven splicing outliers for this sample, with no expression outlier (Supplementary Tables 7 and 8). *MYRF* was detected as an AS outlier (|Δψ| at 0.31, FDR ≤ 0.05). The RNA-seq data showed that this variant not only caused complete intron 14 retention leading to NMD in some transcripts, but also caused an in-frame 69-bp retention of intron 14 in other transcripts (Fig. 4a, b), revising the functional consequence predicted by in silico tool. These aberrant splicing resulted in an abnormal MYRF protein with 1) 23 additional amino acids between p.671–672, and 2) truncation due a STOP codon within intron 14. These features may have crippled the intramolecular chaperone domain and blocked the auto-cleavage of MYRF as reported earlier for the variant NM_001127392.2:c.2036 T > C p.Val679Ala^51^. RT-PCR confirmed the presence of longer *MYRF* transcripts (Fig. 4c). TA cloning and Sanger sequencing confirmed the in-frame insertion at positions c.2014-69 to c.2014-1.

NM_001127392.1:c.2014-1 G > A included an in-frame variant that did not affect *MYRF* mRNA expression (Supplementary Fig. 5b). Myelin genes such as *MBP*, *MOG*, *MAG*, *DUSP15*, and *PLP1* are activated by *MYRF* during oligodendrocyte maturation^52^; however, because the expression of these myelin genes in AF cells, fibroblasts, and whole blood was low, we could not conclude whether the functionality of *MYRF* had been compromised. Further downstream protein studies or RNA-seq in different tissue types are needed to understand the detailed mechanisms of the disease; however, specimen choice especially in prenatal diagnosis is usually limited.

#### Family 3

A 47-year-old pregnant Chinese woman who had opted for in vitro fertilization with a donor egg, was found to have a high-risk first trimester Down syndrome screening test result. An anomaly ultrasonography scan revealed acromelia, contractures (arthrogryposis), and short long bones. After medically terminating the pregnancy, radiography analysis of the abortus revealed hypomineralization of the skull bone, with fractures of the long bones. Duo WES of the fetus and biological father revealed a heterozygous splicing variant (NM_000089.3:c.2133 + 5 G > A) in *COL1A2* (MIM 120160) that was associated with osteogenesis imperfecta Type II (MIM 166210). According to the ACMG Variant Interpretation guidelines, this splicing variant was classified as a VUS (PM2 and PM6). NM_000089.3:c.2133 + 5 G > A in *COL1A2* has not been previously reported, and its molecular mechanisms remain unknown.

**Fig. 5:**
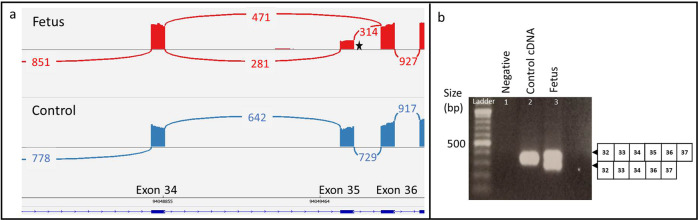
Family 3 with the *COL1A2* variant (NM_000089.3:c.2133 + 5 G > A). **a** Sashimi plot showing the skipping of exon 35. The variant location is indicated with a star. **b** RT-PCR with primer pairs spanning exons 32 to 37 with an expected size of 319 bp for the wildtype and 265 bp for the transcript with the exon 35 deletion. The squared boxes on the right indicate the exon number.

DROP revealed 15 splicing outliers for this sample with no expression outlier (Supplementary Tables 7, 8). *COL1A2* was detected as a splicing outlier (|Δψ| at 0.59, FDR ≤ 0.05). RNA-seq data confirmed that this variant caused the skipping of exon 35 (Fig. 5a) with the same consequence as reported for c.2133 + 6 G > A^53^. TA cloning and Sanger sequencing confirmed the skipping of exon 35 (Fig. 5b). With this new evidence from AF cells RNA-seq analysis, together with the recently updated guidelines^43^, NM_000089.3:c.2133 + 5 G > A can be considered as pathogenic rather than a VUS (PS3, PM2, PM4 and PM6).

NM_000089.3:c.2133 + 5 G > A did not affect *COL1A2* mRNA expression (Supplementary Fig. 5c), nor did it affect the expression of bone development-related genes such as *RETN, FCGR3B, ALPL, AEBP1, and LIMS2*. However, because not all of these bone development-related genes are well-expressed in AF cells (only one out of these five genes was considered as well-expressed in AF cells); further functional studies using other tissue types are needed to delineate the exact mechanisms of the variant.

## Discussion

In recent years, RNA-seq has become an important tool in genetic diagnostics as it contributes to the interpretation of VUS unsolved by WES or WGS. Blood^16–20^, skin fibroblasts^6,15,17–19,21^, and muscle^14,15,17^ are the most commonly used clinically accessible tissues. Skin fibroblasts are preferred as they can capture a high proportion of expressed genes compared to blood^18^. However, fibroblasts require invasive skin biopsies and are therefore disadvantageous. Available specimen choice is limited in prenatal diagnosis. The most common prenatal diagnostic techniques are amniocentesis, CVS, and, to a lesser extent, fetal blood sampling^54^. Enzensberger et al.^55^ retrospectively analyzed 6256 patients who underwent invasive diagnostics tests between 1993 and 2011 and found a procedure-related fetal loss rate of 0.4% after amniocentesis and of 1.1% and 0.4% after CVS and FBS, respectively. Because amniocentesis presents a low risk for miscarriage and is a routine procedure in most prenatal diagnostic laboratories, obtaining AF samples is more feasible for prenatal RNA-seq.

We evaluated the use of AF cells as clinically accessible tissue for RNA-seq in genetic diagnostics. We showed that amniocytes have a gene expression profile similar to fibroblasts due to their similar embryonic origin. The number of well-expressed genes in AF cells was comparable to that in fibroblasts and much higher than that in whole blood in different disease categories. The advantage of AF cells RNA-seq was further exemplified in family 1, in which we found that the *CHD7* splicing variant affected downstream SOX families, such as *SOX9*. Information regarding *SOX9* can only be obtained if AF cells are used because this gene is not expressed in the blood or fibroblasts. Similarly, transcriptomic information regarding *MYRF* in family 2 could only be investigated using AF cells (Supplementary 5B). Although *COL1A2* was highly expressed in fibroblasts in family 3, with a median TPM value > 4000, it was also well-expressed in AF cells, with a median TPM value of 37. However, this gene was barely expressed in whole blood, with a median TPM value of 0.62 (Supplementary Fig. 5c).

As in a previous study^18^, our PCA plot demonstrated less variability in fibroblasts than in the blood. Additionally, we observed less variability in AF cells, implying that both AF cells and fibroblasts can yield robust and consistent results for clinical diagnostics.

Using AF cells RNA-seq with a recently established analytical pipeline, DROP, we successfully detected the disease-causing genes *CHD7, MYRF*, and *COL1A2* as significant splicing outliers in three families (Figs. 3–5). The rationale of this approach is based on the comparison of one patient sample against the rest of the samples in the cohort as internal controls. Therefore, the sequencing read depth of this study was aim high at 100 million reads, for the proper detection of aberrant outliers^22,43^. This comparison allows for the identification of significant aberrant outliers, AE, AS and monoallelic expression (MAE), to stand out due to the causal genetic defects in a sample. Genes with causal variants are not necessarily picked up by all three (AE, AS and MAE) pipelines. While we demonstrated the ability of AF cells RNA-seq and our adapted DROP v1.1.0 pipeline to identify pathogenic splicing variants in three families, their corresponding expression is not being affected. According to a recent paper by Smirnov et al.^43^ that overviewed eight recent studies applying large-scale RNA‐seq, 64%, 62% and 27% of the diagnosed cases were identified by AE, AS and MAE, respectively, half of the splicing outliers did not lead to aberrant expression. In addition, detection as expression outliers could only provide strong evidence of pathogenicity in biallelic splice variants but not monoallelic splice variants; therefore, for a monoallelic splice variant, abnormal expression is not a useful functional consequence to support its pathogenicity. On the other hand, detection as splicing outlier could provide strong evidence of pathogenicity to monoallelic splice variants. Recently, efforts have been made to incorporate RNA-seq evidence into the ACMG Variant Interpretation Guidelines^43,56,57^. Using a set of 723 benign variants and 198 pathogenic variants with known aberrant RNA phenotypes such as aberrant splicing or expression, Smirnov et al. calculated the odds of pathogenicity to evaluate different thresholds for assessing variant pathogenicity strength, with the aim of integrating data from DROP to the ACMG Variant Interpretation Guidelines^43^. Adhering to their recommendation, we identified *CHD7*, *MYRF* and *COL1A2* as significant splicing outliers based on effect size and significance threshold (*CHD7*: |Δψ| =0.63/0.66, FDR ≤ 0.05; *MYRF*: |Δψ| =0.31, FDR ≤ 0.05; *COL1A2*: |Δψ| =0.59, FDR ≤ 0.05), providing strong functional evidence (PS3) to support the pathogenicity of the splice variants identified in these genes. Integrating the above threshold to our cases, the splicing variants observed in families 1 and 3 can be further upgraded to pathogenic. Besides, AF cells RNA-seq revised the functional consequences of intron retention for the *MYRF* variant, revealing that the in silico prediction is not 100% accurate.

We found that AF cells were enriched with genes involved in early development, especially heart, as it is the first functioning organ in developing embryos^58^. The GO results were consistent with our finding that AF cells may be the better choice among the clinically accessible tissues for investigating VUSs associated with congenital cardiac diseases, as AF cells have a high number of well-expressed cardiac-related genes (Supplementary Figs. 3a, 4a). Although expanding the gene expression analysis to a cardiac discovery panel (n = 1628) with known or potential associations with heart development and cardiac muscle structure^59^ did not reveal a better performance of AF cells than skin fibroblasts, their results were comparable, with AF cells expressing 52% and fibroblasts expressing 56% of highly expressed genes.

To the best of our knowledge, this is a pioneer proof-of-concept study to demonstrate the clinical utility of AF cells RNA-seq as a prenatal diagnostic tool and for the interpretation of VUSs. Our study highlights the advantage of storing AF after prenatal tests so that they can also be used for postnatal genetic diagnostics if required. Furthermore, in view of the multipotency of stem cells (SCs) from AF to differentiate into all embryonic germ lineages^60^, storing AF can help elucidating disease consequence and validating candidate genes. Thus, storing AF SCs may have potential therapeutic implications in the future^61^.

In sync with the Developmental Genotype-Tissue Expression (dGTEx) initiative (https://www.genome.gov/Funded-Programs-Projects/Developmental-Genotype-Tissue-Expression) from the National Human Genome Research Institute (NHGRI) in which RNA-seq will be performed on whole tissues and in single cell or homogeneous cell populations collected from neonatal and paediatric donors in a post-mortem setting, and other studies that utilize single-cell technology in profiling gene expression pattern during the prenatal period^62–64^, our data will serve as early preliminary data in the field until the dGTEx is fully developed and accessible in 2026. Over 85% of individuals in the GTEx are of European descent^25^. Currently, we do not know the population distribution of dGTEx; nonetheless, additional samples from underrepresented groups would be beneficial. Therefore, this dataset not only serves as one of the few AF cells expression datasets, but also a non-European cohort to provide insight into AF cells RNA-seq for the development of expression profiles and further embryological and fetal characterization studies.

One of the inevitable limitations of RNA-seq studies is that the results are highly dependent on the tissue source. Among the 2020 curated genes, a few were only expressed in fibroblasts or blood but not in AF cells (full list available in Supplementary Table 4). *PIK3CA*, for example, was highly expressed in fibroblasts, and *KCNQ1* was highly expressed in the blood. In specific situations where the VUS of these genes need to be studied, fibroblasts or blood samples may be a better option than AF. Regardless, various disease genes, such as *ARID1B, SCN2A*, and *SATB2*, which were three of the top diagnostic genes in the DDD study^35^, are not well-expressed in AF cells, fibroblasts, or whole blood. In fact, there are also transcript isoform-specific variant effects. Even though a gene is well expressed, it does not mean all exons or transcripts are equally expressed and some pathogenic variants could lie on those regions. Further transcript-based studies would be needed to investigate if there are any clinically relevant isoforms not expressed in AF cells.

Although recent studies have proposed the transdifferentiation of accessible tissues into specific tissue types^17^ or the use of orthogonal method such as short amplicon RT-PCR to detect splice variant in lowly-expressed gene^65^, others have utilized CRISPR/Cas9 to selectively deplete unwanted overabundant sequences from existing RNA-seq libraries to increase the sensitivity of targeted genes^66^. Nonetheless, these extra manipulation steps may not be cost-effective, easily accessible, or adaptable for clinical laboratories. Because prior knowledge of specific targeted genes (e.g., *PIK3CA* or *KCNQ1*) is usually limited, choosing a tissue such as AF that includes a relatively large number of expressed genes represents a logical starting point for RNA-seq, especially considering the limited specimen choices for prenatal diagnostics. Another inherent limitation of RNA-seq is that various disease-causing missense variants may not affect or may only have a subtle effect on transcript expression. Nonetheless previous studies have predicted that 25% of missense variants would affect gene expression or cause abnormal splicing^67–69^, and Smirnov et al.^43^ found that 10% of missense variants indeed exhibit RNA phenotypes. Finally, AF cells may not be readily available for all postnatal patients as cells cultured from AF samples may not be stored after prenatal tests or may not exist at all. If AF RNA-seq is chosen as a postnatal diagnostic tool in clinical settings, a proper AF banking system should be set up in prenatal laboratories.

Despite these limitations, RNA-seq serves as the only diagnostic tool to reveal functional consequences for variants affecting splicing and gene expression, especially for those in the non-coding region. This remains largely unpredictable from the DNA sequence alone. With advances in the field of machine learning, prediction tools such as SpliceAI^70^ and MMSplice^71^ for aberrant splicing and Enformer^72^ or Expecto^73^ for aberrant expression are constantly emerging with improved algorithms; however, the precision of existing tools is not yet high enough to provide accurate diagnoses. Currently this is only a preliminary study with a small sample size and a focus on monoallelic splicing variants, we aim to expand this study by recruiting more prenatal and postnatal cases to identify the causative variants that remain unresolved by WES and WGS. A larger sample size would help us achieve a higher statistical power to detect outliers^22^ and the inclusion of different variant types, such as biallelic splicing or other biallelic/monoallelic non-coding variants, would allow us to fully evaluate the usefulness of AF cells RNA-seq by providing extra evidence for variant interpretation. AF cells RNA-seq may further be implemented in a clinical setting as a complementary diagnostic tool to WES or WGS, to provide functional evidence for variants affecting expression or splicing that cannot be explained by DNA sequencing.

In this study, we evaluated the expression of clinically significant genes in AF cells compared with that in fibroblasts and blood. We found AF cells to be a reasonable choice for RNA-seq, especially for 1) prenatal diagnoses where AF is readily available, 2) postnatal diagnoses in patients whose mothers have undergone amniocentesis during the prenatal period, with advantages over fibroblasts and whole blood as it prevents further invasive procedures. We further evaluated the application of DROP for prenatal genetic diagnoses using AF cells RNA-seq with three examples of clinical cases involving splicing variants. The pipeline detected *CHD7, MYRF*, and *COL1A2* as splicing outliers and revealed their underlying molecular consequences. These findings shed light on the corresponding disease aetiologies and help to elucidate the pathogenicity of VUSs.

To prevent unnecessary invasive sampling while maintaining the number of well-expressed genes in a sample, we suggest the use of AF cells, if available, to replace fibroblasts or blood for RNA-seq in postnatal settings to maximize the number of testable genes. AF cells RNA-seq may lead to a new era of non-invasive diagnostics for postnatal patients with readily available AF. These few cases highlighted in this study demonstrate the value of using AF cells RNA-seq as an alternative means to strengthen variant interpretation, especially for the interpretation of VUSs. The ascertainment of VUS using AF cells RNA-seq from a clinical standpoint can facilitate prompt Mendelian disease diagnosis and improve patient care in both prenatal and postnatal settings.

## Methods

### Sample collection

AF was collected from pregnant women under the care of Tsan Yuk Hospital, Hong Kong, who opted for amniocenteses during their second trimester based on either high-risk Down syndrome screening test results or abnormal ultrasound findings. Maternal cell contamination was determined based on quantitative analyses of informative short tandem region (STR) markers between the mother and AF cells, with a detection limit of 5%. Samples with maternal cell contamination were excluded from the study.

For nondiseased samples, fetuses with abnormal ultrasound findings were excluded. Chromosomal microarray confirmed whether the fetus was euploid and a healthy pregnancy was expected to continue.

For validation samples, we studied four pregnancies from three families (Family 1 had two affected pregnancies) with known splicing variants detected by WES. AF cells from these affected pregnancies were either retrieved from the stored sample (*n* = 1 from a previous pregnancy in 2017) or were freshly obtained during prenatal testing (*n* = 3).

### RNA extraction and sequencing

AF cells were grown in 25-cm^2^ culture flasks until 70% confluency. The cultured amniocytes were lysed in TRIzol^TM^ Reagent (Thermo Fisher Scientific, Waltham, Massachusetts, USA) for RNA extraction according to the manufacturer’s instructions. RNA with an RNA integrity number (RIN) ≥ 8 was used for non-strand-specific RNA library preparation using the poly-A tailed capture method with the KAPA mRNA HyperPrep Kit (Roche Diagnostics, Tucson, AZ, USA) according to manufacturer’ protocol. RNA libraries were sequenced as 150-bp paired-end runs on an Illumina NovaSeq 6000 platform (Illumina, San Diego, CA, USA) with a minimum 100 million reads per sample at the Center for PanorOmic Sciences (CPOS), University of Hong Kong.

### RNA-seq analysis

Fastq files were mapped to the hg19/GRCh37 human reference genome using STAR (v2.5.2a) in two-pass mode for the detection of novel splice junctions. The duplicates were marked using Picard (https://broadinstitute.github.io/picard/). The sequencing data were processed with DROP v1.1.0, which is a workflow that integrates statistical algorithms to detect significant aberrant gene expression events, including expression levels, splicing, and the monoallelic expression of rare variants^22,74,75^.

AE was detected using OUTRIDER^75^ in DROP. Read counts that significantly deviated from the model were detected as outliers, taking into consideration the calculated multiple-testing corrected *p*-values (false discovery rate [FDR]), z-scores, and fold changes. Genes with an FDR ≤ 0.05 were defined as aberrantly expressed.

AS was detected using FRASER^74^ in DROP. FRASER utilizes an annotation-free splicing algorithm that allows for the detection of novel splicing variants. In FRASER, split (indicating an exon–exon junction) and non-split (spanning the exon–intron boundary for both donor and acceptor sites) reads were counted. Based on the corresponding ratio between the split and non-split reads, splicing metrics quantifying alternative acceptors (ψ_5_), alternative donors (ψ_3_), and splicing efficiencies of donors (θ_5_) and acceptors (θ_3_) were computed. Introns with an FDR ≤ 0.05 and |Δψ| or |Δθ| ≥ 0.3 were defined as aberrantly spliced.

### DROP v1.1.0 validation

To confirm proper implementation of DROP v1.1.0, transcripts per million (TPM) values were compared between GTEx control fibroblasts (Supplementary Fig. 2), in which TPM values were obtained directly online, and in-house generated TPM values from BAM files of 76 control fibroblasts samples. BAM files from 76 non-diseased skin fibroblasts RNA-seq samples downloaded from the NCBI database of Genotypes and Phenotypes (dbGaP) with study accession phs000424.v8.p2 were processed using the adapted DROP v1.1.0 pipeline for benchmark comparison. The conversion of TPM from fragments per kilobase of transcript per million mapped reads (FPKM) generated in DROP was performed using R (v4.1.2).

### Transcriptome comparisons

TPM values from 504 and 755 publicly available reference gene expression samples collected from whole blood and cell-cultured fibroblasts, respectively, were obtained from the V8 data from the GTEx portal on 5 January 2022, with the help of the GTEx portal staff. PCA plots were generated using R (v4.1.2). Genes with a median TPM value of ≥10 were considered well-expressed. Genes with a median TPM values <1 were considered not expressed. Genes with median TPM values between (1 ≤ TMP < 10) were considered lowly expressed.

### Gene lists for evaluating the expression of clinically relevant genes in AF cells RNA-seq

We generated a list of genes with clinical implications for congenital and developmental anomalies by combining 117 genes associated with prenatal presentation identified by Lord et al.^34^ within their cohort of 610 fetuses in the large-scale PAGE study, with 1914 developmental disorder genes from the DDG2P_v2.2 panel (www.ebi.ac.uk/gene2phenotype, accessed in February 2020). Eleven genes were redundant; therefore, 2020 genes were included (Supplementary Table 1).

The 11 gene lists in Fig. 2d that contain genes for holoprosencephaly, familial non-syndromic congenital heart disease (also shown in Supplementary Figs. 3c, 4a), deafness, and congenital structural abnormalities, congenital myopathy, congenital anomalies of kidney and urinary tract (CAKUT), fetal hydrops, rare multisystem ciliopathy disorders, limb disorders, skeletal dysplasia (also shown in Supplementary Figs. 3d, 4b), and fetal anomalies were extracted from the Genomics England panel app (https://panelapp.genomicsengland.co.uk/), accessed on 18 February 2022. These gene lists were chosen based on clinical relevance commonly seen in our genetic clinic.

### Variant identification, interpretation and validation

For the three validation cases with previous WES results, Online Mendelian Inheritance in Man (OMIM) disease-associated genes were prioritized. Manual curation was performed with reference to the genotype-phenotype correlation in each cases, allele frequency (<1% in gnomAD v.2.1.1), inheritance pattern, GTEx TPM values, and relative TPM values across all AF samples. The pathogenicity of variants was classified according to the ACMG Variant Interpretation Guidelines^76,77^. All identified aberrant splicing events were inspected using IGV^78^. Splicing variants were further confirmed using reverse transcription polymerase chain reaction (RT-PCR) and subsequent cDNA sequencing. The respective cDNA was amplified by RT-PCR using a one-step RT-PCR Kit (Thermo Fisher Scientific). Specific primer pairs (Supplementary Table 2) spanning the targeted exons were designed to validate the molecular consequences of these splicing variants.

### Statistics

Statistical differences between the number of well-expressed genes in AF cells versus GTEx whole blood, and GTEx fibroblasts versus GTEx whole blood were calculated using Fisher’s exact test in R (v4.1.2). Measurements were taken from distinct samples.

### Study approval

All participants provided written informed consent. The study was approved by the institutional review board of the University of Hong Kong/Hospital Authority Hong Kong West Cluster (UW11-190 and UW12‐211).

### Reporting summary

Further information on research design is available in the Nature Portfolio Reporting Summary linked to this article.

## Supplementary information

supplementary

Reporting Summary Checklist

## Data Availability

The raw RNA-sequencing datasets for this article are not publicly available due to concerns regarding minor participant/patient anonymity. Requests to access the datasets should be directed to the corresponding authors. Data available from the corresponding authors will be de-identified before the data is handed over to qualified researchers.

## Code availability

The codes used to process the data in this study were deposited in GitHub (https://github.com/gagneurlab/drop) with no access restrictions.
