# Supplementary material for: Diagnostic potential of the amniotic fluid cells transcriptome in deciphering mendelian disease: a proof-of-concept
Source: NPJ Genom Med. 2022 Dec 28;7:74. doi: 10.1038/s41525-022-00347-4 (PMC9797484; doi:10.1038/s41525-022-00347-4)
Supplement: Supplementary file 1 — supplementary [file 41525_2022_347_MOESM1_ESM.pdf]

Supplementary Table 1. Details of the 2,020 curated genes associated with congenital and developmental disorders.

Gene Name	Ensembl ID(GRch37)	Source
AAAS	ENSG00000094914	DDG2P_v2.2 panel
AARS	ENSG00000090861	DDG2P_v2.2 panel
AASS	ENSG00000008311	DDG2P_v2.2 panel
ABCA12	ENSG00000144452	Lord et. al.
ABCB11	ENSG00000073734	DDG2P_v2.2 panel
ABCB6	ENSG00000115657	DDG2P_v2.2 panel
ABCB7	ENSG00000131269	DDG2P_v2.2 panel
ABCC6	ENSG00000091262	DDG2P_v2.2 panel
ABCC8	ENSG00000006071	Lord et. al.
ABCC9	ENSG00000069431	DDG2P_v2.2 panel
ABCD1	ENSG00000101986	DDG2P_v2.2 panel
ABCD4	ENSG00000119688	DDG2P_v2.2 panel
ABHD5	ENSG00000011198	DDG2P_v2.2 panel
ABL1	ENSG00000097007	DDG2P_v2.2 panel
ACAD9	ENSG00000177646	DDG2P_v2.2 panel
ACADM	ENSG00000117054	DDG2P_v2.2 panel
ACADS	ENSG00000122971	DDG2P_v2.2 panel
ACADVL	ENSG00000072778	DDG2P_v2.2 panel
ACAN	ENSG00000157766	DDG2P_v2.2 panel
ACAT1	ENSG00000075239	DDG2P_v2.2 panel
ACBD6	ENSG00000230124	DDG2P_v2.2 panel
ACE	ENSG00000159640	Lord et. al.
ACO2	ENSG00000100412	DDG2P_v2.2 panel
ACOX1	ENSG00000161533	DDG2P_v2.2 panel
ACP5	ENSG00000102575	DDG2P_v2.2 panel
ACSL4	ENSG00000068366	DDG2P_v2.2 panel
ACTA1	ENSG00000143632	DDG2P_v2.2 panel
ACTA2	ENSG00000107796	DDG2P_v2.2 panel
ACTB	ENSG00000075624	DDG2P_v2.2 panel
ACTG1	ENSG00000184009	DDG2P_v2.2 panel
ACTG2	ENSG00000163017	Lord et. al.
ACTL6B	ENSG00000077080	DDG2P_v2.2 panel
ACVR1	ENSG00000115170	DDG2P_v2.2 panel
ACVR2B	ENSG00000114739	DDG2P_v2.2 panel
ACY1	ENSG00000243989	DDG2P_v2.2 panel
ADA	ENSG00000196839	DDG2P_v2.2 panel
ADAMTS10	ENSG00000142303	Lord et. al.
ADAMTS17	ENSG00000140470	Lord et. al.
ADAMTS18	ENSG00000140873	DDG2P_v2.2 panel
ADAMTS9	ENSG00000163638	DDG2P_v2.2 panel
ADAMTSL2	ENSG00000197859	Lord et. al.
ADAR	ENSG00000160710	DDG2P_v2.2 panel
ADGRG1	ENSG00000205336	DDG2P_v2.2 panel
ADGRG6	ENSG00000112414	DDG2P_v2.2 panel
ADK	ENSG00000156110	DDG2P_v2.2 panel
ADNP	ENSG00000101126	DDG2P_v2.2 panel
ADPRHL2	ENSG00000116863	DDG2P_v2.2 panel
ADRA2B	ENSG00000274286	DDG2P_v2.2 panel
ADSL	ENSG00000239900	DDG2P_v2.2 panel
AFF2	ENSG00000155966	DDG2P_v2.2 panel
AFF3	ENSG00000144218	DDG2P_v2.2 panel
AFF4	ENSG00000072364	DDG2P_v2.2 panel
AFG3L2	ENSG00000141385	DDG2P_v2.2 panel
AGA	ENSG00000038002	DDG2P_v2.2 panel
AGK	ENSG00000006530	DDG2P_v2.2 panel
AGL	ENSG00000162688	DDG2P_v2.2 panel
AGPAT2	ENSG00000169692	Lord et. al.
AGPS	ENSG00000018510	DDG2P_v2.2 panel

AGRN	ENSG00000188157	Lord et. al.
AGTR2	ENSG00000180772	DDG2P_v2.2 panel
AGXT	ENSG00000172482	DDG2P_v2.2 panel
AHDC1	ENSG00000126705	DDG2P_v2.2 panel
AHI1	ENSG00000135541	DDG2P_v2.2 panel
AIFM1	ENSG00000156709	DDG2P_v2.2 panel
AIMP1	ENSG00000164022	DDG2P_v2.2 panel
AIPL1	ENSG00000129221	DDG2P_v2.2 panel
AIRE	ENSG00000160224	DDG2P_v2.2 panel
AK2	ENSG00000004455	DDG2P_v2.2 panel
AKR1D1	ENSG00000122787	DDG2P_v2.2 panel
AKT1	ENSG00000142208	DDG2P_v2.2 panel
AKT3	ENSG00000117020	DDG2P_v2.2 panel
ALAD	ENSG00000148218	DDG2P_v2.2 panel
ALDH18A1	ENSG00000059573	DDG2P_v2.2 panel
ALDH1A3	ENSG00000184254	DDG2P_v2.2 panel
ALDH3A2	ENSG00000072210	DDG2P_v2.2 panel
ALDH4A1	ENSG00000159423	DDG2P_v2.2 panel
ALDH5A1	ENSG00000112294	DDG2P_v2.2 panel
ALDH7A1	ENSG00000164904	DDG2P_v2.2 panel
ALDOA	ENSG00000149925	DDG2P_v2.2 panel
ALDOB	ENSG00000136872	DDG2P_v2.2 panel
ALG1	ENSG00000033011	DDG2P_v2.2 panel
ALG11	ENSG00000253710	DDG2P_v2.2 panel
ALG12	ENSG00000182858	DDG2P_v2.2 panel
ALG13	ENSG00000101901	DDG2P_v2.2 panel
ALG2	ENSG00000119523	DDG2P_v2.2 panel
ALG3	ENSG00000214160	DDG2P_v2.2 panel
ALG6	ENSG00000088035	DDG2P_v2.2 panel
ALG8	ENSG00000159063	DDG2P_v2.2 panel
ALG9	ENSG00000086848	DDG2P_v2.2 panel
ALMS1	ENSG00000116127	DDG2P_v2.2 panel
ALPL	ENSG00000162551	DDG2P_v2.2 panel
ALS2	ENSG00000003393	DDG2P_v2.2 panel
ALX1	ENSG00000180318	DDG2P_v2.2 panel
ALX3	ENSG00000156150	DDG2P_v2.2 panel
ALX4	ENSG00000052850	DDG2P_v2.2 panel
AMER1	ENSG00000184675	DDG2P_v2.2 panel
AMPD2	ENSG00000116337	DDG2P_v2.2 panel
AMT	ENSG00000145020	DDG2P_v2.2 panel
ANAPC1	ENSG00000153107	DDG2P_v2.2 panel
ANKH	ENSG00000154122	DDG2P_v2.2 panel
ANKRD11	ENSG00000167522	DDG2P_v2.2 panel
ANKRD26	ENSG00000107890	DDG2P_v2.2 panel
ANO5	ENSG00000171714	DDG2P_v2.2 panel
ANOS1	ENSG00000011201	Lord et. al.
ANTXR1	ENSG00000169604	DDG2P_v2.2 panel
AP1S2	ENSG00000182287	DDG2P_v2.2 panel
AP2M1	ENSG00000161203	DDG2P_v2.2 panel
AP3B1	ENSG00000132842	Lord et. al.
AP3B2	ENSG00000103723	DDG2P_v2.2 panel
AP4B1	ENSG00000134262	DDG2P_v2.2 panel
AP4E1	ENSG00000081014	DDG2P_v2.2 panel
AP4M1	ENSG00000221838	DDG2P_v2.2 panel
AP4S1	ENSG00000100478	DDG2P_v2.2 panel
APC2	ENSG00000115266	DDG2P_v2.2 panel
APOPT1	ENSG00000256053	DDG2P_v2.2 panel
APTX	ENSG00000137074	DDG2P_v2.2 panel
AR	ENSG00000169083	DDG2P_v2.2 panel
ARCN1	ENSG00000095139	DDG2P_v2.2 panel

ARFGEF2	ENSG00000124198	DDG2P_v2.2 panel
ARG1	ENSG00000118520	DDG2P_v2.2 panel
ARHGAP31	ENSG00000031081	DDG2P_v2.2 panel
ARHGEF6	ENSG00000129675	DDG2P_v2.2 panel
ARHGEF9	ENSG00000131089	DDG2P_v2.2 panel
ARID1A	ENSG00000117713	DDG2P_v2.2 panel
ARID1B	ENSG00000049618	DDG2P_v2.2 panel
ARID2	ENSG00000189079	DDG2P_v2.2 panel
ARL13B	ENSG00000169379	Lord et. al.
ARL14EP	ENSG00000152219	DDG2P_v2.2 panel
ARL3	ENSG00000138175	DDG2P_v2.2 panel
ARL6	ENSG00000113966	DDG2P_v2.2 panel
ARMC4	ENSG00000169126	DDG2P_v2.2 panel
ARMC9	ENSG00000135931	DDG2P_v2.2 panel
ARSA	ENSG00000100299	DDG2P_v2.2 panel
ARSB	ENSG00000113273	DDG2P_v2.2 panel
ARSE	ENSG00000157399	DDG2P_v2.2 panel
ARX	ENSG00000004848	DDG2P_v2.2 panel
ASAH1	ENSG00000104763	DDG2P_v2.2 panel
ASCC1	ENSG00000138303	DDG2P_v2.2 panel
ASCC3	ENSG00000112249	DDG2P_v2.2 panel
ASCL1	ENSG00000139352	DDG2P_v2.2 panel
ASH1L	ENSG00000116539	DDG2P_v2.2 panel
ASL	ENSG00000126522	DDG2P_v2.2 panel
ASNS	ENSG00000070669	Lord et. al.
ASPA	ENSG00000108381	DDG2P_v2.2 panel
ASPH	ENSG00000198363	DDG2P_v2.2 panel
ASPM	ENSG00000066279	DDG2P_v2.2 panel
ASS1	ENSG00000130707	DDG2P_v2.2 panel
ASXL1	ENSG00000171456	DDG2P_v2.2 panel
ASXL2	ENSG00000143970	DDG2P_v2.2 panel
ASXL3	ENSG00000141431	DDG2P_v2.2 panel
ATAD3A	ENSG00000197785	DDG2P_v2.2 panel
ATIC	ENSG00000138363	DDG2P_v2.2 panel
ATM	ENSG00000149311	DDG2P_v2.2 panel
ATN1	ENSG00000111676	DDG2P_v2.2 panel
ATOH7	ENSG00000179774	DDG2P_v2.2 panel
ATP13A2	ENSG00000159363	DDG2P_v2.2 panel
ATP1A1	ENSG00000163399	DDG2P_v2.2 panel
ATP1A3	ENSG00000105409	DDG2P_v2.2 panel
ATP5D	ENSG00000099624	DDG2P_v2.2 panel
ATP6AP2	ENSG00000182220	DDG2P_v2.2 panel
ATP6V0A2	ENSG00000185344	Lord et. al.
ATP6V1A	ENSG00000114573	DDG2P_v2.2 panel
ATP6V1B1	ENSG00000116039	DDG2P_v2.2 panel
ATP6V1B2	ENSG00000147416	DDG2P_v2.2 panel
ATP6V1E1	ENSG00000131100	DDG2P_v2.2 panel
ATP7A	ENSG00000165240	DDG2P_v2.2 panel
ATP8A2	ENSG00000132932	DDG2P_v2.2 panel
ATP8B1	ENSG00000081923	DDG2P_v2.2 panel
ATR	ENSG00000175054	DDG2P_v2.2 panel
ATRX	ENSG00000085224	DDG2P_v2.2 panel
AUH	ENSG00000148090	DDG2P_v2.2 panel
AUTS2	ENSG00000158321	DDG2P_v2.2 panel
AXIN1	ENSG00000103126	DDG2P_v2.2 panel
B3GALNT2	ENSG00000162885	DDG2P_v2.2 panel
B3GALT6	ENSG00000176022	DDG2P_v2.2 panel
B3GAT3	ENSG00000149541	DDG2P_v2.2 panel and Lord et. al.
B3GLCT	ENSG00000187676	Lord et. al.
B4GALT7	ENSG00000027847	DDG2P_v2.2 panel

B9D1	ENSG00000108641	DDG2P_v2.2 panel
BANF1	ENSG00000175334	DDG2P_v2.2 panel
BBS1	ENSG00000174483	DDG2P_v2.2 panel
BBS10	ENSG00000179941	DDG2P_v2.2 panel
BBS12	ENSG00000181004	DDG2P_v2.2 panel
BBS2	ENSG00000125124	DDG2P_v2.2 panel
BBS4	ENSG00000140463	DDG2P_v2.2 panel
BBS5	ENSG00000163093	DDG2P_v2.2 panel
BBS7	ENSG00000138686	DDG2P_v2.2 panel
BBS9	ENSG00000122507	DDG2P_v2.2 panel
BCAP31	ENSG00000185825	DDG2P_v2.2 panel
BCKDHA	ENSG00000248098	DDG2P_v2.2 panel
BCKDHB	ENSG00000083123	DDG2P_v2.2 panel
BCL11A	ENSG00000119866	DDG2P_v2.2 panel
BCOR	ENSG00000183337	DDG2P_v2.2 panel
BCS1L	ENSG00000074582	DDG2P_v2.2 panel
BFSP2	ENSG00000170819	DDG2P_v2.2 panel
BGN	ENSG00000182492	DDG2P_v2.2 panel
BHLHA9	ENSG00000205899	DDG2P_v2.2 panel
BICD2	ENSG00000185963	DDG2P_v2.2 panel
BIN1	ENSG00000136717	DDG2P_v2.2 panel
BLM	ENSG00000197299	DDG2P_v2.2 panel
BLOC1S6	ENSG00000104164	DDG2P_v2.2 panel
BMP2	ENSG00000125845	DDG2P_v2.2 panel
BMP4	ENSG00000125378	DDG2P_v2.2 panel
BMPER	ENSG00000164619	DDG2P_v2.2 panel
BMPR1B	ENSG00000138696	DDG2P_v2.2 panel
BNC2	ENSG00000173068	DDG2P_v2.2 panel
BOLA3	ENSG00000163170	DDG2P_v2.2 panel
BPIFB6	ENSG00000167104	DDG2P_v2.2 panel
BPTF	ENSG00000171634	DDG2P_v2.2 panel
BRAF	ENSG00000157764	DDG2P_v2.2 panel
BRAT1	ENSG00000106009	DDG2P_v2.2 panel
BRCA1	ENSG00000012048	DDG2P_v2.2 panel
BRCA2	ENSG00000139618	DDG2P_v2.2 panel
BRD4	ENSG00000141867	DDG2P_v2.2 panel
BRIP1	ENSG00000136492	DDG2P_v2.2 panel
BRPF1	ENSG00000156983	DDG2P_v2.2 panel
BRSK2	ENSG00000174672	DDG2P_v2.2 panel
BRWD3	ENSG00000165288	DDG2P_v2.2 panel
BSND	ENSG00000162399	DDG2P_v2.2 panel
BTD	ENSG00000169814	DDG2P_v2.2 panel
BUB1B	ENSG00000156970	DDG2P_v2.2 panel
C11orf70	ENSG00000137691	DDG2P_v2.2 panel
C12orf57	ENSG00000111678	DDG2P_v2.2 panel
C12orf65	ENSG00000130921	DDG2P_v2.2 panel
C1QBP	ENSG00000108561	DDG2P_v2.2 panel
C21orf2	ENSG00000160226	DDG2P_v2.2 panel
C21orf59	ENSG00000159079	DDG2P_v2.2 panel
C2CD3	ENSG00000168014	DDG2P_v2.2 panel
C2orf71	ENSG00000179270	DDG2P_v2.2 panel
C4orf26	ENSG00000174792	DDG2P_v2.2 panel
C5orf42	ENSG00000197603	DDG2P_v2.2 panel
C8orf37	ENSG00000156172	DDG2P_v2.2 panel
CA2	ENSG00000104267	DDG2P_v2.2 panel
CA5A	ENSG00000174990	DDG2P_v2.2 panel
CA8	ENSG00000178538	DDG2P_v2.2 panel
CACNA1A	ENSG00000141837	DDG2P_v2.2 panel
CACNA1B	ENSG00000148408	DDG2P_v2.2 panel
CACNA1C	ENSG00000151067	DDG2P_v2.2 panel

CACNA1D	ENSG00000157388	DDG2P_v2.2 panel
CACNA1E	ENSG00000198216	DDG2P_v2.2 panel
CACNA1G	ENSG00000006283	DDG2P_v2.2 panel
CACNA1H	ENSG00000196557	DDG2P_v2.2 panel
CACNB4	ENSG00000182389	DDG2P_v2.2 panel
CAD	ENSG00000084774	DDG2P_v2.2 panel
CAMK2A	ENSG00000070808	DDG2P_v2.2 panel
CAMK2B	ENSG00000058404	DDG2P_v2.2 panel
CAMTA1	ENSG00000171735	DDG2P_v2.2 panel
CAPN10	ENSG00000142330	DDG2P_v2.2 panel
CAPRIN1	ENSG00000135387	DDG2P_v2.2 panel
CARS	ENSG00000110619	DDG2P_v2.2 panel
CARS2	ENSG00000134905	DDG2P_v2.2 panel
CASK	ENSG00000147044	DDG2P_v2.2 panel
CASP2	ENSG00000106144	DDG2P_v2.2 panel
CAV1	ENSG00000105974	DDG2P_v2.2 panel
CAVIN1	ENSG00000177469	Lord et. al.
CBL	ENSG00000110395	DDG2P_v2.2 panel
CBS	ENSG00000160200	DDG2P_v2.2 panel
CC2D1A	ENSG00000132024	DDG2P_v2.2 panel
CC2D2A	ENSG00000048342	DDG2P_v2.2 panel
CCBE1	ENSG00000183287	DDG2P_v2.2 panel
CCDC103	ENSG00000167131	DDG2P_v2.2 panel
CCDC114	ENSG00000105479	DDG2P_v2.2 panel
CCDC115	ENSG00000136710	DDG2P_v2.2 panel
CCDC151	ENSG00000198003	DDG2P_v2.2 panel
CCDC22	ENSG00000101997	DDG2P_v2.2 panel
CCDC39	ENSG00000145075	DDG2P_v2.2 panel
CCDC40	ENSG00000141519	DDG2P_v2.2 panel
CCDC47	ENSG00000108588	DDG2P_v2.2 panel
CCDC65	ENSG00000139537	DDG2P_v2.2 panel
CCDC78	ENSG00000162004	DDG2P_v2.2 panel
CCDC8	ENSG00000169515	DDG2P_v2.2 panel
CCDC88A	ENSG00000115355	DDG2P_v2.2 panel
CCDC88C	ENSG00000015133	DDG2P_v2.2 panel
CCNA2	ENSG00000145386	DDG2P_v2.2 panel
CCND2	ENSG00000118971	DDG2P_v2.2 panel
CCNK	ENSG00000090061	DDG2P_v2.2 panel
CCNO	ENSG00000152669	DDG2P_v2.2 panel
CD151	ENSG00000177697	DDG2P_v2.2 panel
CD96	ENSG00000153283	DDG2P_v2.2 panel
CDAN1	ENSG00000140326	Lord et. al.
CDC45	ENSG00000093009	DDG2P_v2.2 panel
CDC6	ENSG00000094804	DDG2P_v2.2 panel
CDH1	ENSG00000039068	DDG2P_v2.2 panel
CDH15	ENSG00000129910	DDG2P_v2.2 panel
CDH2	ENSG00000170558	DDG2P_v2.2 panel
CDH23	ENSG00000107736	DDG2P_v2.2 panel
CDH3	ENSG00000062038	DDG2P_v2.2 panel
CDK10	ENSG00000185324	DDG2P_v2.2 panel
CDK13	ENSG00000065883	DDG2P_v2.2 panel
CDK16	ENSG00000102225	DDG2P_v2.2 panel
CDK5RAP2	ENSG00000136861	DDG2P_v2.2 panel
CDK8	ENSG00000132964	DDG2P_v2.2 panel
CDKL5	ENSG00000008086	DDG2P_v2.2 panel
CDKN1C	ENSG00000129757	DDG2P_v2.2 panel
CDON	ENSG00000064309	DDG2P_v2.2 panel
CDT1	ENSG00000167513	DDG2P_v2.2 panel
CENPF	ENSG00000117724	DDG2P_v2.2 panel
CENPJ	ENSG00000151849	DDG2P_v2.2 panel
CEP104	ENSG00000116198	DDG2P_v2.2 panel

CEP135	ENSG00000174799	DDG2P_v2.2 panel
CEP152	ENSG00000103995	DDG2P_v2.2 panel
CEP164	ENSG00000110274	Lord et. al.
CEP290	ENSG00000198707	DDG2P_v2.2 panel
CEP41	ENSG00000106477	DDG2P_v2.2 panel
CEP57	ENSG00000166037	DDG2P_v2.2 panel
CEP63	ENSG00000182923	DDG2P_v2.2 panel
CEP83	ENSG00000173588	DDG2P_v2.2 panel
CFC1	ENSG00000136698	DDG2P_v2.2 panel
CFL2	ENSG00000165410	DDG2P_v2.2 panel
CFTR	ENSG00000001626	Lord et. al.
CHAMP1	ENSG00000198824	DDG2P_v2.2 panel
CHAT	ENSG00000070748	Lord et. al.
CHD2	ENSG00000173575	DDG2P_v2.2 panel
CHD3	ENSG00000170004	DDG2P_v2.2 panel
CHD4	ENSG00000111642	DDG2P_v2.2 panel
CHD7	ENSG00000171316	DDG2P_v2.2 panel
CHD8	ENSG00000100888	DDG2P_v2.2 panel
CHKB	ENSG00000100288	Lord et. al.
CHM	ENSG00000188419	DDG2P_v2.2 panel
CHMP1A	ENSG00000131165	DDG2P_v2.2 panel
CHRDL1	ENSG00000101938	DDG2P_v2.2 panel
CHRNA1	ENSG00000138435	DDG2P_v2.2 panel
CHRNA2	ENSG00000120903	DDG2P_v2.2 panel
CHRNA4	ENSG00000101204	DDG2P_v2.2 panel
CHRNB2	ENSG00000160716	DDG2P_v2.2 panel
CHRND	ENSG00000135902	Lord et. al.
CHRNG	ENSG00000196811	DDG2P_v2.2 panel
CHST14	ENSG00000169105	DDG2P_v2.2 panel
CHST3	ENSG00000122863	DDG2P_v2.2 panel
CHSY1	ENSG00000131873	DDG2P_v2.2 panel
CHUK	ENSG00000213341	DDG2P_v2.2 panel
CIB2	ENSG00000136425	DDG2P_v2.2 panel
CIC	ENSG00000079432	DDG2P_v2.2 panel
CISD2	ENSG00000145354	DDG2P_v2.2 panel
CIT	ENSG00000122966	DDG2P_v2.2 panel
CKAP2L	ENSG00000169607	DDG2P_v2.2 panel
CLCN4	ENSG00000073464	DDG2P_v2.2 panel
CLCN7	ENSG00000103249	DDG2P_v2.2 panel
CLCNKA	ENSG00000186510	DDG2P_v2.2 panel
CLCNKB	ENSG00000184908	DDG2P_v2.2 panel
CLDN19	ENSG00000164007	DDG2P_v2.2 panel
CLIC2	ENSG00000155962	DDG2P_v2.2 panel
CLMP	ENSG00000166250	DDG2P_v2.2 panel
CLN3	ENSG00000188603	DDG2P_v2.2 panel
CLN5	ENSG00000102805	DDG2P_v2.2 panel
CLN6	ENSG00000128973	DDG2P_v2.2 panel
CLN8	ENSG00000182372	DDG2P_v2.2 panel
CLP1	ENSG00000172409	DDG2P_v2.2 panel
CLPB	ENSG00000162129	DDG2P_v2.2 panel
CLPP	ENSG00000125656	DDG2P_v2.2 panel
CLTC	ENSG00000141367	DDG2P_v2.2 panel
CNKSR1	ENSG00000142675	DDG2P_v2.2 panel
CNKSR2	ENSG00000149970	DDG2P_v2.2 panel
CNOT1	ENSG00000125107	DDG2P_v2.2 panel
CNOT3	ENSG00000088038	DDG2P_v2.2 panel
CNPY3	ENSG00000137161	DDG2P_v2.2 panel
CNTNAP1	ENSG00000108797	DDG2P_v2.2 panel
CNTNAP2	ENSG00000174469	DDG2P_v2.2 panel
COA5	ENSG00000183513	DDG2P_v2.2 panel
COASY	ENSG00000068120	DDG2P_v2.2 panel

COG1	ENSG00000166685	DDG2P_v2.2 panel
COG4	ENSG00000103051	DDG2P_v2.2 panel
COG5	ENSG00000164597	DDG2P_v2.2 panel
COG7	ENSG00000168434	DDG2P_v2.2 panel
COG8	ENSG00000213380	DDG2P_v2.2 panel
COL10A1	ENSG00000123500	DDG2P_v2.2 panel
COL11A1	ENSG00000060718	DDG2P_v2.2 panel
COL11A2	ENSG00000204248	DDG2P_v2.2 panel
COL13A1	ENSG00000197467	DDG2P_v2.2 panel
COL18A1	ENSG00000182871	DDG2P_v2.2 panel
COL1A1	ENSG00000108821	DDG2P_v2.2 panel
COL1A2	ENSG00000164692	Lord et. al.
COL25A1	ENSG00000188517	DDG2P_v2.2 panel
COL2A1	ENSG00000139219	DDG2P_v2.2 panel
COL4A1	ENSG00000187498	DDG2P_v2.2 panel
COL4A2	ENSG00000134871	DDG2P_v2.2 panel
COL4A3	ENSG00000169031	DDG2P_v2.2 panel
COL4A3BP	ENSG00000113163	DDG2P_v2.2 panel
COL4A4	ENSG00000081052	DDG2P_v2.2 panel
COL5A1	ENSG00000130635	Lord et. al.
COL5A2	ENSG00000204262	Lord et. al.
COL6A1	ENSG00000142156	DDG2P_v2.2 panel
COL6A2	ENSG00000142173	Lord et. al.
COL6A3	ENSG00000163359	DDG2P_v2.2 panel
COL9A1	ENSG00000112280	DDG2P_v2.2 panel
COL9A2	ENSG00000049089	DDG2P_v2.2 panel
COL9A3	ENSG00000092758	DDG2P_v2.2 panel
COLEC10	ENSG00000184374	DDG2P_v2.2 panel
COLEC11	ENSG00000118004	DDG2P_v2.2 panel
COMP	ENSG00000105664	DDG2P_v2.2 panel
COQ2	ENSG00000173085	DDG2P_v2.2 panel
COQ4	ENSG00000167113	DDG2P_v2.2 panel
COQ5	ENSG00000110871	DDG2P_v2.2 panel
COQ8A	ENSG00000163050	DDG2P_v2.2 panel
COQ9	ENSG00000088682	DDG2P_v2.2 panel
COX10	ENSG00000006695	DDG2P_v2.2 panel
COX14	ENSG00000178449	DDG2P_v2.2 panel
COX15	ENSG00000014919	DDG2P_v2.2 panel
COX6B1	ENSG00000126267	DDG2P_v2.2 panel
COX7B	ENSG00000131174	DDG2P_v2.2 panel
CPAMD8	ENSG00000160111	DDG2P_v2.2 panel
CPS1	ENSG00000021826	DDG2P_v2.2 panel
CPT2	ENSG00000157184	Lord et. al.
CRADD	ENSG00000169372	DDG2P_v2.2 panel
CRB1	ENSG00000134376	DDG2P_v2.2 panel
CRB2	ENSG00000148204	DDG2P_v2.2 panel
CRBN	ENSG00000113851	DDG2P_v2.2 panel
CREBBP	ENSG00000005339	DDG2P_v2.2 panel
CRELD1	ENSG00000163703	DDG2P_v2.2 panel
CRIM1	ENSG00000150938	DDG2P_v2.2 panel
CRIPT	ENSG00000119878	DDG2P_v2.2 panel
CRKL	ENSG00000099942	DDG2P_v2.2 panel
CRLF1	ENSG00000006016	Lord et. al.
CRTAP	ENSG00000170275	Lord et. al.
CRX	ENSG00000105392	DDG2P_v2.2 panel
CRYAA	ENSG00000160202	DDG2P_v2.2 panel
CRYAB	ENSG00000109846	DDG2P_v2.2 panel
CRYBA1	ENSG00000108255	DDG2P_v2.2 panel
CRYBA4	ENSG00000196431	DDG2P_v2.2 panel
CRYBB1	ENSG00000100122	DDG2P_v2.2 panel
CRYBB2	ENSG00000244752	DDG2P_v2.2 panel

CRYBB3	ENSG00000100053	DDG2P_v2.2 panel
CRYGC	ENSG00000163254	DDG2P_v2.2 panel
CRYGD	ENSG00000118231	DDG2P_v2.2 panel
CSF1R	ENSG00000182578	DDG2P_v2.2 panel
CSNK1G1	ENSG00000169118	DDG2P_v2.2 panel
CSNK2A1	ENSG00000101266	DDG2P_v2.2 panel
CSPP1	ENSG00000104218	DDG2P_v2.2 panel
CSTA	ENSG00000121552	DDG2P_v2.2 panel
CSTB	ENSG00000160213	DDG2P_v2.2 panel
CTC1	ENSG00000178971	DDG2P_v2.2 panel
CTCF	ENSG00000102974	DDG2P_v2.2 panel
CTDP1	ENSG00000060069	DDG2P_v2.2 panel
CTNNA2	ENSG00000066032	DDG2P_v2.2 panel
CTNNB1	ENSG00000168036	DDG2P_v2.2 panel
CTNND1	ENSG00000198561	DDG2P_v2.2 panel
CTNS	ENSG00000040531	DDG2P_v2.2 panel
CTSA	ENSG00000064601	DDG2P_v2.2 panel
CTSD	ENSG00000117984	DDG2P_v2.2 panel
CTSK	ENSG00000143387	DDG2P_v2.2 panel
CUL3	ENSG00000036257	DDG2P_v2.2 panel
CUL4B	ENSG00000158290	DDG2P_v2.2 panel
CUL7	ENSG00000044090	DDG2P_v2.2 panel
CUX2	ENSG00000111249	DDG2P_v2.2 panel
CWC27	ENSG00000153015	DDG2P_v2.2 panel
CYB5R3	ENSG00000100243	DDG2P_v2.2 panel
CYC1	ENSG00000179091	DDG2P_v2.2 panel
CYP11A1	ENSG00000140459	Lord et. al.
CYP11B1	ENSG00000160882	Lord et. al.
CYP17A1	ENSG00000148795	Lord et. al.
CYP1B1	ENSG00000138061	DDG2P_v2.2 panel
CYP21A2	ENSG00000231852	Lord et. al.
CYP24A1	ENSG00000019186	DDG2P_v2.2 panel
CYP27A1	ENSG00000135929	DDG2P_v2.2 panel
CYP2U1	ENSG00000155016	DDG2P_v2.2 panel
D2HGDH	ENSG00000180902	DDG2P_v2.2 panel
DACT1	ENSG00000165617	DDG2P_v2.2 panel
DAG1	ENSG00000173402	DDG2P_v2.2 panel
DARS	ENSG00000115866	DDG2P_v2.2 panel
DARS2	ENSG00000117593	DDG2P_v2.2 panel
DBT	ENSG00000137992	DDG2P_v2.2 panel
DCAF17	ENSG00000115827	DDG2P_v2.2 panel
DCC	ENSG00000187323	DDG2P_v2.2 panel
DCDC2	ENSG00000146038	DDG2P_v2.2 panel
DCHS1	ENSG00000166341	DDG2P_v2.2 panel
DCX	ENSG00000077279	DDG2P_v2.2 panel
DDB2	ENSG00000134574	DDG2P_v2.2 panel
DDC	ENSG00000132437	DDG2P_v2.2 panel
DDHD1	ENSG00000100523	DDG2P_v2.2 panel
DDHD2	ENSG00000085788	DDG2P_v2.2 panel
DDOST	ENSG00000244038	DDG2P_v2.2 panel
DDR2	ENSG00000162733	DDG2P_v2.2 panel
DDX11	ENSG00000013573	DDG2P_v2.2 panel
DDX3X	ENSG00000215301	DDG2P_v2.2 panel
DDX54	ENSG00000123064	DDG2P_v2.2 panel
DDX58	ENSG00000107201	DDG2P_v2.2 panel
DDX59	ENSG00000118197	DDG2P_v2.2 panel
DDX6	ENSG00000110367	DDG2P_v2.2 panel
DEAF1	ENSG00000177030	DDG2P_v2.2 panel
DEGS1	ENSG00000143753	DDG2P_v2.2 panel
DENND5A	ENSG00000184014	DDG2P_v2.2 panel
DEPDC5	ENSG00000100150	DDG2P_v2.2 panel

DGAT1	ENSG00000185000	DDG2P_v2.2 panel
DHCR24	ENSG00000116133	DDG2P_v2.2 panel
DHCR7	ENSG00000172893	DDG2P_v2.2 panel
DHDDS	ENSG00000117682	DDG2P_v2.2 panel
DHFR	ENSG00000228716	DDG2P_v2.2 panel
DHODH	ENSG00000102967	DDG2P_v2.2 panel
DHPS	ENSG00000095059	DDG2P_v2.2 panel
DHRS3	ENSG00000162496	DDG2P_v2.2 panel
DHTKD1	ENSG00000181192	DDG2P_v2.2 panel
DHX16	ENSG00000204560	DDG2P_v2.2 panel
DHX30	ENSG00000132153	DDG2P_v2.2 panel
DHX34	ENSG00000134815	DDG2P_v2.2 panel
DHX37	ENSG00000150990	DDG2P_v2.2 panel
DIP2B	ENSG00000066084	DDG2P_v2.2 panel
DIS3L2	ENSG00000144535	DDG2P_v2.2 panel
DKC1	ENSG00000130826	DDG2P_v2.2 panel
DLAT	ENSG00000150768	DDG2P_v2.2 panel
DLD	ENSG00000091140	DDG2P_v2.2 panel
DLG3	ENSG00000082458	DDG2P_v2.2 panel
DLG4	ENSG00000132535	DDG2P_v2.2 panel
DLL1	ENSG00000198719	DDG2P_v2.2 panel
DLL3	ENSG00000090932	DDG2P_v2.2 panel
DLL4	ENSG00000128917	DDG2P_v2.2 panel
DLX5	ENSG00000105880	DDG2P_v2.2 panel
DMD	ENSG00000198947	DDG2P_v2.2 panel
DMP1	ENSG00000152592	DDG2P_v2.2 panel
DMPK	ENSG00000104936	DDG2P_v2.2 panel
DNA2	ENSG00000138346	DDG2P_v2.2 panel
DNAAF1	ENSG00000154099	Lord et. al.
DNAAF3	ENSG00000167646	DDG2P_v2.2 panel
DNAAF4	ENSG00000256061	DDG2P_v2.2 panel
DNAAF5	ENSG00000164818	DDG2P_v2.2 panel
DNAH11	ENSG00000105877	Lord et. al.
DNAH5	ENSG00000039139	DDG2P_v2.2 panel and Lord et. al.
DNAH9	ENSG00000007174	DDG2P_v2.2 panel
DNAI1	ENSG00000122735	Lord et. al.
DNAJB13	ENSG00000187726	DDG2P_v2.2 panel
DNAJC12	ENSG00000108176	DDG2P_v2.2 panel
DNM1	ENSG00000106976	DDG2P_v2.2 panel
DNMT3A	ENSG00000119772	DDG2P_v2.2 panel
DNMT3B	ENSG00000088305	DDG2P_v2.2 panel
DOCK6	ENSG00000130158	DDG2P_v2.2 panel
DOCK7	ENSG00000116641	DDG2P_v2.2 panel
DOCK8	ENSG00000107099	DDG2P_v2.2 panel
DOLK	ENSG00000175283	DDG2P_v2.2 panel
DPAGT1	ENSG00000172269	DDG2P_v2.2 panel
DPF2	ENSG00000133884	DDG2P_v2.2 panel
DPM1	ENSG00000000419	DDG2P_v2.2 panel
DPM3	ENSG00000179085	DDG2P_v2.2 panel
DRC1	ENSG00000157856	DDG2P_v2.2 panel
DSE	ENSG00000111817	DDG2P_v2.2 panel
DSG1	ENSG00000134760	DDG2P_v2.2 panel
DSP	ENSG00000096696	Lord et. al.
DSPP	ENSG00000152591	DDG2P_v2.2 panel
DSTYK	ENSG00000133059	DDG2P_v2.2 panel
DVL1	ENSG00000107404	DDG2P_v2.2 panel
DVL3	ENSG00000161202	DDG2P_v2.2 panel
DYM	ENSG00000141627	DDG2P_v2.2 panel
DYNC1H1	ENSG00000197102	DDG2P_v2.2 panel
DYNC1I2	ENSG00000077380	DDG2P_v2.2 panel
DYNC2H1	ENSG00000187240	DDG2P_v2.2 panel

DYRK1A	ENSG00000157540	DDG2P_v2.2 panel
EBF3	ENSG00000108001	DDG2P_v2.2 panel
EBP	ENSG00000147155	DDG2P_v2.2 panel
ECEL1	ENSG00000171551	DDG2P_v2.2 panel
EDA	ENSG00000158813	DDG2P_v2.2 panel
EDAR	ENSG00000135960	DDG2P_v2.2 panel
EDN1	ENSG00000078401	DDG2P_v2.2 panel
EDNRA	ENSG00000151617	DDG2P_v2.2 panel
EDNRB	ENSG00000136160	DDG2P_v2.2 panel
EED	ENSG00000074266	DDG2P_v2.2 panel
EEF1A2	ENSG00000101210	DDG2P_v2.2 panel
EEF1B2	ENSG00000114942	DDG2P_v2.2 panel
EFNB1	ENSG00000090776	DDG2P_v2.2 panel
EFTUD2	ENSG00000108883	DDG2P_v2.2 panel
EGR2	ENSG00000122877	DDG2P_v2.2 panel
EHMT1	ENSG00000181090	DDG2P_v2.2 panel
EIF2AK3	ENSG00000172071	DDG2P_v2.2 panel
EIF2S3	ENSG00000130741	DDG2P_v2.2 panel
EIF3F	ENSG00000175390	DDG2P_v2.2 panel
EIF4A3	ENSG00000141543	DDG2P_v2.2 panel
ELAC2	ENSG00000006744	DDG2P_v2.2 panel
ELMO2	ENSG00000062598	DDG2P_v2.2 panel
ELN	ENSG00000049540	DDG2P_v2.2 panel
ELOVL4	ENSG00000118402	DDG2P_v2.2 panel
ELP2	ENSG00000134759	DDG2P_v2.2 panel
EMC1	ENSG00000127463	DDG2P_v2.2 panel
EMD	ENSG00000102119	Lord et. al.
EMG1	ENSG00000126749	DDG2P_v2.2 panel and Lord et. al.
EMX2	ENSG00000170370	DDG2P_v2.2 panel
ENPP1	ENSG00000197594	DDG2P_v2.2 panel
ENTPD1	ENSG00000138185	DDG2P_v2.2 panel
EOGT	ENSG00000163378	DDG2P_v2.2 panel
EOMES	ENSG00000163508	DDG2P_v2.2 panel
EP300	ENSG00000100393	DDG2P_v2.2 panel
EPB41L1	ENSG00000088367	DDG2P_v2.2 panel
EPG5	ENSG00000152223	DDG2P_v2.2 panel
EPHB4	ENSG00000196411	DDG2P_v2.2 panel and Lord et. al.
EPRS	ENSG00000136628	DDG2P_v2.2 panel
ERBB3	ENSG00000065361	DDG2P_v2.2 panel
ERCC1	ENSG00000012061	DDG2P_v2.2 panel
ERCC2	ENSG00000104884	DDG2P_v2.2 panel
ERCC3	ENSG00000163161	DDG2P_v2.2 panel
ERCC4	ENSG00000175595	DDG2P_v2.2 panel
ERCC5	ENSG00000134899	DDG2P_v2.2 panel
ERCC6	ENSG00000225830	DDG2P_v2.2 panel
ERCC6L2	ENSG00000182150	DDG2P_v2.2 panel
ERCC8	ENSG00000049167	DDG2P_v2.2 panel
ERF	ENSG00000105722	DDG2P_v2.2 panel
ERLIN2	ENSG00000147475	DDG2P_v2.2 panel
ERMARD	ENSG00000130023	DDG2P_v2.2 panel
ESCO2	ENSG00000171320	DDG2P_v2.2 panel
ETFA	ENSG00000140374	DDG2P_v2.2 panel
ETFB	ENSG00000105379	DDG2P_v2.2 panel
ETFDH	ENSG00000171503	DDG2P_v2.2 panel
ETHE1	ENSG00000105755	DDG2P_v2.2 panel
EVC	ENSG00000072840	DDG2P_v2.2 panel
EVC2	ENSG00000173040	DDG2P_v2.2 panel
EXOSC3	ENSG00000107371	DDG2P_v2.2 panel
EXOSC9	ENSG00000123737	DDG2P_v2.2 panel
EXPH5	ENSG00000110723	DDG2P_v2.2 panel
EXT1	ENSG00000182197	DDG2P_v2.2 panel

EXT2	ENSG00000151348	DDG2P_v2.2 panel
EXTL3	ENSG00000012232	DDG2P_v2.2 panel
EYA1	ENSG00000104313	DDG2P_v2.2 panel
EZH2	ENSG00000106462	DDG2P_v2.2 panel
FAH	ENSG00000103876	DDG2P_v2.2 panel
FAM111A	ENSG00000166801	DDG2P_v2.2 panel
FAM126A	ENSG00000122591	DDG2P_v2.2 panel
FAM149B1	ENSG00000138286	DDG2P_v2.2 panel
FAM161A	ENSG00000170264	DDG2P_v2.2 panel
FAM20A	ENSG00000108950	DDG2P_v2.2 panel
FAM20C	ENSG00000177706	DDG2P_v2.2 panel
FAM58A	ENSG00000262919	DDG2P_v2.2 panel
FANCA	ENSG00000187741	DDG2P_v2.2 panel
FANCB	ENSG00000181544	DDG2P_v2.2 panel
FANCC	ENSG00000158169	DDG2P_v2.2 panel
FANCD2	ENSG00000144554	DDG2P_v2.2 panel
FANCE	ENSG00000112039	DDG2P_v2.2 panel
FANCF	ENSG00000183161	DDG2P_v2.2 panel
FANCG	ENSG00000221829	DDG2P_v2.2 panel
FANCI	ENSG00000140525	DDG2P_v2.2 panel
FANCL	ENSG00000115392	DDG2P_v2.2 panel
FANCM	ENSG00000187790	DDG2P_v2.2 panel
FAR1	ENSG00000197601	DDG2P_v2.2 panel
FARS2	ENSG00000145982	DDG2P_v2.2 panel
FASN	ENSG00000169710	DDG2P_v2.2 panel
FAT4	ENSG00000196159	DDG2P_v2.2 panel
FBLN1	ENSG00000077942	DDG2P_v2.2 panel
FBLN5	ENSG00000140092	Lord et. al.
FBN1	ENSG00000166147	DDG2P_v2.2 panel
FBN2	ENSG00000138829	DDG2P_v2.2 panel
FBP1	ENSG00000165140	DDG2P_v2.2 panel
FBXL4	ENSG00000112234	DDG2P_v2.2 panel
FBXO11	ENSG00000138081	DDG2P_v2.2 panel
FBXO25	ENSG00000147364	DDG2P_v2.2 panel
FBXW11	ENSG00000072803	DDG2P_v2.2 panel
FBXW4	ENSG00000107829	DDG2P_v2.2 panel
FDFT1	ENSG00000079459	DDG2P_v2.2 panel
FEZF1	ENSG00000128610	DDG2P_v2.2 panel
FGD1	ENSG00000102302	DDG2P_v2.2 panel
FGF10	ENSG00000070193	DDG2P_v2.2 panel
FGF12	ENSG00000114279	DDG2P_v2.2 panel
FGF3	ENSG00000186895	DDG2P_v2.2 panel
FGF8	ENSG00000107831	Lord et. al.
FGF9	ENSG00000102678	DDG2P_v2.2 panel
FGFR1	ENSG00000077782	DDG2P_v2.2 panel
FGFR2	ENSG00000066468	DDG2P_v2.2 panel
FGFR3	ENSG00000068078	DDG2P_v2.2 panel
FH	ENSG00000091483	DDG2P_v2.2 panel
FHL1	ENSG00000022267	DDG2P_v2.2 panel
FIG4	ENSG00000112367	DDG2P_v2.2 panel
FKBP14	ENSG00000106080	DDG2P_v2.2 panel
FKRP	ENSG00000181027	DDG2P_v2.2 panel
FKTN	ENSG00000106692	DDG2P_v2.2 panel
FLAD1	ENSG00000160688	DDG2P_v2.2 panel
FLG	ENSG00000143631	DDG2P_v2.2 panel
FLNA	ENSG00000196924	DDG2P_v2.2 panel
FLNB	ENSG00000136068	DDG2P_v2.2 panel
FLT4	ENSG00000037280	DDG2P_v2.2 panel
FLVCR1	ENSG00000162769	DDG2P_v2.2 panel
FLVCR2	ENSG00000119686	DDG2P_v2.2 panel
FMN2	ENSG00000155816	DDG2P_v2.2 panel

FMR1	ENSG00000102081	DDG2P_v2.2 panel
FN1	ENSG00000115414	DDG2P_v2.2 panel
FOLR1	ENSG00000110195	DDG2P_v2.2 panel
FOXC1	ENSG00000054598	DDG2P_v2.2 panel
FOXC2	ENSG00000176692	DDG2P_v2.2 panel
FOXE1	ENSG00000178919	DDG2P_v2.2 panel
FOXE3	ENSG00000186790	DDG2P_v2.2 panel
FOXF1	ENSG00000103241	DDG2P_v2.2 panel
FOXG1	ENSG00000176165	DDG2P_v2.2 panel
FOXL2	ENSG00000183770	DDG2P_v2.2 panel
FOXN1	ENSG00000109101	DDG2P_v2.2 panel
FOXP1	ENSG00000114861	DDG2P_v2.2 panel
FOXP2	ENSG00000128573	DDG2P_v2.2 panel
FOXP3	ENSG00000049768	DDG2P_v2.2 panel
FOXRED1	ENSG00000110074	DDG2P_v2.2 panel
FRAS1	ENSG00000138759	DDG2P_v2.2 panel
FREM1	ENSG00000164946	DDG2P_v2.2 panel
FREM2	ENSG00000150893	DDG2P_v2.2 panel
FRMD7	ENSG00000165694	DDG2P_v2.2 panel
FRMPD4	ENSG00000169933	DDG2P_v2.2 panel
FRRS1L	ENSG00000260230	DDG2P_v2.2 panel
FRY	ENSG00000073910	DDG2P_v2.2 panel
FTCD	ENSG00000160282	DDG2P_v2.2 panel
FTL	ENSG00000087086	DDG2P_v2.2 panel
FTO	ENSG00000140718	DDG2P_v2.2 panel
FTSJ1	ENSG00000068438	DDG2P_v2.2 panel
FUCA1	ENSG00000179163	DDG2P_v2.2 panel
FUK	ENSG00000157353	DDG2P_v2.2 panel
FUT8	ENSG00000033170	DDG2P_v2.2 panel
FYCO1	ENSG00000163820	DDG2P_v2.2 panel
FZD5	ENSG00000163251	DDG2P_v2.2 panel
FZD6	ENSG00000164930	DDG2P_v2.2 panel
G6PC3	ENSG00000141349	Lord et. al.
GAA	ENSG00000171298	DDG2P_v2.2 panel
GABBR2	ENSG00000136928	DDG2P_v2.2 panel
GABRA1	ENSG00000022355	DDG2P_v2.2 panel
GABRB2	ENSG00000145864	DDG2P_v2.2 panel
GABRB3	ENSG00000166206	DDG2P_v2.2 panel
GABRG2	ENSG00000113327	DDG2P_v2.2 panel
GAD1	ENSG00000128683	DDG2P_v2.2 panel
GALC	ENSG00000054983	DDG2P_v2.2 panel
GALE	ENSG00000117308	DDG2P_v2.2 panel
GALK1	ENSG00000108479	DDG2P_v2.2 panel
GALNS	ENSG00000141012	DDG2P_v2.2 panel
GALT	ENSG00000213930	DDG2P_v2.2 panel
GAMT	ENSG00000130005	DDG2P_v2.2 panel
GAS2L2	ENSG00000270765	DDG2P_v2.2 panel
GAS8	ENSG00000141013	DDG2P_v2.2 panel
GATA2	ENSG00000179348	DDG2P_v2.2 panel
GATA4	ENSG00000136574	DDG2P_v2.2 panel
GATA6	ENSG00000141448	DDG2P_v2.2 panel
GATAD2B	ENSG00000143614	DDG2P_v2.2 panel
GATM	ENSG00000171766	DDG2P_v2.2 panel
GBA	ENSG00000177628	DDG2P_v2.2 panel
GBA2	ENSG00000070610	DDG2P_v2.2 panel
GBE1	ENSG00000114480	Lord et. al.
GCDH	ENSG00000105607	DDG2P_v2.2 panel
GCH1	ENSG00000131979	DDG2P_v2.2 panel
GCSH	ENSG00000140905	DDG2P_v2.2 panel
GDF1	ENSG00000130283	DDG2P_v2.2 panel
GDF3	ENSG00000184344	DDG2P_v2.2 panel

GDF5	ENSG00000125965	DDG2P_v2.2 panel
GDF6	ENSG00000156466	DDG2P_v2.2 panel
GDI1	ENSG00000203879	DDG2P_v2.2 panel
GFAP	ENSG00000131095	DDG2P_v2.2 panel
GFER	ENSG00000127554	DDG2P_v2.2 panel
GFM1	ENSG00000168827	DDG2P_v2.2 panel
GHR	ENSG00000112964	DDG2P_v2.2 panel
GJA1	ENSG00000152661	DDG2P_v2.2 panel
GJA3	ENSG00000121743	DDG2P_v2.2 panel
GJA8	ENSG00000121634	DDG2P_v2.2 panel
GJB2	ENSG00000165474	DDG2P_v2.2 panel
GJB3	ENSG00000188910	DDG2P_v2.2 panel
GJC2	ENSG00000198835	DDG2P_v2.2 panel
GK	ENSG00000198814	DDG2P_v2.2 panel
GLB1	ENSG00000170266	DDG2P_v2.2 panel
GLDC	ENSG00000178445	DDG2P_v2.2 panel
GLDN	ENSG00000186417	DDG2P_v2.2 panel
GLE1	ENSG00000119392	DDG2P_v2.2 panel
GLI2	ENSG00000074047	DDG2P_v2.2 panel
GLI3	ENSG00000106571	DDG2P_v2.2 panel
GLIS2	ENSG00000126603	DDG2P_v2.2 panel
GLIS3	ENSG00000107249	DDG2P_v2.2 panel
GLMN	ENSG00000174842	DDG2P_v2.2 panel
GLUD1	ENSG00000148672	DDG2P_v2.2 panel
GLUL	ENSG00000135821	DDG2P_v2.2 panel
GM2A	ENSG00000196743	DDG2P_v2.2 panel
GMNN	ENSG00000112312	DDG2P_v2.2 panel
GMPPA	ENSG00000144591	DDG2P_v2.2 panel
GMPPB	ENSG00000173540	DDG2P_v2.2 panel
GNA11	ENSG00000088256	DDG2P_v2.2 panel
GNA14	ENSG00000156049	DDG2P_v2.2 panel
GNAI1	ENSG00000127955	DDG2P_v2.2 panel
GNAI3	ENSG00000065135	DDG2P_v2.2 panel
GNAO1	ENSG00000087258	DDG2P_v2.2 panel
GNAQ	ENSG00000156052	DDG2P_v2.2 panel
GNAS	ENSG00000087460	DDG2P_v2.2 panel
GNB1	ENSG00000078369	DDG2P_v2.2 panel
GNB3	ENSG00000111664	DDG2P_v2.2 panel
GNB5	ENSG00000069966	DDG2P_v2.2 panel
GNPAT	ENSG00000116906	DDG2P_v2.2 panel
GNPTAB	ENSG00000111670	DDG2P_v2.2 panel
GNPTG	ENSG00000090581	DDG2P_v2.2 panel
GNS	ENSG00000135677	DDG2P_v2.2 panel
GON4L	ENSG00000116580	DDG2P_v2.2 panel
GORAB	ENSG00000120370	DDG2P_v2.2 panel
GOT2	ENSG00000125166	DDG2P_v2.2 panel
GPAA1	ENSG00000197858	DDG2P_v2.2 panel
GPC3	ENSG00000147257	DDG2P_v2.2 panel
GPC4	ENSG00000076716	DDG2P_v2.2 panel
GPC6	ENSG00000183098	DDG2P_v2.2 panel
GPI	ENSG00000105220	Lord et. al.
GPSM2	ENSG00000121957	DDG2P_v2.2 panel
GPX4	ENSG00000167468	DDG2P_v2.2 panel
GRHL2	ENSG00000083307	DDG2P_v2.2 panel
GRHL3	ENSG00000158055	DDG2P_v2.2 panel
GRIA3	ENSG00000125675	DDG2P_v2.2 panel
GRIA4	ENSG00000152578	DDG2P_v2.2 panel
GRIK2	ENSG00000164418	DDG2P_v2.2 panel
GRIN1	ENSG00000176884	DDG2P_v2.2 panel
GRIN2A	ENSG00000183454	DDG2P_v2.2 panel
GRIN2B	ENSG00000273079	DDG2P_v2.2 panel

GRIN2D	ENSG00000105464	DDG2P_v2.2 panel
GRIP1	ENSG00000155974	Lord et. al.
GRM1	ENSG00000152822	DDG2P_v2.2 panel
GRM6	ENSG00000113262	DDG2P_v2.2 panel
GSPT2	ENSG00000189369	DDG2P_v2.2 panel
GTF2E2	ENSG00000197265	DDG2P_v2.2 panel
GTF2H5	ENSG00000272047	DDG2P_v2.2 panel
GTPBP3	ENSG00000130299	DDG2P_v2.2 panel
GUCY2C	ENSG00000070019	DDG2P_v2.2 panel
GUSB	ENSG00000169919	DDG2P_v2.2 panel
GZF1	ENSG00000125812	DDG2P_v2.2 panel
H19	ENSG00000130600	Lord et. al.
H3F3A	ENSG00000163041	DDG2P_v2.2 panel
HACE1	ENSG00000085382	DDG2P_v2.2 panel
HADH	ENSG00000138796	DDG2P_v2.2 panel
HADHA	ENSG00000084754	DDG2P_v2.2 panel
HARS	ENSG00000170445	DDG2P_v2.2 panel
HAX1	ENSG00000143575	DDG2P_v2.2 panel
HCCS	ENSG00000004961	DDG2P_v2.2 panel
HCFC1	ENSG00000172534	DDG2P_v2.2 panel
HCN1	ENSG00000164588	DDG2P_v2.2 panel
HDAC4	ENSG00000068024	DDG2P_v2.2 panel
HDAC8	ENSG00000147099	DDG2P_v2.2 panel
HECW2	ENSG00000138411	DDG2P_v2.2 panel
HES7	ENSG00000179111	Lord et. al.
HESX1	ENSG00000163666	DDG2P_v2.2 panel
HEXA	ENSG00000213614	DDG2P_v2.2 panel
HEXB	ENSG00000049860	DDG2P_v2.2 panel
HGSNAT	ENSG00000165102	DDG2P_v2.2 panel
HIBCH	ENSG00000198130	DDG2P_v2.2 panel
HINT1	ENSG00000169567	DDG2P_v2.2 panel
HIST1H1E	ENSG00000168298	DDG2P_v2.2 panel
HIST1H4B	ENSG00000278705	DDG2P_v2.2 panel
HIST1H4C	ENSG00000197061	DDG2P_v2.2 panel
HIST1H4J	ENSG00000197238	DDG2P_v2.2 panel
HIST3H3	ENSG00000168148	DDG2P_v2.2 panel
HIVEP2	ENSG00000010818	DDG2P_v2.2 panel
HLCS	ENSG00000159267	DDG2P_v2.2 panel
HMGB3	ENSG00000029993	DDG2P_v2.2 panel
HMGCL	ENSG00000117305	DDG2P_v2.2 panel
HMGCS2	ENSG00000134240	DDG2P_v2.2 panel
HMX1	ENSG00000215612	DDG2P_v2.2 panel
HNF1B	ENSG00000275410	DDG2P_v2.2 panel
HNF4A	ENSG00000101076	DDG2P_v2.2 panel
HNRNPH2	ENSG00000126945	DDG2P_v2.2 panel
HNRNPK	ENSG00000165119	DDG2P_v2.2 panel
HNRNPR	ENSG00000125944	DDG2P_v2.2 panel
HNRNPU	ENSG00000153187	DDG2P_v2.2 panel
HOXA1	ENSG00000105991	DDG2P_v2.2 panel
HOXA11	ENSG00000005073	DDG2P_v2.2 panel
HOXA13	ENSG00000106031	DDG2P_v2.2 panel
HOXB1	ENSG00000120094	DDG2P_v2.2 panel
HOXC13	ENSG00000123364	DDG2P_v2.2 panel
HOXD13	ENSG00000128714	DDG2P_v2.2 panel
HPD	ENSG00000158104	DDG2P_v2.2 panel
HPGD	ENSG00000164120	DDG2P_v2.2 panel
HPRT1	ENSG00000165704	DDG2P_v2.2 panel
HPS1	ENSG00000107521	DDG2P_v2.2 panel
HPSE2	ENSG00000172987	DDG2P_v2.2 panel
HR	ENSG00000168453	DDG2P_v2.2 panel
HRAS	ENSG00000174775	DDG2P_v2.2 panel

HSD17B10	ENSG00000072506	DDG2P_v2.2 panel
HSD17B3	ENSG00000130948	Lord et. al.
HSD17B4	ENSG00000133835	DDG2P_v2.2 panel
HSD3B7	ENSG00000099377	DDG2P_v2.2 panel
HSF4	ENSG00000102878	DDG2P_v2.2 panel
HSPD1	ENSG00000144381	DDG2P_v2.2 panel
HSPG2	ENSG00000142798	DDG2P_v2.2 panel
HTRA2	ENSG00000115317	DDG2P_v2.2 panel
HUWE1	ENSG00000086758	DDG2P_v2.2 panel
HYAL1	ENSG00000114378	DDG2P_v2.2 panel
HYDIN	ENSG00000157423	DDG2P_v2.2 panel
HYLS1	ENSG00000198331	DDG2P_v2.2 panel
IARS	ENSG00000196305	DDG2P_v2.2 panel
IARS2	ENSG00000067704	DDG2P_v2.2 panel
IDS	ENSG00000010404	DDG2P_v2.2 panel
IDUA	ENSG00000127415	DDG2P_v2.2 panel
IER3IP1	ENSG00000134049	Lord et. al.
IFIH1	ENSG00000115267	DDG2P_v2.2 panel
IFITM5	ENSG00000206013	DDG2P_v2.2 panel
IFT122	ENSG00000163913	DDG2P_v2.2 panel
IFT140	ENSG00000187535	DDG2P_v2.2 panel
IFT172	ENSG00000138002	DDG2P_v2.2 panel
IFT43	ENSG00000119650	DDG2P_v2.2 panel
IFT80	ENSG00000068885	DDG2P_v2.2 panel
IGBP1	ENSG00000089289	DDG2P_v2.2 panel
IGF1	ENSG00000017427	DDG2P_v2.2 panel
IGF1R	ENSG00000140443	DDG2P_v2.2 panel
IGF2	ENSG00000167244	DDG2P_v2.2 panel
IGFBP7	ENSG00000163453	DDG2P_v2.2 panel
IGHMBP2	ENSG00000132740	DDG2P_v2.2 panel
IGSF1	ENSG00000147255	DDG2P_v2.2 panel
IHH	ENSG00000163501	DDG2P_v2.2 panel
IKBKG	ENSG00000269335	DDG2P_v2.2 panel
IL11	ENSG00000095752	DDG2P_v2.2 panel
IL11RA	ENSG00000137070	DDG2P_v2.2 panel
IL1RAPL1	ENSG00000169306	DDG2P_v2.2 panel
IMPAD1	ENSG00000104331	DDG2P_v2.2 panel
INPP4A	ENSG00000040933	DDG2P_v2.2 panel
INPP5E	ENSG00000148384	DDG2P_v2.2 panel
INPP5K	ENSG00000132376	DDG2P_v2.2 panel
INPPL1	ENSG00000165458	DDG2P_v2.2 panel
INSR	ENSG00000171105	Lord et. al.
INVS	ENSG00000119509	Lord et. al.
IQCB1	ENSG00000173226	Lord et. al.
IQSEC2	ENSG00000124313	DDG2P_v2.2 panel
IRF2BPL	ENSG00000119669	DDG2P_v2.2 panel
IRF6	ENSG00000117595	DDG2P_v2.2 panel
IRX5	ENSG00000176842	DDG2P_v2.2 panel
ISPD	ENSG00000214960	DDG2P_v2.2 panel
ITCH	ENSG00000078747	DDG2P_v2.2 panel
ITGA3	ENSG00000005884	DDG2P_v2.2 panel
ITGA6	ENSG00000091409	DDG2P_v2.2 panel and Lord et. al.
ITGA7	ENSG00000135424	DDG2P_v2.2 panel
ITGA8	ENSG00000077943	DDG2P_v2.2 panel
ITGB4	ENSG00000132470	Lord et. al.
ITPR1	ENSG00000150995	DDG2P_v2.2 panel
IVD	ENSG00000128928	DDG2P_v2.2 panel
JAG1	ENSG00000101384	DDG2P_v2.2 panel
JAGN1	ENSG00000171135	DDG2P_v2.2 panel
JAK3	ENSG00000105639	DDG2P_v2.2 panel
JAM3	ENSG00000166086	DDG2P_v2.2 panel

KANK1	ENSG00000107104	DDG2P_v2.2 panel
KANSL1	ENSG00000120071	DDG2P_v2.2 panel
KARS	ENSG00000065427	DDG2P_v2.2 panel
KAT6A	ENSG00000083168	DDG2P_v2.2 panel
KAT6B	ENSG00000156650	DDG2P_v2.2 panel
KBTBD13	ENSG00000234438	DDG2P_v2.2 panel
KCNA2	ENSG00000177301	DDG2P_v2.2 panel
KCNA4	ENSG00000182255	DDG2P_v2.2 panel
KCNB1	ENSG00000158445	DDG2P_v2.2 panel
KCNC1	ENSG00000129159	DDG2P_v2.2 panel
KCNC3	ENSG00000131398	DDG2P_v2.2 panel
KCNE1	ENSG00000180509	DDG2P_v2.2 panel
KCNH1	ENSG00000143473	DDG2P_v2.2 panel
KCNH5	ENSG00000140015	DDG2P_v2.2 panel
KCNJ1	ENSG00000151704	Lord et. al.
KCNJ10	ENSG00000177807	DDG2P_v2.2 panel
KCNJ11	ENSG00000187486	DDG2P_v2.2 panel
KCNJ2	ENSG00000123700	Lord et. al.
KCNJ6	ENSG00000157542	DDG2P_v2.2 panel
KCNJ8	ENSG00000121361	DDG2P_v2.2 panel
KCNK4	ENSG00000182450	DDG2P_v2.2 panel
KCNK9	ENSG00000169427	DDG2P_v2.2 panel
KCNMA1	ENSG00000156113	DDG2P_v2.2 panel
KCNN3	ENSG00000143603	DDG2P_v2.2 panel
KCNQ1	ENSG00000053918	DDG2P_v2.2 panel
KCNQ2	ENSG00000075043	DDG2P_v2.2 panel
KCNQ3	ENSG00000184156	DDG2P_v2.2 panel
KCNQ5	ENSG00000185760	DDG2P_v2.2 panel
KCNT1	ENSG00000107147	DDG2P_v2.2 panel
KCNT2	ENSG00000162687	DDG2P_v2.2 panel
KCTD1	ENSG00000134504	DDG2P_v2.2 panel
KCTD7	ENSG00000243335	DDG2P_v2.2 panel
KDM1A	ENSG00000004487	DDG2P_v2.2 panel
KDM3B	ENSG00000120733	DDG2P_v2.2 panel
KDM5A	ENSG00000073614	DDG2P_v2.2 panel
KDM5B	ENSG00000117139	DDG2P_v2.2 panel
KDM5C	ENSG00000126012	DDG2P_v2.2 panel
KDM6A	ENSG00000147050	DDG2P_v2.2 panel
KDM6B	ENSG00000132510	DDG2P_v2.2 panel
KIAA0586	ENSG00000100578	DDG2P_v2.2 panel
KIAA1109	ENSG00000138688	DDG2P_v2.2 panel
KIDINS220	ENSG00000134313	DDG2P_v2.2 panel
KIF11	ENSG00000138160	DDG2P_v2.2 panel
KIF14	ENSG00000118193	DDG2P_v2.2 panel
KIF1A	ENSG00000130294	DDG2P_v2.2 panel
KIF1BP	ENSG00000198954	DDG2P_v2.2 panel
KIF22	ENSG00000079616	DDG2P_v2.2 panel
KIF2A	ENSG00000068796	DDG2P_v2.2 panel
KIF4A	ENSG00000090889	DDG2P_v2.2 panel
KIF5C	ENSG00000168280	DDG2P_v2.2 panel
KIF7	ENSG00000166813	DDG2P_v2.2 panel
KIRREL3	ENSG00000149571	DDG2P_v2.2 panel
KISS1R	ENSG00000116014	Lord et. al.
KIT	ENSG00000157404	DDG2P_v2.2 panel
KITLG	ENSG00000049130	DDG2P_v2.2 panel
KLF1	ENSG00000105610	DDG2P_v2.2 panel
KLF8	ENSG00000102349	DDG2P_v2.2 panel
KLHL15	ENSG00000174010	DDG2P_v2.2 panel
KLHL40	ENSG00000157119	DDG2P_v2.2 panel
KLHL41	ENSG00000239474	Lord et. al.
KLHL7	ENSG00000122550	DDG2P_v2.2 panel

KMT2A	ENSG00000118058	DDG2P_v2.2 panel
KMT2B	ENSG00000272333	DDG2P_v2.2 panel
KMT2C	ENSG00000055609	DDG2P_v2.2 panel
KMT2D	ENSG00000167548	DDG2P_v2.2 panel
KMT2E	ENSG00000005483	DDG2P_v2.2 panel
KMT5B	ENSG00000110066	DDG2P_v2.2 panel
KPNA7	ENSG00000185467	DDG2P_v2.2 panel
KPTN	ENSG00000118162	DDG2P_v2.2 panel
KRAS	ENSG00000133703	DDG2P_v2.2 panel
KRIT1	ENSG00000001631	DDG2P_v2.2 panel
KRT74	ENSG00000170484	DDG2P_v2.2 panel
L1CAM	ENSG00000198910	DDG2P_v2.2 panel
L2HGDH	ENSG00000087299	DDG2P_v2.2 panel
LAGE3	ENSG00000196976	DDG2P_v2.2 panel
LAMA1	ENSG00000101680	DDG2P_v2.2 panel
LAMA2	ENSG00000196569	DDG2P_v2.2 panel
LAMB1	ENSG00000091136	DDG2P_v2.2 panel
LAMC3	ENSG00000050555	DDG2P_v2.2 panel
LAMP2	ENSG00000005893	DDG2P_v2.2 panel
LARGE1	ENSG00000133424	DDG2P_v2.2 panel
LARP7	ENSG00000174720	DDG2P_v2.2 panel
LARS2	ENSG00000011376	DDG2P_v2.2 panel
LAS1L	ENSG00000001497	DDG2P_v2.2 panel
LBR	ENSG00000143815	DDG2P_v2.2 panel
LDB3	ENSG00000122367	DDG2P_v2.2 panel
LEFTY2	ENSG00000143768	DDG2P_v2.2 panel
LEMD2	ENSG00000161904	DDG2P_v2.2 panel
LEMD3	ENSG00000174106	DDG2P_v2.2 panel
LFNG	ENSG00000106003	DDG2P_v2.2 panel
LGI1	ENSG00000108231	DDG2P_v2.2 panel
LGI4	ENSG00000153902	DDG2P_v2.2 panel
LHX3	ENSG00000107187	DDG2P_v2.2 panel
LHX4	ENSG00000121454	DDG2P_v2.2 panel
LIAS	ENSG00000121897	DDG2P_v2.2 panel
LIFR	ENSG00000113594	Lord et. al.
LIG4	ENSG00000174405	DDG2P_v2.2 panel
LINGO1	ENSG00000169783	DDG2P_v2.2 panel
LINS1	ENSG00000140471	DDG2P_v2.2 panel
LIPN	ENSG00000204020	DDG2P_v2.2 panel
LIPT1	ENSG00000144182	DDG2P_v2.2 panel
LIPT2	ENSG00000175536	DDG2P_v2.2 panel
LMBR1	ENSG00000105983	Lord et. al.
LMBRD1	ENSG00000168216	DDG2P_v2.2 panel
LMNA	ENSG00000160789	DDG2P_v2.2 panel
LMOD3	ENSG00000163380	Lord et. al.
LMX1B	ENSG00000136944	DDG2P_v2.2 panel
LNPK	ENSG00000144320	DDG2P_v2.2 panel
LONP1	ENSG00000196365	DDG2P_v2.2 panel
LRAT	ENSG00000121207	DDG2P_v2.2 panel
LRBA	ENSG00000198589	DDG2P_v2.2 panel
LRIG2	ENSG00000198799	DDG2P_v2.2 panel
LRIT3	ENSG00000183423	DDG2P_v2.2 panel
LRP2	ENSG00000081479	DDG2P_v2.2 panel
LRP4	ENSG00000134569	DDG2P_v2.2 panel
LRP5	ENSG00000162337	DDG2P_v2.2 panel
LRP6	ENSG00000070018	DDG2P_v2.2 panel
LRPAP1	ENSG00000163956	DDG2P_v2.2 panel
LRPPRC	ENSG00000138095	DDG2P_v2.2 panel
LRRC56	ENSG00000161328	DDG2P_v2.2 panel
LRRC6	ENSG00000129295	DDG2P_v2.2 panel
LTBP2	ENSG00000119681	DDG2P_v2.2 panel

LTBP3	ENSG00000168056	DDG2P_v2.2 panel
LTBP4	ENSG00000090006	Lord et. al.
LYST	ENSG00000143669	DDG2P_v2.2 panel
LZTFL1	ENSG00000163818	Lord et. al.
LZTR1	ENSG00000099949	DDG2P_v2.2 panel
MAB21L1	ENSG00000180660	DDG2P_v2.2 panel
MAB21L2	ENSG00000181541	DDG2P_v2.2 panel
MACF1	ENSG00000127603	DDG2P_v2.2 panel
MAF	ENSG00000178573	DDG2P_v2.2 panel
MAFB	ENSG00000204103	DDG2P_v2.2 panel
MAGEL2	ENSG00000254585	DDG2P_v2.2 panel
MAGI2	ENSG00000187391	DDG2P_v2.2 panel
MAGT1	ENSG00000102158	DDG2P_v2.2 panel
MAMLD1	ENSG00000013619	DDG2P_v2.2 panel
MAN1B1	ENSG00000177239	DDG2P_v2.2 panel
MAN2B1	ENSG00000104774	DDG2P_v2.2 panel
MANBA	ENSG00000109323	DDG2P_v2.2 panel
MAOA	ENSG00000189221	DDG2P_v2.2 panel
MAP2K1	ENSG00000169032	DDG2P_v2.2 panel
MAP2K2	ENSG00000126934	DDG2P_v2.2 panel
MAP3K1	ENSG00000095015	DDG2P_v2.2 panel
MAP3K7	ENSG00000135341	DDG2P_v2.2 panel
MAPK10	ENSG00000109339	DDG2P_v2.2 panel
MAPK8IP3	ENSG00000138834	DDG2P_v2.2 panel
MAPRE2	ENSG00000166974	DDG2P_v2.2 panel
MASP1	ENSG00000127241	DDG2P_v2.2 panel
MAT1A	ENSG00000151224	DDG2P_v2.2 panel
MATN3	ENSG00000132031	DDG2P_v2.2 panel
MBD5	ENSG00000204406	DDG2P_v2.2 panel
MBOAT7	ENSG00000125505	DDG2P_v2.2 panel
MBTPS2	ENSG00000012174	Lord et. al.
MC2R	ENSG00000185231	DDG2P_v2.2 panel
MCCC1	ENSG00000078070	DDG2P_v2.2 panel
MCCC2	ENSG00000131844	DDG2P_v2.2 panel
MCEE	ENSG00000124370	DDG2P_v2.2 panel
MCOLN1	ENSG00000090674	DDG2P_v2.2 panel
MCPH1	ENSG00000147316	DDG2P_v2.2 panel
MDH2	ENSG00000146701	DDG2P_v2.2 panel
MECOM	ENSG00000085276	DDG2P_v2.2 panel
MECP2	ENSG00000169057	DDG2P_v2.2 panel
MECR	ENSG00000116353	DDG2P_v2.2 panel
MED12	ENSG00000184634	DDG2P_v2.2 panel
MED13	ENSG00000108510	DDG2P_v2.2 panel
MED13L	ENSG00000123066	DDG2P_v2.2 panel
MED17	ENSG00000042429	DDG2P_v2.2 panel
MED23	ENSG00000112282	DDG2P_v2.2 panel
MEF2C	ENSG00000081189	DDG2P_v2.2 panel
MEGF10	ENSG00000145794	DDG2P_v2.2 panel
MEGF8	ENSG00000105429	DDG2P_v2.2 panel
MEOX1	ENSG00000005102	DDG2P_v2.2 panel
MESD	ENSG00000117899	DDG2P_v2.2 panel
MESP2	ENSG00000188095	DDG2P_v2.2 panel
METTL5	ENSG00000138382	DDG2P_v2.2 panel
MFRP	ENSG00000235718	DDG2P_v2.2 panel
MFSD2A	ENSG00000168389	DDG2P_v2.2 panel
MFSD8	ENSG00000164073	DDG2P_v2.2 panel
MGAT2	ENSG00000168282	DDG2P_v2.2 panel
MGP	ENSG00000111341	DDG2P_v2.2 panel
MICU1	ENSG00000107745	DDG2P_v2.2 panel
MID1	ENSG00000101871	DDG2P_v2.2 panel
MIR17HG	ENSG00000215417	DDG2P_v2.2 panel

MIR184	ENSG00000207695	DDG2P_v2.2 panel
MITF	ENSG00000187098	DDG2P_v2.2 panel
MKKS	ENSG00000125863	DDG2P_v2.2 panel
MKS1	ENSG00000011143	DDG2P_v2.2 panel
MLC1	ENSG00000100427	DDG2P_v2.2 panel
MLH1	ENSG00000076242	Lord et. al.
MLYCD	ENSG00000103150	DDG2P_v2.2 panel
MMAA	ENSG00000151611	DDG2P_v2.2 panel
MMAB	ENSG00000139428	DDG2P_v2.2 panel
MMACHC	ENSG00000132763	DDG2P_v2.2 panel
MMADHC	ENSG00000168288	DDG2P_v2.2 panel
MMP13	ENSG00000137745	DDG2P_v2.2 panel
MMP14	ENSG00000157227	DDG2P_v2.2 panel
MMP21	ENSG00000154485	DDG2P_v2.2 panel
MN1	ENSG00000169184	DDG2P_v2.2 panel
MNX1	ENSG00000130675	DDG2P_v2.2 panel
MOCS1	ENSG00000124615	DDG2P_v2.2 panel
MOCS2	ENSG00000164172	DDG2P_v2.2 panel
MOGS	ENSG00000115275	DDG2P_v2.2 panel
MORC2	ENSG00000133422	DDG2P_v2.2 panel
MPDU1	ENSG00000129255	DDG2P_v2.2 panel
MPDZ	ENSG00000107186	DDG2P_v2.2 panel
MPI	ENSG00000178802	DDG2P_v2.2 panel
MPLKIP	ENSG00000168303	DDG2P_v2.2 panel
MPV17	ENSG00000115204	DDG2P_v2.2 panel
MRE11	ENSG00000020922	DDG2P_v2.2 panel
MRPS2	ENSG00000122140	DDG2P_v2.2 panel
MRPS22	ENSG00000175110	DDG2P_v2.2 panel
MRPS34	ENSG00000074071	DDG2P_v2.2 panel
MSH2	ENSG00000095002	Lord et. al.
MSH6	ENSG00000116062	Lord et. al.
MSI1	ENSG00000135097	DDG2P_v2.2 panel
MSL3	ENSG00000005302	DDG2P_v2.2 panel
MSX1	ENSG00000163132	DDG2P_v2.2 panel
MSX2	ENSG00000120149	DDG2P_v2.2 panel
MTF1	ENSG00000188786	DDG2P_v2.2 panel
MTHFR	ENSG00000177000	DDG2P_v2.2 panel
MTM1	ENSG00000171100	DDG2P_v2.2 panel
MTMR14	ENSG00000163719	DDG2P_v2.2 panel
MTO1	ENSG00000135297	DDG2P_v2.2 panel
MTOR	ENSG00000198793	DDG2P_v2.2 panel
MTR	ENSG00000116984	DDG2P_v2.2 panel
MTRR	ENSG00000124275	DDG2P_v2.2 panel
MT-TP	ENSG00000210196	DDG2P_v2.2 panel
MUSK	ENSG00000030304	Lord et. al.
MUT	ENSG00000146085	DDG2P_v2.2 panel
MYBPC1	ENSG00000196091	Lord et. al.
MYCN	ENSG00000134323	DDG2P_v2.2 panel
MYF5	ENSG00000111049	DDG2P_v2.2 panel
MYH10	ENSG00000133026	DDG2P_v2.2 panel
MYH11	ENSG00000133392	DDG2P_v2.2 panel
MYH3	ENSG00000109063	DDG2P_v2.2 panel
MYH6	ENSG00000197616	DDG2P_v2.2 panel
MYH8	ENSG00000133020	DDG2P_v2.2 panel
MYH9	ENSG00000100345	DDG2P_v2.2 panel
MYLK	ENSG00000065534	DDG2P_v2.2 panel
MYO5A	ENSG00000197535	DDG2P_v2.2 panel
MYO5B	ENSG00000167306	DDG2P_v2.2 panel
MYO7A	ENSG00000137474	DDG2P_v2.2 panel
MYOC	ENSG00000034971	DDG2P_v2.2 panel
MYOCD	ENSG00000141052	DDG2P_v2.2 panel

MYPN	ENSG00000138347	DDG2P_v2.2 panel
MYRF	ENSG00000124920	DDG2P_v2.2 panel
MYT1	ENSG00000196132	DDG2P_v2.2 panel
MYT1L	ENSG00000186487	DDG2P_v2.2 panel
NAA10	ENSG00000102030	DDG2P_v2.2 panel
NAA15	ENSG00000164134	DDG2P_v2.2 panel
NACC1	ENSG00000160877	DDG2P_v2.2 panel
NADK2	ENSG00000152620	DDG2P_v2.2 panel
NAGA	ENSG00000198951	DDG2P_v2.2 panel
NAGLU	ENSG00000108784	DDG2P_v2.2 panel
NAGS	ENSG00000161653	DDG2P_v2.2 panel
NALCN	ENSG00000102452	DDG2P_v2.2 panel
NANS	ENSG00000095380	DDG2P_v2.2 panel
NAXD	ENSG00000213995	DDG2P_v2.2 panel
NAXE	ENSG00000163382	DDG2P_v2.2 panel
NBAS	ENSG00000151779	DDG2P_v2.2 panel
NBEA	ENSG00000172915	DDG2P_v2.2 panel
NBN	ENSG00000104320	DDG2P_v2.2 panel
NCAPD2	ENSG00000010292	DDG2P_v2.2 panel
NCAPD3	ENSG00000151503	DDG2P_v2.2 panel
NCAPG2	ENSG00000146918	DDG2P_v2.2 panel
NCAPH	ENSG00000121152	DDG2P_v2.2 panel
NDE1	ENSG00000072864	DDG2P_v2.2 panel
NDP	ENSG00000124479	DDG2P_v2.2 panel
NDST1	ENSG00000070614	DDG2P_v2.2 panel
NDUFA1	ENSG00000125356	DDG2P_v2.2 panel
NDUFA10	ENSG00000130414	DDG2P_v2.2 panel
NDUFA6	ENSG00000184983	DDG2P_v2.2 panel
NDUFA9	ENSG00000139180	DDG2P_v2.2 panel
NDUFAF2	ENSG00000164182	DDG2P_v2.2 panel
NDUFB11	ENSG00000147123	DDG2P_v2.2 panel
NDUFB8	ENSG00000166136	DDG2P_v2.2 panel
NDUFS1	ENSG00000023228	DDG2P_v2.2 panel
NDUFS4	ENSG00000164258	DDG2P_v2.2 panel
NDUFS7	ENSG00000115286	DDG2P_v2.2 panel
NDUFS8	ENSG00000110717	DDG2P_v2.2 panel
NDUFV1	ENSG00000167792	DDG2P_v2.2 panel
NEB	ENSG00000183091	DDG2P_v2.2 panel
NECTIN1	ENSG00000110400	DDG2P_v2.2 panel
NECTIN4	ENSG00000143217	DDG2P_v2.2 panel
NEDD4L	ENSG00000049759	DDG2P_v2.2 panel
NEK1	ENSG00000137601	DDG2P_v2.2 panel
NEK8	ENSG00000160602	DDG2P_v2.2 panel
NEU1	ENSG00000204386	DDG2P_v2.2 panel
NEXMIF	ENSG00000050030	DDG2P_v2.2 panel
NF1	ENSG00000196712	DDG2P_v2.2 panel
NFIA	ENSG00000162599	DDG2P_v2.2 panel
NFIB	ENSG00000147862	DDG2P_v2.2 panel
NFIX	ENSG00000008441	DDG2P_v2.2 panel
NFU1	ENSG00000169599	DDG2P_v2.2 panel
NGLY1	ENSG00000151092	DDG2P_v2.2 panel
NHEJ1	ENSG00000187736	Lord et. al.
NHP2	ENSG00000145912	DDG2P_v2.2 panel
NHS	ENSG00000188158	DDG2P_v2.2 panel
NIPBL	ENSG00000164190	DDG2P_v2.2 panel
NKX2-1	ENSG00000136352	DDG2P_v2.2 panel
NKX2-5	ENSG00000183072	DDG2P_v2.2 panel
NKX3-2	ENSG00000109705	DDG2P_v2.2 panel
NKX6-2	ENSG00000148826	DDG2P_v2.2 panel
NLGN3	ENSG00000196338	DDG2P_v2.2 panel
NLGN4X	ENSG00000146938	DDG2P_v2.2 panel

NLRP5	ENSG00000171487	DDG2P_v2.2 panel
NMNAT1	ENSG00000173614	DDG2P_v2.2 panel
NODAL	ENSG00000156574	DDG2P_v2.2 panel
NOG	ENSG00000183691	DDG2P_v2.2 panel
NONO	ENSG00000147140	DDG2P_v2.2 panel
NOP10	ENSG00000182117	DDG2P_v2.2 panel
NOTCH1	ENSG00000148400	DDG2P_v2.2 panel
NOTCH2	ENSG00000134250	DDG2P_v2.2 panel
NOTCH3	ENSG00000074181	DDG2P_v2.2 panel
NOVA2	ENSG00000104967	DDG2P_v2.2 panel
NPC1	ENSG00000141458	DDG2P_v2.2 panel
NPC2	ENSG00000119655	DDG2P_v2.2 panel
NPHP1	ENSG00000144061	DDG2P_v2.2 panel
NPHP3	ENSG00000113971	DDG2P_v2.2 panel
NPHP4	ENSG00000131697	DDG2P_v2.2 panel
NPHS1	ENSG00000161270	DDG2P_v2.2 panel
NPHS2	ENSG00000116218	DDG2P_v2.2 panel
NPM1	ENSG00000181163	DDG2P_v2.2 panel
NPR2	ENSG00000159899	DDG2P_v2.2 panel
NPR3	ENSG00000113389	DDG2P_v2.2 panel
NR0B1	ENSG00000169297	Lord et. al.
NR1I3	ENSG00000143257	DDG2P_v2.2 panel
NR2F1	ENSG00000175745	DDG2P_v2.2 panel
NR2F2	ENSG00000185551	DDG2P_v2.2 panel
NR5A1	ENSG00000136931	DDG2P_v2.2 panel
NRAS	ENSG00000213281	DDG2P_v2.2 panel
NRXN1	ENSG00000179915	DDG2P_v2.2 panel
NRXN2	ENSG00000110076	DDG2P_v2.2 panel
NRXN3	ENSG00000021645	DDG2P_v2.2 panel
NSD1	ENSG00000165671	DDG2P_v2.2 panel
NSDHL	ENSG00000147383	DDG2P_v2.2 panel
NSMCE3	ENSG00000185115	DDG2P_v2.2 panel
NSUN2	ENSG00000037474	DDG2P_v2.2 panel
NT5C3A	ENSG00000122643	DDG2P_v2.2 panel
NTRK1	ENSG00000198400	DDG2P_v2.2 panel
NTRK2	ENSG00000148053	DDG2P_v2.2 panel
NUBPL	ENSG00000151413	DDG2P_v2.2 panel
NUP107	ENSG00000111581	DDG2P_v2.2 panel
NUP133	ENSG00000069248	DDG2P_v2.2 panel
NUP214	ENSG00000126883	DDG2P_v2.2 panel
NUP62	ENSG00000213024	DDG2P_v2.2 panel
NUS1	ENSG00000153989	DDG2P_v2.2 panel
NYX	ENSG00000188937	DDG2P_v2.2 panel
OBSL1	ENSG00000124006	DDG2P_v2.2 panel
OCLN	ENSG00000197822	Lord et. al.
OCRL	ENSG00000122126	DDG2P_v2.2 panel
OFD1	ENSG00000046651	DDG2P_v2.2 panel
OPHN1	ENSG00000079482	DDG2P_v2.2 panel
ORC1	ENSG00000085840	DDG2P_v2.2 panel
ORC4	ENSG00000115947	DDG2P_v2.2 panel
ORC6	ENSG00000091651	DDG2P_v2.2 panel
OSGEP	ENSG00000092094	DDG2P_v2.2 panel
OSTM1	ENSG00000081087	Lord et. al.
OTC	ENSG00000036473	DDG2P_v2.2 panel
OTOGL	ENSG00000165899	DDG2P_v2.2 panel
OTUD6B	ENSG00000155100	DDG2P_v2.2 panel
OTUD7A	ENSG00000169918	DDG2P_v2.2 panel
OTULIN	ENSG00000154124	DDG2P_v2.2 panel
OTX2	ENSG00000165588	DDG2P_v2.2 panel
OXCT1	ENSG00000083720	DDG2P_v2.2 panel
P3H1	ENSG00000117385	DDG2P_v2.2 panel

P4HB	ENSG00000185624	DDG2P_v2.2 panel
PACS1	ENSG00000175115	DDG2P_v2.2 panel
PACS2	ENSG00000179364	DDG2P_v2.2 panel
PAFAH1B1	ENSG00000007168	DDG2P_v2.2 panel
PAH	ENSG00000171759	DDG2P_v2.2 panel
PAK1	ENSG00000149269	DDG2P_v2.2 panel
PAK3	ENSG00000077264	DDG2P_v2.2 panel
PALB2	ENSG00000083093	DDG2P_v2.2 panel
PAPSS2	ENSG00000198682	DDG2P_v2.2 panel
PARN	ENSG00000140694	DDG2P_v2.2 panel
PARP1	ENSG00000143799	DDG2P_v2.2 panel
PAX2	ENSG00000075891	DDG2P_v2.2 panel
PAX3	ENSG00000135903	DDG2P_v2.2 panel
PAX6	ENSG00000007372	DDG2P_v2.2 panel
PAX8	ENSG00000125618	DDG2P_v2.2 panel
PAX9	ENSG00000198807	DDG2P_v2.2 panel
PC	ENSG00000173599	DDG2P_v2.2 panel
PCBD1	ENSG00000166228	DDG2P_v2.2 panel
PCCA	ENSG00000175198	DDG2P_v2.2 panel
PCCB	ENSG00000114054	DDG2P_v2.2 panel
PCDH19	ENSG00000165194	DDG2P_v2.2 panel
PCGF2	ENSG00000277258	DDG2P_v2.2 panel
PCNT	ENSG00000160299	DDG2P_v2.2 panel
PCYT1A	ENSG00000161217	DDG2P_v2.2 panel
PDCD10	ENSG00000114209	DDG2P_v2.2 panel
PDE10A	ENSG00000112541	DDG2P_v2.2 panel
PDE4D	ENSG00000113448	DDG2P_v2.2 panel
PDE6G	ENSG00000185527	DDG2P_v2.2 panel
PDE6H	ENSG00000139053	DDG2P_v2.2 panel
PDGFRB	ENSG00000113721	DDG2P_v2.2 panel
PDHA1	ENSG00000131828	DDG2P_v2.2 panel
PDHX	ENSG00000110435	DDG2P_v2.2 panel
PDSS1	ENSG00000148459	DDG2P_v2.2 panel
PDSS2	ENSG00000164494	DDG2P_v2.2 panel
PECR	ENSG00000115425	DDG2P_v2.2 panel
PEPD	ENSG00000124299	DDG2P_v2.2 panel
PET100	ENSG00000229833	DDG2P_v2.2 panel
PEX1	ENSG00000127980	DDG2P_v2.2 panel
PEX10	ENSG00000157911	DDG2P_v2.2 panel
PEX11B	ENSG00000131779	DDG2P_v2.2 panel
PEX12	ENSG00000108733	DDG2P_v2.2 panel
PEX13	ENSG00000162928	DDG2P_v2.2 panel
PEX14	ENSG00000142655	DDG2P_v2.2 panel
PEX16	ENSG00000121680	DDG2P_v2.2 panel
PEX19	ENSG00000162735	DDG2P_v2.2 panel
PEX2	ENSG00000164751	DDG2P_v2.2 panel
PEX26	ENSG00000215193	DDG2P_v2.2 panel
PEX3	ENSG00000034693	DDG2P_v2.2 panel
PEX5	ENSG00000139197	DDG2P_v2.2 panel
PEX6	ENSG00000124587	DDG2P_v2.2 panel
PEX7	ENSG00000112357	DDG2P_v2.2 panel
PGAP1	ENSG00000197121	DDG2P_v2.2 panel
PGAP2	ENSG00000148985	DDG2P_v2.2 panel
PGAP3	ENSG00000161395	DDG2P_v2.2 panel
PGK1	ENSG00000102144	DDG2P_v2.2 panel
PGM1	ENSG00000079739	DDG2P_v2.2 panel
PGM3	ENSG00000013375	DDG2P_v2.2 panel
PHACTR1	ENSG00000112137	DDG2P_v2.2 panel
PHC1	ENSG00000111752	DDG2P_v2.2 panel
PHF21A	ENSG00000135365	DDG2P_v2.2 panel
PHF6	ENSG00000156531	DDG2P_v2.2 panel

PHF8	ENSG00000172943	DDG2P_v2.2 panel
PHGDH	ENSG00000092621	DDG2P_v2.2 panel
PHIP	ENSG00000146247	DDG2P_v2.2 panel
PHOX2B	ENSG00000109132	DDG2P_v2.2 panel
PIEZO1	ENSG00000103335	DDG2P_v2.2 panel and Lord et. al.
PIEZO2	ENSG00000154864	DDG2P_v2.2 panel
PIGA	ENSG00000165195	DDG2P_v2.2 panel
PIGB	ENSG00000069943	DDG2P_v2.2 panel
PIGG	ENSG00000174227	DDG2P_v2.2 panel
PIGL	ENSG00000108474	DDG2P_v2.2 panel
PIGM	ENSG00000143315	DDG2P_v2.2 panel
PIGN	ENSG00000197563	DDG2P_v2.2 panel
PIGO	ENSG00000165282	DDG2P_v2.2 panel
PIGQ	ENSG00000007541	DDG2P_v2.2 panel
PIGS	ENSG00000087111	DDG2P_v2.2 panel
PIGT	ENSG00000124155	DDG2P_v2.2 panel
PIGU	ENSG00000101464	DDG2P_v2.2 panel
PIGV	ENSG00000060642	DDG2P_v2.2 panel
PIGW	ENSG00000277161	DDG2P_v2.2 panel
PIGY	ENSG00000255072	DDG2P_v2.2 panel
PIH1D3	ENSG00000080572	DDG2P_v2.2 panel
PIK3CA	ENSG00000121879	DDG2P_v2.2 panel
PIK3R1	ENSG00000145675	DDG2P_v2.2 panel
PIK3R2	ENSG00000105647	DDG2P_v2.2 panel
PIP5K1C	ENSG00000186111	DDG2P_v2.2 panel
PITX1	ENSG00000069011	DDG2P_v2.2 panel
PITX2	ENSG00000164093	DDG2P_v2.2 panel
PITX3	ENSG00000107859	DDG2P_v2.2 panel
PKD1	ENSG00000008710	Lord et. al.
PKD1L1	ENSG00000158683	DDG2P_v2.2 panel
PKD2	ENSG00000118762	Lord et. al.
PKHD1	ENSG00000170927	DDG2P_v2.2 panel
PKLR	ENSG00000143627	Lord et. al.
PLA2G6	ENSG00000184381	DDG2P_v2.2 panel
PLAA	ENSG00000137055	DDG2P_v2.2 panel
PLCB1	ENSG00000182621	DDG2P_v2.2 panel
PLCB4	ENSG00000101333	DDG2P_v2.2 panel
PLCE1	ENSG00000138193	DDG2P_v2.2 panel
PLCG2	ENSG00000197943	DDG2P_v2.2 panel
PLEC	ENSG00000178209	DDG2P_v2.2 panel
PLK4	ENSG00000142731	DDG2P_v2.2 panel
PLOD1	ENSG00000083444	DDG2P_v2.2 panel
PLOD2	ENSG00000152952	DDG2P_v2.2 panel
PLOD3	ENSG00000106397	DDG2P_v2.2 panel
PLP1	ENSG00000123560	DDG2P_v2.2 panel
PLPBP	ENSG00000147471	DDG2P_v2.2 panel
PLXND1	ENSG00000004399	DDG2P_v2.2 panel
PMM2	ENSG00000140650	DDG2P_v2.2 panel
PMPCB	ENSG00000105819	DDG2P_v2.2 panel
PMS2	ENSG00000122512	DDG2P_v2.2 panel
PNKP	ENSG00000039650	DDG2P_v2.2 panel
PNPLA1	ENSG00000180316	DDG2P_v2.2 panel
PNPLA2	ENSG00000177666	DDG2P_v2.2 panel
PNPT1	ENSG00000138035	DDG2P_v2.2 panel
POC1A	ENSG00000164087	DDG2P_v2.2 panel
POC1B	ENSG00000139323	DDG2P_v2.2 panel
POGZ	ENSG00000143442	DDG2P_v2.2 panel
POLA1	ENSG00000101868	DDG2P_v2.2 panel
POLD1	ENSG00000062822	DDG2P_v2.2 panel
POLG	ENSG00000140521	DDG2P_v2.2 panel
POLR1A	ENSG00000068654	DDG2P_v2.2 panel

POLR1C	ENSG00000171453	DDG2P_v2.2 panel
POLR1D	ENSG00000186184	DDG2P_v2.2 panel
POLR2A	ENSG00000181222	DDG2P_v2.2 panel
POLR3A	ENSG00000148606	DDG2P_v2.2 panel
POLR3B	ENSG00000013503	DDG2P_v2.2 panel
POMGNT1	ENSG00000085998	DDG2P_v2.2 panel
POMGNT2	ENSG00000144647	DDG2P_v2.2 panel
POMK	ENSG00000185900	Lord et. al.
POMP	ENSG00000132963	DDG2P_v2.2 panel
POMT1	ENSG00000130714	DDG2P_v2.2 panel
POMT2	ENSG00000009830	DDG2P_v2.2 panel
POR	ENSG00000127948	Lord et. al.
PORCN	ENSG00000102312	DDG2P_v2.2 panel
POT1	ENSG00000128513	DDG2P_v2.2 panel
POU1F1	ENSG00000064835	DDG2P_v2.2 panel
POU3F3	ENSG00000198914	DDG2P_v2.2 panel
PPA2	ENSG00000138777	DDG2P_v2.2 panel
PPIB	ENSG00000166794	Lord et. al.
PPM1D	ENSG00000170836	DDG2P_v2.2 panel
PPP1CB	ENSG00000213639	DDG2P_v2.2 panel
PPP1R15B	ENSG00000158615	DDG2P_v2.2 panel
PPP2CA	ENSG00000113575	DDG2P_v2.2 panel
PPP2R1A	ENSG00000105568	DDG2P_v2.2 panel
PPP2R5D	ENSG00000112640	DDG2P_v2.2 panel
PPP3CA	ENSG00000138814	DDG2P_v2.2 panel
PPT1	ENSG00000131238	DDG2P_v2.2 panel
PQBP1	ENSG00000102103	DDG2P_v2.2 panel
PRDM12	ENSG00000130711	DDG2P_v2.2 panel
PRDM6	ENSG00000061455	DDG2P_v2.2 panel
PREPL	ENSG00000138078	DDG2P_v2.2 panel
PRG4	ENSG00000116690	Lord et. al.
PRKAR1A	ENSG00000108946	DDG2P_v2.2 panel
PRKD1	ENSG00000184304	DDG2P_v2.2 panel
PRMT7	ENSG00000132600	DDG2P_v2.2 panel
PRMT9	ENSG00000164169	DDG2P_v2.2 panel
PROK2	ENSG00000163421	Lord et. al.
PROKR2	ENSG00000101292	Lord et. al.
PROP1	ENSG00000175325	DDG2P_v2.2 panel
PRPS1	ENSG00000147224	DDG2P_v2.2 panel
PRR12	ENSG00000126464	DDG2P_v2.2 panel
PRRT2	ENSG00000167371	DDG2P_v2.2 panel
PRRX1	ENSG00000116132	DDG2P_v2.2 panel
PRSS12	ENSG00000164099	DDG2P_v2.2 panel
PRSS56	ENSG00000237412	DDG2P_v2.2 panel
PRUNE1	ENSG00000143363	DDG2P_v2.2 panel
PSAP	ENSG00000197746	DDG2P_v2.2 panel
PSAT1	ENSG00000135069	DDG2P_v2.2 panel
PSMB8	ENSG00000204264	DDG2P_v2.2 panel
PSMD12	ENSG00000197170	DDG2P_v2.2 panel
PSPH	ENSG00000146733	DDG2P_v2.2 panel
PTCH1	ENSG00000185920	DDG2P_v2.2 panel
PTCHD1	ENSG00000165186	DDG2P_v2.2 panel
PTDSS1	ENSG00000156471	DDG2P_v2.2 panel
PTEN	ENSG00000171862	DDG2P_v2.2 panel
PTF1A	ENSG00000168267	DDG2P_v2.2 panel
PTH	ENSG00000152266	DDG2P_v2.2 panel
PTH1R	ENSG00000160801	DDG2P_v2.2 panel
PTHLH	ENSG00000087494	DDG2P_v2.2 panel
PTPN11	ENSG00000179295	DDG2P_v2.2 panel
PTPN14	ENSG00000152104	DDG2P_v2.2 panel
PTPRF	ENSG00000142949	DDG2P_v2.2 panel

PTS	ENSG00000150787	DDG2P_v2.2 panel
PUF60	ENSG00000179950	DDG2P_v2.2 panel
PURA	ENSG00000185129	DDG2P_v2.2 panel
PUS7	ENSG00000091127	DDG2P_v2.2 panel
PXDN	ENSG00000130508	DDG2P_v2.2 panel
PYCR1	ENSG00000183010	DDG2P_v2.2 panel
PYCR2	ENSG00000143811	DDG2P_v2.2 panel
PYGL	ENSG00000100504	DDG2P_v2.2 panel
PYROXD1	ENSG00000121350	DDG2P_v2.2 panel
QARS	ENSG00000172053	DDG2P_v2.2 panel
QDPR	ENSG00000151552	DDG2P_v2.2 panel
QKI	ENSG00000112531	DDG2P_v2.2 panel
QRICH1	ENSG00000198218	DDG2P_v2.2 panel
RAB11A	ENSG00000103769	DDG2P_v2.2 panel
RAB11B	ENSG00000185236	DDG2P_v2.2 panel
RAB18	ENSG00000099246	DDG2P_v2.2 panel
RAB23	ENSG00000112210	DDG2P_v2.2 panel
RAB39B	ENSG00000155961	DDG2P_v2.2 panel
RAB3GAP1	ENSG00000115839	DDG2P_v2.2 panel
RAB3GAP2	ENSG00000118873	DDG2P_v2.2 panel
RABL6	ENSG00000196642	DDG2P_v2.2 panel
RAC1	ENSG00000136238	DDG2P_v2.2 panel
RAC3	ENSG00000169750	DDG2P_v2.2 panel
RAD21	ENSG00000164754	DDG2P_v2.2 panel
RAD50	ENSG00000113522	DDG2P_v2.2 panel
RAD51	ENSG00000051180	DDG2P_v2.2 panel
RAD51C	ENSG00000108384	DDG2P_v2.2 panel
RAF1	ENSG00000132155	DDG2P_v2.2 panel
RAI1	ENSG00000108557	DDG2P_v2.2 panel
RALGDS	ENSG00000160271	DDG2P_v2.2 panel
RANBP2	ENSG00000153201	DDG2P_v2.2 panel
RAPSN	ENSG00000165917	DDG2P_v2.2 panel
RARB	ENSG00000077092	DDG2P_v2.2 panel
RARS2	ENSG00000146282	DDG2P_v2.2 panel
RASA1	ENSG00000145715	DDG2P_v2.2 panel
RAX	ENSG00000134438	DDG2P_v2.2 panel
RBM10	ENSG00000182872	DDG2P_v2.2 panel
RBM28	ENSG00000106344	DDG2P_v2.2 panel
RBM8A	ENSG00000265241	DDG2P_v2.2 panel
RBPJ	ENSG00000168214	DDG2P_v2.2 panel
RECQL4	ENSG00000160957	DDG2P_v2.2 panel
RELN	ENSG00000189056	DDG2P_v2.2 panel
REN	ENSG00000143839	Lord et. al.
RERE	ENSG00000142599	DDG2P_v2.2 panel
RET	ENSG00000165731	DDG2P_v2.2 panel
RETREG1	ENSG00000154153	DDG2P_v2.2 panel
RFT1	ENSG00000163933	DDG2P_v2.2 panel
RFX6	ENSG00000185002	DDG2P_v2.2 panel
RGS7	ENSG00000182901	DDG2P_v2.2 panel
RHOBTB2	ENSG00000008853	DDG2P_v2.2 panel
RIN2	ENSG00000132669	DDG2P_v2.2 panel
RINT1	ENSG00000135249	DDG2P_v2.2 panel
RIPK4	ENSG00000183421	DDG2P_v2.2 panel
RIT1	ENSG00000143622	DDG2P_v2.2 panel
RLIM	ENSG00000131263	DDG2P_v2.2 panel
RMI1	ENSG00000178966	DDG2P_v2.2 panel
RMND1	ENSG00000155906	DDG2P_v2.2 panel
RMRP	ENSG00000269900	DDG2P_v2.2 panel
RNASEH2A	ENSG00000104889	DDG2P_v2.2 panel
RNASEH2B	ENSG00000136104	DDG2P_v2.2 panel
RNASEH2C	ENSG00000172922	DDG2P_v2.2 panel

RNASET2	ENSG00000026297	DDG2P_v2.2 panel
RNF113A	ENSG00000125352	DDG2P_v2.2 panel
RNF13	ENSG00000082996	DDG2P_v2.2 panel
RNF135	ENSG00000181481	DDG2P_v2.2 panel
RNF168	ENSG00000163961	DDG2P_v2.2 panel
RNU4ATAC	ENSG00000264229	DDG2P_v2.2 panel
ROBO1	ENSG00000169855	Lord et. al.
ROBO3	ENSG00000154134	DDG2P_v2.2 panel
ROBO4	ENSG00000154133	DDG2P_v2.2 panel
ROGDI	ENSG00000067836	DDG2P_v2.2 panel
ROR2	ENSG00000169071	DDG2P_v2.2 panel
RORA	ENSG00000069667	DDG2P_v2.2 panel
RPE65	ENSG00000116745	DDG2P_v2.2 panel
RPGRIP1	ENSG00000092200	DDG2P_v2.2 panel
RPGRIP1L	ENSG00000103494	DDG2P_v2.2 panel
RPL11	ENSG00000142676	DDG2P_v2.2 panel and Lord et. al.
RPL5	ENSG00000122406	Lord et. al.
RPS10	ENSG00000124614	Lord et. al.
RPS17	ENSG00000182774	Lord et. al.
RPS19	ENSG00000105372	DDG2P_v2.2 panel
RPS23	ENSG00000186468	DDG2P_v2.2 panel
RPS26	ENSG00000197728	Lord et. al.
RPS6KA3	ENSG00000177189	DDG2P_v2.2 panel
RRAS	ENSG00000126458	DDG2P_v2.2 panel
RRAS2	ENSG00000133818	DDG2P_v2.2 panel
RRM2B	ENSG00000048392	DDG2P_v2.2 panel
RSPH1	ENSG00000160188	DDG2P_v2.2 panel
RSPH3	ENSG00000130363	DDG2P_v2.2 panel
RSPO2	ENSG00000147655	DDG2P_v2.2 panel
RSPO4	ENSG00000101282	DDG2P_v2.2 panel
RSPRY1	ENSG00000159579	DDG2P_v2.2 panel
RTEL1	ENSG00000258366	DDG2P_v2.2 panel
RTN4IP1	ENSG00000130347	DDG2P_v2.2 panel
RTTN	ENSG00000176225	DDG2P_v2.2 panel
RUBCN	ENSG00000145016	DDG2P_v2.2 panel
RUNX2	ENSG00000124813	DDG2P_v2.2 panel
RYR1	ENSG00000196218	DDG2P_v2.2 panel
RYR3	ENSG00000198838	DDG2P_v2.2 panel
SACS	ENSG00000151835	DDG2P_v2.2 panel
SALL1	ENSG00000103449	DDG2P_v2.2 panel
SALL4	ENSG00000101115	DDG2P_v2.2 panel
SAMD9	ENSG00000205413	DDG2P_v2.2 panel
SAMD9L	ENSG00000177409	DDG2P_v2.2 panel
SAMHD1	ENSG00000101347	DDG2P_v2.2 panel
SATB2	ENSG00000119042	DDG2P_v2.2 panel
SBDS	ENSG00000126524	DDG2P_v2.2 panel
SC5D	ENSG00000109929	DDG2P_v2.2 panel
SCAPER	ENSG00000140386	DDG2P_v2.2 panel
SCARF2	ENSG00000244486	DDG2P_v2.2 panel
SCN11A	ENSG00000168356	DDG2P_v2.2 panel
SCN1A	ENSG00000144285	DDG2P_v2.2 panel
SCN1B	ENSG00000105711	DDG2P_v2.2 panel
SCN2A	ENSG00000136531	DDG2P_v2.2 panel
SCN3A	ENSG00000153253	DDG2P_v2.2 panel
SCN4A	ENSG00000007314	DDG2P_v2.2 panel
SCN8A	ENSG00000196876	DDG2P_v2.2 panel
SCO1	ENSG00000133028	DDG2P_v2.2 panel
SCO2	ENSG00000130489	DDG2P_v2.2 panel
SCRIB	ENSG00000180900	DDG2P_v2.2 panel
SCYL1	ENSG00000142186	DDG2P_v2.2 panel
SDCCAG8	ENSG00000054282	DDG2P_v2.2 panel

SDHA	ENSG00000073578	DDG2P_v2.2 panel
SDHAF1	ENSG00000205138	DDG2P_v2.2 panel
SEC23A	ENSG00000100934	DDG2P_v2.2 panel
SEC23B	ENSG00000101310	DDG2P_v2.2 panel
SEC24D	ENSG00000150961	DDG2P_v2.2 panel
SEC61A1	ENSG00000058262	DDG2P_v2.2 panel
SECISBP2	ENSG00000187742	DDG2P_v2.2 panel
SELENOI	ENSG00000138018	DDG2P_v2.2 panel
SELENON	ENSG00000162430	Lord et. al.
SEPSECS	ENSG00000109618	DDG2P_v2.2 panel
SET	ENSG00000119335	DDG2P_v2.2 panel
SETBP1	ENSG00000152217	DDG2P_v2.2 panel
SETD1A	ENSG00000099381	DDG2P_v2.2 panel
SETD1B	ENSG00000139718	DDG2P_v2.2 panel
SETD2	ENSG00000181555	DDG2P_v2.2 panel
SETD5	ENSG00000168137	DDG2P_v2.2 panel
SF3B4	ENSG00000143368	DDG2P_v2.2 panel
SGCA	ENSG00000108823	Lord et. al.
SGSH	ENSG00000181523	DDG2P_v2.2 panel
SH3BP2	ENSG00000087266	DDG2P_v2.2 panel
SH3PXD2B	ENSG00000174705	DDG2P_v2.2 panel
SHANK1	ENSG00000161681	DDG2P_v2.2 panel
SHANK2	ENSG00000162105	DDG2P_v2.2 panel
SHANK3	ENSG00000251322	DDG2P_v2.2 panel
SHH	ENSG00000164690	DDG2P_v2.2 panel
SHOC2	ENSG00000108061	DDG2P_v2.2 panel
SHOX	ENSG00000185960	DDG2P_v2.2 panel
SHROOM3	ENSG00000138771	DDG2P_v2.2 panel
SIK1	ENSG00000142178	DDG2P_v2.2 panel
SIL1	ENSG00000120725	DDG2P_v2.2 panel
SIM1	ENSG00000112246	DDG2P_v2.2 panel
SIN3A	ENSG00000169375	DDG2P_v2.2 panel
SIX1	ENSG00000126778	DDG2P_v2.2 panel
SIX3	ENSG00000138083	DDG2P_v2.2 panel
SIX5	ENSG00000177045	DDG2P_v2.2 panel
SIX6	ENSG00000184302	DDG2P_v2.2 panel
SKI	ENSG00000157933	DDG2P_v2.2 panel
SKIV2L	ENSG00000204351	DDG2P_v2.2 panel
SLC10A7	ENSG00000120519	DDG2P_v2.2 panel
SLC12A1	ENSG00000074803	Lord et. al.
SLC12A5	ENSG00000124140	DDG2P_v2.2 panel
SLC12A6	ENSG00000140199	DDG2P_v2.2 panel
SLC13A5	ENSG00000141485	DDG2P_v2.2 panel
SLC16A2	ENSG00000147100	DDG2P_v2.2 panel
SLC17A5	ENSG00000119899	DDG2P_v2.2 panel
SLC19A3	ENSG00000135917	DDG2P_v2.2 panel
SLC1A2	ENSG00000110436	DDG2P_v2.2 panel
SLC22A5	ENSG00000197375	DDG2P_v2.2 panel
SLC24A1	ENSG00000074621	DDG2P_v2.2 panel
SLC24A4	ENSG00000140090	DDG2P_v2.2 panel
SLC25A15	ENSG00000102743	DDG2P_v2.2 panel
SLC25A19	ENSG00000125454	DDG2P_v2.2 panel
SLC25A20	ENSG00000178537	DDG2P_v2.2 panel
SLC25A22	ENSG00000177542	DDG2P_v2.2 panel
SLC25A24	ENSG00000085491	DDG2P_v2.2 panel
SLC25A26	ENSG00000144741	DDG2P_v2.2 panel
SLC25A38	ENSG00000144659	DDG2P_v2.2 panel
SLC25A4	ENSG00000151729	DDG2P_v2.2 panel
SLC26A2	ENSG00000155850	DDG2P_v2.2 panel
SLC26A3	ENSG00000091138	Lord et. al.
SLC27A4	ENSG00000167114	DDG2P_v2.2 panel

SLC2A1	ENSG00000117394	DDG2P_v2.2 panel
SLC2A10	ENSG00000197496	DDG2P_v2.2 panel
SLC2A2	ENSG00000163581	DDG2P_v2.2 panel
SLC31A1	ENSG00000136868	DDG2P_v2.2 panel
SLC33A1	ENSG00000169359	DDG2P_v2.2 panel
SLC35A1	ENSG00000164414	DDG2P_v2.2 panel
SLC35A2	ENSG00000102100	DDG2P_v2.2 panel
SLC35C1	ENSG00000181830	DDG2P_v2.2 panel
SLC35D1	ENSG00000116704	DDG2P_v2.2 panel
SLC39A13	ENSG00000165915	DDG2P_v2.2 panel
SLC39A8	ENSG00000138821	DDG2P_v2.2 panel
SLC45A1	ENSG00000162426	DDG2P_v2.2 panel
SLC46A1	ENSG00000076351	DDG2P_v2.2 panel
SLC4A1	ENSG00000004939	DDG2P_v2.2 panel
SLC4A11	ENSG00000088836	DDG2P_v2.2 panel
SLC4A4	ENSG00000080493	DDG2P_v2.2 panel
SLC52A2	ENSG00000185803	DDG2P_v2.2 panel
SLC52A3	ENSG00000101276	DDG2P_v2.2 panel
SLC5A5	ENSG00000105641	DDG2P_v2.2 panel
SLC5A7	ENSG00000115665	DDG2P_v2.2 panel
SLC6A1	ENSG00000157103	DDG2P_v2.2 panel
SLC6A17	ENSG00000197106	DDG2P_v2.2 panel
SLC6A3	ENSG00000142319	DDG2P_v2.2 panel
SLC6A5	ENSG00000165970	DDG2P_v2.2 panel
SLC6A8	ENSG00000130821	DDG2P_v2.2 panel
SLC6A9	ENSG00000196517	DDG2P_v2.2 panel
SLC9A6	ENSG00000198689	DDG2P_v2.2 panel
SLC9A9	ENSG00000181804	DDG2P_v2.2 panel
SLX4	ENSG00000188827	DDG2P_v2.2 panel
SMAD2	ENSG00000175387	DDG2P_v2.2 panel
SMAD3	ENSG00000166949	DDG2P_v2.2 panel
SMAD4	ENSG00000141646	DDG2P_v2.2 panel
SMAD6	ENSG00000137834	DDG2P_v2.2 panel
SMARCA2	ENSG00000080503	DDG2P_v2.2 panel
SMARCA4	ENSG00000127616	DDG2P_v2.2 panel
SMARCAL1	ENSG00000138375	DDG2P_v2.2 panel
SMARCB1	ENSG00000099956	DDG2P_v2.2 panel
SMARCC2	ENSG00000139613	DDG2P_v2.2 panel
SMARCD1	ENSG00000066117	DDG2P_v2.2 panel
SMARCE1	ENSG00000073584	DDG2P_v2.2 panel
SMC1A	ENSG00000072501	DDG2P_v2.2 panel
SMC3	ENSG00000108055	DDG2P_v2.2 panel
SMCHD1	ENSG00000101596	DDG2P_v2.2 panel
SMG9	ENSG00000105771	DDG2P_v2.2 panel
SMN1	ENSG00000172062	Lord et. al.
SMO	ENSG00000128602	DDG2P_v2.2 panel
SMOC1	ENSG00000198732	DDG2P_v2.2 panel
SMOC2	ENSG00000112562	DDG2P_v2.2 panel
SMPD1	ENSG00000166311	DDG2P_v2.2 panel
SMPD4	ENSG00000136699	DDG2P_v2.2 panel
SMS	ENSG00000102172	DDG2P_v2.2 panel
SNAP25	ENSG00000132639	DDG2P_v2.2 panel
SNAP29	ENSG00000099940	DDG2P_v2.2 panel
SNIP1	ENSG00000163877	DDG2P_v2.2 panel
SNORD118	ENSG00000200463	DDG2P_v2.2 panel
SNRPB	ENSG00000125835	DDG2P_v2.2 panel
SNRPE	ENSG00000182004	DDG2P_v2.2 panel
SNX14	ENSG00000135317	DDG2P_v2.2 panel
SNX3	ENSG00000112335	DDG2P_v2.2 panel
SOBP	ENSG00000112320	DDG2P_v2.2 panel
SON	ENSG00000159140	DDG2P_v2.2 panel

SOS1	ENSG00000115904	DDG2P_v2.2 panel
SOST	ENSG00000167941	Lord et. al.
SOX10	ENSG00000100146	DDG2P_v2.2 panel
SOX11	ENSG00000176887	DDG2P_v2.2 panel
SOX17	ENSG00000164736	DDG2P_v2.2 panel
SOX2	ENSG00000181449	DDG2P_v2.2 panel
SOX3	ENSG00000134595	DDG2P_v2.2 panel
SOX4	ENSG00000124766	DDG2P_v2.2 panel
SOX5	ENSG00000134532	DDG2P_v2.2 panel
SOX9	ENSG00000125398	DDG2P_v2.2 panel
SP110	ENSG00000135899	Lord et. al.
SPAG1	ENSG00000104450	DDG2P_v2.2 panel
SPARC	ENSG00000113140	DDG2P_v2.2 panel
SPATA5	ENSG00000145375	DDG2P_v2.2 panel
SPECC1L	ENSG00000100014	DDG2P_v2.2 panel
SPEG	ENSG00000072195	DDG2P_v2.2 panel
SPG11	ENSG00000104133	DDG2P_v2.2 panel
SPR	ENSG00000116096	DDG2P_v2.2 panel
SPRED1	ENSG00000166068	DDG2P_v2.2 panel
SPRTN	ENSG00000010072	DDG2P_v2.2 panel
SPTAN1	ENSG00000197694	DDG2P_v2.2 panel
SPTBN2	ENSG00000173898	DDG2P_v2.2 panel
SPTLC2	ENSG00000100596	DDG2P_v2.2 panel
SRCAP	ENSG00000080603	DDG2P_v2.2 panel
SRD5A2	ENSG00000277893	Lord et. al.
SRD5A3	ENSG00000128039	DDG2P_v2.2 panel
SRGAP3	ENSG00000196220	DDG2P_v2.2 panel
SRP54	ENSG00000100883	DDG2P_v2.2 panel
SRPX2	ENSG00000102359	DDG2P_v2.2 panel
SRY	ENSG00000184895	DDG2P_v2.2 panel
ST14	ENSG00000149418	DDG2P_v2.2 panel
ST3GAL3	ENSG00000126091	DDG2P_v2.2 panel
ST3GAL5	ENSG00000115525	DDG2P_v2.2 panel
STAG1	ENSG00000118007	DDG2P_v2.2 panel
STAG2	ENSG00000101972	DDG2P_v2.2 panel
STAMBP	ENSG00000124356	DDG2P_v2.2 panel
STAR	ENSG00000147465	DDG2P_v2.2 panel
STAT2	ENSG00000170581	DDG2P_v2.2 panel
STAT5B	ENSG00000173757	DDG2P_v2.2 panel
STIL	ENSG00000123473	DDG2P_v2.2 panel
STIM1	ENSG00000167323	DDG2P_v2.2 panel
STN1	ENSG00000107960	DDG2P_v2.2 panel
STRA6	ENSG00000137868	DDG2P_v2.2 panel
STS	ENSG00000101846	DDG2P_v2.2 panel
STT3A	ENSG00000134910	DDG2P_v2.2 panel
STT3B	ENSG00000163527	DDG2P_v2.2 panel
STX1B	ENSG00000099365	DDG2P_v2.2 panel
STXBP1	ENSG00000136854	DDG2P_v2.2 panel
SUCLG1	ENSG00000163541	DDG2P_v2.2 panel
SUFU	ENSG00000107882	DDG2P_v2.2 panel and Lord et. al.
SUMF1	ENSG00000144455	DDG2P_v2.2 panel
SUMO1	ENSG00000116030	DDG2P_v2.2 panel
SURF1	ENSG00000148290	DDG2P_v2.2 panel
SUZ12	ENSG00000178691	DDG2P_v2.2 panel
SYN1	ENSG00000008056	DDG2P_v2.2 panel
SYNE1	ENSG00000131018	DDG2P_v2.2 panel
SYNGAP1	ENSG00000197283	DDG2P_v2.2 panel
SYP	ENSG00000102003	DDG2P_v2.2 panel
SYT1	ENSG00000067715	DDG2P_v2.2 panel
SZT2	ENSG00000198198	DDG2P_v2.2 panel
TAB2	ENSG00000055208	DDG2P_v2.2 panel

TAC3	ENSG00000166863	DDG2P_v2.2 panel
TACO1	ENSG00000136463	DDG2P_v2.2 panel
TACR3	ENSG00000169836	DDG2P_v2.2 panel
TAF1	ENSG00000147133	DDG2P_v2.2 panel
TAF13	ENSG00000197780	DDG2P_v2.2 panel
TAF2	ENSG00000064313	DDG2P_v2.2 panel
TANGO2	ENSG00000183597	DDG2P_v2.2 panel
TAOK1	ENSG00000160551	DDG2P_v2.2 panel
TAPT1	ENSG00000169762	DDG2P_v2.2 panel
TARS	ENSG00000113407	DDG2P_v2.2 panel
TAT	ENSG00000198650	DDG2P_v2.2 panel
TAZ	ENSG00000102125	DDG2P_v2.2 panel
TBC1D20	ENSG00000125875	DDG2P_v2.2 panel and Lord et. al.
TBC1D23	ENSG00000036054	DDG2P_v2.2 panel
TBC1D24	ENSG00000162065	DDG2P_v2.2 panel
TBCD	ENSG00000141556	DDG2P_v2.2 panel
TBCE	ENSG00000116957	DDG2P_v2.2 panel
TBCK	ENSG00000145348	DDG2P_v2.2 panel
TBL1XR1	ENSG00000177565	DDG2P_v2.2 panel
TBR1	ENSG00000136535	DDG2P_v2.2 panel
TBX1	ENSG00000184058	DDG2P_v2.2 panel
TBX15	ENSG00000092607	DDG2P_v2.2 panel
TBX18	ENSG00000112837	DDG2P_v2.2 panel
TBX20	ENSG00000164532	DDG2P_v2.2 panel
TBX22	ENSG00000122145	DDG2P_v2.2 panel
TBX3	ENSG00000135111	DDG2P_v2.2 panel
TBX4	ENSG00000121075	DDG2P_v2.2 panel
TBX5	ENSG00000089225	DDG2P_v2.2 panel
TBX6	ENSG00000149922	Lord et. al.
TBXAS1	ENSG00000059377	DDG2P_v2.2 panel
TCF12	ENSG00000140262	DDG2P_v2.2 panel
TCF20	ENSG00000100207	DDG2P_v2.2 panel
TCF4	ENSG00000196628	DDG2P_v2.2 panel
TCIRG1	ENSG00000110719	Lord et. al.
TCN2	ENSG00000185339	DDG2P_v2.2 panel
TCOF1	ENSG00000070814	DDG2P_v2.2 panel
TCTN1	ENSG00000204852	DDG2P_v2.2 panel and Lord et. al.
TCTN2	ENSG00000168778	DDG2P_v2.2 panel
TCTN3	ENSG00000119977	DDG2P_v2.2 panel
TDRD7	ENSG00000196116	DDG2P_v2.2 panel
TECPR2	ENSG00000196663	DDG2P_v2.2 panel
TEK	ENSG00000120156	DDG2P_v2.2 panel
TELO2	ENSG00000100726	DDG2P_v2.2 panel
TERC	ENSG00000270141	DDG2P_v2.2 panel
TERT	ENSG00000164362	DDG2P_v2.2 panel
TFAP2A	ENSG00000137203	DDG2P_v2.2 panel
TFAP2B	ENSG00000008196	DDG2P_v2.2 panel
TFRC	ENSG00000072274	DDG2P_v2.2 panel
TGDS	ENSG00000088451	DDG2P_v2.2 panel
TGFB1	ENSG00000105329	DDG2P_v2.2 panel
TGFB2	ENSG00000092969	DDG2P_v2.2 panel
TGFB3	ENSG00000119699	DDG2P_v2.2 panel
TGFBR1	ENSG00000106799	DDG2P_v2.2 panel
TGFBR2	ENSG00000163513	DDG2P_v2.2 panel
TGIF1	ENSG00000177426	DDG2P_v2.2 panel and Lord et. al.
TGM1	ENSG00000092295	Lord et. al.
TH	ENSG00000180176	DDG2P_v2.2 panel
THAP1	ENSG00000131931	DDG2P_v2.2 panel
THOC2	ENSG00000125676	DDG2P_v2.2 panel
THOC6	ENSG00000131652	DDG2P_v2.2 panel
THRA	ENSG00000126351	DDG2P_v2.2 panel

TIMM8A	ENSG00000126953	DDG2P_v2.2 panel
TINF2	ENSG00000092330	DDG2P_v2.2 panel
TK2	ENSG00000166548	DDG2P_v2.2 panel
TKT	ENSG00000163931	DDG2P_v2.2 panel
TLK2	ENSG00000146872	DDG2P_v2.2 panel
TLL1	ENSG00000038295	DDG2P_v2.2 panel
TM4SF20	ENSG00000168955	DDG2P_v2.2 panel
TMCO1	ENSG00000143183	DDG2P_v2.2 panel
TMEM114	ENSG00000232258	DDG2P_v2.2 panel
TMEM126B	ENSG00000171204	DDG2P_v2.2 panel
TMEM135	ENSG00000166575	DDG2P_v2.2 panel
TMEM138	ENSG00000149483	Lord et. al.
TMEM165	ENSG00000134851	DDG2P_v2.2 panel
TMEM199	ENSG00000244045	DDG2P_v2.2 panel
TMEM216	ENSG00000187049	DDG2P_v2.2 panel
TMEM231	ENSG00000205084	Lord et. al.
TMEM237	ENSG00000155755	DDG2P_v2.2 panel
TMEM260	ENSG00000070269	DDG2P_v2.2 panel
TMEM5	ENSG00000118600	DDG2P_v2.2 panel
TMEM67	ENSG00000164953	DDG2P_v2.2 panel
TMEM70	ENSG00000175606	DDG2P_v2.2 panel
TMEM94	ENSG00000177728	DDG2P_v2.2 panel
TMPRSS6	ENSG00000187045	DDG2P_v2.2 panel
TMTC3	ENSG00000139324	DDG2P_v2.2 panel
TNFRSF13B	ENSG00000240505	DDG2P_v2.2 panel
TNNI2	ENSG00000130598	Lord et. al.
TNNT1	ENSG00000105048	Lord et. al.
TNXB	ENSG00000168477	Lord et. al.
TOE1	ENSG00000132773	DDG2P_v2.2 panel
TOP3A	ENSG00000177302	DDG2P_v2.2 panel
TP53RK	ENSG00000172315	DDG2P_v2.2 panel
TP63	ENSG00000073282	DDG2P_v2.2 panel
TPM2	ENSG00000198467	DDG2P_v2.2 panel
TPM3	ENSG00000143549	Lord et. al.
TPP1	ENSG00000166340	DDG2P_v2.2 panel
TPRKB	ENSG00000144034	DDG2P_v2.2 panel
TRAF7	ENSG00000131653	DDG2P_v2.2 panel
TRAIP	ENSG00000183763	DDG2P_v2.2 panel
TRAPPC11	ENSG00000168538	DDG2P_v2.2 panel
TRAPPC12	ENSG00000171853	DDG2P_v2.2 panel
TRAPPC2	ENSG00000196459	DDG2P_v2.2 panel
TRAPPC9	ENSG00000167632	DDG2P_v2.2 panel
TREX1	ENSG00000213689	DDG2P_v2.2 panel
TRIM32	ENSG00000119401	DDG2P_v2.2 panel
TRIM37	ENSG00000108395	DDG2P_v2.2 panel
TRIO	ENSG00000038382	DDG2P_v2.2 panel
TRIP11	ENSG00000100815	DDG2P_v2.2 panel
TRIP12	ENSG00000153827	DDG2P_v2.2 panel
TRIP13	ENSG00000071539	DDG2P_v2.2 panel
TRIP4	ENSG00000103671	DDG2P_v2.2 panel
TRIT1	ENSG00000043514	DDG2P_v2.2 panel
TRMT1	ENSG00000104907	DDG2P_v2.2 panel
TRMT10C	ENSG00000174173	DDG2P_v2.2 panel
TRPM1	ENSG00000134160	DDG2P_v2.2 panel
TRPS1	ENSG00000104447	DDG2P_v2.2 panel
TRPV3	ENSG00000167723	DDG2P_v2.2 panel
TRPV4	ENSG00000111199	DDG2P_v2.2 panel
TRPV6	ENSG00000165125	DDG2P_v2.2 panel
TRRAP	ENSG00000196367	DDG2P_v2.2 panel
TSC1	ENSG00000165699	DDG2P_v2.2 panel
TSC2	ENSG00000103197	DDG2P_v2.2 panel

TSEN15	ENSG00000198860	DDG2P_v2.2 panel
TSEN2	ENSG00000154743	DDG2P_v2.2 panel
TSEN34	ENSG00000170892	DDG2P_v2.2 panel
TSEN54	ENSG00000182173	DDG2P_v2.2 panel
TSHB	ENSG00000134200	DDG2P_v2.2 panel
TSHR	ENSG00000165409	DDG2P_v2.2 panel
TSHZ1	ENSG00000179981	DDG2P_v2.2 panel
TSPAN7	ENSG00000156298	DDG2P_v2.2 panel
TTC19	ENSG00000011295	DDG2P_v2.2 panel
TTC21B	ENSG00000123607	Lord et. al.
TTC25	ENSG00000204815	DDG2P_v2.2 panel
TTC37	ENSG00000198677	DDG2P_v2.2 panel
TTC7A	ENSG00000068724	DDG2P_v2.2 panel
TTC8	ENSG00000165533	DDG2P_v2.2 panel
TTI2	ENSG00000129696	DDG2P_v2.2 panel
TTN	ENSG00000155657	DDG2P_v2.2 panel
TUBA1A	ENSG00000167552	DDG2P_v2.2 panel
TUBA8	ENSG00000183785	DDG2P_v2.2 panel
TUBB	ENSG00000196230	DDG2P_v2.2 panel
TUBB2A	ENSG00000137267	DDG2P_v2.2 panel
TUBB2B	ENSG00000137285	DDG2P_v2.2 panel
TUBB3	ENSG00000258947	DDG2P_v2.2 panel
TUBB4A	ENSG00000104833	DDG2P_v2.2 panel
TUBG1	ENSG00000131462	DDG2P_v2.2 panel
TUBGCP4	ENSG00000137822	DDG2P_v2.2 panel
TUBGCP6	ENSG00000128159	DDG2P_v2.2 panel
TUFM	ENSG00000178952	DDG2P_v2.2 panel
TUSC3	ENSG00000104723	DDG2P_v2.2 panel
TWIST1	ENSG00000122691	DDG2P_v2.2 panel
TWIST2	ENSG00000233608	DDG2P_v2.2 panel
TXNL4A	ENSG00000141759	DDG2P_v2.2 panel
TYR	ENSG00000077498	DDG2P_v2.2 panel
TYRP1	ENSG00000107165	DDG2P_v2.2 panel
UBA1	ENSG00000130985	Lord et. al.
UBA5	ENSG00000081307	DDG2P_v2.2 panel
UBE2A	ENSG00000077721	DDG2P_v2.2 panel
UBE2T	ENSG00000077152	DDG2P_v2.2 panel
UBE3A	ENSG00000114062	DDG2P_v2.2 panel
UBE3B	ENSG00000151148	DDG2P_v2.2 panel
UBR1	ENSG00000159459	DDG2P_v2.2 panel
UBR7	ENSG00000012963	DDG2P_v2.2 panel
UBTF	ENSG00000108312	DDG2P_v2.2 panel
UFC1	ENSG00000143222	DDG2P_v2.2 panel
UFM1	ENSG00000120686	DDG2P_v2.2 panel
UGT1A1	ENSG00000241635	DDG2P_v2.2 panel
UMPS	ENSG00000114491	DDG2P_v2.2 panel
UNC80	ENSG00000144406	DDG2P_v2.2 panel
UPF3B	ENSG00000125351	DDG2P_v2.2 panel
UQCRB	ENSG00000156467	DDG2P_v2.2 panel
UQCRQ	ENSG00000164405	DDG2P_v2.2 panel
UROC1	ENSG00000159650	DDG2P_v2.2 panel
UROS	ENSG00000188690	DDG2P_v2.2 panel
USB1	ENSG00000103005	DDG2P_v2.2 panel
USP18	ENSG00000184979	DDG2P_v2.2 panel
USP27X	ENSG00000273820	DDG2P_v2.2 panel
USP7	ENSG00000187555	DDG2P_v2.2 panel
USP9X	ENSG00000124486	DDG2P_v2.2 panel
UTP4	ENSG00000141076	DDG2P_v2.2 panel
UVSSA	ENSG00000163945	DDG2P_v2.2 panel
VAC14	ENSG00000103043	DDG2P_v2.2 panel
VAMP2	ENSG00000220205	DDG2P_v2.2 panel

VANGL1	ENSG00000173218	DDG2P_v2.2 panel
VDR	ENSG00000111424	DDG2P_v2.2 panel
VIP	ENSG00000146469	DDG2P_v2.2 panel
VIPAS39	ENSG00000151445	DDG2P_v2.2 panel
VLDLR	ENSG00000147852	DDG2P_v2.2 panel
VPS13B	ENSG00000132549	DDG2P_v2.2 panel
VPS33B	ENSG00000184056	DDG2P_v2.2 panel
VPS53	ENSG00000141252	DDG2P_v2.2 panel
VRK1	ENSG00000100749	DDG2P_v2.2 panel
VSX2	ENSG00000119614	DDG2P_v2.2 panel
WAC	ENSG00000095787	DDG2P_v2.2 panel
WASF1	ENSG00000112290	DDG2P_v2.2 panel
WDFY3	ENSG00000163625	DDG2P_v2.2 panel
WDPCP	ENSG00000143951	DDG2P_v2.2 panel
WDR11	ENSG00000120008	DDG2P_v2.2 panel
WDR19	ENSG00000157796	DDG2P_v2.2 panel
WDR26	ENSG00000162923	DDG2P_v2.2 panel
WDR34	ENSG00000119333	DDG2P_v2.2 panel
WDR35	ENSG00000118965	DDG2P_v2.2 panel
WDR37	ENSG00000047056	DDG2P_v2.2 panel
WDR4	ENSG00000160193	DDG2P_v2.2 panel
WDR45	ENSG00000196998	DDG2P_v2.2 panel
WDR45B	ENSG00000141580	DDG2P_v2.2 panel
WDR60	ENSG00000126870	DDG2P_v2.2 panel
WDR62	ENSG00000075702	DDG2P_v2.2 panel
WDR73	ENSG00000177082	DDG2P_v2.2 panel
WDR81	ENSG00000167716	DDG2P_v2.2 panel
WNT1	ENSG00000125084	DDG2P_v2.2 panel
WNT10B	ENSG00000169884	DDG2P_v2.2 panel
WNT3	ENSG00000108379	DDG2P_v2.2 panel
WNT4	ENSG00000162552	DDG2P_v2.2 panel
WNT5A	ENSG00000114251	DDG2P_v2.2 panel
WNT7A	ENSG00000154764	DDG2P_v2.2 panel
WRAP53	ENSG00000141499	DDG2P_v2.2 panel
WT1	ENSG00000184937	DDG2P_v2.2 panel
WWOX	ENSG00000186153	DDG2P_v2.2 panel
XPA	ENSG00000136936	DDG2P_v2.2 panel
XPC	ENSG00000154767	DDG2P_v2.2 panel
XPNPEP3	ENSG00000196236	DDG2P_v2.2 panel
XRCC4	ENSG00000152422	DDG2P_v2.2 panel
XYLT1	ENSG00000103489	DDG2P_v2.2 panel
XYLT2	ENSG00000015532	DDG2P_v2.2 panel
YAP1	ENSG00000137693	DDG2P_v2.2 panel
YWHAG	ENSG00000170027	DDG2P_v2.2 panel
YY1	ENSG00000100811	DDG2P_v2.2 panel
ZBTB16	ENSG00000109906	DDG2P_v2.2 panel
ZBTB18	ENSG00000179456	DDG2P_v2.2 panel
ZBTB20	ENSG00000181722	DDG2P_v2.2 panel
ZBTB40	ENSG00000184677	DDG2P_v2.2 panel
ZC4H2	ENSG00000126970	DDG2P_v2.2 panel
ZCCHC8	ENSG00000033030	DDG2P_v2.2 panel
ZDHHC15	ENSG00000102383	DDG2P_v2.2 panel
ZDHHC9	ENSG00000188706	DDG2P_v2.2 panel
ZEB1	ENSG00000148516	DDG2P_v2.2 panel
ZEB2	ENSG00000169554	DDG2P_v2.2 panel
ZFP57	ENSG00000204644	DDG2P_v2.2 panel
ZFPM2	ENSG00000169946	DDG2P_v2.2 panel
ZFYVE26	ENSG00000072121	DDG2P_v2.2 panel
ZIC1	ENSG00000152977	DDG2P_v2.2 panel
ZIC2	ENSG00000043355	DDG2P_v2.2 panel
ZIC3	ENSG00000156925	DDG2P_v2.2 panel

ZMIZ1	ENSG00000108175	DDG2P_v2.2 panel
ZMPSTE24	ENSG00000084073	DDG2P_v2.2 panel
ZMYM6	ENSG00000163867	DDG2P_v2.2 panel
ZMYND10	ENSG00000004838	DDG2P_v2.2 panel
ZMYND11	ENSG00000015171	DDG2P_v2.2 panel
ZNF423	ENSG00000102935	Lord et. al.
ZNF462	ENSG00000148143	DDG2P_v2.2 panel
ZNF526	ENSG00000167625	DDG2P_v2.2 panel
ZNF599	ENSG00000153896	DDG2P_v2.2 panel
ZNF711	ENSG00000147180	DDG2P_v2.2 panel
ZNF713	ENSG00000178665	DDG2P_v2.2 panel
ZNF750	ENSG00000141579	DDG2P_v2.2 panel
ZSWIM6	ENSG00000130449	DDG2P_v2.2 panel

Supplementary Table 2. Primer sequences used for the validation of splicing variants by RT-PCR.

Case	Variants to validate	Primer	Sequence (5'-3')
1	*CHD7* :c.7164+1G>A	CHD7-Ex32-F	GGAGCTGTCTCTAGAGGGAAGA
		CHD7-Ex34-R	CAATGACGTCCGTTTGTGGC
2	*MYRF* :c.2014-1G>A	MYRF-Ex14-F	GTGTCATCGCTCAGGAGG
		MYRF-Ex17-R	CTGAAGGCCATGACCACCA
3	*COL1A2* :c.2133+5G>A	COL1A2-Ex32-F	CCTGGTATTGTAGGCACTGATTT
		COL1A2-Ex37-F	CAGCACCCTGTTGGAAATAAAC

Supplementary Table 3. A list of 81 out of the 2,020 curated genes that were well-expressed (TPM values ≥ 10) in AF cells but not expressed (TPM values < 1) or lowly expressed (1 ≤ TMP < 10) in the whole blood and skin fibroblasts.

Gene Name	Ensembl ID(GRch37)	Control Amniotic Fluid Cells	Fibroblast_GTEx	Whole blood_GTEx
ADAMTS9	ENSG00000163638	Well expressed	Low expressed	Not expressed
ADGRG1	ENSG00000205336	Well expressed	Not expressed	Low expressed
ADGRG6	ENSG00000112414	Well expressed	Low expressed	Not expressed
ALDH1A3	ENSG00000184254	Well expressed	Low expressed	Not expressed
B9D1	ENSG00000108641	Well expressed	Low expressed	Not expressed
BMP2	ENSG00000125845	Well expressed	Not expressed	Not expressed
CASK	ENSG00000147044	Well expressed	Low expressed	Not expressed
CBS	ENSG00000160200	Well expressed	Low expressed	Not expressed
CCND2	ENSG00000118971	Well expressed	Low expressed	Low expressed
CDH3	ENSG00000062038	Well expressed	Not expressed	Not expressed
CDKN1C	ENSG00000129757	Well expressed	Low expressed	Low expressed
CHRDL1	ENSG00000101938	Well expressed	Low expressed	Not expressed
CIB2	ENSG00000136425	Well expressed	Low expressed	Not expressed
DACT1	ENSG00000165617	Well expressed	Low expressed	Not expressed
DCDC2	ENSG00000146038	Well expressed	Not expressed	Not expressed
DDX58	ENSG00000107201	Well expressed	Low expressed	Low expressed
DNMT3B	ENSG00000088305	Well expressed	Low expressed	Not expressed
DSP	ENSG00000096696	Well expressed	Low expressed	Not expressed
EDN1	ENSG00000078401	Well expressed	Low expressed	Not expressed
EEF1A2	ENSG00000101210	Well expressed	Not expressed	Not expressed
EHMT1	ENSG00000181090	Well expressed	Low expressed	Low expressed
ERCC5	ENSG00000134899	Well expressed	Low expressed	Not expressed
FANCF	ENSG00000183161	Well expressed	Low expressed	Low expressed
FOXC1	ENSG00000054598	Well expressed	Low expressed	Not expressed
GATA6	ENSG00000141448	Well expressed	Not expressed	Not expressed
GCSH	ENSG00000140905	Well expressed	Low expressed	Not expressed
GDF5	ENSG00000125965	Well expressed	Low expressed	Not expressed
GLDC	ENSG00000178445	Well expressed	Not expressed	Not expressed
GLIS3	ENSG00000107249	Well expressed	Low expressed	Not expressed
GPC4	ENSG00000076716	Well expressed	Low expressed	Not expressed
H4C11	ENSG00000197238	Well expressed	Not expressed	Not expressed
HNF1B	ENSG00000275410	Well expressed	Not expressed	Not expressed
IKBKG	ENSG00000269335	Well expressed	Low expressed	Low expressed
IL11	ENSG00000095752	Well expressed	Low expressed	Not expressed
IRF6	ENSG00000117595	Well expressed	Not expressed	Not expressed
ITGA6	ENSG00000091409	Well expressed	Low expressed	Low expressed
ITGB4	ENSG00000132470	Well expressed	Not expressed	Not expressed
KCNMA1	ENSG00000156113	Well expressed	Low expressed	Not expressed
KDM6A	ENSG00000147050	Well expressed	Low expressed	Low expressed
L1CAM	ENSG00000198910	Well expressed	Not expressed	Not expressed
MATN3	ENSG00000132031	Well expressed	Low expressed	Not expressed
MID1	ENSG00000101871	Well expressed	Low expressed	Not expressed
MITF	ENSG00000187098	Well expressed	Low expressed	Not expressed
MMACHC	ENSG00000132763	Well expressed	Low expressed	Not expressed
MT-TP	ENSG00000210196	Well expressed	Not expressed	Not expressed
MYO5B	ENSG00000167306	Well expressed	Not expressed	Not expressed
MYRF	ENSG00000124920	Well expressed	Low expressed	Not expressed
NEDD4L	ENSG00000049759	Well expressed	Low expressed	Low expressed
NOG	ENSG00000183691	Well expressed	Low expressed	Not expressed
ODAPH	ENSG00000174792	Well expressed	Not expressed	Not expressed
PAX2	ENSG00000075891	Well expressed	Not expressed	Not expressed
PAX8	ENSG00000125618	Well expressed	Low expressed	Not expressed
PEX26	ENSG00000215193	Well expressed	Low expressed	Low expressed
PHC1	ENSG00000111752	Well expressed	Low expressed	Low expressed
POMK	ENSG00000185900	Well expressed	Low expressed	Not expressed
PPM1D	ENSG00000170836	Well expressed	Low expressed	Low expressed
RIPK4	ENSG00000183421	Well expressed	Not expressed	Not expressed
ROBO3	ENSG00000154134	Well expressed	Low expressed	Not expressed
RUBCN	ENSG00000145016	Well expressed	Low expressed	Low expressed
SALL1	ENSG00000103449	Well expressed	Low expressed	Not expressed
SELENOI	ENSG00000138018	Well expressed	Low expressed	Not expressed
SHROOM3	ENSG00000138771	Well expressed	Low expressed	Not expressed
SIK1	ENSG00000142178	Well expressed	Not expressed	Not expressed
SLC25A4	ENSG00000151729	Well expressed	Low expressed	Not expressed
SLC6A9	ENSG00000196517	Well expressed	Low expressed	Low expressed
SMN1	ENSG00000172062	Well expressed	Low expressed	Not expressed
SMO	ENSG00000128602	Well expressed	Low expressed	Not expressed
SOX11	ENSG00000176887	Well expressed	Not expressed	Not expressed
SOX4	ENSG00000124766	Well expressed	Low expressed	Not expressed
SOX9	ENSG00000125398	Well expressed	Not expressed	Not expressed
SPTBN2	ENSG00000173898	Well expressed	Not expressed	Not expressed
ST14	ENSG00000149418	Well expressed	Not expressed	Low expressed
TDRD7	ENSG00000196116	Well expressed	Low expressed	Low expressed
TGFB2	ENSG00000092969	Well expressed	Low expressed	Not expressed
TNNT1	ENSG00000105048	Well expressed	Low expressed	Low expressed

Supplementary Table 4. List of 247 of the 2,020 curated genes not expressed (TPM values < 1) or lowly expressed (1 ≤ TMP < 10) in AF cells but well-expressed (TPM values ≥ 10) in the whole blood and skin fibroblasts.

Gene Name	Ensembl ID(GRch37)	Control Amniotic Fluid Cells	Fibroblast_GTEx	Whole blood_GTEx
AASS	ENSG00000008311	Low expressed	Well expressed	Not expressed
ABCB6	ENSG00000115657	Low expressed	Well expressed	Low expressed
ABCC9	ENSG00000069431	Not expressed	Well expressed	Not expressed
ABHD5	ENSG00000011198	Low expressed	Well expressed	Well expressed
ACP5	ENSG00000102575	Low expressed	Low expressed	Well expressed
AGK	ENSG00000006530	Low expressed	Well expressed	Low expressed
ALDH3A2	ENSG00000072210	Low expressed	Well expressed	Low expressed
ALG13	ENSG00000101901	Low expressed	Well expressed	Low expressed
ALPL	ENSG00000162551	Not expressed	Low expressed	Well expressed
ANKH	ENSG00000154122	Low expressed	Well expressed	Low expressed
AP4B1	ENSG00000134262	Low expressed	Well expressed	Low expressed
AP4E1	ENSG00000081014	Low expressed	Well expressed	Low expressed
AR	ENSG00000169083	Not expressed	Well expressed	Not expressed
ARG1	ENSG00000118520	Not expressed	Not expressed	Well expressed
ARHGEF6	ENSG00000129675	Not expressed	Well expressed	Well expressed
ARL13B	ENSG00000169379	Low expressed	Well expressed	Not expressed
ARL14EP	ENSG00000152219	Low expressed	Well expressed	Low expressed
ASPM	ENSG00000066279	Low expressed	Well expressed	Not expressed
ATM	ENSG00000149311	Low expressed	Well expressed	Low expressed
ATR	ENSG00000175054	Low expressed	Well expressed	Low expressed
AUH	ENSG00000148090	Low expressed	Well expressed	Low expressed
B3GLCT	ENSG00000187676	Low expressed	Well expressed	Not expressed
BBS2	ENSG00000125124	Low expressed	Well expressed	Low expressed
BBS5	ENSG00000163093	Low expressed	Well expressed	Not expressed
BMPER	ENSG00000164619	Not expressed	Well expressed	Not expressed
BNC2	ENSG00000173068	Low expressed	Well expressed	Not expressed
BRAF	ENSG00000157764	Low expressed	Well expressed	Low expressed
BUB1B	ENSG00000156970	Low expressed	Well expressed	Not expressed
CA2	ENSG00000104267	Low expressed	Not expressed	Well expressed
CC2D2A	ENSG00000048342	Low expressed	Well expressed	Not expressed
CCBE1	ENSG00000183287	Low expressed	Well expressed	Not expressed
CCDC88A	ENSG00000115355	Low expressed	Well expressed	Low expressed
CCDC88C	ENSG00000015133	Low expressed	Not expressed	Well expressed
CDAN1	ENSG00000140326	Low expressed	Well expressed	Low expressed
CDC45	ENSG00000093009	Low expressed	Well expressed	Not expressed
CDK13	ENSG00000065883	Low expressed	Well expressed	Low expressed
CDT1	ENSG00000167513	Low expressed	Well expressed	Low expressed
CENPF	ENSG00000117724	Low expressed	Well expressed	Not expressed
CEP164	ENSG00000110274	Low expressed	Well expressed	Low expressed
CEP290	ENSG00000198707	Low expressed	Well expressed	Not expressed
CEP63	ENSG00000182923	Low expressed	Well expressed	Low expressed
CERT1	ENSG00000113163	Low expressed	Well expressed	Well expressed
CFAP410	ENSG00000160226	Low expressed	Well expressed	Low expressed
CHM	ENSG00000188419	Low expressed	Well expressed	Not expressed
CIT	ENSG00000122966	Low expressed	Well expressed	Not expressed
CLN3	ENSG00000188603	Low expressed	Well expressed	Well expressed
CLN6	ENSG00000128973	Low expressed	Well expressed	Well expressed
CLPB	ENSG00000162129	Low expressed	Well expressed	Low expressed
COL11A1	ENSG00000060718	Low expressed	Well expressed	Not expressed
CPS1	ENSG00000021826	Low expressed	Well expressed	Not expressed
CPT2	ENSG00000157184	Low expressed	Well expressed	Low expressed
CRLF1	ENSG00000006016	Low expressed	Well expressed	Not expressed
CSF1R	ENSG00000182578	Not expressed	Not expressed	Well expressed
CSTA	ENSG00000121552	Not expressed	Well expressed	Well expressed
CTC1	ENSG00000178971	Low expressed	Low expressed	Well expressed
CTSK	ENSG00000143387	Low expressed	Well expressed	Low expressed
CYP2U1	ENSG00000155016	Low expressed	Well expressed	Not expressed
D2HGDH	ENSG00000180902	Low expressed	Well expressed	Low expressed
DCAF17	ENSG00000115827	Low expressed	Well expressed	Low expressed
DDR2	ENSG00000162733	Low expressed	Well expressed	Not expressed
DLG4	ENSG00000132535	Low expressed	Well expressed	Low expressed
DMPK	ENSG00000104936	Low expressed	Well expressed	Low expressed
DOCK8	ENSG00000107099	Not expressed	Not expressed	Well expressed
DYNC2H1	ENSG00000187240	Low expressed	Well expressed	Not expressed
DYNC2I1	ENSG00000126870	Low expressed	Well expressed	Low expressed
DYRK1A	ENSG00000157540	Low expressed	Well expressed	Low expressed
EED	ENSG00000074266	Low expressed	Well expressed	Low expressed
ENTPD1	ENSG00000138185	Not expressed	Low expressed	Well expressed
ERCC8	ENSG00000049167	Low expressed	Well expressed	Not expressed
ERMARD	ENSG00000130023	Low expressed	Well expressed	Low expressed
FAM111A	ENSG00000166801	Low expressed	Well expressed	Low expressed
FANCE	ENSG00000112039	Low expressed	Well expressed	Low expressed
FANCG	ENSG00000221829	Low expressed	Well expressed	Low expressed
FANCI	ENSG00000140525	Low expressed	Well expressed	Low expressed
FARS2	ENSG00000145982	Low expressed	Well expressed	Low expressed
FAT4	ENSG00000196159	Low expressed	Well expressed	Not expressed
FBLN1	ENSG00000077942	Low expressed	Well expressed	Not expressed
FBLN5	ENSG00000140092	Low expressed	Well expressed	Not expressed
FBP1	ENSG00000165140	Not expressed	Not expressed	Well expressed
FBXL4	ENSG00000112234	Low expressed	Well expressed	Low expressed
FKTN	ENSG00000106692	Low expressed	Well expressed	Not expressed
FMN2	ENSG00000155816	Not expressed	Well expressed	Not expressed
FMR1	ENSG00000102081	Low expressed	Well expressed	Low expressed
FOXF1	ENSG00000103241	Not expressed	Well expressed	Not expressed
FRAS1	ENSG00000138759	Low expressed	Well expressed	Not expressed
GALNS	ENSG00000141012	Low expressed	Well expressed	Well expressed
GK	ENSG00000198814	Low expressed	Well expressed	Well expressed
GLI3	ENSG00000106571	Low expressed	Well expressed	Not expressed
GLMN	ENSG00000174842	Low expressed	Well expressed	Not expressed
GNAI1	ENSG00000127955	Low expressed	Well expressed	Not expressed
GORAB	ENSG00000120370	Low expressed	Well expressed	Not expressed
GPSM2	ENSG00000121957	Low expressed	Well expressed	Low expressed
GTF2H5	ENSG00000272047	Low expressed	Well expressed	Not expressed
GTPBP3	ENSG00000130299	Low expressed	Well expressed	Low expressed
HACE1	ENSG00000085382	Low expressed	Well expressed	Not expressed
HADH	ENSG00000138796	Low expressed	Well expressed	Low expressed
HDAC4	ENSG00000068024	Low expressed	Low expressed	Well expressed
HIBCH	ENSG00000198130	Low expressed	Well expressed	Low expressed
IDUA	ENSG00000127415	Low expressed	Well expressed	Low expressed
IFT122	ENSG00000163913	Low expressed	Well expressed	Low expressed
IFT172	ENSG00000138002	Low expressed	Well expressed	Not expressed
IFT80	ENSG00000068885	Low expressed	Well expressed	Not expressed
IGF2	ENSG00000167244	Low expressed	Well expressed	Not expressed

IL11RA	ENSG00000137070	Low expressed	Well expressed	Low expressed
INPP5E	ENSG00000148384	Low expressed	Well expressed	Low expressed
INVS	ENSG00000119509	Low expressed	Well expressed	Low expressed
JAK3	ENSG00000105639	Low expressed	Low expressed	Well expressed
KANK1	ENSG00000107104	Low expressed	Well expressed	Not expressed
KANSL1	ENSG00000120071	Low expressed	Well expressed	Low expressed
KAT6A	ENSG00000083168	Low expressed	Well expressed	Well expressed
KCNJ8	ENSG00000121361	Low expressed	Well expressed	Not expressed
KCNQ1	ENSG00000053918	Not expressed	Not expressed	Well expressed
KIAA1109	ENSG00000138688	Low expressed	Well expressed	Low expressed
KIF11	ENSG00000138160	Low expressed	Well expressed	Not expressed
KIF4A	ENSG00000090889	Low expressed	Well expressed	Not expressed
KIF7	ENSG00000166813	Low expressed	Well expressed	Not expressed
KLF1	ENSG00000105610	Not expressed	Not expressed	Well expressed
KMT2C	ENSG00000055609	Low expressed	Well expressed	Low expressed
KMT5B	ENSG00000110066	Low expressed	Well expressed	Low expressed
KRIT1	ENSG00000001631	Low expressed	Well expressed	Low expressed
LAMA1	ENSG00000101680	Low expressed	Well expressed	Not expressed
LAMA2	ENSG00000196569	Not expressed	Well expressed	Not expressed
LMBRD1	ENSG00000168216	Low expressed	Well expressed	Well expressed
LNPK	ENSG00000144320	Low expressed	Well expressed	Low expressed
LYST	ENSG00000143669	Low expressed	Low expressed	Well expressed
LZTFL1	ENSG00000163818	Low expressed	Well expressed	Not expressed
MAFB	ENSG00000204103	Not expressed	Low expressed	Well expressed
MAMLD1	ENSG00000013619	Low expressed	Well expressed	Not expressed
MANBA	ENSG00000109323	Low expressed	Well expressed	Well expressed
MAOA	ENSG00000189221	Low expressed	Well expressed	Low expressed
MASP1	ENSG00000127241	Not expressed	Well expressed	Not expressed
MBTPS2	ENSG00000012174	Low expressed	Well expressed	Not expressed
MCEE	ENSG00000124370	Low expressed	Well expressed	Low expressed
MED13L	ENSG00000123066	Low expressed	Well expressed	Low expressed
MED17	ENSG00000042429	Low expressed	Well expressed	Low expressed
MGP	ENSG00000111341	Not expressed	Well expressed	Low expressed
MKS1	ENSG00000011143	Low expressed	Well expressed	Low expressed
MPLKIP	ENSG00000168303	Low expressed	Well expressed	Low expressed
MRE11	ENSG00000020922	Low expressed	Well expressed	Low expressed
MSH2	ENSG00000095002	Low expressed	Well expressed	Low expressed
MSL3	ENSG00000005302	Low expressed	Well expressed	Well expressed
MSX1	ENSG00000163132	Not expressed	Well expressed	Not expressed
MTF1	ENSG00000188786	Low expressed	Well expressed	Well expressed
NADK2	ENSG00000152620	Low expressed	Well expressed	Low expressed
NAGS	ENSG00000161653	Low expressed	Well expressed	Not expressed
NCAPD3	ENSG00000151503	Low expressed	Well expressed	Low expressed
NCAPG2	ENSG00000146918	Low expressed	Well expressed	Low expressed
NCAPH	ENSG00000121152	Low expressed	Well expressed	Not expressed
NEK1	ENSG00000137601	Low expressed	Well expressed	Not expressed
NGLY1	ENSG00000151092	Low expressed	Well expressed	Well expressed
NMNAT1	ENSG00000173614	Low expressed	Well expressed	Low expressed
NOTCH3	ENSG00000074181	Low expressed	Well expressed	Not expressed
NPHP3	ENSG00000113971	Low expressed	Well expressed	Low expressed
NPR3	ENSG00000113389	Not expressed	Well expressed	Not expressed
NR2F1	ENSG00000175745	Low expressed	Well expressed	Not expressed
OFD1	ENSG00000046651	Low expressed	Well expressed	Low expressed
ORC6	ENSG00000091651	Low expressed	Well expressed	Not expressed
OTULIN	ENSG00000154124	Low expressed	Well expressed	Low expressed
PALB2	ENSG00000083093	Low expressed	Well expressed	Not expressed
PC	ENSG00000173599	Low expressed	Well expressed	Low expressed
PCNT	ENSG00000160299	Low expressed	Well expressed	Low expressed
PEX1	ENSG00000127980	Low expressed	Well expressed	Low expressed
PEX12	ENSG00000108733	Low expressed	Well expressed	Not expressed
PHF21A	ENSG00000135365	Low expressed	Well expressed	Well expressed
PHIP	ENSG00000146247	Low expressed	Well expressed	Low expressed
PIGB	ENSG00000069943	Low expressed	Well expressed	Low expressed
PIGG	ENSG00000174227	Low expressed	Well expressed	Low expressed
PIGM	ENSG00000143315	Low expressed	Well expressed	Low expressed
PIGV	ENSG00000060642	Low expressed	Well expressed	Low expressed
PIK3CA	ENSG00000121879	Low expressed	Well expressed	Low expressed
PIK3R1	ENSG00000145675	Low expressed	Well expressed	Low expressed
PLCG2	ENSG00000197943	Low expressed	Not expressed	Well expressed
POC1A	ENSG00000164087	Low expressed	Well expressed	Low expressed
POT1	ENSG00000128513	Low expressed	Well expressed	Low expressed
PRKD1	ENSG00000184304	Low expressed	Well expressed	Not expressed
PRMT7	ENSG00000132600	Low expressed	Well expressed	Low expressed
PRMT9	ENSG00000164169	Low expressed	Well expressed	Low expressed
PROK2	ENSG00000163421	Not expressed	Not expressed	Well expressed
PRRX1	ENSG00000116132	Not expressed	Well expressed	Not expressed
PRSS12	ENSG00000164099	Low expressed	Well expressed	Not expressed
PURA	ENSG00000185129	Low expressed	Well expressed	Not expressed
RECQL4	ENSG00000160957	Low expressed	Well expressed	Low expressed
RHOBTB2	ENSG00000008853	Low expressed	Well expressed	Low expressed
RMI1	ENSG00000178966	Low expressed	Well expressed	Low expressed
RNF135	ENSG00000181481	Low expressed	Well expressed	Well expressed
RNF168	ENSG00000163961	Low expressed	Well expressed	Low expressed
SAMD9	ENSG00000205413	Low expressed	Well expressed	Low expressed
SECISBP2	ENSG00000187742	Low expressed	Well expressed	Low expressed
SIX1	ENSG00000126778	Low expressed	Well expressed	Not expressed
SLC12A6	ENSG00000140199	Low expressed	Low expressed	Well expressed
SLC2A10	ENSG00000197496	Low expressed	Well expressed	Not expressed
SLC35D1	ENSG00000116704	Low expressed	Well expressed	Low expressed
SLC39A8	ENSG00000138821	Low expressed	Well expressed	Low expressed
SLC4A1	ENSG00000004939	Not expressed	Not expressed	Well expressed
SLC9A6	ENSG00000198689	Low expressed	Well expressed	Low expressed
SMARCA2	ENSG00000080503	Low expressed	Well expressed	Low expressed
SNIP1	ENSG00000163877	Low expressed	Well expressed	Low expressed
SP110	ENSG00000135899	Low expressed	Well expressed	Well expressed
SRD5A3	ENSG00000128039	Low expressed	Well expressed	Low expressed
SRPX2	ENSG00000102359	Low expressed	Well expressed	Not expressed
ST3GAL3	ENSG00000126091	Low expressed	Well expressed	Low expressed
STS	ENSG00000101846	Low expressed	Well expressed	Low expressed
SYNGAP1	ENSG00000197283	Low expressed	Well expressed	Low expressed
TAF1	ENSG00000147133	Low expressed	Well expressed	Low expressed
TAZ	ENSG00000102125	Low expressed	Well expressed	Well expressed
TBCE	ENSG00000284770	Low expressed	Well expressed	Low expressed
TBCK	ENSG00000145348	Low expressed	Well expressed	Not expressed
TBX15	ENSG00000092607	Not expressed	Well expressed	Not expressed

TBX18	ENSG00000112837	Low expressed	Well expressed	Not expressed
TBX3	ENSG00000135111	Low expressed	Well expressed	Not expressed
TBXAS1	ENSG00000059377	Low expressed	Low expressed	Well expressed
TCF4	ENSG00000196628	Low expressed	Well expressed	Not expressed
TCTN1	ENSG00000204852	Low expressed	Well expressed	Not expressed
TCTN2	ENSG00000168778	Low expressed	Well expressed	Not expressed
TGDS	ENSG00000088451	Low expressed	Well expressed	Low expressed
THRA	ENSG00000126351	Low expressed	Well expressed	Low expressed
TIMM8A	ENSG00000126953	Low expressed	Well expressed	Not expressed
TMEM135	ENSG00000166575	Low expressed	Well expressed	Not expressed
TMEM138	ENSG00000149483	Low expressed	Well expressed	Low expressed
TMEM216	ENSG00000187049	Low expressed	Well expressed	Low expressed
TMEM260	ENSG00000070269	Low expressed	Well expressed	Low expressed
TNNI2	ENSG00000130598	Not expressed	Not expressed	Well expressed
TOE1	ENSG00000132773	Low expressed	Well expressed	Low expressed
TREX1	ENSG00000213689	Low expressed	Well expressed	Well expressed
TRIP11	ENSG00000100815	Low expressed	Well expressed	Low expressed
TRIT1	ENSG00000043514	Low expressed	Well expressed	Low expressed
TSC1	ENSG00000165699	Low expressed	Well expressed	Low expressed
TSEN54	ENSG00000182173	Low expressed	Well expressed	Well expressed
TSHZ1	ENSG00000179981	Low expressed	Well expressed	Low expressed
TTC21B	ENSG00000123607	Low expressed	Well expressed	Low expressed
TTC7A	ENSG00000068724	Low expressed	Well expressed	Well expressed
TTC8	ENSG00000165533	Low expressed	Well expressed	Not expressed
TWIST1	ENSG00000122691	Not expressed	Well expressed	Not expressed
TWIST2	ENSG00000233608	Low expressed	Well expressed	Not expressed
UPF3B	ENSG00000125351	Low expressed	Well expressed	Low expressed
VDR	ENSG00000111424	Low expressed	Well expressed	Low expressed
VPS13B	ENSG00000132549	Low expressed	Well expressed	Low expressed
WDR19	ENSG00000157796	Low expressed	Well expressed	Not expressed
WDR35	ENSG00000118965	Low expressed	Well expressed	Not expressed
WDR73	ENSG00000177082	Low expressed	Well expressed	Low expressed
XPA	ENSG00000136936	Low expressed	Well expressed	Low expressed
XYLT1	ENSG00000103489	Low expressed	Well expressed	Low expressed
ZBTB16	ENSG00000109906	Not expressed	Well expressed	Low expressed
ZBTB40	ENSG00000184677	Low expressed	Well expressed	Low expressed
ZCCHC8	ENSG00000033030	Low expressed	Well expressed	Low expressed
ZEB1	ENSG00000148516	Low expressed	Well expressed	Low expressed
ZEB2	ENSG00000169554	Low expressed	Well expressed	Low expressed

Supplementary Table 5. Expression analysis of 2,020 curated genes associated with congenital and developmental disorders.

		Median TPM			Expression Conclusion		
		Amniotic Fluid Cells	Fibroblast_GTEx	Whole blood_GTEx	Amniotic Fluid Cells	Fibroblast_GTEx	Whole blood_GTEx
ENSG00000115414	*FN1*	3476	20890	1	Well expressed	Well expressed	Low expressed
ENSG00000100345	*MYH9*	2390	436	209	Well expressed	Well expressed	Well expressed
ENSG00000196230	*TUBB*	2106	892	76	Well expressed	Well expressed	Well expressed
ENSG00000196924	*FLNA*	1879	932	173	Well expressed	Well expressed	Well expressed
ENSG00000167552	*TUBA1A*	1141	237	117	Well expressed	Well expressed	Well expressed
ENSG00000087086	*FTL*	6147	12570	13155	Well expressed	Well expressed	Well expressed
ENSG00000149925	*ALDOA*	978	852	648	Well expressed	Well expressed	Well expressed
ENSG00000163453	*IGFBP7*	874	235	19	Well expressed	Well expressed	Well expressed
ENSG00000105372	*RPS19*	776	781	198	Well expressed	Well expressed	Well expressed
ENSG00000177469	*CAVIN1*	665	760	2	Well expressed	Well expressed	Low expressed
ENSG00000075624	*ACTB*	9312	6699	4804	Well expressed	Well expressed	Well expressed
ENSG00000198467	*TPM2*	520	470	5	Well expressed	Well expressed	Low expressed
ENSG00000157227	*MMP14*	636	620	2	Well expressed	Well expressed	Low expressed
ENSG00000187498	*COL4A1*	557	95	0	Well expressed	Well expressed	Not expressed
ENSG00000186468	*RPS23*	553	373	65	Well expressed	Well expressed	Well expressed
ENSG00000182117	*NOP10*	493	350	273	Well expressed	Well expressed	Well expressed
ENSG00000134871	*COL4A2*	488	132	0	Well expressed	Well expressed	Not expressed
ENSG00000197728	*RPS26*	419	495	117	Well expressed	Well expressed	Well expressed
ENSG00000165119	*HNRNPK*	411	521	193	Well expressed	Well expressed	Well expressed
ENSG00000170027	*YWHAG*	400	184	21	Well expressed	Well expressed	Well expressed
ENSG00000167468	*GPX4*	411	296	104	Well expressed	Well expressed	Well expressed
ENSG00000126267	*COX6B1*	382	232	155	Well expressed	Well expressed	Well expressed
ENSG00000136068	*FLNB*	386	89	2	Well expressed	Well expressed	Low expressed
ENSG00000135069	*PSAT1*	391	213	1	Well expressed	Well expressed	Low expressed
ENSG00000005884	*ITGA3*	391	58	1	Well expressed	Well expressed	Not expressed
ENSG00000125835	*SNRPB*	383	228	113	Well expressed	Well expressed	Well expressed
ENSG00000137267	*TUBB2A*	376	45	5	Well expressed	Well expressed	Low expressed
ENSG00000258947	*TUBB3*	385	48	0	Well expressed	Well expressed	Not expressed
ENSG00000169567	*HINT1*	358	251	28	Well expressed	Well expressed	Well expressed
ENSG00000102172	*SMS*	360	123	13	Well expressed	Well expressed	Well expressed
ENSG00000161203	*AP2M1*	364	489	129	Well expressed	Well expressed	Well expressed
ENSG00000244038	*DDOST*	355	426	69	Well expressed	Well expressed	Well expressed
ENSG00000102144	*PGK1*	348	374	236	Well expressed	Well expressed	Well expressed
ENSG00000058262	*SEC61A1*	362	396	49	Well expressed	Well expressed	Well expressed
ENSG00000177697	*CD151*	352	339	27	Well expressed	Well expressed	Well expressed
ENSG00000136238	*RAC1*	357	287	105	Well expressed	Well expressed	Well expressed
ENSG00000117984	*CTSD*	289	363	809	Well expressed	Well expressed	Well expressed
ENSG00000126458	*RRAS*	323	227	23	Well expressed	Well expressed	Well expressed
ENSG00000130741	*EIF2S3*	308	177	47	Well expressed	Well expressed	Well expressed
ENSG00000144381	*HSPD1*	301	287	31	Well expressed	Well expressed	Well expressed
ENSG00000147140	*NONO*	316	182	44	Well expressed	Well expressed	Well expressed
ENSG00000178952	*TUFM*	318	246	56	Well expressed	Well expressed	Well expressed
ENSG00000182871	*COL18A1*	292	27	23	Well expressed	Well expressed	Well expressed
ENSG00000137285	*TUBB2B*	300	2	0	Well expressed	Low expressed	Not expressed
ENSG00000078369	*GNB1*	283	327	152	Well expressed	Well expressed	Well expressed
ENSG00000119335	*SET*	275	239	32	Well expressed	Well expressed	Well expressed
ENSG00000188157	*AGRN*	290	32	1	Well expressed	Well expressed	Not expressed
ENSG00000131238	*PPT1*	257	95	124	Well expressed	Well expressed	Well expressed
ENSG00000130985	*UBA1*	263	173	72	Well expressed	Well expressed	Well expressed
ENSG00000152661	*GJA1*	238	200	0	Well expressed	Well expressed	Not expressed
ENSG00000116133	*DHCR24*	251	90	4	Well expressed	Well expressed	Low expressed
ENSG00000170558	*CDH2*	227	22	0	Well expressed	Well expressed	Not expressed
ENSG00000163041	*H3-3A*	246	29	121	Well expressed	Well expressed	Well expressed
ENSG00000087460	*GNAS*	232	245	183	Well expressed	Well expressed	Well expressed
ENSG00000175334	*BANF1*	224	188	34	Well expressed	Well expressed	Well expressed
ENSG00000163931	*TKT*	219	238	522	Well expressed	Well expressed	Well expressed
ENSG00000112335	*SNX3*	214	245	171	Well expressed	Well expressed	Well expressed
ENSG00000198910	*L1CAM*	197	0	0	Well expressed	Not expressed	Not expressed
ENSG00000125356	*NDUFA1*	206	340	76	Well expressed	Well expressed	Well expressed
ENSG00000117394	*SLC2A1*	209	71	29	Well expressed	Well expressed	Well expressed
ENSG00000146701	*MDH2*	217	177	37	Well expressed	Well expressed	Well expressed
ENSG00000091136	*LAMB1*	198	416	0	Well expressed	Well expressed	Not expressed
ENSG00000105974	*CAV1*	188	890	1	Well expressed	Well expressed	Not expressed
ENSG00000105568	*PPP2R1A*	210	116	34	Well expressed	Well expressed	Well expressed
ENSG00000160789	*LMNA*	194	393	6	Well expressed	Well expressed	Low expressed
ENSG00000107796	*ACTA2*	77	52	5	Well expressed	Well expressed	Low expressed
ENSG00000143549	*TPM3*	194	135	128	Well expressed	Well expressed	Well expressed
ENSG00000095139	*ARCN1*	199	201	35	Well expressed	Well expressed	Well expressed
ENSG00000145912	*NHP2*	203	177	24	Well expressed	Well expressed	Well expressed
ENSG00000130508	*PXDN*	185	169	0	Well expressed	Well expressed	Not expressed
ENSG00000178209	*PLEC*	202	212	33	Well expressed	Well expressed	Well expressed
ENSG00000164405	*UQCRQ*	185	237	23	Well expressed	Well expressed	Well expressed
ENSG00000072506	*HSD17B10*	191	88	35	Well expressed	Well expressed	Well expressed
ENSG00000125166	*GOT2*	192	103	12	Well expressed	Well expressed	Well expressed
ENSG00000132963	*POMP*	173	200	43	Well expressed	Well expressed	Well expressed
ENSG00000108561	*C1QBP*	174	205	22	Well expressed	Well expressed	Well expressed
ENSG00000065427	*KARS1*	178	143	36	Well expressed	Well expressed	Well expressed
ENSG00000150938	*CRIM1*	175	272	0	Well expressed	Well expressed	Not expressed
ENSG00000196305	*IARS1*	176	141	4	Well expressed	Well expressed	Low expressed
ENSG00000142949	*PTPRF*	186	23	0	Well expressed	Well expressed	Not expressed
ENSG00000143753	*DEGS1*	168	135	31	Well expressed	Well expressed	Well expressed
ENSG00000142798	*HSPG2*	179	143	0	Well expressed	Well expressed	Not expressed
ENSG00000141367	*CLTC*	182	186	26	Well expressed	Well expressed	Well expressed
ENSG00000084754	*HADHA*	174	194	83	Well expressed	Well expressed	Well expressed
ENSG00000183010	*PYCR1*	164	80	1	Well expressed	Well expressed	Not expressed
ENSG00000105329	*TGFB1*	169	132	306	Well expressed	Well expressed	Well expressed
ENSG00000070614	*NDST1*	157	86	9	Well expressed	Well expressed	Low expressed
ENSG00000166147	*FBN1*	138	296	0	Well expressed	Well expressed	Not expressed
ENSG00000163399	*ATP1A1*	167	209	39	Well expressed	Well expressed	Well expressed
ENSG00000072778	*ACADVL*	161	203	114	Well expressed	Well expressed	Well expressed

ENSG00000135387	*CAPRIN1*	163	111	15	Well expressed	Well expressed	Well expressed
ENSG00000070669	*ASNS*	141	114	8	Well expressed	Well expressed	Low expressed
ENSG00000136628	*EPRS1*	154	177	9	Well expressed	Well expressed	Low expressed
ENSG00000074071	*MRPS34*	146	141	36	Well expressed	Well expressed	Well expressed
ENSG00000146038	*DCDC2*	139	0	0	Well expressed	Not expressed	Not expressed
ENSG00000168288	*MMADHC*	143	145	58	Well expressed	Well expressed	Well expressed
ENSG00000169710	*FASN*	141	74	3	Well expressed	Well expressed	Low expressed
ENSG00000204262	*COL5A2*	125	346	0	Well expressed	Well expressed	Not expressed
ENSG00000090861	*AARS1*	141	191	10	Well expressed	Well expressed	Low expressed
ENSG00000143575	*HAX1*	127	129	40	Well expressed	Well expressed	Well expressed
ENSG00000179091	*CYC1*	143	186	62	Well expressed	Well expressed	Well expressed
ENSG00000213639	*PPP1CB*	134	65	37	Well expressed	Well expressed	Well expressed
ENSG00000185236	*RAB11B*	138	105	94	Well expressed	Well expressed	Well expressed
ENSG00000096696	*DSP*	128	10	0	Well expressed	Low expressed	Not expressed
ENSG00000172053	*QARS1*	139	127	41	Well expressed	Well expressed	Well expressed
ENSG00000116030	*SUMO1*	126	127	30	Well expressed	Well expressed	Well expressed
ENSG00000143368	*SF3B4*	129	87	46	Well expressed	Well expressed	Well expressed
ENSG00000126524	*SBDS*	133	171	10	Well expressed	Well expressed	Well expressed
ENSG00000078401	*EDN1*	112	2	1	Well expressed	Low expressed	Not expressed
ENSG00000100243	*CYB5R3*	126	316	53	Well expressed	Well expressed	Well expressed
ENSG00000166228	*PCBD1*	132	36	6	Well expressed	Well expressed	Low expressed
ENSG00000092969	*TGFB2*	115	2	0	Well expressed	Low expressed	Not expressed
ENSG00000083444	*PLOD1*	125	315	39	Well expressed	Well expressed	Well expressed
ENSG00000185825	*BCAP31*	123	182	129	Well expressed	Well expressed	Well expressed
ENSG00000148672	*GLUD1*	127	145	26	Well expressed	Well expressed	Well expressed
ENSG00000166136	*NDUFB8*	130	141	49	Well expressed	Well expressed	Well expressed
ENSG00000059573	*ALDH18A1*	120	96	3	Well expressed	Well expressed	Low expressed
ENSG00000198561	*CTNND1*	125	90	3	Well expressed	Well expressed	Low expressed
ENSG00000196365	*LONP1*	119	82	10	Well expressed	Well expressed	Well expressed
ENSG00000131100	*ATP6V1E1*	115	139	87	Well expressed	Well expressed	Well expressed
ENSG00000197694	*SPTAN1*	123	96	15	Well expressed	Well expressed	Well expressed
ENSG00000077380	*DYNC1I2*	121	97	10	Well expressed	Well expressed	Low expressed
ENSG00000125618	*PAX8*	120	2	0	Well expressed	Low expressed	Not expressed
ENSG00000162430	*SELENON*	117	167	9	Well expressed	Well expressed	Low expressed
ENSG00000147123	*NDUFB11*	117	211	89	Well expressed	Well expressed	Well expressed
ENSG00000124766	*SOX4*	117	8	1	Well expressed	Low expressed	Not expressed
ENSG00000163382	*NAXE*	117	51	14	Well expressed	Well expressed	Well expressed
ENSG00000119655	*NPC2*	106	87	74	Well expressed	Well expressed	Well expressed
ENSG00000135677	*GNS*	104	129	75	Well expressed	Well expressed	Well expressed
ENSG00000138363	*ATIC*	108	101	10	Well expressed	Well expressed	Low expressed
ENSG00000091483	*FH*	100	129	12	Well expressed	Well expressed	Well expressed
ENSG00000089289	*IGBP1*	110	86	41	Well expressed	Well expressed	Well expressed
ENSG00000126934	*MAP2K2*	111	129	96	Well expressed	Well expressed	Well expressed
ENSG00000130635	*COL5A1*	79	202	0	Well expressed	Well expressed	Not expressed
ENSG00000160710	*ADAR*	115	114	107	Well expressed	Well expressed	Well expressed
ENSG00000160877	*NACC1*	105	65	15	Well expressed	Well expressed	Well expressed
ENSG00000181222	*POLR2A*	105	129	47	Well expressed	Well expressed	Well expressed
ENSG00000097007	*ABL1*	102	91	4	Well expressed	Well expressed	Low expressed
ENSG00000105220	*GPI*	96	181	59	Well expressed	Well expressed	Well expressed
ENSG00000170275	*CRTAP*	102	162	20	Well expressed	Well expressed	Well expressed
ENSG00000108946	*PRKAR1A*	100	124	38	Well expressed	Well expressed	Well expressed
ENSG00000111642	*CHD4*	107	124	28	Well expressed	Well expressed	Well expressed
ENSG00000131653	*TRAF7*	100	88	25	Well expressed	Well expressed	Well expressed
ENSG00000203879	*GDI1*	97	98	100	Well expressed	Well expressed	Well expressed
ENSG00000090776	*EFNB1*	92	26	1	Well expressed	Well expressed	Low expressed
ENSG00000133818	*RRAS2*	86	67	1	Well expressed	Well expressed	Low expressed
ENSG00000141580	*WDR45B*	100	77	18	Well expressed	Well expressed	Well expressed
ENSG00000147224	*PRPS1*	87	59	14	Well expressed	Well expressed	Well expressed
ENSG00000126945	*HNRNPH2*	91	140	78	Well expressed	Well expressed	Well expressed
ENSG00000131165	*CHMP1A*	100	96	70	Well expressed	Well expressed	Well expressed
ENSG00000079459	*FDFT1*	98	53	32	Well expressed	Well expressed	Well expressed
ENSG00000179295	*PTPN11*	99	111	4	Well expressed	Well expressed	Low expressed
ENSG00000213281	*NRAS*	96	52	6	Well expressed	Well expressed	Low expressed
ENSG00000175390	*EIF3F*	100	102	27	Well expressed	Well expressed	Well expressed
ENSG00000163527	*STT3B*	83	124	23	Well expressed	Well expressed	Well expressed
ENSG00000099624	*ATP5F1D*	96	169	63	Well expressed	Well expressed	Well expressed
ENSG00000100412	*ACO2*	95	67	17	Well expressed	Well expressed	Well expressed
ENSG00000102119	*EMD*	95	139	54	Well expressed	Well expressed	Well expressed
ENSG00000168036	*CTNNB1*	92	167	26	Well expressed	Well expressed	Well expressed
ENSG00000111676	*ATN1*	92	121	9	Well expressed	Well expressed	Low expressed
ENSG00000004487	*KDM1A*	87	78	9	Well expressed	Well expressed	Low expressed
ENSG00000095752	*IL11*	89	5	0	Well expressed	Low expressed	Not expressed
ENSG00000184983	*NDUFA6*	83	80	15	Well expressed	Well expressed	Well expressed
ENSG00000086758	*HUWE1*	89	65	7	Well expressed	Well expressed	Low expressed
ENSG00000119681	*LTBP2*	85	99	1	Well expressed	Well expressed	Low expressed
ENSG00000143222	*UFC1*	86	84	34	Well expressed	Well expressed	Well expressed
ENSG00000103769	*RAB11A*	88	48	16	Well expressed	Well expressed	Well expressed
ENSG00000102103	*PQBP1*	88	45	31	Well expressed	Well expressed	Well expressed
ENSG00000113575	*PPP2CA*	82	75	35	Well expressed	Well expressed	Well expressed
ENSG00000067704	*IARS2*	88	108	9	Well expressed	Well expressed	Low expressed
ENSG00000105755	*ETHE1*	87	37	19	Well expressed	Well expressed	Well expressed
ENSG00000122140	*MRPS2*	87	56	7	Well expressed	Well expressed	Low expressed
ENSG00000137161	*CNPY3*	80	62	237	Well expressed	Well expressed	Well expressed
ENSG00000005893	*LAMP2*	76	112	72	Well expressed	Well expressed	Well expressed
ENSG00000004455	*AK2*	80	73	19	Well expressed	Well expressed	Well expressed
ENSG00000134910	*STT3A*	83	135	8	Well expressed	Well expressed	Low expressed
ENSG00000197780	*TAF13*	76	59	6	Well expressed	Well expressed	Low expressed
ENSG00000163513	*TGFBR2*	78	377	47	Well expressed	Well expressed	Well expressed
ENSG00000182004	*SNRPE*	76	125	6	Well expressed	Well expressed	Low expressed
ENSG00000000419	*DPM1*	79	90	12	Well expressed	Well expressed	Well expressed
ENSG00000103335	*PIEZO1*	80	95	12	Well expressed	Well expressed	Well expressed
ENSG00000102225	*CDK16*	79	67	19	Well expressed	Well expressed	Well expressed
ENSG00000197102	*DYNC1H1*	80	148	7	Well expressed	Well expressed	Low expressed

ENSG00000125505	*MBOAT7*	72	45	247	Well expressed	Well expressed	Well expressed
ENSG00000134250	*NOTCH2*	68	76	18	Well expressed	Well expressed	Well expressed
ENSG00000112640	*PPP2R5D*	76	59	18	Well expressed	Well expressed	Well expressed
ENSG00000169692	*AGPAT2*	78	71	71	Well expressed	Well expressed	Well expressed
ENSG00000129757	*CDKN1C*	41	5	4	Well expressed	Low expressed	Low expressed
ENSG00000197785	*ATAD3A*	73	32	5	Well expressed	Well expressed	Low expressed
ENSG00000170004	*CHD3*	74	74	29	Well expressed	Well expressed	Well expressed
ENSG00000160213	*CSTB*	73	127	66	Well expressed	Well expressed	Well expressed
ENSG00000147416	*ATP6V1B2*	71	93	161	Well expressed	Well expressed	Well expressed
ENSG00000107404	*DVL1*	75	64	10	Well expressed	Well expressed	Well expressed
ENSG00000165458	*INPPL1*	73	67	49	Well expressed	Well expressed	Well expressed
ENSG00000167792	*NDUFV1*	73	137	41	Well expressed	Well expressed	Well expressed
ENSG00000142186	*SCYL1*	74	94	46	Well expressed	Well expressed	Well expressed
ENSG00000125656	*CLPP*	75	122	34	Well expressed	Well expressed	Well expressed
ENSG00000131462	*TUBG1*	64	79	9	Well expressed	Well expressed	Low expressed
ENSG00000153187	*HNRNPU*	73	105	34	Well expressed	Well expressed	Well expressed
ENSG00000182197	*EXT1*	73	50	4	Well expressed	Well expressed	Low expressed
ENSG00000170266	*GLB1*	69	68	18	Well expressed	Well expressed	Well expressed
ENSG00000116863	*ADPRS*	75	45	17	Well expressed	Well expressed	Well expressed
ENSG00000105647	*PIK3R2*	72	44	6	Well expressed	Well expressed	Low expressed
ENSG00000102158	*MAGT1*	68	52	11	Well expressed	Well expressed	Well expressed
ENSG00000159140	*SON*	75	81	14	Well expressed	Well expressed	Well expressed
ENSG00000182492	*BGN*	36	578	2	Well expressed	Well expressed	Low expressed
ENSG00000164182	*NDUFAF2*	67	65	2	Well expressed	Well expressed	Low expressed
ENSG00000137693	*YAP1*	66	118	0	Well expressed	Well expressed	Not expressed
ENSG00000100504	*PYGL*	65	77	311	Well expressed	Well expressed	Well expressed
ENSG00000164258	*NDUFS4*	62	116	6	Well expressed	Well expressed	Low expressed
ENSG00000068366	*ACSL4*	66	49	20	Well expressed	Well expressed	Well expressed
ENSG00000072274	*TFRC*	57	148	11	Well expressed	Well expressed	Well expressed
ENSG00000068438	*FTSJ1*	72	49	8	Well expressed	Well expressed	Low expressed
ENSG00000197858	*GPAA1*	68	131	43	Well expressed	Well expressed	Well expressed
ENSG00000064601	*CTSA*	65	285	164	Well expressed	Well expressed	Well expressed
ENSG00000115866	*DARS1*	68	121	10	Well expressed	Well expressed	Low expressed
ENSG00000143799	*PARP1*	66	54	9	Well expressed	Well expressed	Low expressed
ENSG00000133026	*MYH10*	68	55	1	Well expressed	Well expressed	Not expressed
ENSG00000174775	*HRAS*	65	49	6	Well expressed	Well expressed	Low expressed
ENSG00000125107	*CNOT1*	66	63	17	Well expressed	Well expressed	Well expressed
ENSG00000215301	*DDX3X*	67	125	49	Well expressed	Well expressed	Well expressed
ENSG00000119669	*IRF2BPL*	62	29	16	Well expressed	Well expressed	Well expressed
ENSG00000177666	*PNPLA2*	64	113	174	Well expressed	Well expressed	Well expressed
ENSG00000160200	*CBS*	65	3	0	Well expressed	Low expressed	Not expressed
ENSG00000115275	*MOGS*	65	75	22	Well expressed	Well expressed	Well expressed
ENSG00000172893	*DHCR7*	60	25	8	Well expressed	Well expressed	Low expressed
ENSG00000101310	*SEC23B*	64	35	16	Well expressed	Well expressed	Well expressed
ENSG00000142208	*AKT1*	65	81	27	Well expressed	Well expressed	Well expressed
ENSG00000175115	*PACS1*	61	47	57	Well expressed	Well expressed	Well expressed
ENSG00000179950	*PUF60*	65	81	25	Well expressed	Well expressed	Well expressed
ENSG00000177628	*GBA*	59	44	33	Well expressed	Well expressed	Well expressed
ENSG00000100934	*SEC23A*	61	116	4	Well expressed	Well expressed	Low expressed
ENSG00000132763	*MMACHC*	65	7	0	Well expressed	Low expressed	Not expressed
ENSG00000179163	*FUCA1*	55	5	11	Well expressed	Low expressed	Well expressed
ENSG00000152952	*PLOD2*	45	252	0	Well expressed	Well expressed	Not expressed
ENSG00000120948	*TARDBP*	59	83	16	Well expressed	Well expressed	Well expressed
ENSG00000185551	*NR2F2*	55	42	0	Well expressed	Well expressed	Not expressed
ENSG00000142178	*SIK1*	62	0	0	Well expressed	Not expressed	Not expressed
ENSG00000198682	*PAPSS2*	56	97	2	Well expressed	Well expressed	Low expressed
ENSG00000275410	*HNF1B*	66	0	0	Well expressed	Not expressed	Not expressed
ENSG00000066117	*SMARCD1*	64	39	16	Well expressed	Well expressed	Well expressed
ENSG00000131174	*COX7B*	58	53	5	Well expressed	Well expressed	Low expressed
ENSG00000176692	*FOXC2*	55	11	0	Well expressed	Well expressed	Not expressed
ENSG00000138095	*LRPPRC*	60	69	4	Well expressed	Well expressed	Low expressed
ENSG00000196411	*EPHB4*	60	26	5	Well expressed	Well expressed	Low expressed
ENSG00000153827	*TRIP12*	61	74	23	Well expressed	Well expressed	Well expressed
ENSG00000171135	*JAGN1*	62	68	14	Well expressed	Well expressed	Well expressed
ENSG00000072364	*AFF4*	51	47	5	Well expressed	Well expressed	Low expressed
ENSG00000166949	*SMAD3*	54	59	4	Well expressed	Well expressed	Low expressed
ENSG00000101210	*EEF1A2*	51	1	1	Well expressed	Not expressed	Not expressed
ENSG00000102030	*NAA10*	55	79	27	Well expressed	Well expressed	Well expressed
ENSG00000183691	*NOG*	53	2	0	Well expressed	Low expressed	Not expressed
ENSG00000136717	*BIN1*	58	46	23	Well expressed	Well expressed	Well expressed
ENSG00000239900	*ADSL*	58	31	6	Well expressed	Well expressed	Low expressed
ENSG00000151348	*EXT2*	58	102	5	Well expressed	Well expressed	Low expressed
ENSG00000107745	*MICU1*	57	54	34	Well expressed	Well expressed	Well expressed
ENSG00000110717	*NDUFS8*	57	66	20	Well expressed	Well expressed	Well expressed
ENSG00000127603	*MACF1*	57	24	4	Well expressed	Well expressed	Low expressed
ENSG00000079739	*PGM1*	55	60	20	Well expressed	Well expressed	Well expressed
ENSG00000265241	*RBM8A*	58	39	16	Well expressed	Well expressed	Well expressed
ENSG00000178449	*COX14*	55	49	15	Well expressed	Well expressed	Well expressed
ENSG00000161202	*DVL3*	56	62	21	Well expressed	Well expressed	Well expressed
ENSG00000038382	*TRIO*	56	58	1	Well expressed	Well expressed	Low expressed
ENSG00000111678	*C12orf57*	57	81	17	Well expressed	Well expressed	Well expressed
ENSG00000125965	*GDF5*	51	7	0	Well expressed	Low expressed	Not expressed
ENSG00000101333	*PLCB4*	50	14	0	Well expressed	Well expressed	Not expressed
ENSG00000007168	*PAFAH1B1*	53	74	26	Well expressed	Well expressed	Well expressed
ENSG00000136699	*SMPD4*	56	40	7	Well expressed	Well expressed	Low expressed
ENSG00000204264	*PSMB8*	57	81	155	Well expressed	Well expressed	Well expressed
ENSG00000174705	*SH3PXD2B*	56	36	2	Well expressed	Well expressed	Low expressed
ENSG00000084774	*CAD*	56	37	3	Well expressed	Well expressed	Low expressed
ENSG00000088256	*GNA11*	56	67	1	Well expressed	Well expressed	Low expressed
ENSG00000197265	*GTF2E2*	54	45	9	Well expressed	Well expressed	Low expressed
ENSG00000204386	*NEU1*	47	51	37	Well expressed	Well expressed	Well expressed
ENSG00000092621	*PHGDH*	52	83	1	Well expressed	Well expressed	Not expressed
ENSG00000184014	*DENND5A*	55	67	53	Well expressed	Well expressed	Well expressed

ENSG00000169032	*MAP2K1*	57	48	22	Well expressed	Well expressed	Well expressed
ENSG00000185803	*SLC52A2*	55	68	8	Well expressed	Well expressed	Low expressed
ENSG00000100883	*SRP54*	52	73	17	Well expressed	Well expressed	Well expressed
ENSG00000159363	*ATP13A2*	52	24	12	Well expressed	Well expressed	Well expressed
ENSG00000105722	*ERF*	54	51	41	Well expressed	Well expressed	Well expressed
ENSG00000172062	*SMN1*	50	6	1	Well expressed	Low expressed	Not expressed
ENSG00000123064	*DDX54*	54	43	10	Well expressed	Well expressed	Low expressed
ENSG00000168282	*MGAT2*	55	36	4	Well expressed	Well expressed	Low expressed
ENSG00000164754	*RAD21*	52	90	28	Well expressed	Well expressed	Well expressed
ENSG00000162735	*PEX19*	54	49	13	Well expressed	Well expressed	Well expressed
ENSG00000173402	*DAG1*	49	46	1	Well expressed	Well expressed	Low expressed
ENSG00000205336	*ADGRG1*	51	1	9	Well expressed	Not expressed	Low expressed
ENSG00000104774	*MAN2B1*	53	64	74	Well expressed	Well expressed	Well expressed
ENSG00000184009	*ACTG1*	9727	4888	1087	Well expressed	Well expressed	Well expressed
ENSG00000106003	*LFNG*	52	5	24	Well expressed	Low expressed	Well expressed
ENSG00000068120	*COASY*	52	58	28	Well expressed	Well expressed	Well expressed
ENSG00000117308	*GALE*	53	12	3	Well expressed	Well expressed	Low expressed
ENSG00000065534	*MYLK*	48	18	1	Well expressed	Well expressed	Not expressed
ENSG00000004961	*HCCS*	49	31	8	Well expressed	Well expressed	Low expressed
ENSG00000116337	*AMPD2*	52	36	55	Well expressed	Well expressed	Well expressed
ENSG00000153989	*NUS1*	52	33	3	Well expressed	Well expressed	Low expressed
ENSG00000150961	*SEC24D*	49	90	7	Well expressed	Well expressed	Low expressed
ENSG00000010292	*NCAPD2*	38	43	10	Well expressed	Well expressed	Well expressed
ENSG00000108883	*EFTUD2*	51	61	18	Well expressed	Well expressed	Well expressed
ENSG00000146733	*PSPH*	47	27	1	Well expressed	Well expressed	Not expressed
ENSG00000085998	*POMGNT1*	50	34	3	Well expressed	Well expressed	Low expressed
ENSG00000166311	*SMPD1*	48	79	16	Well expressed	Well expressed	Well expressed
ENSG00000152104	*PTPN14*	45	33	0	Well expressed	Well expressed	Not expressed
ENSG00000130821	*SLC6A8*	49	21	12	Well expressed	Well expressed	Well expressed
ENSG00000182872	*RBM10*	50	47	34	Well expressed	Well expressed	Well expressed
ENSG00000151552	*QDPR*	50	33	3	Well expressed	Well expressed	Low expressed
ENSG00000116906	*GNPAT*	48	47	12	Well expressed	Well expressed	Well expressed
ENSG00000106397	*PLOD3*	48	161	10	Well expressed	Well expressed	Low expressed
ENSG00000277258	*PCGF2*	50	48	0	Well expressed	Well expressed	Not expressed
ENSG00000165915	*SLC39A13*	48	87	9	Well expressed	Well expressed	Low expressed
ENSG00000127616	*SMARCA4*	51	49	9	Well expressed	Well expressed	Low expressed
ENSG00000140374	*ETFA*	49	83	19	Well expressed	Well expressed	Well expressed
ENSG00000125398	*SOX9*	50	1	0	Well expressed	Not expressed	Not expressed
ENSG00000170445	*HARS1*	47	60	10	Well expressed	Well expressed	Low expressed
ENSG00000099942	*CRKL*	50	47	20	Well expressed	Well expressed	Well expressed
ENSG00000131828	*PDHA1*	49	50	9	Well expressed	Well expressed	Low expressed
ENSG00000110367	*DDX6*	48	67	26	Well expressed	Well expressed	Well expressed
ENSG00000184254	*ALDH1A3*	39	8	0	Well expressed	Low expressed	Not expressed
ENSG00000166340	*TPP1*	45	138	85	Well expressed	Well expressed	Well expressed
ENSG00000165704	*HPRT1*	44	58	10	Well expressed	Well expressed	Well expressed
ENSG00000044090	*CUL7*	48	46	4	Well expressed	Well expressed	Low expressed
ENSG00000141543	*EIF4A3*	45	157	62	Well expressed	Well expressed	Well expressed
ENSG00000146282	*RARS2*	46	47	5	Well expressed	Well expressed	Low expressed
ENSG00000130826	*DKC1*	46	68	8	Well expressed	Well expressed	Low expressed
ENSG00000120686	*UFM1*	46	90	5	Well expressed	Well expressed	Low expressed
ENSG00000148290	*SURF1*	44	35	32	Well expressed	Well expressed	Well expressed
ENSG00000012061	*ERCC1*	46	57	12	Well expressed	Well expressed	Well expressed
ENSG00000180900	*SCRIB*	47	40	7	Well expressed	Well expressed	Low expressed
ENSG00000095059	*DHPS*	46	62	30	Well expressed	Well expressed	Well expressed
ENSG00000111752	*PHC1*	47	9	1	Well expressed	Low expressed	Low expressed
ENSG00000151835	*SACS*	40	23	0	Well expressed	Well expressed	Not expressed
ENSG00000077721	*UBE2A*	44	92	35	Well expressed	Well expressed	Well expressed
ENSG00000164930	*FZD6*	43	43	0	Well expressed	Well expressed	Not expressed
ENSG00000106799	*TGFBR1*	42	28	13	Well expressed	Well expressed	Well expressed
ENSG00000006744	*ELAC2*	45	52	11	Well expressed	Well expressed	Well expressed
ENSG00000125944	*HNRNPR*	46	51	7	Well expressed	Well expressed	Low expressed
ENSG00000170892	*TSEN34*	47	39	71	Well expressed	Well expressed	Well expressed
ENSG00000114573	*ATP6V1A*	43	33	21	Well expressed	Well expressed	Well expressed
ENSG00000147383	*NSDHL*	46	36	5	Well expressed	Well expressed	Low expressed
ENSG00000072803	*FBXW11*	47	33	4	Well expressed	Well expressed	Low expressed
ENSG00000129255	*MPDU1*	44	64	22	Well expressed	Well expressed	Well expressed
ENSG00000177706	*FAM20C*	40	71	4	Well expressed	Well expressed	Low expressed
ENSG00000159423	*ALDH4A1*	44	12	3	Well expressed	Well expressed	Low expressed
ENSG00000073578	*SDHA*	44	132	28	Well expressed	Well expressed	Well expressed
ENSG00000104884	*ERCC2*	45	40	3	Well expressed	Well expressed	Low expressed
ENSG00000084073	*ZMPSTE24*	41	63	12	Well expressed	Well expressed	Well expressed
ENSG00000095380	*NANS*	42	24	9	Well expressed	Well expressed	Low expressed
ENSG00000170581	*STAT2*	42	114	25	Well expressed	Well expressed	Well expressed
ENSG00000198218	*QRICH1*	46	54	17	Well expressed	Well expressed	Well expressed
ENSG00000037474	*NSUN2*	44	71	10	Well expressed	Well expressed	Low expressed
ENSG00000108588	*CCDC47*	43	77	12	Well expressed	Well expressed	Well expressed
ENSG00000126522	*ASL*	40	66	17	Well expressed	Well expressed	Well expressed
ENSG00000124299	*PEPD*	43	61	17	Well expressed	Well expressed	Well expressed
ENSG00000117385	*P3H1*	41	107	9	Well expressed	Well expressed	Low expressed
ENSG00000186111	*PIP5K1C*	40	29	9	Well expressed	Well expressed	Low expressed
ENSG00000213024	*NUP62*	44	40	10	Well expressed	Well expressed	Well expressed
ENSG00000248098	*BCKDHA*	42	29	31	Well expressed	Well expressed	Well expressed
ENSG00000156471	*PTDSS1*	42	47	18	Well expressed	Well expressed	Well expressed
ENSG00000172534	*HCFC1*	41	27	7	Well expressed	Well expressed	Low expressed
ENSG00000169750	*RAC3*	43	12	1	Well expressed	Well expressed	Not expressed
ENSG00000163541	*SUCLG1*	42	66	24	Well expressed	Well expressed	Well expressed
ENSG00000130707	*ASS1*	33	108	1	Well expressed	Well expressed	Not expressed
ENSG00000114480	*GBE1*	39	147	9	Well expressed	Well expressed	Low expressed
ENSG00000126012	*KDM5C*	40	40	25	Well expressed	Well expressed	Well expressed
ENSG00000110619	*CARS1*	40	48	17	Well expressed	Well expressed	Well expressed
ENSG00000156709	*AIFM1*	43	30	7	Well expressed	Well expressed	Low expressed
ENSG00000156110	*ADK*	40	34	4	Well expressed	Well expressed	Low expressed
ENSG00000010404	*IDS*	38	32	40	Well expressed	Well expressed	Well expressed

ENSG00000092330	*TINF2*	42	43	63	Well expressed	Well expressed	Well expressed
ENSG00000143183	*TMCO1*	40	43	5	Well expressed	Well expressed	Low expressed
ENSG00000091140	*DLD*	38	55	9	Well expressed	Well expressed	Low expressed
ENSG00000078747	*ITCH*	41	33	14	Well expressed	Well expressed	Well expressed
ENSG00000033170	*FUT8*	42	26	1	Well expressed	Well expressed	Not expressed
ENSG00000116096	*SPR*	42	34	2	Well expressed	Well expressed	Low expressed
ENSG00000157933	*SKI*	41	59	12	Well expressed	Well expressed	Well expressed
ENSG00000196642	*RABL6*	41	45	17	Well expressed	Well expressed	Well expressed
ENSG00000104331	*BPNT2*	38	56	4	Well expressed	Well expressed	Low expressed
ENSG00000110719	*TCIRG1*	40	83	474	Well expressed	Well expressed	Well expressed
ENSG00000104723	*TUSC3*	40	63	0	Well expressed	Well expressed	Not expressed
ENSG00000131779	*PEX11B*	41	50	14	Well expressed	Well expressed	Well expressed
ENSG00000103051	*COG4*	40	59	21	Well expressed	Well expressed	Well expressed
ENSG00000153201	*RANBP2*	35	37	6	Well expressed	Well expressed	Low expressed
ENSG00000091409	*ITGA6*	38	3	3	Well expressed	Low expressed	Low expressed
ENSG00000141076	*UTP4*	38	31	3	Well expressed	Well expressed	Low expressed
ENSG00000157911	*PEX10*	39	20	2	Well expressed	Well expressed	Low expressed
ENSG00000167114	*SLC27A4*	39	22	5	Well expressed	Well expressed	Low expressed
ENSG00000105819	*PMPCB*	38	63	13	Well expressed	Well expressed	Well expressed
ENSG00000136868	*SLC31A1*	38	42	10	Well expressed	Well expressed	Low expressed
ENSG00000197170	*PSMD12*	38	39	7	Well expressed	Well expressed	Low expressed
ENSG00000119333	*DYNC2I2*	37	32	4	Well expressed	Well expressed	Low expressed
ENSG00000147471	*PLPBP*	39	38	16	Well expressed	Well expressed	Well expressed
ENSG00000134851	*TMEM165*	40	62	6	Well expressed	Well expressed	Low expressed
ENSG00000149541	*B3GAT3*	40	49	22	Well expressed	Well expressed	Well expressed
ENSG00000013375	*PGM3*	40	42	1	Well expressed	Well expressed	Not expressed
ENSG00000187555	*USP7*	39	54	20	Well expressed	Well expressed	Well expressed
ENSG00000131844	*MCCC2*	40	22	3	Well expressed	Well expressed	Low expressed
ENSG00000161217	*PCYT1A*	36	43	25	Well expressed	Well expressed	Well expressed
ENSG00000100811	*YY1*	39	37	11	Well expressed	Well expressed	Well expressed
ENSG00000122126	*OCRL*	37	27	1	Well expressed	Well expressed	Low expressed
ENSG00000124486	*USP9X*	36	45	12	Well expressed	Well expressed	Well expressed
ENSG00000141349	*G6PC3*	37	106	13	Well expressed	Well expressed	Well expressed
ENSG00000055208	*TAB2*	35	46	17	Well expressed	Well expressed	Well expressed
ENSG00000087111	*PIGS*	38	55	24	Well expressed	Well expressed	Well expressed
ENSG00000108312	*UBTF*	37	53	17	Well expressed	Well expressed	Well expressed
ENSG00000029993	*HMGB3*	35	25	1	Well expressed	Well expressed	Low expressed
ENSG00000141759	*TXNL4A*	36	28	7	Well expressed	Well expressed	Low expressed
ENSG00000119523	*ALG2*	38	47	5	Well expressed	Well expressed	Low expressed
ENSG00000048392	*RRM2B*	35	25	5	Well expressed	Well expressed	Low expressed
ENSG00000119977	*TCTN3*	37	62	4	Well expressed	Well expressed	Low expressed
ENSG00000119401	*TRIM32*	38	25	1	Well expressed	Well expressed	Not expressed
ENSG00000145715	*RASA1*	30	38	4	Well expressed	Well expressed	Low expressed
ENSG00000085491	*SLC25A24*	39	33	3	Well expressed	Well expressed	Low expressed
ENSG00000076242	*MLH1*	34	34	7	Well expressed	Well expressed	Low expressed
ENSG00000204560	*DHX16*	37	39	23	Well expressed	Well expressed	Well expressed
ENSG00000049860	*HEXB*	35	138	39	Well expressed	Well expressed	Well expressed
ENSG00000112414	*ADGRG6*	32	2	0	Well expressed	Low expressed	Not expressed
ENSG00000099246	*RAB18*	35	38	10	Well expressed	Well expressed	Low expressed
ENSG00000115839	*RAB3GAP1*	36	41	5	Well expressed	Well expressed	Low expressed
ENSG00000132153	*DHX30*	36	37	9	Well expressed	Well expressed	Low expressed
ENSG00000198363	*ASPH*	31	91	6	Well expressed	Well expressed	Low expressed
ENSG00000014919	*COX15*	35	32	10	Well expressed	Well expressed	Well expressed
ENSG00000140521	*POLG*	38	36	18	Well expressed	Well expressed	Well expressed
ENSG00000108784	*NAGLU*	34	41	9	Well expressed	Well expressed	Low expressed
ENSG00000229833	*PET100*	35	22	10	Well expressed	Well expressed	Well expressed
ENSG00000104907	*TRMT1*	37	45	15	Well expressed	Well expressed	Well expressed
ENSG00000228716	*DHFR*	21	18	2	Well expressed	Well expressed	Low expressed
ENSG00000100596	*SPTLC2*	36	18	22	Well expressed	Well expressed	Well expressed
ENSG00000143815	*LBR*	34	28	41	Well expressed	Well expressed	Well expressed
ENSG00000167522	*ANKRD11*	37	36	10	Well expressed	Well expressed	Low expressed
ENSG00000124920	*MYRF*	25	1	0	Well expressed	Low expressed	Not expressed
ENSG00000158290	*CUL4B*	29	96	11	Well expressed	Well expressed	Well expressed
ENSG00000130005	*GAMT*	35	25	2	Well expressed	Well expressed	Low expressed
ENSG00000120725	*SIL1*	33	41	8	Well expressed	Well expressed	Low expressed
ENSG00000165410	*CFL2*	32	48	0	Well expressed	Well expressed	Not expressed
ENSG00000204351	*SKIV2L*	35	39	29	Well expressed	Well expressed	Well expressed
ENSG00000196998	*WDR45*	38	30	49	Well expressed	Well expressed	Well expressed
ENSG00000122863	*CHST3*	33	24	0	Well expressed	Well expressed	Not expressed
ENSG00000049130	*KITLG*	30	66	0	Well expressed	Well expressed	Not expressed
ENSG00000147155	*EBP*	33	27	11	Well expressed	Well expressed	Well expressed
ENSG00000284194	*SCO2*	36	60	18	Well expressed	Well expressed	Well expressed
ENSG00000136463	*TACO1*	35	42	7	Well expressed	Well expressed	Low expressed
ENSG00000101126	*ADNP*	33	40	7	Well expressed	Well expressed	Low expressed
ENSG00000168214	*RBPJ*	29	39	28	Well expressed	Well expressed	Well expressed
ENSG00000188706	*ZDHHC9*	33	45	1	Well expressed	Well expressed	Low expressed
ENSG00000110871	*COQ5*	33	49	8	Well expressed	Well expressed	Low expressed
ENSG00000138814	*PPP3CA*	33	32	12	Well expressed	Well expressed	Well expressed
ENSG00000173898	*SPTBN2*	34	0	0	Well expressed	Not expressed	Not expressed
ENSG00000163703	*CRELD1*	30	17	6	Well expressed	Well expressed	Low expressed
ENSG00000162496	*DHRS3*	29	29	10	Well expressed	Well expressed	Low expressed
ENSG00000144659	*SLC25A38*	34	47	18	Well expressed	Well expressed	Well expressed
ENSG00000119899	*SLC17A5*	30	41	5	Well expressed	Well expressed	Low expressed
ENSG00000154767	*XPC*	33	82	13	Well expressed	Well expressed	Well expressed
ENSG00000139613	*SMARCC2*	33	69	19	Well expressed	Well expressed	Well expressed
ENSG00000094914	*AAAS*	32	54	14	Well expressed	Well expressed	Well expressed
ENSG00000107521	*HPS1*	34	41	48	Well expressed	Well expressed	Well expressed
ENSG00000177239	*MAN1B1*	32	63	17	Well expressed	Well expressed	Well expressed
ENSG00000015171	*ZMYND11*	33	51	3	Well expressed	Well expressed	Low expressed
ENSG00000160688	*FLAD1*	33	14	7	Well expressed	Well expressed	Low expressed
ENSG00000103005	*USB1*	32	29	54	Well expressed	Well expressed	Well expressed
ENSG00000220205	*VAMP2*	31	43	69	Well expressed	Well expressed	Well expressed
ENSG00000137055	*PLAA*	31	25	6	Well expressed	Well expressed	Low expressed

ENSG00000178537	*SLC25A20*	29	16	17	Well expressed	Well expressed	Well expressed
ENSG00000127463	*EMC1*	33	42	2	Well expressed	Well expressed	Low expressed
ENSG00000179085	*DPM3*	31	67	17	Well expressed	Well expressed	Well expressed
ENSG00000128928	*IVD*	33	39	5	Well expressed	Well expressed	Low expressed
ENSG00000176022	*B3GALT6*	31	30	4	Well expressed	Well expressed	Low expressed
ENSG00000162923	*WDR26*	29	53	38	Well expressed	Well expressed	Well expressed
ENSG00000143811	*PYCR2*	31	77	30	Well expressed	Well expressed	Well expressed
ENSG00000181830	*SLC35C1*	32	28	5	Well expressed	Well expressed	Low expressed
ENSG00000108055	*SMC3*	29	52	7	Well expressed	Well expressed	Low expressed
ENSG00000132155	*RAF1*	33	121	131	Well expressed	Well expressed	Well expressed
ENSG00000138777	*PPA2*	30	39	8	Well expressed	Well expressed	Low expressed
ENSG00000023228	*NDUFS1*	33	24	3	Well expressed	Well expressed	Low expressed
ENSG00000150768	*DLAT*	32	31	2	Well expressed	Well expressed	Low expressed
ENSG00000004399	*PLXND1*	30	65	13	Well expressed	Well expressed	Well expressed
ENSG00000079432	*CIC*	31	25	28	Well expressed	Well expressed	Well expressed
ENSG00000049759	*NEDD4L*	33	2	1	Well expressed	Low expressed	Low expressed
ENSG00000142655	*PEX14*	32	22	7	Well expressed	Well expressed	Low expressed
ENSG00000125875	*TBC1D20*	31	38	11	Well expressed	Well expressed	Well expressed
ENSG00000130414	*NDUFA10*	32	44	10	Well expressed	Well expressed	Low expressed
ENSG00000120733	*KDM3B*	33	32	23	Well expressed	Well expressed	Well expressed
ENSG00000156467	*UQCRB*	30	65	4	Well expressed	Well expressed	Low expressed
ENSG00000158615	*PPP1R15B*	31	32	22	Well expressed	Well expressed	Well expressed
ENSG00000138382	*METTL5*	31	52	3	Well expressed	Well expressed	Low expressed
ENSG00000256053	*COA8*	31	18	4	Well expressed	Well expressed	Low expressed
ENSG00000177426	*TGIF1*	31	29	4	Well expressed	Well expressed	Low expressed
ENSG00000171204	*TMEM126B*	30	38	4	Well expressed	Well expressed	Low expressed
ENSG00000177646	*ACAD9*	31	31	6	Well expressed	Well expressed	Low expressed
ENSG00000185963	*BICD2*	31	35	13	Well expressed	Well expressed	Well expressed
ENSG00000125352	*RNF113A*	30	29	26	Well expressed	Well expressed	Well expressed
ENSG00000164022	*AIMP1*	29	60	5	Well expressed	Well expressed	Low expressed
ENSG00000108175	*ZMIZ1*	30	30	6	Well expressed	Well expressed	Low expressed
ENSG00000100299	*ARSA*	29	41	43	Well expressed	Well expressed	Well expressed
ENSG00000198954	*KIFBP*	28	22	1	Well expressed	Well expressed	Low expressed
ENSG00000174173	*TRMT10C*	29	40	3	Well expressed	Well expressed	Low expressed
ENSG00000106080	*FKBP14*	28	34	0	Well expressed	Well expressed	Not expressed
ENSG00000156052	*GNAQ*	27	30	18	Well expressed	Well expressed	Well expressed
ENSG00000105429	*MEGF8*	29	29	1	Well expressed	Well expressed	Low expressed
ENSG00000213995	*NAXD*	30	47	10	Well expressed	Well expressed	Well expressed
ENSG00000114209	*PDCD10*	30	34	8	Well expressed	Well expressed	Low expressed
ENSG00000112249	*ASCC3*	29	32	1	Well expressed	Well expressed	Low expressed
ENSG00000114054	*PCCB*	29	15	3	Well expressed	Well expressed	Low expressed
ENSG00000117682	*DHDDS*	29	27	5	Well expressed	Well expressed	Low expressed
ENSG00000168056	*LTBP3*	28	72	7	Well expressed	Well expressed	Low expressed
ENSG00000169599	*NFU1*	29	25	4	Well expressed	Well expressed	Low expressed
ENSG00000100288	*CHKB*	30	32	51	Well expressed	Well expressed	Well expressed
ENSG00000126603	*GLIS2*	29	24	1	Well expressed	Well expressed	Not expressed
ENSG00000090006	*LTBP4*	27	24	7	Well expressed	Well expressed	Low expressed
ENSG00000081923	*ATP8B1*	24	29	0	Well expressed	Well expressed	Not expressed
ENSG00000132024	*CC2D1A*	29	21	15	Well expressed	Well expressed	Well expressed
ENSG00000141867	*BRD4*	29	22	20	Well expressed	Well expressed	Well expressed
ENSG00000103249	*CLCN7*	27	29	16	Well expressed	Well expressed	Well expressed
ENSG00000177030	*DEAF1*	30	17	6	Well expressed	Well expressed	Low expressed
ENSG00000262919	*CCNQ*	29	23	9	Well expressed	Well expressed	Low expressed
ENSG00000141385	*AFG3L2*	29	50	6	Well expressed	Well expressed	Low expressed
ENSG00000144591	*GMPPA*	28	56	14	Well expressed	Well expressed	Well expressed
ENSG00000127554	*GFER*	29	17	5	Well expressed	Well expressed	Low expressed
ENSG00000166685	*COG1*	28	27	8	Well expressed	Well expressed	Low expressed
ENSG00000140905	*GCSH*	30	9	0	Well expressed	Low expressed	Not expressed
ENSG00000140262	*TCF12*	26	37	2	Well expressed	Well expressed	Low expressed
ENSG00000068796	*KIF2A*	30	28	5	Well expressed	Well expressed	Low expressed
ENSG00000135821	*GLUL*	30	167	207	Well expressed	Well expressed	Well expressed
ENSG00000151779	*NBAS*	29	24	3	Well expressed	Well expressed	Low expressed
ENSG00000139180	*NDUFA9*	29	15	4	Well expressed	Well expressed	Low expressed
ENSG00000101384	*JAG1*	20	11	1	Well expressed	Well expressed	Not expressed
ENSG00000132842	*AP3B1*	27	43	9	Well expressed	Well expressed	Low expressed
ENSG00000159579	*RSPRY1*	26	34	5	Well expressed	Well expressed	Low expressed
ENSG00000107201	*DDX58*	28	7	7	Well expressed	Low expressed	Low expressed
ENSG00000145386	*CCNA2*	11	40	1	Well expressed	Well expressed	Low expressed
ENSG00000012232	*EXTL3*	27	21	12	Well expressed	Well expressed	Well expressed
ENSG00000131873	*CHSY1*	28	31	12	Well expressed	Well expressed	Well expressed
ENSG00000151445	*VIPAS39*	28	24	12	Well expressed	Well expressed	Well expressed
ENSG00000198793	*MTOR*	28	18	3	Well expressed	Well expressed	Low expressed
ENSG00000162337	*LRP5*	26	19	0	Well expressed	Well expressed	Not expressed
ENSG00000118762	*PKD2*	22	52	1	Well expressed	Well expressed	Low expressed
ENSG00000090061	*CCNK*	28	21	9	Well expressed	Well expressed	Low expressed
ENSG00000169515	*CCDC8*	27	20	0	Well expressed	Well expressed	Not expressed
ENSG00000054598	*FOXC1*	25	7	0	Well expressed	Low expressed	Not expressed
ENSG00000075239	*ACAT1*	27	38	3	Well expressed	Well expressed	Low expressed
ENSG00000119392	*GLE1*	26	32	15	Well expressed	Well expressed	Well expressed
ENSG00000198677	*TTC37*	25	76	4	Well expressed	Well expressed	Low expressed
ENSG00000169105	*CHST14*	27	30	2	Well expressed	Well expressed	Low expressed
ENSG00000018510	*AGPS*	29	21	3	Well expressed	Well expressed	Low expressed
ENSG00000196839	*ADA*	25	19	8	Well expressed	Well expressed	Low expressed
ENSG00000080603	*SRCAP*	26	29	12	Well expressed	Well expressed	Well expressed
ENSG00000104320	*NBN*	26	35	9	Well expressed	Well expressed	Low expressed
ENSG00000104164	*BLOC1S6*	26	30	4	Well expressed	Well expressed	Low expressed
ENSG00000108061	*SHOC2*	24	40	18	Well expressed	Well expressed	Well expressed
ENSG00000131263	*RLIM*	24	29	10	Well expressed	Well expressed	Low expressed
ENSG00000108395	*TRIM37*	25	19	2	Well expressed	Well expressed	Low expressed
ENSG00000163820	*FYCO1*	26	18	2	Well expressed	Well expressed	Low expressed
ENSG00000126749	*EMG1*	26	39	6	Well expressed	Well expressed	Low expressed
ENSG00000149269	*PAK1*	27	26	66	Well expressed	Well expressed	Well expressed
ENSG00000163956	*LRPAP1*	26	35	41	Well expressed	Well expressed	Well expressed

ENSG00000214160	*ALG3*	25	65	12	Well expressed	Well expressed	Well expressed
ENSG00000072501	*SMC1A*	24	24	6	Well expressed	Well expressed	Low expressed
ENSG00000070610	*GBA2*	26	26	26	Well expressed	Well expressed	Well expressed
ENSG00000131652	*THOC6*	23	46	18	Well expressed	Well expressed	Well expressed
ENSG00000115317	*HTRA2*	27	34	21	Well expressed	Well expressed	Well expressed
ENSG00000068654	*POLR1A*	26	20	1	Well expressed	Well expressed	Not expressed
ENSG00000171298	*GAA*	25	52	43	Well expressed	Well expressed	Well expressed
ENSG00000196743	*GM2A*	25	39	26	Well expressed	Well expressed	Well expressed
ENSG00000159079	*CFAP298*	25	28	5	Well expressed	Well expressed	Low expressed
ENSG00000182220	*ATP6AP2*	24	172	38	Well expressed	Well expressed	Well expressed
ENSG00000115170	*ACVR1*	27	43	2	Well expressed	Well expressed	Low expressed
ENSG00000140443	*IGF1R*	26	18	9	Well expressed	Well expressed	Low expressed
ENSG00000168137	*SETD5*	25	42	11	Well expressed	Well expressed	Well expressed
ENSG00000134574	*DDB2*	24	41	10	Well expressed	Well expressed	Well expressed
ENSG00000107960	*STN1*	23	27	14	Well expressed	Well expressed	Well expressed
ENSG00000135317	*SNX14*	25	34	6	Well expressed	Well expressed	Low expressed
ENSG00000186184	*POLR1D*	26	23	12	Well expressed	Well expressed	Well expressed
ENSG00000134759	*ELP2*	25	29	3	Well expressed	Well expressed	Low expressed
ENSG00000066084	*DIP2B*	23	14	10	Well expressed	Well expressed	Well expressed
ENSG00000138175	*ARL3*	23	26	3	Well expressed	Well expressed	Low expressed
ENSG00000130449	*ZSWIM6*	25	12	10	Well expressed	Well expressed	Low expressed
ENSG00000069248	*NUP133*	25	31	4	Well expressed	Well expressed	Low expressed
ENSG00000100014	*SPECC1L*	26	35	6	Well expressed	Well expressed	Low expressed
ENSG00000175283	*DOLK*	25	32	2	Well expressed	Well expressed	Low expressed
ENSG00000127948	*POR*	24	36	62	Well expressed	Well expressed	Well expressed
ENSG00000111670	*GNPTAB*	20	17	5	Well expressed	Well expressed	Low expressed
ENSG00000141458	*NPC1*	23	32	4	Well expressed	Well expressed	Low expressed
ENSG00000039650	*PNKP*	25	25	25	Well expressed	Well expressed	Well expressed
ENSG00000183098	*GPC6*	24	17	0	Well expressed	Well expressed	Not expressed
ENSG00000133884	*DPF2*	25	45	27	Well expressed	Well expressed	Well expressed
ENSG00000165102	*HGSNAT*	23	41	14	Well expressed	Well expressed	Well expressed
ENSG00000011295	*TTC19*	25	26	3	Well expressed	Well expressed	Low expressed
ENSG00000137074	*APTX*	24	29	4	Well expressed	Well expressed	Low expressed
ENSG00000164597	*COG5*	24	17	4	Well expressed	Well expressed	Low expressed
ENSG00000112531	*QKI*	23	26	7	Well expressed	Well expressed	Low expressed
ENSG00000100393	*EP300*	25	34	11	Well expressed	Well expressed	Well expressed
ENSG00000022267	*FHL1*	15	52	3	Well expressed	Well expressed	Low expressed
ENSG00000166086	*JAM3*	24	60	1	Well expressed	Well expressed	Low expressed
ENSG00000196976	*LAGE3*	24	32	8	Well expressed	Well expressed	Low expressed
ENSG00000169604	*ANTXR1*	22	140	0	Well expressed	Well expressed	Not expressed
ENSG00000147475	*ERLIN2*	24	30	3	Well expressed	Well expressed	Low expressed
ENSG00000076716	*GPC4*	25	3	0	Well expressed	Low expressed	Not expressed
ENSG00000015532	*XYLT2*	24	28	6	Well expressed	Well expressed	Low expressed
ENSG00000110395	*CBL*	24	13	16	Well expressed	Well expressed	Well expressed
ENSG00000150990	*DHX37*	24	19	4	Well expressed	Well expressed	Low expressed
ENSG00000088682	*COQ9*	23	45	9	Well expressed	Well expressed	Low expressed
ENSG00000090581	*GNPTG*	23	72	26	Well expressed	Well expressed	Well expressed
ENSG00000088038	*CNOT3*	23	31	21	Well expressed	Well expressed	Well expressed
ENSG00000151729	*SLC25A4*	23	5	1	Well expressed	Low expressed	Not expressed
ENSG00000177565	*TBL1XR1*	21	37	9	Well expressed	Well expressed	Low expressed
ENSG00000181192	*DHTKD1*	24	13	7	Well expressed	Well expressed	Low expressed
ENSG00000243989	*ACY1*	24	31	3	Well expressed	Well expressed	Low expressed
ENSG00000161904	*LEMD2*	24	39	25	Well expressed	Well expressed	Well expressed
ENSG00000124356	*STAMBP*	23	11	5	Well expressed	Well expressed	Low expressed
ENSG00000172315	*TP53RK*	24	19	4	Well expressed	Well expressed	Low expressed
ENSG00000071539	*TRIP13*	12	17	0	Well expressed	Well expressed	Not expressed
ENSG00000117595	*IRF6*	24	0	0	Well expressed	Not expressed	Not expressed
ENSG00000213614	*HEXA*	23	40	10	Well expressed	Well expressed	Well expressed
ENSG00000073584	*SMARCE1*	23	48	7	Well expressed	Well expressed	Low expressed
ENSG00000124198	*ARFGEF2*	23	24	2	Well expressed	Well expressed	Low expressed
ENSG00000155850	*SLC26A2*	20	12	1	Well expressed	Well expressed	Not expressed
ENSG00000102100	*SLC35A2*	23	22	6	Well expressed	Well expressed	Low expressed
ENSG00000001497	*LAS1L*	22	21	4	Well expressed	Well expressed	Low expressed
ENSG00000134049	*IER3IP1*	22	41	2	Well expressed	Well expressed	Low expressed
ENSG00000164134	*NAA15*	23	24	2	Well expressed	Well expressed	Low expressed
ENSG00000133835	*HSD17B4*	22	79	17	Well expressed	Well expressed	Well expressed
ENSG00000160551	*TAOK1*	22	32	5	Well expressed	Well expressed	Low expressed
ENSG00000099940	*SNAP29*	22	28	11	Well expressed	Well expressed	Well expressed
ENSG00000101997	*CCDC22*	23	16	19	Well expressed	Well expressed	Well expressed
ENSG00000103671	*TRIP4*	22	23	12	Well expressed	Well expressed	Well expressed
ENSG00000117713	*ARID1A*	22	25	11	Well expressed	Well expressed	Well expressed
ENSG00000184634	*MED12*	23	19	14	Well expressed	Well expressed	Well expressed
ENSG00000070814	*TCOF1*	22	37	6	Well expressed	Well expressed	Low expressed
ENSG00000077152	*UBE2T*	15	29	2	Well expressed	Well expressed	Low expressed
ENSG00000210196	*MT-TP*	18	1	0	Well expressed	Not expressed	Not expressed
ENSG00000183421	*RIPK4*	21	0	0	Well expressed	Not expressed	Not expressed
ENSG00000171456	*ASXL1*	22	40	8	Well expressed	Well expressed	Low expressed
ENSG00000008441	*NFIX*	18	89	6	Well expressed	Well expressed	Low expressed
ENSG00000101266	*CSNK2A1*	23	61	8	Well expressed	Well expressed	Low expressed
ENSG00000083720	*OXCT1*	20	41	2	Well expressed	Well expressed	Low expressed
ENSG00000197601	*FAR1*	20	36	13	Well expressed	Well expressed	Well expressed
ENSG00000005073	*HOXA11*	21	20	0	Well expressed	Well expressed	Not expressed
ENSG00000143622	*RIT1*	22	16	28	Well expressed	Well expressed	Well expressed
ENSG00000141627	*DYM*	22	20	7	Well expressed	Well expressed	Low expressed
ENSG00000103449	*SALL1*	21	5	0	Well expressed	Low expressed	Not expressed
ENSG00000213380	*COG8*	22	17	6	Well expressed	Well expressed	Low expressed
ENSG00000116353	*MECR*	22	16	1	Well expressed	Well expressed	Low expressed
ENSG00000135341	*MAP3K7*	22	30	4	Well expressed	Well expressed	Low expressed
ENSG00000182287	*AP1S2*	21	10	8	Well expressed	Well expressed	Low expressed
ENSG00000128602	*SMO*	22	9	0	Well expressed	Low expressed	Not expressed
ENSG00000173540	*GMPPB*	20	22	7	Well expressed	Well expressed	Low expressed
ENSG00000125845	*BMP2*	21	1	0	Well expressed	Not expressed	Not expressed
ENSG00000072840	*EVC*	20	41	0	Well expressed	Well expressed	Not expressed

ENSG00000036054	*TBC1D23*	21	36	5	Well expressed	Well expressed	Low expressed
ENSG00000095787	*WAC*	21	51	30	Well expressed	Well expressed	Well expressed
ENSG00000172922	*RNASEH2C*	22	28	16	Well expressed	Well expressed	Well expressed
ENSG00000140650	*PMM2*	22	31	2	Well expressed	Well expressed	Low expressed
ENSG00000108479	*GALK1*	22	27	21	Well expressed	Well expressed	Well expressed
ENSG00000062038	*CDH3*	16	0	0	Well expressed	Not expressed	Not expressed
ENSG00000181555	*SETD2*	20	26	9	Well expressed	Well expressed	Low expressed
ENSG00000164751	*PEX2*	20	17	4	Well expressed	Well expressed	Low expressed
ENSG00000148606	*POLR3A*	21	15	2	Well expressed	Well expressed	Low expressed
ENSG00000155906	*RMND1*	20	34	2	Well expressed	Well expressed	Low expressed
ENSG00000163719	*MTMR14*	22	23	41	Well expressed	Well expressed	Well expressed
ENSG00000198951	*NAGA*	22	33	23	Well expressed	Well expressed	Well expressed
ENSG00000142599	*RERE*	22	33	17	Well expressed	Well expressed	Well expressed
ENSG00000112367	*FIG4*	20	14	10	Well expressed	Well expressed	Well expressed
ENSG00000221838	*AP4M1*	22	15	3	Well expressed	Well expressed	Low expressed
ENSG00000165671	*NSD1*	21	19	6	Well expressed	Well expressed	Low expressed
ENSG00000133703	*KRAS*	20	17	9	Well expressed	Well expressed	Low expressed
ENSG00000140694	*PARN*	21	32	7	Well expressed	Well expressed	Low expressed
ENSG00000166250	*CLMP*	20	152	0	Well expressed	Well expressed	Not expressed
ENSG00000115204	*MPV17*	20	57	9	Well expressed	Well expressed	Low expressed
ENSG00000101972	*STAG2*	20	37	17	Well expressed	Well expressed	Well expressed
ENSG00000062598	*ELMO2*	21	17	22	Well expressed	Well expressed	Well expressed
ENSG00000099956	*SMARCB1*	22	78	19	Well expressed	Well expressed	Well expressed
ENSG00000114251	*WNT5A*	18	128	0	Well expressed	Well expressed	Not expressed
ENSG00000100726	*TELO2*	20	32	10	Well expressed	Well expressed	Low expressed
ENSG00000120008	*WDR11*	21	26	3	Well expressed	Well expressed	Low expressed
ENSG00000116062	*MSH6*	20	20	2	Well expressed	Well expressed	Low expressed
ENSG00000074582	*BCS1L*	21	42	5	Well expressed	Well expressed	Low expressed
ENSG00000164692	*COL1A2*	37	4674	1	Well expressed	Well expressed	Not expressed
ENSG00000135249	*RINT1*	20	23	3	Well expressed	Well expressed	Low expressed
ENSG00000138081	*FBXO11*	20	38	7	Well expressed	Well expressed	Low expressed
ENSG00000161533	*ACOX1*	20	15	20	Well expressed	Well expressed	Well expressed
ENSG00000185115	*NSMCE3*	20	11	3	Well expressed	Well expressed	Low expressed
ENSG00000099949	*LZTR1*	20	34	11	Well expressed	Well expressed	Well expressed
ENSG00000244486	*SCARF2*	20	45	1	Well expressed	Well expressed	Not expressed
ENSG00000141556	*TBCD*	20	20	6	Well expressed	Well expressed	Low expressed
ENSG00000138375	*SMARCAL1*	21	25	6	Well expressed	Well expressed	Low expressed
ENSG00000079616	*KIF22*	12	19	10	Well expressed	Well expressed	Low expressed
ENSG00000108557	*RAI1*	20	16	1	Well expressed	Well expressed	Low expressed
ENSG00000178802	*MPI*	20	23	4	Well expressed	Well expressed	Low expressed
ENSG00000132510	*KDM6B*	21	8	64	Well expressed	Low expressed	Well expressed
ENSG00000117899	*MESD*	21	35	3	Well expressed	Well expressed	Low expressed
ENSG00000164818	*DNAAF5*	20	23	1	Well expressed	Well expressed	Low expressed
ENSG00000031081	*ARHGAP31*	20	13	1	Well expressed	Well expressed	Low expressed
ENSG00000173757	*STAT5B*	21	30	139	Well expressed	Well expressed	Well expressed
ENSG00000109846	*CRYAB*	11	70	1	Well expressed	Well expressed	Low expressed
ENSG00000159063	*ALG8*	19	29	3	Well expressed	Well expressed	Low expressed
ENSG00000101938	*CHRDL1*	15	4	0	Well expressed	Low expressed	Not expressed
ENSG00000101464	*PIGU*	19	31	4	Well expressed	Well expressed	Low expressed
ENSG00000144647	*POMGNT2*	19	23	3	Well expressed	Well expressed	Low expressed
ENSG00000110435	*PDHX*	19	25	2	Well expressed	Well expressed	Low expressed
ENSG00000118971	*CCND2*	15	10	8	Well expressed	Low expressed	Low expressed
ENSG00000172269	*DPAGT1*	19	50	8	Well expressed	Well expressed	Low expressed
ENSG00000117020	*AKT3*	18	23	1	Well expressed	Well expressed	Low expressed
ENSG00000138035	*PNPT1*	20	26	1	Well expressed	Well expressed	Low expressed
ENSG00000100207	*TCF20*	19	15	5	Well expressed	Well expressed	Low expressed
ENSG00000196367	*TRRAP*	19	25	3	Well expressed	Well expressed	Low expressed
ENSG00000174720	*LARP7*	18	42	5	Well expressed	Well expressed	Low expressed
ENSG00000033011	*ALG1*	19	31	4	Well expressed	Well expressed	Low expressed
ENSG00000102743	*SLC25A15*	19	14	1	Well expressed	Well expressed	Not expressed
ENSG00000255072	*PIGY*	19	No data	No data	Well expressed	No data	No data
ENSG00000138303	*ASCC1*	19	19	6	Well expressed	Well expressed	Low expressed
ENSG00000244045	*TMEM199*	20	14	7	Well expressed	Well expressed	Low expressed
ENSG00000205138	*SDHAF1*	20	20	10	Well expressed	Well expressed	Low expressed
ENSG00000012963	*UBR7*	16	26	6	Well expressed	Well expressed	Low expressed
ENSG00000153015	*CWC27*	19	30	3	Well expressed	Well expressed	Low expressed
ENSG00000117305	*HMGCL*	20	25	7	Well expressed	Well expressed	Low expressed
ENSG00000133422	*MORC2*	19	30	5	Well expressed	Well expressed	Low expressed
ENSG00000138829	*FBN2*	19	127	1	Well expressed	Well expressed	Not expressed
ENSG00000152223	*EPG5*	18	27	5	Well expressed	Well expressed	Low expressed
ENSG00000159899	*NPR2*	19	39	0	Well expressed	Well expressed	Not expressed
ENSG00000167323	*STIM1*	18	23	26	Well expressed	Well expressed	Well expressed
ENSG00000138834	*MAPK8IP3*	17	49	43	Well expressed	Well expressed	Well expressed
ENSG00000117139	*KDM5B*	18	25	7	Well expressed	Well expressed	Low expressed
ENSG00000036257	*CUL3*	18	25	8	Well expressed	Well expressed	Low expressed
ENSG00000163161	*ERCC3*	18	51	12	Well expressed	Well expressed	Well expressed
ENSG00000134899	*ERCC5*	18	3	1	Well expressed	Low expressed	Not expressed
ENSG00000143363	*PRUNE1*	19	11	9	Well expressed	Well expressed	Low expressed
ENSG00000174792	*ODAPH*	16	0	0	Well expressed	Not expressed	Not expressed
ENSG00000103043	*VAC14*	19	19	6	Well expressed	Well expressed	Low expressed
ENSG00000108797	*CNTNAP1*	17	32	0	Well expressed	Well expressed	Not expressed
ENSG00000102302	*FGD1*	18	18	0	Well expressed	Well expressed	Not expressed
ENSG00000138078	*PREPL*	16	22	1	Well expressed	Well expressed	Low expressed
ENSG00000188690	*UROS*	18	21	3	Well expressed	Well expressed	Low expressed
ENSG00000160193	*WDR4*	17	19	2	Well expressed	Well expressed	Low expressed
ENSG00000077782	*FGFR1*	18	93	2	Well expressed	Well expressed	Low expressed
ENSG00000062822	*POLD1*	15	15	7	Well expressed	Well expressed	Low expressed
ENSG00000114062	*UBE3A*	17	20	1	Well expressed	Well expressed	Low expressed
ENSG00000167113	*COQ4*	18	22	5	Well expressed	Well expressed	Low expressed
ENSG00000169919	*GUSB*	18	57	34	Well expressed	Well expressed	Well expressed
ENSG00000073614	*KDM5A*	18	20	11	Well expressed	Well expressed	Well expressed
ENSG00000198824	*CHAMP1*	18	41	2	Well expressed	Well expressed	Low expressed
ENSG00000122971	*ACADS*	18	14	10	Well expressed	Well expressed	Well expressed

ENSG00000198589	*LRBA*	19	12	1	Well expressed	Well expressed	Low expressed
ENSG00000177542	*SLC25A22*	17	27	4	Well expressed	Well expressed	Low expressed
ENSG00000150787	*PTS*	17	31	1	Well expressed	Well expressed	Low expressed
ENSG00000107249	*GLIS3*	17	7	0	Well expressed	Low expressed	Not expressed
ENSG00000139197	*PEX5*	19	27	4	Well expressed	Well expressed	Low expressed
ENSG00000105379	*ETFB*	18	41	11	Well expressed	Well expressed	Well expressed
ENSG00000081087	*OSTM1*	15	17	7	Well expressed	Well expressed	Low expressed
ENSG00000230124	*ACBD6*	18	21	5	Well expressed	Well expressed	Low expressed
ENSG00000179364	*PACS2*	18	20	5	Well expressed	Well expressed	Low expressed
ENSG00000136425	*CIB2*	18	2	1	Well expressed	Low expressed	Not expressed
ENSG00000153107	*ANAPC1*	18	12	1	Well expressed	Well expressed	Not expressed
ENSG00000269335	*IKBKG*	18	7	7	Well expressed	Low expressed	Low expressed
ENSG00000132964	*CDK8*	17	15	2	Well expressed	Well expressed	Low expressed
ENSG00000011376	*LARS2*	18	20	1	Well expressed	Well expressed	Low expressed
ENSG00000103197	*TSC2*	17	34	14	Well expressed	Well expressed	Well expressed
ENSG00000175110	*MRPS22*	17	35	4	Well expressed	Well expressed	Low expressed
ENSG00000143442	*POGZ*	18	31	9	Well expressed	Well expressed	Low expressed
ENSG00000197535	*MYO5A*	17	18	5	Well expressed	Well expressed	Low expressed
ENSG00000126464	*PRR12*	17	18	4	Well expressed	Well expressed	Low expressed
ENSG00000106976	*DNM1*	17	14	0	Well expressed	Well expressed	Not expressed
ENSG00000118873	*RAB3GAP2*	16	20	3	Well expressed	Well expressed	Low expressed
ENSG00000189369	*GSPT2*	16	17	1	Well expressed	Well expressed	Not expressed
ENSG00000163638	*ADAMTS9*	15	1	0	Well expressed	Low expressed	Not expressed
ENSG00000040531	*CTNS*	14	16	7	Well expressed	Well expressed	Low expressed
ENSG00000112210	*RAB23*	16	46	0	Well expressed	Well expressed	Not expressed
ENSG00000108510	*MED13*	15	21	5	Well expressed	Well expressed	Low expressed
ENSG00000099381	*SETD1A*	17	15	4	Well expressed	Well expressed	Low expressed
ENSG00000167548	*KMT2D*	16	19	8	Well expressed	Well expressed	Low expressed
ENSG00000082996	*RNF13*	15	30	42	Well expressed	Well expressed	Well expressed
ENSG00000168538	*TRAPPC11*	17	29	7	Well expressed	Well expressed	Low expressed
ENSG00000064313	*TAF2*	17	26	2	Well expressed	Well expressed	Low expressed
ENSG00000038002	*AGA*	17	20	7	Well expressed	Well expressed	Low expressed
ENSG00000141448	*GATA6*	17	1	0	Well expressed	Not expressed	Not expressed
ENSG00000144034	*TPRKB*	16	24	3	Well expressed	Well expressed	Low expressed
ENSG00000132470	*ITGB4*	13	1	1	Well expressed	Not expressed	Not expressed
ENSG00000171453	*POLR1C*	17	38	4	Well expressed	Well expressed	Low expressed
ENSG00000173575	*CHD2*	15	29	11	Well expressed	Well expressed	Well expressed
ENSG00000156531	*PHF6*	17	14	1	Well expressed	Well expressed	Not expressed
ENSG00000169855	*ROBO1*	15	25	0	Well expressed	Well expressed	Not expressed
ENSG00000005483	*KMT2E*	15	27	13	Well expressed	Well expressed	Well expressed
ENSG00000164172	*MOCS2*	15	22	1	Well expressed	Well expressed	Low expressed
ENSG00000160271	*RALGDS*	16	15	18	Well expressed	Well expressed	Well expressed
ENSG00000146085	*MMUT*	16	33	3	Well expressed	Well expressed	Low expressed
ENSG00000104889	*RNASEH2A*	12	45	8	Well expressed	Well expressed	Low expressed
ENSG00000124813	*RUNX2*	15	19	3	Well expressed	Well expressed	Low expressed
ENSG00000169118	*CSNK1G1*	16	11	2	Well expressed	Well expressed	Low expressed
ENSG00000124006	*OBSL1*	16	22	0	Well expressed	Well expressed	Not expressed
ENSG00000171862	*PTEN*	15	25	40	Well expressed	Well expressed	Well expressed
ENSG00000156970	*BUB1B*	8	16	0	Low expressed	Well expressed	Not expressed
ENSG00000106462	*EZH2*	14	12	2	Well expressed	Well expressed	Low expressed
ENSG00000187098	*MITF*	14	8	0	Well expressed	Low expressed	Not expressed
ENSG00000027847	*B4GALT7*	16	41	9	Well expressed	Well expressed	Low expressed
ENSG00000112290	*WASF1*	17	37	1	Well expressed	Well expressed	Not expressed
ENSG00000185324	*CDK10*	16	25	9	Well expressed	Well expressed	Low expressed
ENSG00000177189	*RPS6KA3*	16	27	17	Well expressed	Well expressed	Well expressed
ENSG00000126883	*NUP214*	17	21	19	Well expressed	Well expressed	Well expressed
ENSG00000101986	*ABCD1*	16	10	11	Well expressed	Well expressed	Well expressed
ENSG00000091127	*PUS7*	17	17	0	Well expressed	Well expressed	Not expressed
ENSG00000006695	*COX10*	17	12	3	Well expressed	Well expressed	Low expressed
ENSG00000167306	*MYO5B*	15	0	0	Well expressed	Not expressed	Not expressed
ENSG00000124587	*PEX6*	14	26	12	Well expressed	Well expressed	Well expressed
ENSG00000100888	*CHD8*	16	28	9	Well expressed	Well expressed	Low expressed
ENSG00000112312	*GMNN*	10	18	2	Well expressed	Well expressed	Low expressed
ENSG00000065135	*GNAI3*	16	15	8	Well expressed	Well expressed	Low expressed
ENSG00000113721	*PDGFRB*	12	257	1	Well expressed	Well expressed	Low expressed
ENSG00000067836	*ROGDI*	15	8	33	Well expressed	Low expressed	Well expressed
ENSG00000172409	*CLP1*	16	20	7	Well expressed	Well expressed	Low expressed
ENSG00000103126	*AXIN1*	16	23	27	Well expressed	Well expressed	Well expressed
ENSG00000166548	*TK2*	15	26	7	Well expressed	Well expressed	Low expressed
ENSG00000107371	*EXOSC3*	15	21	5	Well expressed	Well expressed	Low expressed
ENSG00000113522	*RAD50*	15	28	1	Well expressed	Well expressed	Low expressed
ENSG00000144455	*SUMF1*	16	21	5	Well expressed	Well expressed	Low expressed
ENSG00000147100	*SLC16A2*	15	27	0	Well expressed	Well expressed	Not expressed
ENSG00000134313	*KIDINS220*	14	20	5	Well expressed	Well expressed	Low expressed
ENSG00000166974	*MAPRE2*	14	14	12	Well expressed	Well expressed	Well expressed
ENSG00000183161	*FANCF*	15	6	1	Well expressed	Low expressed	Low expressed
ENSG00000147050	*KDM6A*	15	8	7	Well expressed	Low expressed	Low expressed
ENSG00000185000	*DGAT1*	15	20	41	Well expressed	Well expressed	Well expressed
ENSG00000138286	*FAM149B1*	14	17	2	Well expressed	Well expressed	Low expressed
ENSG00000103876	*FAH*	15	19	4	Well expressed	Well expressed	Low expressed
ENSG00000148985	*PGAP2*	15	16	8	Well expressed	Well expressed	Low expressed
ENSG00000134905	*CARS2*	15	50	25	Well expressed	Well expressed	Well expressed
ENSG00000104133	*SPG11*	14	23	13	Well expressed	Well expressed	Well expressed
ENSG00000125676	*THOC2*	13	39	3	Well expressed	Well expressed	Low expressed
ENSG00000117593	*DARS2*	14	28	2	Well expressed	Well expressed	Low expressed
ENSG00000115286	*NDUFS7*	15	30	17	Well expressed	Well expressed	Well expressed
ENSG00000168827	*GFM1*	16	32	3	Well expressed	Well expressed	Low expressed
ENSG00000104763	*ASAH1*	14	37	82	Well expressed	Well expressed	Well expressed
ENSG00000149418	*ST14*	11	0	10	Well expressed	Not expressed	Low expressed
ENSG00000117724	*CENPF*	7	17	0	Low expressed	Well expressed	Not expressed
ENSG00000167625	*ZNF526*	16	12	3	Well expressed	Well expressed	Low expressed
ENSG00000143614	*GATAD2B*	16	9	15	Well expressed	Low expressed	Well expressed
ENSG00000143631	*FLG*	6	1	0	Low expressed	Not expressed	Not expressed

ENSG00000163625	*WDFY3*	13	18	10	Well expressed	Well expressed	Low expressed
ENSG00000105607	*GCDH*	15	18	6	Well expressed	Well expressed	Low expressed
ENSG00000162928	*PEX13*	14	12	2	Well expressed	Well expressed	Low expressed
ENSG00000198198	*SZT2*	15	11	6	Well expressed	Well expressed	Low expressed
ENSG00000123737	*EXOSC9*	13	34	4	Well expressed	Well expressed	Low expressed
ENSG00000138018	*SELENOI*	14	10	1	Well expressed	Low expressed	Not expressed
ENSG00000132376	*INPP5K*	14	21	35	Well expressed	Well expressed	Well expressed
ENSG00000121680	*PEX16*	15	15	14	Well expressed	Well expressed	Well expressed
ENSG00000167513	*CDT1*	10	13	2	Low expressed	Well expressed	Low expressed
ENSG00000135929	*CYP27A1*	12	20	18	Well expressed	Well expressed	Well expressed
ENSG00000113273	*ARSB*	13	13	2	Well expressed	Well expressed	Low expressed
ENSG00000215193	*PEX26*	15	8	2	Well expressed	Low expressed	Low expressed
ENSG00000102974	*CTCF*	15	35	14	Well expressed	Well expressed	Well expressed
ENSG00000178691	*SUZ12*	13	29	6	Well expressed	Well expressed	Low expressed
ENSG00000145354	*CISD2*	14	21	5	Well expressed	Well expressed	Low expressed
ENSG00000116580	*GON4L*	15	14	4	Well expressed	Well expressed	Low expressed
ENSG00000106144	*CASP2*	14	20	14	Well expressed	Well expressed	Well expressed
ENSG00000116198	*CEP104*	15	12	3	Well expressed	Well expressed	Low expressed
ENSG00000163933	*RFT1*	14	20	4	Well expressed	Well expressed	Low expressed
ENSG00000164087	*POC1A*	10	12	2	Low expressed	Well expressed	Low expressed
ENSG00000132669	*RIN2*	12	31	2	Well expressed	Well expressed	Low expressed
ENSG00000196517	*SLC6A9*	15	7	1	Well expressed	Low expressed	Low expressed
ENSG00000117054	*ACADM*	14	27	3	Well expressed	Well expressed	Low expressed
ENSG00000060069	*CTDP1*	15	11	17	Well expressed	Well expressed	Well expressed
ENSG00000178919	*FOXE1*	10	0	0	Low expressed	Not expressed	Not expressed
ENSG00000104833	*TUBB4A*	10	0	0	Well expressed	Not expressed	Not expressed
ENSG00000269900	*RMRP*	4	0	0	Low expressed	Not expressed	Not expressed
ENSG00000182858	*ALG12*	14	12	5	Well expressed	Well expressed	Low expressed
ENSG00000115392	*FANCL*	14	14	1	Well expressed	Well expressed	Not expressed
ENSG00000003393	*ALS2*	13	16	1	Well expressed	Well expressed	Not expressed
ENSG00000109929	*SC5D*	12	26	3	Well expressed	Well expressed	Low expressed
ENSG00000007541	*PIGQ*	15	13	8	Well expressed	Well expressed	Low expressed
ENSG00000155755	*TMEM237*	11	18	0	Well expressed	Well expressed	Not expressed
ENSG00000072864	*NDE1*	13	15	17	Well expressed	Well expressed	Well expressed
ENSG00000184056	*VPS33B*	14	20	8	Well expressed	Well expressed	Low expressed
ENSG00000136710	*CCDC115*	13	36	18	Well expressed	Well expressed	Well expressed
ENSG00000167716	*WDR81*	13	22	7	Well expressed	Well expressed	Low expressed
ENSG00000179456	*ZBTB18*	14	8	16	Well expressed	Low expressed	Well expressed
ENSG00000101871	*MID1*	10	8	0	Well expressed	Low expressed	Not expressed
ENSG00000173218	*VANGL1*	14	13	0	Well expressed	Well expressed	Not expressed
ENSG00000099377	*HSD3B7*	13	47	6	Well expressed	Well expressed	Low expressed
ENSG00000156983	*BRPF1*	14	14	6	Well expressed	Well expressed	Low expressed
ENSG00000108641	*B9D1*	13	10	0	Well expressed	Low expressed	Not expressed
ENSG00000136854	*STXBP1*	12	16	0	Well expressed	Well expressed	Not expressed
ENSG00000154134	*ROBO3*	14	7	1	Well expressed	Low expressed	Not expressed
ENSG00000164414	*SLC35A1*	12	31	9	Well expressed	Well expressed	Low expressed
ENSG00000166068	*SPRED1*	13	27	0	Well expressed	Well expressed	Not expressed
ENSG00000171634	*BPTF*	12	21	4	Well expressed	Well expressed	Low expressed
ENSG00000100749	*VRK1*	11	13	4	Well expressed	Well expressed	Low expressed
ENSG00000124155	*PIGT*	14	35	9	Well expressed	Well expressed	Low expressed
ENSG00000147852	*VLDLR*	13	5	0	Well expressed	Low expressed	Not expressed
ENSG00000138160	*KIF11*	5	18	1	Low expressed	Well expressed	Not expressed
ENSG00000133059	*DSTYK*	14	15	2	Well expressed	Well expressed	Low expressed
ENSG00000087266	*SH3BP2*	13	12	53	Well expressed	Well expressed	Well expressed
ENSG00000187736	*NHEJ1*	14	13	4	Well expressed	Well expressed	Low expressed
ENSG00000175606	*TMEM70*	13	25	8	Well expressed	Well expressed	Low expressed
ENSG00000105048	*TNNT1*	13	3	3	Well expressed	Low expressed	Low expressed
ENSG00000138771	*SHROOM3*	13	3	0	Well expressed	Low expressed	Not expressed
ENSG00000122512	*PMS2*	14	15	2	Well expressed	Well expressed	Low expressed
ENSG00000176887	*SOX11*	13	0	0	Well expressed	Not expressed	Not expressed
ENSG00000272333	*KMT2B*	14	20	21	Well expressed	Well expressed	Well expressed
ENSG00000172071	*EIF2AK3*	11	11	2	Well expressed	Well expressed	Low expressed
ENSG00000039068	*CDH1*	10	0	0	Low expressed	Not expressed	Not expressed
ENSG00000151148	*UBE3B*	13	22	5	Well expressed	Well expressed	Low expressed
ENSG00000177045	*SIX5*	13	15	0	Well expressed	Well expressed	Not expressed
ENSG00000196116	*TDRD7*	13	9	7	Well expressed	Low expressed	Low expressed
ENSG00000083123	*BCKDHB*	14	14	1	Well expressed	Well expressed	Not expressed
ENSG00000107882	*SUFU*	14	11	3	Well expressed	Well expressed	Low expressed
ENSG00000131979	*GCH1*	13	3	11	Well expressed	Low expressed	Well expressed
ENSG00000122550	*KLHL7*	14	14	2	Well expressed	Well expressed	Low expressed
ENSG00000111817	*DSE*	13	45	4	Well expressed	Well expressed	Low expressed
ENSG00000141252	*VPS53*	13	7	3	Well expressed	Low expressed	Low expressed
ENSG00000139324	*TMTC3*	10	22	0	Well expressed	Well expressed	Not expressed
ENSG00000119878	*CRIPT*	13	21	6	Well expressed	Well expressed	Low expressed
ENSG00000005339	*CREBBP*	14	24	16	Well expressed	Well expressed	Well expressed
ENSG00000034693	*PEX3*	12	23	1	Well expressed	Well expressed	Low expressed
ENSG00000141646	*SMAD4*	13	24	5	Well expressed	Well expressed	Low expressed
ENSG00000116984	*MTR*	12	20	1	Well expressed	Well expressed	Not expressed
ENSG00000174106	*LEMD3*	13	19	4	Well expressed	Well expressed	Low expressed
ENSG00000181027	*FKRP*	13	18	3	Well expressed	Well expressed	Low expressed
ENSG00000114491	*UMPS*	12	16	1	Well expressed	Well expressed	Low expressed
ENSG00000115904	*SOS1*	12	19	3	Well expressed	Well expressed	Low expressed
ENSG00000101347	*SAMHD1*	12	44	45	Well expressed	Well expressed	Well expressed
ENSG00000136861	*CDK5RAP2*	13	32	20	Well expressed	Well expressed	Well expressed
ENSG00000107829	*FBXW4*	13	15	10	Well expressed	Well expressed	Low expressed
ENSG00000125378	*BMP4*	6	0	0	Low expressed	Not expressed	Not expressed
ENSG00000183513	*COA5*	13	27	7	Well expressed	Well expressed	Low expressed
ENSG00000118007	*STAG1*	12	30	5	Well expressed	Well expressed	Low expressed
ENSG00000174405	*LIG4*	11	13	2	Well expressed	Well expressed	Low expressed
ENSG00000121152	*NCAPH*	5	10	1	Low expressed	Well expressed	Not expressed
ENSG00000118162	*KPTN*	13	11	4	Well expressed	Well expressed	Low expressed
ENSG00000130714	*POMT1*	13	14	4	Well expressed	Well expressed	Low expressed
ENSG00000166037	*CEP57*	13	27	4	Well expressed	Well expressed	Low expressed

ENSG00000169359	*SLC33A1*	12	14	1	Well expressed	Well expressed	Low expressed
ENSG00000090889	*KIF4A*	5	12	0	Low expressed	Well expressed	Not expressed
ENSG00000008710	*PKD1*	12	35	2	Well expressed	Well expressed	Low expressed
ENSG00000116539	*ASH1L*	12	22	3	Well expressed	Well expressed	Low expressed
ENSG00000088305	*DNMT3B*	13	3	0	Well expressed	Low expressed	Not expressed
ENSG00000047056	*WDR37*	13	16	11	Well expressed	Well expressed	Well expressed
ENSG00000119650	*IFT43*	13	19	1	Well expressed	Well expressed	Low expressed
ENSG00000164190	*NIPBL*	11	21	8	Well expressed	Well expressed	Low expressed
ENSG00000168434	*COG7*	12	17	3	Well expressed	Well expressed	Low expressed
ENSG00000198914	*POU3F3*	12	15	0	Well expressed	Well expressed	Not expressed
ENSG00000118058	*KMT2A*	12	16	2	Well expressed	Well expressed	Low expressed
ENSG00000085224	*ATRX*	11	18	3	Well expressed	Well expressed	Low expressed
ENSG00000121350	*PYROXD1*	11	11	2	Well expressed	Well expressed	Low expressed
ENSG00000147044	*CASK*	13	9	1	Well expressed	Low expressed	Not expressed
ENSG00000139718	*SETD1B*	12	15	8	Well expressed	Well expressed	Low expressed
ENSG00000164904	*ALDH7A1*	12	22	0	Well expressed	Well expressed	Not expressed
ENSG00000075891	*PAX2*	12	0	0	Well expressed	Not expressed	Not expressed
ENSG00000138061	*CYP1B1*	10	63	10	Well expressed	Well expressed	Low expressed
ENSG00000111581	*NUP107*	12	19	2	Well expressed	Well expressed	Low expressed
ENSG00000107186	*MPDZ*	11	22	0	Well expressed	Well expressed	Not expressed
ENSG00000197238	*H4C11*	13	0	0	Well expressed	Not expressed	Not expressed
ENSG00000169375	*SIN3A*	13	18	6	Well expressed	Well expressed	Low expressed
ENSG00000009830	*POMT2*	12	14	1	Well expressed	Well expressed	Low expressed
ENSG00000171503	*ETFDH*	12	20	4	Well expressed	Well expressed	Low expressed
ENSG00000163170	*BOLA3*	11	29	1	Well expressed	Well expressed	Low expressed
ENSG00000132031	*MATN3*	13	5	0	Well expressed	Low expressed	Not expressed
ENSG00000181523	*SGSH*	11	45	23	Well expressed	Well expressed	Well expressed
ENSG00000094804	*CDC6*	7	9	1	Low expressed	Low expressed	Not expressed
ENSG00000122591	*FAM126A*	12	26	1	Well expressed	Well expressed	Not expressed
ENSG00000078070	*MCCC1*	12	21	2	Well expressed	Well expressed	Low expressed
ENSG00000185339	*TCN2*	10	9	17	Well expressed	Low expressed	Well expressed
ENSG00000196712	*NF1*	12	18	2	Well expressed	Well expressed	Low expressed
ENSG00000173085	*COQ2*	12	16	4	Well expressed	Well expressed	Low expressed
ENSG00000095015	*MAP3K1*	11	4	12	Well expressed	Low expressed	Well expressed
ENSG00000085788	*DDHD2*	12	19	2	Well expressed	Well expressed	Low expressed
ENSG00000081307	*UBA5*	12	32	2	Well expressed	Well expressed	Low expressed
ENSG00000159459	*UBR1*	12	13	2	Well expressed	Well expressed	Low expressed
ENSG00000112282	*MED23*	11	18	4	Well expressed	Well expressed	Low expressed
ENSG00000133028	*SCO1*	12	14	2	Well expressed	Well expressed	Low expressed
ENSG00000090674	*MCOLN1*	11	25	62	Well expressed	Well expressed	Well expressed
ENSG00000010818	*HIVEP2*	12	13	2	Well expressed	Well expressed	Low expressed
ENSG00000173226	*IQCB1*	11	18	6	Well expressed	Well expressed	Low expressed
ENSG00000198860	*TSEN15*	11	51	4	Well expressed	Well expressed	Low expressed
ENSG00000213930	*GALT*	12	25	11	Well expressed	Well expressed	Well expressed
ENSG00000128159	*TUBGCP6*	12	23	20	Well expressed	Well expressed	Well expressed
ENSG00000101596	*SMCHD1*	11	23	31	Well expressed	Well expressed	Well expressed
ENSG00000170836	*PPM1D*	11	8	4	Well expressed	Low expressed	Low expressed
ENSG00000140525	*FANCI*	7	14	2	Low expressed	Well expressed	Low expressed
ENSG00000179941	*BBS10*	11	21	1	Well expressed	Well expressed	Not expressed
ENSG00000196663	*TECPR2*	12	11	20	Well expressed	Well expressed	Well expressed
ENSG00000148218	*ALAD*	11	28	9	Well expressed	Well expressed	Low expressed
ENSG00000143970	*ASXL2*	11	14	3	Well expressed	Well expressed	Low expressed
ENSG00000126705	*AHDC1*	12	12	1	Well expressed	Well expressed	Low expressed
ENSG00000070018	*LRP6*	10	11	0	Well expressed	Well expressed	Not expressed
ENSG00000165617	*DACT1*	12	1	0	Well expressed	Low expressed	Not expressed
ENSG00000110074	*FOXRED1*	11	22	8	Well expressed	Well expressed	Low expressed
ENSG00000171853	*TRAPPC12*	12	20	9	Well expressed	Well expressed	Low expressed
ENSG00000130158	*DOCK6*	12	12	0	Well expressed	Well expressed	Not expressed
ENSG00000054282	*SDCCAG8*	11	12	3	Well expressed	Well expressed	Low expressed
ENSG00000146872	*TLK2*	12	20	6	Well expressed	Well expressed	Low expressed
ENSG00000183337	*BCOR*	11	10	1	Well expressed	Well expressed	Low expressed
ENSG00000124615	*MOCS1*	11	14	0	Well expressed	Well expressed	Not expressed
ENSG00000154764	*WNT7A*	10	0	0	Low expressed	Not expressed	Not expressed
ENSG00000131269	*ABCB7*	11	19	5	Well expressed	Well expressed	Low expressed
ENSG00000134815	*DHX34*	11	16	85	Well expressed	Well expressed	Well expressed
ENSG00000159267	*HLCS*	11	12	1	Well expressed	Well expressed	Not expressed
ENSG00000146918	*NCAPG2*	6	18	1	Low expressed	Well expressed	Low expressed
ENSG00000154153	*RETREG1*	9	1	2	Low expressed	Not expressed	Low expressed
ENSG00000129696	*TTI2*	11	8	3	Well expressed	Low expressed	Low expressed
ENSG00000177302	*TOP3A*	11	17	12	Well expressed	Well expressed	Well expressed
ENSG00000092094	*OSGEP*	11	22	11	Well expressed	Well expressed	Well expressed
ENSG00000013503	*POLR3B*	11	11	1	Well expressed	Well expressed	Not expressed
ENSG00000258366	*RTEL1*	11	18	5	Well expressed	Well expressed	Low expressed
ENSG00000131931	*THAP1*	11	14	2	Well expressed	Well expressed	Low expressed
ENSG00000161395	*PGAP3*	11	17	6	Well expressed	Well expressed	Low expressed
ENSG00000026297	*RNASET2*	10	18	156	Well expressed	Well expressed	Well expressed
ENSG00000102312	*PORCN*	11	24	5	Well expressed	Well expressed	Low expressed
ENSG00000148400	*NOTCH1*	10	8	24	Well expressed	Low expressed	Well expressed
ENSG00000138686	*BBS7*	11	22	1	Well expressed	Well expressed	Not expressed
ENSG00000174483	*BBS1*	11	16	2	Well expressed	Well expressed	Low expressed
ENSG00000165282	*PIGO*	11	24	4	Well expressed	Well expressed	Low expressed
ENSG00000100815	*TRIP11*	10	20	2	Low expressed	Well expressed	Low expressed
ENSG00000152422	*XRCC4*	8	8	2	Low expressed	Low expressed	Low expressed
ENSG00000155100	*OTUD6B*	10	12	1	Well expressed	Well expressed	Low expressed
ENSG00000137822	*TUBGCP4*	11	11	2	Well expressed	Well expressed	Low expressed
ENSG00000185900	*POMK*	11	6	0	Well expressed	Low expressed	Not expressed
ENSG00000167632	*TRAPPC9*	11	7	7	Well expressed	Low expressed	Low expressed
ENSG00000163378	*EOGT*	10	24	1	Well expressed	Well expressed	Not expressed
ENSG00000140463	*BBS4*	10	17	2	Well expressed	Well expressed	Low expressed
ENSG00000106009	*BRAT1*	11	36	23	Well expressed	Well expressed	Well expressed
ENSG00000151617	*EDNRA*	7	6	0	Low expressed	Low expressed	Not expressed
ENSG00000221829	*FANCG*	8	17	4	Low expressed	Well expressed	Low expressed
ENSG00000125812	*GZF1*	10	11	6	Well expressed	Well expressed	Low expressed

ENSG00000156113	*KCNMA1*	10	1	0	Well expressed	Low expressed	Not expressed
ENSG00000128973	*CLN6*	10	32	11	Low expressed	Well expressed	Well expressed
ENSG00000183597	*TANGO2*	10	12	25	Well expressed	Well expressed	Well expressed
ENSG00000124275	*MTRR*	10	22	4	Well expressed	Well expressed	Low expressed
ENSG00000178445	*GLDC*	11	1	0	Well expressed	Not expressed	Not expressed
ENSG00000132773	*TOE1*	10	16	4	Low expressed	Well expressed	Low expressed
ENSG00000005102	*MEOX1*	5	0	0	Low expressed	Not expressed	Not expressed
ENSG00000164494	*PDSS2*	10	14	2	Well expressed	Well expressed	Low expressed
ENSG00000054983	*GALC*	10	14	8	Well expressed	Well expressed	Low expressed
ENSG00000105983	*LMBR1*	10	10	2	Well expressed	Well expressed	Low expressed
ENSG00000135931	*ARMC9*	10	11	0	Well expressed	Well expressed	Not expressed
ENSG00000213341	*CHUK*	10	12	4	Well expressed	Well expressed	Low expressed
ENSG00000277161	*PIGW*	11	13	1	Well expressed	Well expressed	Not expressed
ENSG00000151503	*NCAPD3*	7	12	1	Low expressed	Well expressed	Low expressed
ENSG00000135111	*TBX3*	7	38	0	Low expressed	Well expressed	Not expressed
ENSG00000105771	*SMG9*	10	12	5	Well expressed	Well expressed	Low expressed
ENSG00000146247	*PHIP*	9	13	4	Low expressed	Well expressed	Low expressed
ENSG00000093009	*CDC45*	4	13	1	Low expressed	Well expressed	Not expressed
ENSG00000253710	*ALG11*	9	4	0	Low expressed	Low expressed	Not expressed
ENSG00000104936	*DMPK*	10	41	5	Low expressed	Well expressed	Low expressed
ENSG00000119688	*ABCD4*	10	17	6	Well expressed	Well expressed	Low expressed
ENSG00000102081	*FMR1*	10	16	5	Low expressed	Well expressed	Low expressed
ENSG00000177728	*TMEM94*	11	23	10	Well expressed	Well expressed	Low expressed
ENSG00000113851	*CRBN*	10	22	9	Well expressed	Well expressed	Low expressed
ENSG00000122643	*NT5C3A*	10	23	16	Well expressed	Well expressed	Well expressed
ENSG00000116641	*DOCK7*	10	18	0	Well expressed	Well expressed	Not expressed
ENSG00000148143	*ZNF462*	9	5	0	Low expressed	Low expressed	Not expressed
ENSG00000284770	*TBCE*	10	32	4	Low expressed	Well expressed	Low expressed
ENSG00000187742	*SECISBP2*	10	12	7	Low expressed	Well expressed	Low expressed
ENSG00000173614	*NMNAT1*	10	11	2	Low expressed	Well expressed	Low expressed
ENSG00000134504	*KCTD1*	10	6	0	Low expressed	Low expressed	Not expressed
ENSG00000145016	*RUBCN*	10	7	9	Well expressed	Low expressed	Low expressed
ENSG00000166575	*TMEM135*	9	11	1	Low expressed	Well expressed	Not expressed
ENSG00000082458	*DLG3*	10	3	1	Low expressed	Low expressed	Not expressed
ENSG00000169057	*MECP2*	10	12	11	Well expressed	Well expressed	Well expressed
ENSG00000046651	*OFD1*	10	14	6	Low expressed	Well expressed	Low expressed
ENSG00000148459	*PDSS1*	9	9	4	Low expressed	Low expressed	Low expressed
ENSG00000174227	*PIGG*	10	19	5	Low expressed	Well expressed	Low expressed
ENSG00000170370	*EMX2*	10	16	0	Well expressed	Well expressed	Not expressed
ENSG00000106692	*FKTN*	9	22	1	Low expressed	Well expressed	Not expressed
ENSG00000143473	*KCNH1*	9	0	0	Low expressed	Not expressed	Not expressed
ENSG00000135899	*SP110*	9	13	41	Low expressed	Well expressed	Well expressed
ENSG00000181090	*EHMT1*	10	8	3	Well expressed	Low expressed	Low expressed
ENSG00000125863	*MKKS*	10	13	3	Well expressed	Well expressed	Low expressed
ENSG00000115947	*ORC4*	10	8	3	Low expressed	Low expressed	Low expressed
ENSG00000055609	*KMT2C*	9	11	4	Low expressed	Well expressed	Low expressed
ENSG00000171735	*CAMTA1*	10	6	1	Low expressed	Low expressed	Low expressed
ENSG00000188419	*CHM*	10	12	1	Low expressed	Well expressed	Not expressed
ENSG00000011198	*ABHD5*	10	21	58	Low expressed	Well expressed	Well expressed
ENSG00000086848	*ALG9*	10	11	1	Well expressed	Well expressed	Low expressed
ENSG00000154124	*OTULIN*	10	10	4	Low expressed	Well expressed	Low expressed
ENSG00000162688	*AGL*	9	9	1	Low expressed	Low expressed	Low expressed
ENSG00000068724	*TTC7A*	10	32	13	Low expressed	Well expressed	Well expressed
ENSG00000144741	*SLC25A26*	10	8	2	Low expressed	Low expressed	Low expressed
ENSG00000184677	*ZBTB40*	9	14	4	Low expressed	Well expressed	Low expressed
ENSG00000038295	*TLL1*	7	1	0	Low expressed	Not expressed	Not expressed
ENSG00000130921	*C12orf65*	10	9	1	Low expressed	Low expressed	Low expressed
ENSG00000104450	*SPAG1*	10	2	1	Low expressed	Low expressed	Low expressed
ENSG00000005302	*MSL3*	10	16	43	Low expressed	Well expressed	Well expressed
ENSG00000111424	*VDR*	10	21	7	Low expressed	Well expressed	Low expressed
ENSG00000152219	*ARL14EP*	9	13	1	Low expressed	Well expressed	Low expressed
ENSG00000130347	*RTN4IP1*	9	9	1	Low expressed	Low expressed	Low expressed
ENSG00000110066	*KMT5B*	9	12	6	Low expressed	Well expressed	Low expressed
ENSG00000107104	*KANK1*	10	12	0	Low expressed	Well expressed	Not expressed
ENSG00000102125	*TAZ*	9	24	48	Low expressed	Well expressed	Well expressed
ENSG00000169184	*MN1*	8	3	0	Low expressed	Low expressed	Not expressed
ENSG00000126778	*SIX1*	5	15	0	Low expressed	Well expressed	Not expressed
ENSG00000088367	*EPB41L1*	9	10	0	Low expressed	Low expressed	Not expressed
ENSG00000126970	*ZC4H2*	9	5	1	Low expressed	Low expressed	Not expressed
ENSG00000152620	*NADK2*	8	18	1	Low expressed	Well expressed	Low expressed
ENSG00000188786	*MTF1*	10	11	11	Low expressed	Well expressed	Well expressed
ENSG00000138688	*KIAA1109*	8	18	3	Low expressed	Well expressed	Low expressed
ENSG00000012174	*MBTPS2*	9	17	0	Low expressed	Well expressed	Not expressed
ENSG00000163877	*SNIP1*	9	13	4	Low expressed	Well expressed	Low expressed
ENSG00000128039	*SRD5A3*	9	14	2	Low expressed	Well expressed	Low expressed
ENSG00000187535	*IFT140*	9	9	1	Low expressed	Low expressed	Not expressed
ENSG00000006530	*AGK*	9	17	3	Low expressed	Well expressed	Low expressed
ENSG00000121879	*PIK3CA*	8	20	4	Low expressed	Well expressed	Low expressed
ENSG00000162129	*CLPB*	9	13	4	Low expressed	Well expressed	Low expressed
ENSG00000119772	*DNMT3A*	9	4	4	Low expressed	Low expressed	Low expressed
ENSG00000147133	*TAF1*	9	22	3	Low expressed	Well expressed	Low expressed
ENSG00000173599	*PC*	9	18	1	Low expressed	Well expressed	Low expressed
ENSG00000112357	*PEX7*	9	10	1	Low expressed	Low expressed	Low expressed
ENSG00000177082	*WDR73*	9	13	2	Low expressed	Well expressed	Low expressed
ENSG00000011143	*MKS1*	9	12	1	Low expressed	Well expressed	Low expressed
ENSG00000088451	*TGDS*	9	17	2	Low expressed	Well expressed	Low expressed
ENSG00000060718	*COL11A1*	5	20	0	Low expressed	Well expressed	Not expressed
ENSG00000077942	*FBLN1*	7	662	0	Low expressed	Well expressed	Not expressed
ENSG00000166801	*FAM111A*	7	29	8	Low expressed	Well expressed	Low expressed
ENSG00000148516	*ZEB1*	7	79	2	Low expressed	Well expressed	Low expressed
ENSG00000163017	*ACTG2*	2	1	1	Low expressed	Low expressed	Not expressed
ENSG00000081014	*AP4E1*	8	14	1	Low expressed	Well expressed	Low expressed
ENSG00000072121	*ZFYVE26*	9	9	3	Low expressed	Low expressed	Low expressed

ENSG00000138759	*FRAS1*	9	11	0	Low expressed	Well expressed	Not expressed
ENSG00000149483	*TMEM138*	8	23	6	Low expressed	Well expressed	Low expressed
ENSG00000163961	*RNF168*	8	13	4	Low expressed	Well expressed	Low expressed
ENSG00000130363	*RSPH3*	8	7	1	Low expressed	Low expressed	Low expressed
ENSG00000168216	*LMBRD1*	8	37	28	Low expressed	Well expressed	Well expressed
ENSG00000197496	*SLC2A10*	8	46	0	Low expressed	Well expressed	Not expressed
ENSG00000168014	*C2CD3*	8	8	3	Low expressed	Low expressed	Low expressed
ENSG00000175745	*NR2F1*	5	28	0	Low expressed	Well expressed	Not expressed
ENSG00000151092	*NGLY1*	8	22	16	Low expressed	Well expressed	Well expressed
ENSG00000178573	*MAF*	5	0	3	Low expressed	Not expressed	Low expressed
ENSG00000141499	*WRAP53*	7	10	3	Low expressed	Low expressed	Low expressed
ENSG00000060642	*PIGV*	8	16	5	Low expressed	Well expressed	Low expressed
ENSG00000008853	*RHOBTB2*	8	16	2	Low expressed	Well expressed	Low expressed
ENSG00000162885	*B3GALNT2*	8	8	0	Low expressed	Low expressed	Not expressed
ENSG00000068885	*IFT80*	7	34	1	Low expressed	Well expressed	Not expressed
ENSG00000087494	*PTHLH*	6	0	0	Low expressed	Not expressed	Not expressed
ENSG00000144320	*LNPK*	7	19	3	Low expressed	Well expressed	Low expressed
ENSG00000140326	*CDAN1*	8	11	3	Low expressed	Well expressed	Low expressed
ENSG00000083093	*PALB2*	8	10	1	Low expressed	Well expressed	Not expressed
ENSG00000135365	*PHF21A*	8	22	56	Low expressed	Well expressed	Well expressed
ENSG00000178971	*CTC1*	9	5	14	Low expressed	Low expressed	Well expressed
ENSG00000172943	*PHF8*	9	10	8	Low expressed	Low expressed	Low expressed
ENSG00000125124	*BBS2*	8	35	4	Low expressed	Well expressed	Low expressed
ENSG00000198331	*HYLS1*	7	9	1	Low expressed	Low expressed	Low expressed
ENSG00000072210	*ALDH3A2*	7	21	4	Low expressed	Well expressed	Low expressed
ENSG00000116704	*SLC35D1*	8	24	2	Low expressed	Well expressed	Low expressed
ENSG00000182372	*CLN8*	8	6	3	Low expressed	Low expressed	Low expressed
ENSG00000110400	*NECTIN1*	8	5	2	Low expressed	Low expressed	Low expressed
ENSG00000136936	*XPA*	8	15	3	Low expressed	Well expressed	Low expressed
ENSG00000188603	*CLN3*	7	11	19	Low expressed	Well expressed	Well expressed
ENSG00000132740	*IGHMBP2*	8	10	8	Low expressed	Low expressed	Low expressed
ENSG00000188910	*GJB3*	6	0	0	Low expressed	Not expressed	Not expressed
ENSG00000140718	*FTO*	8	9	1	Low expressed	Low expressed	Not expressed
ENSG00000139428	*MMAB*	8	8	1	Low expressed	Low expressed	Low expressed
ENSG00000148384	*INPP5E*	8	11	2	Low expressed	Well expressed	Low expressed
ENSG00000085276	*MECOM*	7	1	0	Low expressed	Not expressed	Not expressed
ENSG00000114861	*FOXP1*	8	6	6	Low expressed	Low expressed	Low expressed
ENSG00000130023	*ERMARD*	8	25	4	Low expressed	Well expressed	Low expressed
ENSG00000168389	*MFSD2A*	6	6	1	Low expressed	Low expressed	Low expressed
ENSG00000141013	*GAS8*	8	7	1	Low expressed	Low expressed	Not expressed
ENSG00000144535	*DIS3L2*	8	7	2	Low expressed	Low expressed	Low expressed
ENSG00000148090	*AUH*	7	12	3	Low expressed	Well expressed	Low expressed
ENSG00000134569	*LRP4*	7	6	0	Low expressed	Low expressed	Not expressed
ENSG00000272047	*GTF2H5*	7	17	1	Low expressed	Well expressed	Not expressed
ENSG00000001631	*KRIT1*	7	26	2	Low expressed	Well expressed	Low expressed
ENSG00000118965	*WDR35*	8	11	1	Low expressed	Well expressed	Not expressed
ENSG00000181481	*RNF135*	8	15	17	Low expressed	Well expressed	Well expressed
ENSG00000138796	*HADH*	8	20	2	Low expressed	Well expressed	Low expressed
ENSG00000132549	*VPS13B*	7	11	3	Low expressed	Well expressed	Low expressed
ENSG00000074181	*NOTCH3*	6	29	0	Low expressed	Well expressed	Not expressed
ENSG00000175054	*ATR*	7	14	1	Low expressed	Well expressed	Low expressed
ENSG00000169814	*BTD*	7	10	3	Low expressed	Low expressed	Low expressed
ENSG00000074047	*GLI2*	7	8	0	Low expressed	Low expressed	Not expressed
ENSG00000187049	*TMEM216*	8	16	8	Low expressed	Well expressed	Low expressed
ENSG00000121957	*GPSM2*	6	13	1	Low expressed	Well expressed	Low expressed
ENSG00000174842	*GLMN*	7	14	1	Low expressed	Well expressed	Not expressed
ENSG00000051180	*RAD51*	4	7	1	Low expressed	Low expressed	Not expressed
ENSG00000079482	*OPHN1*	7	4	0	Low expressed	Low expressed	Not expressed
ENSG00000175387	*SMAD2*	7	6	2	Low expressed	Low expressed	Low expressed
ENSG00000115425	*PECR*	7	7	7	Low expressed	Low expressed	Low expressed
ENSG00000101901	*ALG13*	7	12	2	Low expressed	Well expressed	Low expressed
ENSG00000130299	*GTPBP3*	7	13	2	Low expressed	Well expressed	Low expressed
ENSG00000112234	*FBXL4*	8	11	2	Low expressed	Well expressed	Low expressed
ENSG00000105991	*HOXA1*	8	3	0	Low expressed	Low expressed	Not expressed
ENSG00000125351	*UPF3B*	7	18	3	Low expressed	Well expressed	Low expressed
ENSG00000149311	*ATM*	6	16	4	Low expressed	Well expressed	Low expressed
ENSG00000008196	*TFAP2B*	7	0	0	Low expressed	Not expressed	Not expressed
ENSG00000091651	*ORC6*	4	12	0	Low expressed	Well expressed	Not expressed
ENSG00000118197	*DDX59*	8	8	5	Low expressed	Low expressed	Low expressed
ENSG00000119509	*INVS*	7	10	2	Low expressed	Well expressed	Low expressed
ENSG00000126351	*THRA*	7	25	5	Low expressed	Well expressed	Low expressed
ENSG00000147364	*FBXO25*	7	6	1	Low expressed	Low expressed	Low expressed
ENSG00000197283	*SYNGAP1*	7	14	3	Low expressed	Well expressed	Low expressed
ENSG00000133424	*LARGE1*	7	4	0	Low expressed	Low expressed	Not expressed
ENSG00000166813	*KIF7*	7	19	0	Low expressed	Well expressed	Not expressed
ENSG00000134262	*AP4B1*	7	11	7	Low expressed	Well expressed	Low expressed
ENSG00000145982	*FARS2*	7	14	3	Low expressed	Well expressed	Low expressed
ENSG00000160299	*PCNT*	6	15	3	Low expressed	Well expressed	Low expressed
ENSG00000165699	*TSC1*	7	23	4	Low expressed	Well expressed	Low expressed
ENSG00000141012	*GALNS*	7	18	13	Low expressed	Well expressed	Well expressed
ENSG00000157353	*FCSK*	7	6	4	Low expressed	Low expressed	Low expressed
ENSG00000010072	*SPRTN*	7	6	1	Low expressed	Low expressed	Low expressed
ENSG00000080503	*SMARCA2*	7	17	6	Low expressed	Well expressed	Low expressed
ENSG00000143387	*CTSK*	2	347	6	Low expressed	Well expressed	Low expressed
ENSG00000066279	*ASPM*	2	11	0	Low expressed	Well expressed	Not expressed
ENSG00000137203	*TFAP2A*	5	0	0	Low expressed	Not expressed	Not expressed
ENSG00000138821	*SLC39A8*	7	14	3	Low expressed	Well expressed	Low expressed
ENSG00000165533	*TTC8*	7	12	0	Low expressed	Well expressed	Not expressed
ENSG00000184979	*USP18*	6	4	0	Low expressed	Low expressed	Not expressed
ENSG00000137601	*NEK1*	6	12	0	Low expressed	Well expressed	Not expressed
ENSG00000126953	*TIMM8A*	7	11	1	Low expressed	Well expressed	Not expressed
ENSG00000187676	*B3GLCT*	7	11	1	Low expressed	Well expressed	Not expressed
ENSG00000157399	*ARSL*	6	1	0	Low expressed	Not expressed	Not expressed

ENSG00000213689	*TREX1*	7	21	18	Low expressed	Well expressed	Well expressed
ENSG00000042429	*MED17*	7	11	3	Low expressed	Well expressed	Low expressed
ENSG00000110274	*CEP164*	7	17	4	Low expressed	Well expressed	Low expressed
ENSG00000171100	*MTM1*	7	7	3	Low expressed	Low expressed	Low expressed
ENSG00000189079	*ARID2*	6	8	3	Low expressed	Low expressed	Low expressed
ENSG00000140199	*SLC12A6*	7	9	28	Low expressed	Low expressed	Well expressed
ENSG00000126870	*DYNC2I1*	6	17	1	Low expressed	Well expressed	Low expressed
ENSG00000185527	*PDE6G*	5	0	3	Low expressed	Not expressed	Low expressed
ENSG00000069943	*PIGB*	7	14	6	Low expressed	Well expressed	Low expressed
ENSG00000147316	*MCPH1*	7	6	1	Low expressed	Low expressed	Low expressed
ENSG00000138002	*IFT172*	7	13	1	Low expressed	Well expressed	Not expressed
ENSG00000132535	*DLG4*	6	19	5	Low expressed	Well expressed	Low expressed
ENSG00000143315	*PIGM*	6	11	1	Low expressed	Well expressed	Low expressed
ENSG00000184937	*WT1*	4	0	0	Low expressed	Not expressed	Not expressed
ENSG00000169607	*CKAP2L*	2	9	0	Low expressed	Low expressed	Not expressed
ENSG00000101868	*POLA1*	6	8	1	Low expressed	Low expressed	Not expressed
ENSG00000112039	*FANCE*	6	14	3	Low expressed	Well expressed	Low expressed
ENSG00000048342	*CC2D2A*	6	11	0	Low expressed	Well expressed	Not expressed
ENSG00000167244	*IGF2*	2	21	0	Low expressed	Well expressed	Not expressed
ENSG00000033030	*ZCCHC8*	6	11	3	Low expressed	Well expressed	Low expressed
ENSG00000113971	*NPHP3*	6	26	2	Low expressed	Well expressed	Low expressed
ENSG00000043514	*TRIT1*	6	23	3	Low expressed	Well expressed	Low expressed
ENSG00000164736	*SOX17*	4	0	0	Low expressed	Not expressed	Not expressed
ENSG00000008311	*AASS*	6	23	0	Low expressed	Well expressed	Not expressed
ENSG00000076351	*SLC46A1*	6	3	0	Low expressed	Low expressed	Not expressed
ENSG00000168303	*MPLKIP*	6	12	2	Low expressed	Well expressed	Low expressed
ENSG00000145675	*PIK3R1*	5	36	9	Low expressed	Well expressed	Low expressed
ENSG00000128513	*POT1*	7	13	2	Low expressed	Well expressed	Low expressed
ENSG00000198689	*SLC9A6*	6	12	3	Low expressed	Well expressed	Low expressed
ENSG00000132600	*PRMT7*	6	13	3	Low expressed	Well expressed	Low expressed
ENSG00000137992	*DBT*	6	8	1	Low expressed	Low expressed	Not expressed
ENSG00000188158	*NHS*	6	2	0	Low expressed	Low expressed	Not expressed
ENSG00000142330	*CAPN10*	6	10	8	Low expressed	Low expressed	Low expressed
ENSG00000182173	*TSEN54*	6	12	12	Low expressed	Well expressed	Well expressed
ENSG00000095002	*MSH2*	6	20	1	Low expressed	Well expressed	Low expressed
ENSG00000205084	*TMEM231*	7	7	0	Low expressed	Low expressed	Not expressed
ENSG00000184304	*PRKD1*	6	46	0	Low expressed	Well expressed	Not expressed
ENSG00000013573	*DDX11*	5	9	3	Low expressed	Low expressed	Low expressed
ENSG00000020922	*MRE11*	6	12	3	Low expressed	Well expressed	Low expressed
ENSG00000085840	*ORC1*	3	5	1	Low expressed	Low expressed	Low expressed
ENSG00000069966	*GNB5*	6	8	1	Low expressed	Low expressed	Low expressed
ENSG00000115267	*IFIH1*	6	4	3	Low expressed	Low expressed	Low expressed
ENSG00000198707	*CEP290*	5	12	0	Low expressed	Well expressed	Not expressed
ENSG00000196236	*XPNPEP3*	6	9	1	Low expressed	Low expressed	Low expressed
ENSG00000124370	*MCEE*	6	16	2	Low expressed	Well expressed	Low expressed
ENSG00000157184	*CPT2*	6	12	3	Low expressed	Well expressed	Low expressed
ENSG00000110195	*FOLR1*	4	0	0	Low expressed	Not expressed	Not expressed
ENSG00000164169	*PRMT9*	6	11	2	Low expressed	Well expressed	Low expressed
ENSG00000243335	*KCTD7*	6	9	3	Low expressed	Low expressed	Low expressed
ENSG00000083168	*KAT6A*	6	17	17	Low expressed	Well expressed	Well expressed
ENSG00000049167	*ERCC8*	6	10	1	Low expressed	Well expressed	Not expressed
ENSG00000127980	*PEX1*	6	12	2	Low expressed	Well expressed	Low expressed
ENSG00000162733	*DDR2*	2	110	0	Low expressed	Well expressed	Not expressed
ENSG00000019186	*CYP24A1*	1	0	0	Not expressed	Not expressed	Not expressed
ENSG00000163818	*LZTFL1*	6	11	1	Low expressed	Well expressed	Not expressed
ENSG00000177000	*MTHFR*	6	8	5	Low expressed	Low expressed	Low expressed
ENSG00000108384	*RAD51C*	5	6	1	Low expressed	Low expressed	Not expressed
ENSG00000106344	*RBM28*	6	9	1	Low expressed	Low expressed	Low expressed
ENSG00000157796	*WDR19*	6	18	1	Low expressed	Well expressed	Not expressed
ENSG00000144554	*FANCD2*	3	4	2	Low expressed	Low expressed	Low expressed
ENSG00000178966	*RMI1*	5	11	2	Low expressed	Well expressed	Low expressed
ENSG00000137868	*STRA6*	4	0	0	Low expressed	Not expressed	Not expressed
ENSG00000160226	*CFAP410*	6	11	7	Low expressed	Well expressed	Low expressed
ENSG00000013619	*MAMLD1*	6	13	0	Low expressed	Well expressed	Not expressed
ENSG00000197822	*OCLN*	5	0	0	Low expressed	Not expressed	Not expressed
ENSG00000088836	*SLC4A11*	5	3	0	Low expressed	Low expressed	Not expressed
ENSG00000180902	*D2HGDH*	5	21	9	Low expressed	Well expressed	Low expressed
ENSG00000074266	*EED*	5	15	3	Low expressed	Well expressed	Low expressed
ENSG00000196459	*TRAPPC2*	5	9	6	Low expressed	Low expressed	Low expressed
ENSG00000175198	*PCCA*	5	7	1	Low expressed	Low expressed	Not expressed
ENSG00000123473	*STIL*	4	6	0	Low expressed	Low expressed	Not expressed
ENSG00000185129	*PURA*	5	11	1	Low expressed	Well expressed	Not expressed
ENSG00000160957	*RECQL4*	4	11	2	Low expressed	Well expressed	Low expressed
ENSG00000067715	*SYT1*	5	0	0	Low expressed	Not expressed	Not expressed
ENSG00000123607	*TTC21B*	6	12	1	Low expressed	Well expressed	Low expressed
ENSG00000072195	*SPEG*	5	5	0	Low expressed	Low expressed	Not expressed
ENSG00000070269	*TMEM260*	5	10	6	Low expressed	Well expressed	Low expressed
ENSG00000113163	*CERT1*	5	19	12	Low expressed	Well expressed	Well expressed
ENSG00000145348	*TBCK*	6	11	1	Low expressed	Well expressed	Not expressed
ENSG00000155016	*CYP2U1*	5	11	0	Low expressed	Well expressed	Not expressed
ENSG00000115827	*DCAF17*	5	11	1	Low expressed	Well expressed	Low expressed
ENSG00000123066	*MED13L*	5	29	6	Low expressed	Well expressed	Low expressed
ENSG00000169379	*ARL13B*	5	13	0	Low expressed	Well expressed	Not expressed
ENSG00000163867	*ZMYM6*	5	8	2	Low expressed	Low expressed	Low expressed
ENSG00000108733	*PEX12*	5	11	1	Low expressed	Well expressed	Not expressed
ENSG00000120370	*GORAB*	5	15	1	Low expressed	Well expressed	Not expressed
ENSG00000120149	*MSX2*	5	6	0	Low expressed	Low expressed	Not expressed
ENSG00000175595	*ERCC4*	5	6	0	Low expressed	Low expressed	Not expressed
ENSG00000163251	*FZD5*	5	1	0	Low expressed	Not expressed	Not expressed
ENSG00000171316	*CHD7*	5	1	3	Low expressed	Low expressed	Low expressed
ENSG00000198130	*HIBCH*	5	11	2	Low expressed	Well expressed	Low expressed
ENSG00000122507	*BBS9*	5	9	1	Low expressed	Low expressed	Low expressed
ENSG00000115657	*ABCB6*	5	26	8	Low expressed	Well expressed	Low expressed

ENSG00000065883	*CDK13*	5	22	6	Low expressed	Well expressed	Low expressed
ENSG00000173068	*BNC2*	5	12	0	Low expressed	Well expressed	Not expressed
ENSG00000121361	*KCNJ8*	2	21	0	Low expressed	Well expressed	Not expressed
ENSG00000187741	*FANCA*	4	7	2	Low expressed	Low expressed	Low expressed
ENSG00000118600	*RXYLT1*	5	8	1	Low expressed	Low expressed	Not expressed
ENSG00000188827	*SLX4*	5	6	3	Low expressed	Low expressed	Low expressed
ENSG00000181541	*MAB21L2*	2	0	0	Low expressed	Not expressed	Not expressed
ENSG00000168778	*TCTN2*	5	13	0	Low expressed	Well expressed	Not expressed
ENSG00000142731	*PLK4*	2	7	0	Low expressed	Low expressed	Not expressed
ENSG00000151413	*NUBPL*	5	10	0	Low expressed	Low expressed	Not expressed
ENSG00000102359	*SRPX2*	3	35	0	Low expressed	Well expressed	Not expressed
ENSG00000103489	*XYLT1*	1	29	2	Low expressed	Well expressed	Low expressed
ENSG00000181722	*ZBTB20*	4	0	0	Low expressed	Not expressed	Not expressed
ENSG00000120071	*KANSL1*	5	11	9	Low expressed	Well expressed	Low expressed
ENSG00000142675	*CNKSR1*	4	0	0	Low expressed	Not expressed	Not expressed
ENSG00000139323	*POC1B*	5	9	8	Low expressed	Low expressed	Low expressed
ENSG00000102575	*ACP5*	4	1	13	Low expressed	Low expressed	Well expressed
ENSG00000197603	*CPLANE1*	4	9	0	Low expressed	Low expressed	Not expressed
ENSG00000163913	*IFT122*	5	18	3	Low expressed	Well expressed	Low expressed
ENSG00000165288	*BRWD3*	4	6	4	Low expressed	Low expressed	Low expressed
ENSG00000127415	*IDUA*	5	12	5	Low expressed	Well expressed	Low expressed
ENSG00000109323	*MANBA*	4	26	19	Low expressed	Well expressed	Well expressed
ENSG00000015133	*CCDC88C*	5	0	11	Low expressed	Not expressed	Well expressed
ENSG00000241635	*UGT1A1*	2	0	0	Low expressed	Not expressed	Not expressed
ENSG00000164099	*PRSS12*	3	36	0	Low expressed	Well expressed	Not expressed
ENSG00000169762	*TAPT1*	4	7	4	Low expressed	Low expressed	Low expressed
ENSG00000049618	*ARID1B*	5	8	2	Low expressed	Low expressed	Low expressed
ENSG00000040933	*INPP4A*	5	8	10	Low expressed	Low expressed	Low expressed
ENSG00000104218	*CSPP1*	4	7	1	Low expressed	Low expressed	Not expressed
ENSG00000185344	*ATP6V0A2*	4	6	2	Low expressed	Low expressed	Low expressed
ENSG00000154122	*ANKH*	4	15	5	Low expressed	Well expressed	Low expressed
ENSG00000165240	*ATP7A*	4	6	2	Low expressed	Low expressed	Low expressed
ENSG00000172915	*NBEA*	4	1	0	Low expressed	Low expressed	Not expressed
ENSG00000106477	*CEP41*	5	5	0	Low expressed	Low expressed	Not expressed
ENSG00000125454	*SLC25A19*	4	6	4	Low expressed	Low expressed	Low expressed
ENSG00000136492	*BRIP1*	3	4	0	Low expressed	Low expressed	Not expressed
ENSG00000182150	*ERCC6L2*	4	6	1	Low expressed	Low expressed	Not expressed
ENSG00000156466	*GDF6*	1	3	0	Low expressed	Low expressed	Not expressed
ENSG00000204852	*TCTN1*	4	19	1	Low expressed	Well expressed	Not expressed
ENSG00000080493	*SLC4A4*	3	1	0	Low expressed	Low expressed	Not expressed
ENSG00000182923	*CEP63*	4	13	8	Low expressed	Well expressed	Low expressed
ENSG00000100578	*KIAA0586*	4	6	1	Low expressed	Low expressed	Low expressed
ENSG00000135297	*MTO1*	4	6	2	Low expressed	Low expressed	Low expressed
ENSG00000088035	*ALG6*	4	7	3	Low expressed	Low expressed	Low expressed
ENSG00000122966	*CIT*	2	11	0	Low expressed	Well expressed	Not expressed
ENSG00000101846	*STS*	4	19	2	Low expressed	Well expressed	Low expressed
ENSG00000107890	*ANKRD26*	4	7	0	Low expressed	Low expressed	Not expressed
ENSG00000073464	*CLCN4*	4	1	0	Low expressed	Low expressed	Not expressed
ENSG00000081052	*COL4A4*	4	7	0	Low expressed	Low expressed	Not expressed
ENSG00000104447	*TRPS1*	4	7	2	Low expressed	Low expressed	Low expressed
ENSG00000251322	*SHANK3*	4	3	0	Low expressed	Low expressed	Not expressed
ENSG00000183287	*CCBE1*	3	121	0	Low expressed	Well expressed	Not expressed
ENSG00000197594	*ENPP1*	3	6	0	Low expressed	Low expressed	Not expressed
ENSG00000273820	*USP27X*	4	5	0	Low expressed	Low expressed	Not expressed
ENSG00000115355	*CCDC88A*	3	17	2	Low expressed	Well expressed	Low expressed
ENSG00000101680	*LAMA1*	3	17	0	Low expressed	Well expressed	Not expressed
ENSG00000113594	*LIFR*	4	7	0	Low expressed	Low expressed	Not expressed
ENSG00000147862	*NFIB*	3	5	0	Low expressed	Low expressed	Not expressed
ENSG00000116127	*ALMS1*	4	5	0	Low expressed	Low expressed	Not expressed
ENSG00000112294	*ALDH5A1*	4	1	2	Low expressed	Not expressed	Low expressed
ENSG00000064309	*CDON*	4	2	0	Low expressed	Low expressed	Not expressed
ENSG00000161653	*NAGS*	4	18	1	Low expressed	Well expressed	Not expressed
ENSG00000074621	*SLC24A1*	4	9	0	Low expressed	Low expressed	Not expressed
ENSG00000111341	*MGP*	1	17	3	Not expressed	Well expressed	Low expressed
ENSG00000165195	*PIGA*	4	6	2	Low expressed	Low expressed	Low expressed
ENSG00000157540	*DYRK1A*	4	15	7	Low expressed	Well expressed	Low expressed
ENSG00000197375	*SLC22A5*	4	4	1	Low expressed	Low expressed	Low expressed
ENSG00000102805	*CLN5*	4	3	1	Low expressed	Low expressed	Not expressed
ENSG00000154743	*TSEN2*	4	10	1	Low expressed	Low expressed	Not expressed
ENSG00000181004	*BBS12*	4	7	0	Low expressed	Low expressed	Not expressed
ENSG00000011201	*ANOS1*	3	0	0	Low expressed	Not expressed	Not expressed
ENSG00000149571	*KIRREL3*	4	1	0	Low expressed	Not expressed	Not expressed
ENSG00000169126	*ARMC4*	3	0	0	Low expressed	Not expressed	Not expressed
ENSG00000118193	*KIF14*	2	6	0	Low expressed	Low expressed	Not expressed
ENSG00000104267	*CA2*	3	0	14	Low expressed	Not expressed	Well expressed
ENSG00000186153	*WWOX*	4	2	1	Low expressed	Low expressed	Not expressed
ENSG00000163093	*BBS5*	4	12	0	Low expressed	Well expressed	Not expressed
ENSG00000138346	*DNA2*	2	3	1	Low expressed	Low expressed	Not expressed
ENSG00000135541	*AHI1*	3	9	0	Low expressed	Low expressed	Not expressed
ENSG00000112541	*PDE10A*	3	0	0	Low expressed	Not expressed	Not expressed
ENSG00000198799	*LRIG2*	4	6	1	Low expressed	Low expressed	Low expressed
ENSG00000113448	*PDE4D*	4	9	3	Low expressed	Low expressed	Low expressed
ENSG00000148204	*CRB2*	1	0	0	Low expressed	Not expressed	Not expressed
ENSG00000169372	*CRADD*	3	7	2	Low expressed	Low expressed	Low expressed
ENSG00000197563	*PIGN*	4	7	1	Low expressed	Low expressed	Not expressed
ENSG00000233608	*TWIST2*	1	259	0	Low expressed	Well expressed	Not expressed
ENSG00000163050	*COQ8A*	4	6	7	Low expressed	Low expressed	Low expressed
ENSG00000077092	*RARB*	2	2	0	Low expressed	Low expressed	Not expressed
ENSG00000164690	*SHH*	3	0	0	Low expressed	Not expressed	Not expressed
ENSG00000116132	*PRRX1*	0	96	0	Not expressed	Well expressed	Not expressed
ENSG00000174010	*KLHL15*	3	7	1	Low expressed	Low expressed	Low expressed
ENSG00000165474	*GJB2*	3	0	0	Low expressed	Not expressed	Not expressed
ENSG00000012048	*BRCA1*	2	7	1	Low expressed	Low expressed	Not expressed

ENSG00000145375	*SPATA5*	3	3	0	Low expressed	Low expressed	Not expressed
ENSG00000144218	*AFF3*	3	0	1	Low expressed	Not expressed	Not expressed
ENSG00000008086	*CDKL5*	3	2	1	Low expressed	Low expressed	Not expressed
ENSG00000131018	*SYNE1*	3	6	4	Low expressed	Low expressed	Low expressed
ENSG00000151849	*CENPJ*	2	7	1	Low expressed	Low expressed	Not expressed
ENSG00000151611	*MMAA*	3	4	0	Low expressed	Low expressed	Not expressed
ENSG00000102967	*DHODH*	3	5	1	Low expressed	Low expressed	Not expressed
ENSG00000196628	*TCF4*	3	12	0	Low expressed	Well expressed	Not expressed
ENSG00000173040	*EVC2*	4	10	0	Low expressed	Low expressed	Not expressed
ENSG00000187240	*DYNC2H1*	3	14	0	Low expressed	Well expressed	Not expressed
ENSG00000106571	*GLI3*	3	15	0	Low expressed	Well expressed	Not expressed
ENSG00000137070	*IL11RA*	3	24	7	Low expressed	Well expressed	Low expressed
ENSG00000197121	*PGAP1*	3	4	0	Low expressed	Low expressed	Not expressed
ENSG00000183763	*TRAIP*	2	5	1	Low expressed	Low expressed	Not expressed
ENSG00000163945	*UVSSA*	3	7	4	Low expressed	Low expressed	Low expressed
ENSG00000100523	*DDHD1*	3	8	1	Low expressed	Low expressed	Low expressed
ENSG00000077264	*PAK3*	3	0	0	Low expressed	Not expressed	Not expressed
ENSG00000256061	*DNAAF4*	3	2	0	Low expressed	Low expressed	Not expressed
ENSG00000169554	*ZEB2*	3	14	10	Low expressed	Well expressed	Low expressed
ENSG00000164073	*MFSD8*	3	9	2	Low expressed	Low expressed	Low expressed
ENSG00000143669	*LYST*	3	7	12	Low expressed	Low expressed	Well expressed
ENSG00000006016	*CRLF1*	3	22	0	Low expressed	Well expressed	Not expressed
ENSG00000184675	*AMER1*	3	3	0	Low expressed	Low expressed	Not expressed
ENSG00000108474	*PIGL*	3	6	1	Low expressed	Low expressed	Low expressed
ENSG00000087299	*L2HGDH*	3	3	0	Low expressed	Low expressed	Not expressed
ENSG00000188517	*COL25A1*	2	0	0	Low expressed	Not expressed	Not expressed
ENSG00000103494	*RPGRIP1L*	3	7	0	Low expressed	Low expressed	Not expressed
ENSG00000144182	*LIPT1*	3	5	1	Low expressed	Low expressed	Low expressed
ENSG00000162769	*FLVCR1*	2	5	1	Low expressed	Low expressed	Not expressed
ENSG00000147180	*ZNF711*	3	1	0	Low expressed	Low expressed	Not expressed
ENSG00000157764	*BRAF*	3	11	6	Low expressed	Well expressed	Low expressed
ENSG00000085382	*HACE1*	3	10	1	Low expressed	Well expressed	Not expressed
ENSG00000118402	*ELOVL4*	3	9	0	Low expressed	Low expressed	Not expressed
ENSG00000160602	*NEK8*	3	4	2	Low expressed	Low expressed	Low expressed
ENSG00000174799	*CEP135*	2	8	1	Low expressed	Low expressed	Low expressed
ENSG00000130600	*H19*	2	1	1	Low expressed	Not expressed	Not expressed
ENSG00000127955	*GNAI1*	3	31	0	Low expressed	Well expressed	Not expressed
ENSG00000225830	*ERCC6*	2	8	1	Low expressed	Low expressed	Not expressed
ENSG00000197299	*BLM*	2	2	1	Low expressed	Low expressed	Low expressed
ENSG00000141052	*MYOCD*	2	0	0	Low expressed	Not expressed	Not expressed
ENSG00000162065	*TBC1D24*	3	6	1	Low expressed	Low expressed	Not expressed
ENSG00000121897	*LIAS*	3	3	1	Low expressed	Low expressed	Not expressed
ENSG00000189056	*RELN*	2	0	0	Low expressed	Not expressed	Not expressed
ENSG00000132932	*ATP8A2*	2	0	0	Low expressed	Not expressed	Not expressed
ENSG00000160801	*PTH1R*	1	2	0	Not expressed	Low expressed	Not expressed
ENSG00000158169	*FANCC*	3	4	0	Low expressed	Low expressed	Not expressed
ENSG00000205413	*SAMD9*	2	12	5	Low expressed	Well expressed	Low expressed
ENSG00000068024	*HDAC4*	3	6	10	Low expressed	Low expressed	Well expressed
ENSG00000059377	*TBXAS1*	2	2	68	Low expressed	Low expressed	Well expressed
ENSG00000179981	*TSHZ1*	3	12	2	Low expressed	Well expressed	Low expressed
ENSG00000147257	*GPC3*	1	3	0	Low expressed	Low expressed	Not expressed
ENSG00000075702	*WDR62*	1	3	1	Low expressed	Low expressed	Not expressed
ENSG00000114739	*ACVR2B*	3	1	0	Low expressed	Low expressed	Not expressed
ENSG00000131089	*ARHGEF9*	3	4	1	Low expressed	Low expressed	Not expressed
ENSG00000179348	*GATA2*	1	6	1	Low expressed	Low expressed	Not expressed
ENSG00000126091	*ST3GAL3*	3	12	2	Low expressed	Well expressed	Low expressed
ENSG00000155974	*GRIP1*	2	0	0	Low expressed	Not expressed	Not expressed
ENSG00000137834	*SMAD6*	2	3	0	Low expressed	Low expressed	Not expressed
ENSG00000144452	*ABCA12*	2	0	0	Low expressed	Not expressed	Not expressed
ENSG00000198947	*DMD*	2	1	0	Low expressed	Low expressed	Not expressed
ENSG00000163132	*MSX1*	1	23	0	Not expressed	Well expressed	Not expressed
ENSG00000173588	*CEP83*	2	7	0	Low expressed	Low expressed	Not expressed
ENSG00000184381	*PLA2G6*	2	7	10	Low expressed	Low expressed	Low expressed
ENSG00000122691	*TWIST1*	1	128	0	Not expressed	Well expressed	Not expressed
ENSG00000110723	*EXPH5*	2	0	0	Low expressed	Not expressed	Not expressed
ENSG00000140092	*FBLN5*	2	292	1	Low expressed	Well expressed	Not expressed
ENSG00000197467	*COL13A1*	1	6	0	Low expressed	Low expressed	Not expressed
ENSG00000124313	*IQSEC2*	2	5	1	Low expressed	Low expressed	Not expressed
ENSG00000198814	*GK*	2	10	26	Low expressed	Well expressed	Well expressed
ENSG00000148826	*NKX6-2*	3	0	0	Low expressed	Not expressed	Not expressed
ENSG00000113966	*ARL6*	2	4	0	Low expressed	Low expressed	Not expressed
ENSG00000167131	*CCDC103*	2	0	0	Low expressed	Not expressed	Not expressed
ENSG00000162105	*SHANK2*	2	1	0	Low expressed	Not expressed	Not expressed
ENSG00000156172	*C8orf37*	2	2	0	Low expressed	Low expressed	Not expressed
ENSG00000109618	*SEPSECS*	2	7	1	Low expressed	Low expressed	Not expressed
ENSG00000162551	*ALPL*	1	4	598	Not expressed	Low expressed	Well expressed
ENSG00000100478	*AP4S1*	2	3	1	Low expressed	Low expressed	Not expressed
ENSG00000140471	*LINS1*	2	5	3	Low expressed	Low expressed	Low expressed
ENSG00000142303	*ADAMTS10*	2	4	3	Low expressed	Low expressed	Low expressed
ENSG00000131697	*NPHP4*	2	8	2	Low expressed	Low expressed	Low expressed
ENSG00000105639	*JAK3*	2	3	139	Low expressed	Low expressed	Well expressed
ENSG00000175536	*LIPT2*	2	2	1	Low expressed	Low expressed	Not expressed
ENSG00000196159	*FAT4*	2	21	0	Low expressed	Well expressed	Not expressed
ENSG00000143632	*ACTA1*	2	3	4	Low expressed	Low expressed	Low expressed
ENSG00000130294	*KIF1A*	1	0	0	Low expressed	Not expressed	Not expressed
ENSG00000115525	*ST3GAL5*	2	4	1	Low expressed	Low expressed	Low expressed
ENSG00000270141	*TERC*	2	0	0	Low expressed	Not expressed	Not expressed
ENSG00000103150	*MLYCD*	2	3	1	Low expressed	Low expressed	Not expressed
ENSG00000147099	*HDAC8*	2	8	1	Low expressed	Low expressed	Low expressed
ENSG00000156150	*ALX3*	2	0	0	Low expressed	Not expressed	Not expressed
ENSG00000176842	*IRX5*	2	7	0	Low expressed	Low expressed	Not expressed
ENSG00000164093	*PITX2*	2	4	0	Low expressed	Low expressed	Not expressed
ENSG00000112837	*TBX18*	2	27	0	Low expressed	Well expressed	Not expressed

ENSG00000138696	*BMPR1B*	2	1	0	Low expressed	Low expressed	Not expressed
ENSG00000112246	*SIM1*	1	6	0	Low expressed	Low expressed	Not expressed
ENSG00000204406	*MBD5*	2	3	1	Low expressed	Low expressed	Not expressed
ENSG00000120519	*SLC10A7*	2	4	1	Low expressed	Low expressed	Not expressed
ENSG00000158321	*AUTS2*	2	4	1	Low expressed	Low expressed	Not expressed
ENSG00000138193	*PLCE1*	2	3	0	Low expressed	Low expressed	Not expressed
ENSG00000183770	*FOXL2*	2	1	0	Low expressed	Not expressed	Not expressed
ENSG00000103995	*CEP152*	2	4	1	Low expressed	Low expressed	Not expressed
ENSG00000069011	*PITX1*	0	2	0	Not expressed	Low expressed	Not expressed
ENSG00000065361	*ERBB3*	2	0	0	Low expressed	Not expressed	Not expressed
ENSG00000143217	*NECTIN4*	2	0	0	Low expressed	Not expressed	Not expressed
ENSG00000103241	*FOXF1*	0	12	0	Not expressed	Well expressed	Not expressed
ENSG00000099365	*STX1B*	2	2	0	Low expressed	Low expressed	Not expressed
ENSG00000171320	*ESCO2*	1	3	0	Not expressed	Low expressed	Not expressed
ENSG00000140386	*SCAPER*	2	4	1	Low expressed	Low expressed	Low expressed
ENSG00000154133	*ROBO4*	2	4	0	Low expressed	Low expressed	Not expressed
ENSG00000119042	*SATB2*	2	8	0	Low expressed	Low expressed	Not expressed
ENSG00000164120	*HPGD*	1	0	5	Low expressed	Not expressed	Low expressed
ENSG00000156650	*KAT6B*	2	10	1	Low expressed	Low expressed	Not expressed
ENSG00000204644	*ZFP57*	1	0	0	Not expressed	Not expressed	Not expressed
ENSG00000107859	*PITX3*	2	0	0	Low expressed	Not expressed	Not expressed
ENSG00000090932	*DLL3*	2	1	0	Low expressed	Not expressed	Not expressed
ENSG00000171105	*INSR*	2	8	1	Low expressed	Low expressed	Low expressed
ENSG00000178665	*ZNF713*	2	1	0	Low expressed	Not expressed	Not expressed
ENSG00000164953	*TMEM67*	2	7	0	Low expressed	Low expressed	Not expressed
ENSG00000100150	*DEPDC5*	2	6	3	Low expressed	Low expressed	Low expressed
ENSG00000137691	*CFAP300*	2	6	0	Low expressed	Low expressed	Not expressed
ENSG00000049089	*COL9A2*	1	1	9	Low expressed	Not expressed	Low expressed
ENSG00000170264	*FAM161A*	1	4	0	Low expressed	Low expressed	Not expressed
ENSG00000108379	*WNT3*	2	1	0	Low expressed	Low expressed	Not expressed
ENSG00000164619	*BMPER*	1	19	0	Not expressed	Well expressed	Not expressed
ENSG00000119699	*TGFB3*	2	4	1	Low expressed	Low expressed	Not expressed
ENSG00000136944	*LMX1B*	1	0	0	Low expressed	Not expressed	Not expressed
ENSG00000169297	*NR0B1*	1	0	0	Low expressed	Not expressed	Not expressed
ENSG00000123700	*KCNJ2*	1	2	6	Not expressed	Low expressed	Low expressed
ENSG00000155816	*FMN2*	1	12	0	Not expressed	Well expressed	Not expressed
ENSG00000111199	*TRPV4*	1	3	0	Low expressed	Low expressed	Not expressed
ENSG00000185760	*KCNQ5*	1	0	0	Low expressed	Not expressed	Not expressed
ENSG00000176225	*RTTN*	1	7	0	Low expressed	Low expressed	Not expressed
ENSG00000130283	*GDF1*	1	No data	No data	Low expressed	No data	No data
ENSG00000162426	*SLC45A1*	2	5	0	Low expressed	Low expressed	Not expressed
ENSG00000174469	*CNTNAP2*	1	0	0	Low expressed	Low expressed	Not expressed
ENSG00000122877	*EGR2*	0	0	0	Not expressed	Not expressed	Not expressed
ENSG00000021645	*NRXN3*	1	0	0	Low expressed	Not expressed	Not expressed
ENSG00000153896	*ZNF599*	1	3	0	Low expressed	Low expressed	Not expressed
ENSG00000145794	*MEGF10*	1	0	0	Not expressed	Not expressed	Not expressed
ENSG00000105464	*GRIN2D*	1	7	0	Low expressed	Low expressed	Not expressed
ENSG00000120156	*TEK*	0	2	0	Not expressed	Low expressed	Not expressed
ENSG00000189221	*MAOA*	1	47	1	Low expressed	Well expressed	Low expressed
ENSG00000136104	*RNASEH2B*	1	8	3	Low expressed	Low expressed	Low expressed
ENSG00000170927	*PKHD1*	1	0	0	Low expressed	Not expressed	Not expressed
ENSG00000135424	*ITGA7*	1	5	5	Low expressed	Low expressed	Low expressed
ENSG00000129295	*LRRC6*	1	3	3	Low expressed	Low expressed	Low expressed
ENSG00000152669	*CCNO*	1	1	0	Low expressed	Not expressed	Not expressed
ENSG00000139618	*BRCA2*	1	4	0	Not expressed	Low expressed	Not expressed
ENSG00000154864	*PIEZO2*	1	0	0	Not expressed	Not expressed	Not expressed
ENSG00000187790	*FANCM*	1	3	0	Low expressed	Low expressed	Not expressed
ENSG00000197943	*PLCG2*	1	0	26	Low expressed	Not expressed	Well expressed
ENSG00000197061	*H4C3*	1	0	0	Not expressed	Not expressed	Not expressed
ENSG00000143257	*NR1I3*	1	0	0	Low expressed	Not expressed	Not expressed
ENSG00000102452	*NALCN*	1	1	0	Not expressed	Low expressed	Not expressed
ENSG00000181544	*FANCB*	1	3	0	Not expressed	Low expressed	Not expressed
ENSG00000196876	*SCN8A*	1	8	0	Not expressed	Low expressed	Not expressed
ENSG00000105877	*DNAH11*	1	0	0	Low expressed	Not expressed	Not expressed
ENSG00000161681	*SHANK1*	1	1	0	Low expressed	Low expressed	Not expressed
ENSG00000039139	*DNAH5*	1	0	0	Low expressed	Not expressed	Not expressed
ENSG00000108176	*DNAJC12*	1	1	0	Not expressed	Low expressed	Not expressed
ENSG00000162599	*NFIA*	1	6	1	Not expressed	Low expressed	Not expressed
ENSG00000161328	*LRRC56*	1	1	1	Low expressed	Low expressed	Not expressed
ENSG00000073910	*FRY*	1	2	3	Low expressed	Low expressed	Low expressed
ENSG00000171714	*ANO5*	1	0	0	Low expressed	Not expressed	Not expressed
ENSG00000164532	*TBX20*	1	0	0	Not expressed	Not expressed	Not expressed
ENSG00000166341	*DCHS1*	1	4	0	Not expressed	Low expressed	Not expressed
ENSG00000105711	*SCN1B*	1	3	2	Low expressed	Low expressed	Low expressed
ENSG00000021826	*CPS1*	1	14	0	Low expressed	Well expressed	Not expressed
ENSG00000177409	*SAMD9L*	1	8	8	Not expressed	Low expressed	Low expressed
ENSG00000156298	*TSPAN7*	1	0	1	Low expressed	Not expressed	Not expressed
ENSG00000145020	*AMT*	1	7	5	Low expressed	Low expressed	Low expressed
ENSG00000008056	*SYN1*	1	1	1	Low expressed	Not expressed	Not expressed
ENSG00000129675	*ARHGEF6*	1	15	18	Not expressed	Well expressed	Well expressed
ENSG00000135097	*MSI1*	1	0	0	Not expressed	Not expressed	Not expressed
ENSG00000214960	*CRPPA*	1	1	0	Low expressed	Not expressed	Not expressed
ENSG00000144061	*NPHP1*	1	4	0	Low expressed	Low expressed	Not expressed
ENSG00000162687	*KCNT2*	1	1	0	Not expressed	Not expressed	Not expressed
ENSG00000081189	*MEF2C*	1	1	2	Not expressed	Not expressed	Low expressed
ENSG00000001626	*CFTR*	1	0	0	Not expressed	Not expressed	Not expressed
ENSG00000184058	*TBX1*	0	2	0	Not expressed	Low expressed	Not expressed
ENSG00000069431	*ABCC9*	0	17	0	Not expressed	Well expressed	Not expressed
ENSG00000112137	*PHACTR1*	1	2	3	Not expressed	Low expressed	Low expressed
ENSG00000198732	*SMOC1*	1	4	0	Not expressed	Low expressed	Not expressed
ENSG00000143951	*WDPCP*	1	1	0	Not expressed	Low expressed	Not expressed
ENSG00000196557	*CACNA1H*	1	0	0	Not expressed	Not expressed	Not expressed
ENSG00000162004	*CCDC78*	1	2	1	Not expressed	Low expressed	Low expressed

ENSG00000158055	*GRHL3*	1	0	0	Not expressed	Not expressed	Not expressed
ENSG00000139537	*CCDC65*	1	1	1	Not expressed	Low expressed	Not expressed
ENSG00000119686	*FLVCR2*	1	2	6	Not expressed	Low expressed	Low expressed
ENSG00000130675	*MNX1*	1	0	0	Not expressed	Not expressed	Not expressed
ENSG00000152217	*SETBP1*	1	2	0	Not expressed	Low expressed	Not expressed
ENSG00000164418	*GRIK2*	0	0	0	Not expressed	Not expressed	Not expressed
ENSG00000165140	*FBP1*	1	0	27	Not expressed	Not expressed	Well expressed
ENSG00000204815	*TTC25*	1	1	1	Not expressed	Low expressed	Not expressed
ENSG00000111664	*GNB3*	1	6	0	Not expressed	Low expressed	Not expressed
ENSG00000092758	*COL9A3*	1	0	2	Not expressed	Not expressed	Low expressed
ENSG00000113389	*NPR3*	0	11	0	Not expressed	Well expressed	Not expressed
ENSG00000168298	*H1-4*	1	0	3	Not expressed	Not expressed	Low expressed
ENSG00000141519	*CCDC40*	1	1	0	Not expressed	Not expressed	Not expressed
ENSG00000121454	*LHX4*	1	2	1	Not expressed	Low expressed	Not expressed
ENSG00000091262	*ABCC6*	1	0	0	Not expressed	Not expressed	Not expressed
ENSG00000155961	*RAB39B*	1	2	1	Not expressed	Low expressed	Low expressed
ENSG00000171766	*GATM*	1	0	1	Not expressed	Low expressed	Not expressed
ENSG00000138185	*ENTPD1*	1	2	28	Not expressed	Low expressed	Well expressed
ENSG00000185920	*PTCH1*	1	4	0	Not expressed	Low expressed	Not expressed
ENSG00000119866	*BCL11A*	1	0	1	Not expressed	Not expressed	Not expressed
ENSG00000101282	*RSPO4*	0	2	0	Not expressed	Low expressed	Not expressed
ENSG00000125675	*GRIA3*	0	4	0	Not expressed	Low expressed	Not expressed
ENSG00000066468	*FGFR2*	0	0	0	Not expressed	Not expressed	Not expressed
ENSG00000114378	*HYAL1*	1	1	1	Not expressed	Low expressed	Not expressed
ENSG00000087258	*GNAO1*	0	0	0	Not expressed	Not expressed	Not expressed
ENSG00000180509	*KCNE1*	1	0	6	Not expressed	Not expressed	Low expressed
ENSG00000244752	*CRYBB2*	0	0	0	Not expressed	Not expressed	Not expressed
ENSG00000128683	*GAD1*	1	0	0	Not expressed	Not expressed	Not expressed
ENSG00000167646	*DNAAF3*	1	0	0	Not expressed	Not expressed	Not expressed
ENSG00000235718	*MFRP*	1	0	0	Not expressed	Not expressed	Not expressed
ENSG00000198719	*DLL1*	0	1	0	Not expressed	Low expressed	Not expressed
ENSG00000169031	*COL4A3*	0	1	0	Not expressed	Low expressed	Not expressed
ENSG00000140459	*CYP11A1*	1	1	0	Not expressed	Low expressed	Not expressed
ENSG00000132639	*SNAP25*	0	1	0	Not expressed	Low expressed	Not expressed
ENSG00000204103	*MAFB*	1	4	20	Not expressed	Low expressed	Well expressed
ENSG00000150995	*ITPR1*	1	4	1	Not expressed	Low expressed	Low expressed
ENSG00000083307	*GRHL2*	0	0	0	Not expressed	Not expressed	Not expressed
ENSG00000198835	*GJC2*	1	1	0	Not expressed	Low expressed	Not expressed
ENSG00000168280	*KIF5C*	1	0	0	Not expressed	Not expressed	Not expressed
ENSG00000068078	*FGFR3*	0	1	0	Not expressed	Not expressed	Not expressed
ENSG00000138411	*HECW2*	0	1	0	Not expressed	Low expressed	Not expressed
ENSG00000138347	*MYPN*	0	6	0	Not expressed	Low expressed	Not expressed
ENSG00000102349	*KLF8*	1	2	0	Not expressed	Low expressed	Not expressed
ENSG00000077943	*ITGA8*	0	0	0	Not expressed	Not expressed	Not expressed
ENSG00000138435	*CHRNA1*	0	0	0	Not expressed	Not expressed	Not expressed
ENSG00000167371	*PRRT2*	0	4	1	Not expressed	Low expressed	Not expressed
ENSG00000180176	*TH*	0	0	0	Not expressed	Not expressed	Not expressed
ENSG00000147255	*IGSF1*	1	0	0	Not expressed	Not expressed	Not expressed
ENSG00000169946	*ZFPM2*	0	1	0	Not expressed	Not expressed	Not expressed
ENSG00000070808	*CAMK2A*	0	0	0	Not expressed	Not expressed	Not expressed
ENSG00000163581	*SLC2A2*	0	0	0	Not expressed	Not expressed	Not expressed
ENSG00000184156	*KCNQ3*	0	2	0	Not expressed	Low expressed	Not expressed
ENSG00000181804	*SLC9A9*	0	6	3	Not expressed	Low expressed	Low expressed
ENSG00000121207	*LRAT*	0	0	0	Not expressed	Not expressed	Not expressed
ENSG00000158104	*HPD*	0	3	3	Not expressed	Low expressed	Low expressed
ENSG00000196569	*LAMA2*	0	63	0	Not expressed	Well expressed	Not expressed
ENSG00000102935	*ZNF423*	0	1	0	Not expressed	Not expressed	Not expressed
ENSG00000146938	*NLGN4X*	0	0	0	Not expressed	Not expressed	Not expressed
ENSG00000179111	*HES7*	0	1	0	Not expressed	Not expressed	Not expressed
ENSG00000129910	*CDH15*	0	0	0	Not expressed	Not expressed	Not expressed
ENSG00000163666	*HESX1*	0	1	0	Not expressed	Low expressed	Not expressed
ENSG00000102678	*FGF9*	0	0	0	Not expressed	Not expressed	Not expressed
ENSG00000114279	*FGF12*	0	0	0	Not expressed	Not expressed	Not expressed
ENSG00000101115	*SALL4*	0	0	0	Not expressed	Not expressed	Not expressed
ENSG00000150893	*FREM2*	0	0	0	Not expressed	Not expressed	Not expressed
ENSG00000145075	*CCDC39*	0	1	0	Not expressed	Low expressed	Not expressed
ENSG00000187391	*MAGI2*	0	1	0	Not expressed	Not expressed	Not expressed
ENSG00000184908	*CLCNKB*	0	0	0	Not expressed	Not expressed	Not expressed
ENSG00000112320	*SOBP*	0	2	0	Not expressed	Low expressed	Not expressed
ENSG00000131398	*KCNC3*	0	0	1	Not expressed	Not expressed	Not expressed
ENSG00000182901	*RGS7*	0	2	0	Not expressed	Low expressed	Not expressed
ENSG00000167723	*TRPV3*	0	1	0	Not expressed	Not expressed	Not expressed
ENSG00000107099	*DOCK8*	0	0	26	Not expressed	Not expressed	Well expressed
ENSG00000043355	*ZIC2*	0	0	0	Not expressed	Not expressed	Not expressed
ENSG00000092295	*TGM1*	0	1	1	Not expressed	Not expressed	Not expressed
ENSG00000122367	*LDB3*	0	0	0	Not expressed	Not expressed	Not expressed
ENSG00000131095	*GFAP*	0	0	1	Not expressed	Not expressed	Not expressed
ENSG00000182578	*CSF1R*	0	0	30	Not expressed	Not expressed	Well expressed
ENSG00000143603	*KCNN3*	0	0	0	Not expressed	Not expressed	Not expressed
ENSG00000137745	*MMP13*	0	0	0	Not expressed	Not expressed	Not expressed
ENSG00000134532	*SOX5*	0	0	0	Not expressed	Not expressed	Not expressed
ENSG00000184374	*COLEC10*	0	0	0	Not expressed	Not expressed	Not expressed
ENSG00000154099	*DNAAF1*	0	0	0	Not expressed	Not expressed	Not expressed
ENSG00000133392	*MYH11*	0	2	2	Not expressed	Low expressed	Low expressed
ENSG00000124140	*SLC12A5*	0	0	0	Not expressed	Not expressed	Not expressed
ENSG00000102878	*HSF4*	0	6	3	Not expressed	Low expressed	Low expressed
ENSG00000158813	*EDA*	0	1	0	Not expressed	Not expressed	Not expressed
ENSG00000109339	*MAPK10*	0	0	0	Not expressed	Not expressed	Not expressed
ENSG00000204248	*COL11A2*	0	1	0	Not expressed	Not expressed	Not expressed
ENSG00000200463	*SNORD118*	0	No data	No data	Not expressed	No data	No data
ENSG00000156049	*GNA14*	0	0	0	Not expressed	Not expressed	Not expressed
ENSG00000168453	*HR*	0	3	0	Not expressed	Low expressed	Not expressed
ENSG00000169071	*ROR2*	0	10	0	Not expressed	Low expressed	Not expressed

ENSG00000160202	*CRYAA*	0	0	0	Not expressed	Not expressed	Not expressed
ENSG00000007372	*PAX6*	0	0	0	Not expressed	Not expressed	Not expressed
ENSG00000215417	*MIR17HG*	0	2	0	Not expressed	Low expressed	Not expressed
ENSG00000169884	*WNT10B*	0	0	2	Not expressed	Not expressed	Low expressed
ENSG00000160111	*CPAMD8*	0	0	1	Not expressed	Not expressed	Not expressed
ENSG00000182621	*PLCB1*	0	0	0	Not expressed	Not expressed	Not expressed
ENSG00000128573	*FOXP2*	0	1	0	Not expressed	Not expressed	Not expressed
ENSG00000109063	*MYH3*	0	1	1	Not expressed	Low expressed	Not expressed
ENSG00000121743	*GJA3*	0	0	0	Not expressed	Not expressed	Not expressed
ENSG00000180660	*MAB21L1*	0	2	0	Not expressed	Low expressed	Not expressed
ENSG00000254585	*MAGEL2*	0	0	0	Not expressed	Not expressed	Not expressed
ENSG00000069667	*RORA*	0	5	2	Not expressed	Low expressed	Low expressed
ENSG00000157423	*HYDIN*	0	0	0	Not expressed	Not expressed	Not expressed
ENSG00000183785	*TUBA8*	0	0	0	Not expressed	Not expressed	Not expressed
ENSG00000215417	*MIR17HG*	0	2	2	Not expressed	Low expressed	Low expressed
ENSG00000143627	*PKLR*	0	0	0	Not expressed	Not expressed	Not expressed
ENSG00000136928	*GABBR2*	0	3	0	Not expressed	Low expressed	Not expressed
ENSG00000174672	*BRSK2*	0	0	0	Not expressed	Not expressed	Not expressed
ENSG00000180318	*ALX1*	0	1	0	Not expressed	Not expressed	Not expressed
ENSG00000136531	*SCN2A*	0	0	0	Not expressed	Not expressed	Not expressed
ENSG00000196338	*NLGN3*	0	1	3	Not expressed	Not expressed	Low expressed
ENSG00000116690	*PRG4*	0	1	0	Not expressed	Not expressed	Not expressed
ENSG00000115266	*APC2*	0	0	0	Not expressed	Not expressed	Not expressed
ENSG00000160188	*RSPH1*	0	0	0	Not expressed	Not expressed	Not expressed
ENSG00000166206	*GABRB3*	0	0	0	Not expressed	Not expressed	Not expressed
ENSG00000260230	*FRRS1L*	0	0	0	Not expressed	Not expressed	Not expressed
ENSG00000165194	*PCDH19*	0	0	0	Not expressed	Not expressed	Not expressed
ENSG00000140873	*ADAMTS18*	0	0	0	Not expressed	Not expressed	Not expressed
ENSG00000159640	*ACE*	0	6	0	Not expressed	Low expressed	Not expressed
ENSG00000169427	*KCNK9*	0	0	0	Not expressed	Not expressed	Not expressed
ENSG00000134323	*MYCN*	0	0	0	Not expressed	Not expressed	Not expressed
ENSG00000198807	*PAX9*	0	2	0	Not expressed	Low expressed	Not expressed
ENSG00000153253	*SCN3A*	0	0	0	Not expressed	Not expressed	Not expressed
ENSG00000157404	*KIT*	0	0	0	Not expressed	Not expressed	Not expressed
ENSG00000188095	*MESP2*	0	0	0	Not expressed	Not expressed	Not expressed
ENSG00000135917	*SLC19A3*	0	1	0	Not expressed	Not expressed	Not expressed
ENSG00000143839	*REN*	0	0	0	Not expressed	Not expressed	Not expressed
ENSG00000149922	*TBX6*	0	1	1	Not expressed	Not expressed	Not expressed
ENSG00000162552	*WNT4*	0	0	0	Not expressed	Not expressed	Not expressed
ENSG00000187323	*DCC*	0	0	0	Not expressed	Not expressed	Not expressed
ENSG00000102003	*SYP*	0	1	1	Not expressed	Not expressed	Not expressed
ENSG00000004838	*ZMYND10*	0	0	1	Not expressed	Not expressed	Not expressed
ENSG00000164007	*CLDN19*	0	0	0	Not expressed	Not expressed	Not expressed
ENSG00000164946	*FREM1*	0	0	0	Not expressed	Not expressed	Not expressed
ENSG00000178538	*CA8*	0	0	0	Not expressed	Not expressed	Not expressed
ENSG00000049768	*FOXP3*	0	0	2	Not expressed	Not expressed	Low expressed
ENSG00000141431	*ASXL3*	0	0	0	Not expressed	Not expressed	Not expressed
ENSG00000196220	*SRGAP3*	0	0	0	Not expressed	Not expressed	Not expressed
ENSG00000124479	*NDP*	0	0	0	Not expressed	Not expressed	Not expressed
ENSG00000169306	*IL1RAPL1*	0	0	0	Not expressed	Not expressed	Not expressed
ENSG00000198003	*CCDC151*	0	0	0	Not expressed	Not expressed	Not expressed
ENSG00000198838	*RYR3*	0	0	0	Not expressed	Not expressed	Not expressed
ENSG00000123500	*COL10A1*	0	3	0	Not expressed	Low expressed	Not expressed
ENSG00000105664	*COMP*	0	5	0	Not expressed	Low expressed	Not expressed
ENSG00000176165	*FOXG1*	0	0	0	Not expressed	Not expressed	Not expressed
ENSG00000169083	*AR*	0	15	0	Not expressed	Well expressed	Not expressed
ENSG00000101276	*SLC52A3*	0	0	0	Not expressed	Not expressed	Not expressed
ENSG00000053918	*KCNQ1*	0	0	13	Not expressed	Not expressed	Well expressed
ENSG00000066032	*CTNNA2*	0	0	0	Not expressed	Not expressed	Not expressed
ENSG00000239474	*KLHL41*	0	1	0	Not expressed	Not expressed	Not expressed
ENSG00000112964	*GHR*	0	6	0	Not expressed	Low expressed	Not expressed
ENSG00000037280	*FLT4*	0	0	1	Not expressed	Not expressed	Not expressed
ENSG00000130711	*PRDM12*	0	0	0	Not expressed	Not expressed	Not expressed
ENSG00000264229	*RNU4ATAC*	0	0	0	Not expressed	Not expressed	Not expressed
ENSG00000176884	*GRIN1*	0	0	0	Not expressed	Not expressed	Not expressed
ENSG00000105479	*CCDC114*	0	0	0	Not expressed	Not expressed	Not expressed
ENSG00000187726	*DNAJB13*	0	0	0	Not expressed	Not expressed	Not expressed
ENSG00000165731	*RET*	0	0	0	Not expressed	Not expressed	Not expressed
ENSG00000187486	*KCNJ11*	0	0	0	Not expressed	Not expressed	Not expressed
ENSG00000155657	*TTN*	0	0	0	Not expressed	Not expressed	Not expressed
ENSG00000102383	*ZDHHC15*	0	0	0	Not expressed	Not expressed	Not expressed
ENSG00000147465	*STAR*	0	1	0	Not expressed	Not expressed	Not expressed
ENSG00000160716	*CHRNB2*	0	0	0	Not expressed	Not expressed	Not expressed
ENSG00000146469	*VIP*	0	0	0	Not expressed	Not expressed	Not expressed
ENSG00000127241	*MASP1*	0	13	0	Not expressed	Well expressed	Not expressed
ENSG00000197106	*SLC6A17*	0	0	0	Not expressed	Not expressed	Not expressed
ENSG00000148408	*CACNA1B*	0	0	0	Not expressed	Not expressed	Not expressed
ENSG00000156574	*NODAL*	0	0	0	Not expressed	Not expressed	Not expressed
ENSG00000157388	*CACNA1D*	0	0	0	Not expressed	Not expressed	Not expressed
ENSG00000137474	*MYO7A*	0	0	1	Not expressed	Not expressed	Low expressed
ENSG00000122787	*AKR1D1*	0	0	0	Not expressed	Not expressed	Not expressed
ENSG00000049540	*ELN*	0	9	0	Not expressed	Low expressed	Not expressed
ENSG00000136160	*EDNRB*	0	4	0	Not expressed	Low expressed	Not expressed
ENSG00000185467	*KPNA7*	0	0	0	Not expressed	Not expressed	Not expressed
ENSG00000165917	*RAPSN*	0	0	1	Not expressed	Not expressed	Not expressed
ENSG00000160224	*AIRE*	0	0	0	Not expressed	Not expressed	Not expressed
ENSG00000183454	*GRIN2A*	0	0	0	Not expressed	Not expressed	Not expressed
ENSG00000104313	*EYA1*	0	0	0	Not expressed	Not expressed	Not expressed
ENSG00000153283	*CD96*	0	0	8	Not expressed	Not expressed	Low expressed
ENSG00000157856	*DRC1*	0	0	0	Not expressed	Not expressed	Not expressed
ENSG00000155966	*AFF2*	0	0	0	Not expressed	Not expressed	Not expressed
ENSG00000183091	*NEB*	0	0	0	Not expressed	Not expressed	Not expressed
ENSG00000140470	*ADAMTS17*	0	0	0	Not expressed	Not expressed	Not expressed

ENSG00000092607	*TBX15*	0	25	0	Not expressed	Well expressed	Not expressed
ENSG00000050555	*LAMC3*	0	0	0	Not expressed	Not expressed	Not expressed
ENSG00000116039	*ATP6V1B1*	0	2	0	Not expressed	Low expressed	Not expressed
ENSG00000108823	*SGCA*	0	1	0	Not expressed	Not expressed	Not expressed
ENSG00000105409	*ATP1A3*	0	0	1	Not expressed	Not expressed	Low expressed
ENSG00000169933	*FRMPD4*	0	2	0	Not expressed	Low expressed	Not expressed
ENSG00000073282	*TP63*	0	0	0	Not expressed	Not expressed	Not expressed
ENSG00000155962	*CLIC2*	0	3	2	Not expressed	Low expressed	Low expressed
ENSG00000135960	*EDAR*	0	0	0	Not expressed	Not expressed	Not expressed
ENSG00000103723	*AP3B2*	0	0	1	Not expressed	Not expressed	Not expressed
ENSG00000112562	*SMOC2*	0	0	0	Not expressed	Not expressed	Not expressed
ENSG00000061455	*PRDM6*	0	0	0	Not expressed	Not expressed	Not expressed
ENSG00000108001	*EBF3*	0	4	0	Not expressed	Low expressed	Not expressed
ENSG00000109705	*NKX3-2*	0	0	0	Not expressed	Not expressed	Not expressed
ENSG00000104967	*NOVA2*	0	0	0	Not expressed	Not expressed	Not expressed
ENSG00000118004	*COLEC11*	0	0	0	Not expressed	Not expressed	Not expressed
ENSG00000179774	*ATOH7*	0	0	0	Not expressed	Not expressed	Not expressed
ENSG00000168477	*TNXB*	0	4	0	Not expressed	Low expressed	Not expressed
ENSG00000113262	*GRM6*	0	0	0	Not expressed	Not expressed	Not expressed
ENSG00000116218	*NPHS2*	0	0	0	Not expressed	Not expressed	Not expressed
ENSG00000157542	*KCNJ6*	0	0	0	Not expressed	Not expressed	Not expressed
ENSG00000149970	*CNKSR2*	0	0	0	Not expressed	Not expressed	Not expressed
ENSG00000118231	*CRYGD*	0	0	0	Not expressed	Not expressed	Not expressed
ENSG00000128917	*DLL4*	0	0	0	Not expressed	Not expressed	Not expressed
ENSG00000186510	*CLCNKA*	0	0	0	Not expressed	Not expressed	Not expressed
ENSG00000108950	*FAM20A*	0	9	4	Not expressed	Low expressed	Low expressed
ENSG00000121552	*CSTA*	0	11	42	Not expressed	Well expressed	Well expressed
ENSG00000168356	*SCN11A*	0	0	0	Not expressed	Not expressed	Not expressed
ENSG00000182389	*CACNB4*	0	0	0	Not expressed	Not expressed	Not expressed
ENSG00000165125	*TRPV6*	0	0	0	Not expressed	Not expressed	Not expressed
ENSG00000188937	*NYX*	0	0	0	Not expressed	Not expressed	Not expressed
ENSG00000151067	*CACNA1C*	0	6	0	Not expressed	Low expressed	Not expressed
ENSG00000148795	*CYP17A1*	0	0	0	Not expressed	Not expressed	Not expressed
ENSG00000148053	*NTRK2*	0	0	0	Not expressed	Not expressed	Not expressed
ENSG00000167941	*SOST*	0	0	0	Not expressed	Not expressed	Not expressed
ENSG00000277893	*SRD5A2*	0	0	0	Not expressed	Not expressed	Not expressed
ENSG00000183423	*LRIT3*	0	0	0	Not expressed	Not expressed	Not expressed
ENSG00000130948	*HSD17B3*	0	0	0	Not expressed	Not expressed	Not expressed
ENSG00000163380	*LMOD3*	0	0	0	Not expressed	Not expressed	Not expressed
ENSG00000160282	*FTCD*	0	0	0	Not expressed	Not expressed	Not expressed
ENSG00000143768	*LEFTY2*	0	0	0	Not expressed	Not expressed	Not expressed
ENSG00000198400	*NTRK1*	0	0	0	Not expressed	Not expressed	Not expressed
ENSG00000058404	*CAMK2B*	0	0	0	Not expressed	Not expressed	Not expressed
ENSG00000141837	*CACNA1A*	0	1	0	Not expressed	Low expressed	Not expressed
ENSG00000107165	*TYRP1*	0	0	0	Not expressed	Not expressed	Not expressed
ENSG00000165186	*PTCHD1*	0	0	0	Not expressed	Not expressed	Not expressed
ENSG00000231852	*CYP21A2*	0	0	0	Not expressed	Not expressed	Not expressed
ENSG00000169918	*OTUD7A*	0	0	0	Not expressed	Not expressed	Not expressed
ENSG00000273079	*GRIN2B*	0	0	0	Not expressed	Not expressed	Not expressed
ENSG00000116014	*KISS1R*	0	0	0	Not expressed	Not expressed	Not expressed
ENSG00000052850	*ALX4*	0	0	0	Not expressed	Not expressed	Not expressed
ENSG00000141579	*ZNF750*	0	0	0	Not expressed	Not expressed	Not expressed
ENSG00000006283	*CACNA1G*	0	0	0	Not expressed	Not expressed	Not expressed
ENSG00000100053	*CRYBB3*	0	0	0	Not expressed	Not expressed	Not expressed
ENSG00000158445	*KCNB1*	0	0	0	Not expressed	Not expressed	Not expressed
ENSG00000139219	*COL2A1*	0	0	0	Not expressed	Not expressed	Not expressed
ENSG00000186790	*FOXE3*	0	0	0	Not expressed	Not expressed	Not expressed
ENSG00000154485	*MMP21*	0	0	0	Not expressed	Not expressed	Not expressed
ENSG00000169783	*LINGO1*	0	0	0	Not expressed	Not expressed	Not expressed
ENSG00000187045	*TMPRSS6*	0	0	0	Not expressed	Not expressed	Not expressed
ENSG00000141485	*SLC13A5*	0	0	0	Not expressed	Not expressed	Not expressed
ENSG00000171551	*ECEL1*	0	0	0	Not expressed	Not expressed	Not expressed
ENSG00000152578	*GRIA4*	0	0	0	Not expressed	Not expressed	Not expressed
ENSG00000182450	*KCNK4*	0	0	0	Not expressed	Not expressed	Not expressed
ENSG00000108255	*CRYBA1*	0	0	0	Not expressed	Not expressed	Not expressed
ENSG00000152822	*GRM1*	0	0	0	Not expressed	Not expressed	Not expressed
ENSG00000120094	*HOXB1*	0	0	0	Not expressed	Not expressed	Not expressed
ENSG00000136352	*NKX2-1*	0	0	0	Not expressed	Not expressed	Not expressed
ENSG00000134200	*TSHB*	0	0	0	Not expressed	Not expressed	Not expressed
ENSG00000118520	*ARG1*	0	0	141	Not expressed	Not expressed	Well expressed
ENSG00000165899	*OTOGL*	0	0	0	Not expressed	Not expressed	Not expressed
ENSG00000107831	*FGF8*	0	0	0	Not expressed	Not expressed	Not expressed
ENSG00000007314	*SCN4A*	0	0	0	Not expressed	Not expressed	Not expressed
ENSG00000184895	*SRY*	0	2	0	Not expressed	Low expressed	Not expressed
ENSG00000070019	*GUCY2C*	0	0	0	Not expressed	Not expressed	Not expressed
ENSG00000158683	*PKD1L1*	0	0	0	Not expressed	Not expressed	Not expressed
ENSG00000196218	*RYR1*	0	0	0	Not expressed	Not expressed	Not expressed
ENSG00000205899	*BHLHA9*	0	0	0	Not expressed	Not expressed	Not expressed
ENSG00000130598	*TNNI2*	0	0	18	Not expressed	Not expressed	Well expressed
ENSG00000122735	*DNAI1*	0	0	0	Not expressed	Not expressed	Not expressed
ENSG00000136872	*ALDOB*	0	0	0	Not expressed	Not expressed	Not expressed
ENSG00000134376	*CRB1*	0	0	0	Not expressed	Not expressed	Not expressed
ENSG00000123364	*HOXC13*	0	2	0	Not expressed	Low expressed	Not expressed
ENSG00000157103	*SLC6A1*	0	0	0	Not expressed	Not expressed	Not expressed
ENSG00000177807	*KCNJ10*	0	0	0	Not expressed	Not expressed	Not expressed
ENSG00000151704	*KCNJ1*	0	0	0	Not expressed	Not expressed	Not expressed
ENSG00000169836	*TACR3*	0	0	0	Not expressed	Not expressed	Not expressed
ENSG00000139053	*PDE6H*	0	0	0	Not expressed	Not expressed	Not expressed
ENSG00000181449	*SOX2*	0	0	0	Not expressed	Not expressed	Not expressed
ENSG00000153902	*LGI4*	0	1	0	Not expressed	Not expressed	Not expressed
ENSG00000196132	*MYT1*	0	0	0	Not expressed	Not expressed	Not expressed
ENSG00000089225	*TBX5*	0	2	0	Not expressed	Low expressed	Not expressed
ENSG00000110076	*NRXN2*	0	1	0	Not expressed	Low expressed	Not expressed

ENSG00000151224	*MAT1A*	0	0	0	Not expressed	Not expressed	Not expressed
ENSG00000196811	*CHRNG*	0	0	0	Not expressed	Not expressed	Not expressed
ENSG00000110436	*SLC1A2*	0	0	0	Not expressed	Not expressed	Not expressed
ENSG00000197859	*ADAMTSL2*	0	0	0	Not expressed	Not expressed	Not expressed
ENSG00000064835	*POU1F1*	0	0	0	Not expressed	Not expressed	Not expressed
ENSG00000198650	*TAT*	0	0	0	Not expressed	Not expressed	Not expressed
ENSG00000108231	*LGI1*	0	0	0	Not expressed	Not expressed	Not expressed
ENSG00000123560	*PLP1*	0	2	0	Not expressed	Low expressed	Not expressed
ENSG00000172987	*HPSE2*	0	0	0	Not expressed	Not expressed	Not expressed
ENSG00000129159	*KCNC1*	0	0	0	Not expressed	Not expressed	Not expressed
ENSG00000163254	*CRYGC*	0	0	0	Not expressed	Not expressed	Not expressed
ENSG00000022355	*GABRA1*	0	0	0	Not expressed	Not expressed	Not expressed
ENSG00000163421	*PROK2*	0	0	210	Not expressed	Not expressed	Well expressed
ENSG00000232258	*TMEM114*	0	0	0	Not expressed	Not expressed	Not expressed
ENSG00000139352	*ASCL1*	0	0	0	Not expressed	Not expressed	Not expressed
ENSG00000105610	*KLF1*	0	0	22	Not expressed	Not expressed	Well expressed
ENSG00000168148	*H3-4*	0	0	0	Not expressed	Not expressed	Not expressed
ENSG00000183072	*NKX2-5*	0	0	0	Not expressed	Not expressed	Not expressed
ENSG00000100146	*SOX10*	0	No data	No data	Not expressed	No data	No data
ENSG00000128714	*HOXD13*	0	0	0	Not expressed	Not expressed	Not expressed
ENSG00000092200	*RPGRIP1*	0	0	1	Not expressed	Not expressed	Not expressed
ENSG00000163501	*IHH*	0	0	0	Not expressed	Not expressed	Not expressed
ENSG00000274286	*ADRA2B*	0	0	0	Not expressed	Not expressed	Not expressed
ENSG00000007174	*DNAH9*	0	0	0	Not expressed	Not expressed	Not expressed
ENSG00000196431	*CRYBA4*	0	0	0	Not expressed	Not expressed	Not expressed
ENSG00000171487	*NLRP5*	0	0	0	Not expressed	Not expressed	Not expressed
ENSG00000017427	*IGF1*	0	0	0	Not expressed	Not expressed	Not expressed
ENSG00000107736	*CDH23*	0	0	4	Not expressed	Not expressed	Low expressed
ENSG00000050030	*NEXMIF*	0	0	0	Not expressed	Not expressed	Not expressed
ENSG00000074803	*SLC12A1*	0	0	0	Not expressed	Not expressed	Not expressed
ENSG00000182255	*KCNA4*	0	0	0	Not expressed	Not expressed	Not expressed
ENSG00000161270	*NPHS1*	0	0	0	Not expressed	Not expressed	Not expressed
ENSG00000165409	*TSHR*	0	0	0	Not expressed	Not expressed	Not expressed
ENSG00000168955	*TM4SF20*	0	1	0	Not expressed	Not expressed	Not expressed
ENSG00000145864	*GABRB2*	0	0	0	Not expressed	Not expressed	Not expressed
ENSG00000165694	*FRMD7*	0	0	0	Not expressed	Not expressed	Not expressed
ENSG00000136535	*TBR1*	0	0	0	Not expressed	Not expressed	Not expressed
ENSG00000106031	*HOXA13*	0	0	0	Not expressed	Not expressed	Not expressed
ENSG00000135903	*PAX3*	0	1	0	Not expressed	Low expressed	Not expressed
ENSG00000121075	*TBX4*	0	1	0	Not expressed	Not expressed	Not expressed
ENSG00000107147	*KCNT1*	0	0	0	Not expressed	Not expressed	Not expressed
ENSG00000270765	*GAS2L2*	0	0	0	Not expressed	Not expressed	Not expressed
ENSG00000180316	*PNPLA1*	0	0	3	Not expressed	Not expressed	Low expressed
ENSG00000081479	*LRP2*	0	0	0	Not expressed	Not expressed	Not expressed
ENSG00000108381	*ASPA*	0	1	0	Not expressed	Low expressed	Not expressed
ENSG00000132437	*DDC*	0	0	0	Not expressed	Not expressed	Not expressed
ENSG00000144285	*SCN1A*	0	0	0	Not expressed	Not expressed	Not expressed
ENSG00000105641	*SLC5A5*	0	0	0	Not expressed	Not expressed	Not expressed
ENSG00000100122	*CRYBB1*	0	0	0	Not expressed	Not expressed	Not expressed
ENSG00000157766	*ACAN*	0	0	0	Not expressed	Not expressed	Not expressed
ENSG00000077498	*TYR*	0	0	0	Not expressed	Not expressed	Not expressed
ENSG00000170819	*BFSP2*	0	0	0	Not expressed	Not expressed	Not expressed
ENSG00000159650	*UROC1*	0	0	0	Not expressed	Not expressed	Not expressed
ENSG00000136574	*GATA4*	0	0	0	Not expressed	Not expressed	Not expressed
ENSG00000122145	*TBX22*	0	0	0	Not expressed	Not expressed	Not expressed
ENSG00000105880	*DLX5*	0	0	0	Not expressed	Not expressed	Not expressed
ENSG00000030304	*MUSK*	0	1	0	Not expressed	Low expressed	Not expressed
ENSG00000111249	*CUX2*	0	0	0	Not expressed	Not expressed	Not expressed
ENSG00000142319	*SLC6A3*	0	0	0	Not expressed	Not expressed	Not expressed
ENSG00000152592	*DMP1*	0	0	0	Not expressed	Not expressed	Not expressed
ENSG00000004848	*ARX*	0	0	0	Not expressed	Not expressed	Not expressed
ENSG00000116745	*RPE65*	0	0	0	Not expressed	Not expressed	Not expressed
ENSG00000162399	*BSND*	0	0	0	Not expressed	Not expressed	Not expressed
ENSG00000206013	*IFITM5*	0	0	0	Not expressed	Not expressed	Not expressed
ENSG00000134760	*DSG1*	0	0	0	Not expressed	Not expressed	Not expressed
ENSG00000134160	*TRPM1*	0	0	0	Not expressed	Not expressed	Not expressed
ENSG00000125084	*WNT1*	0	0	0	Not expressed	Not expressed	Not expressed
ENSG00000121634	*GJA8*	0	0	0	Not expressed	Not expressed	Not expressed
ENSG00000034971	*MYOC*	0	0	0	Not expressed	Not expressed	Not expressed
ENSG00000174990	*CA5A*	0	0	0	Not expressed	Not expressed	Not expressed
ENSG00000186417	*GLDN*	0	2	0	Not expressed	Low expressed	Not expressed
ENSG00000156925	*ZIC3*	0	0	0	Not expressed	Not expressed	Not expressed
ENSG00000080572	*DNAAF6*	0	0	0	Not expressed	Not expressed	Not expressed
ENSG00000004939	*SLC4A1*	0	0	79	Not expressed	Not expressed	Well expressed
ENSG00000105392	*CRX*	0	0	0	Not expressed	Not expressed	Not expressed
ENSG00000073734	*ABCB11*	0	0	0	Not expressed	Not expressed	Not expressed
ENSG00000133020	*MYH8*	0	0	0	Not expressed	Not expressed	Not expressed
ENSG00000134595	*SOX3*	0	0	0	Not expressed	Not expressed	Not expressed
ENSG00000164362	*TERT*	0	0	0	Not expressed	Not expressed	Not expressed
ENSG00000077279	*DCX*	0	0	0	Not expressed	Not expressed	Not expressed
ENSG00000140015	*KCNH5*	0	0	0	Not expressed	Not expressed	Not expressed
ENSG00000152591	*DSPP*	0	0	0	Not expressed	Not expressed	Not expressed
ENSG00000179915	*NRXN1*	0	0	0	Not expressed	Not expressed	Not expressed
ENSG00000108821	*COL1A1*	781	3664	1	Well expressed	Well expressed	Not expressed
ENSG00000152977	*ZIC1*	0	8	0	Not expressed	Low expressed	Not expressed
ENSG00000100427	*MLC1*	0	0	5	Not expressed	Not expressed	Low expressed
ENSG00000147655	*RSPO2*	0	0	0	Not expressed	Not expressed	Not expressed
ENSG00000138083	*SIX3*	0	0	0	Not expressed	Not expressed	Not expressed
ENSG00000185960	*SHOX*	0	0	0	Not expressed	Not expressed	Not expressed
ENSG00000109101	*FOXN1*	0	0	0	Not expressed	Not expressed	Not expressed
ENSG00000177301	*KCNA2*	0	0	0	Not expressed	Not expressed	Not expressed
ENSG00000166863	*TAC3*	0	0	0	Not expressed	Not expressed	Not expressed
ENSG00000101076	*HNF4A*	0	0	0	Not expressed	Not expressed	Not expressed

ENSG00000136931	*NR5A1*	0	0	0	Not expressed	Not expressed	Not expressed
ENSG00000140090	*SLC24A4*	0	0	4	Not expressed	Not expressed	Low expressed
ENSG00000077080	*ACTL6B*	0	0	0	Not expressed	Not expressed	Not expressed
ENSG00000144406	*UNC80*	0	0	0	Not expressed	Not expressed	Not expressed
ENSG00000111049	*MYF5*	0	0	0	Not expressed	Not expressed	Not expressed
ENSG00000091138	*SLC26A3*	0	0	0	Not expressed	Not expressed	Not expressed
ENSG00000152266	*PTH*	0	0	0	Not expressed	Not expressed	Not expressed
ENSG00000163508	*EOMES*	0	0	5	Not expressed	Not expressed	Low expressed
ENSG00000129221	*AIPL1*	0	0	0	Not expressed	Not expressed	Not expressed
ENSG00000186487	*MYT1L*	0	0	0	Not expressed	Not expressed	Not expressed
ENSG00000171759	*PAH*	0	0	0	Not expressed	Not expressed	Not expressed
ENSG00000075043	*KCNQ2*	0	0	0	Not expressed	Not expressed	Not expressed
ENSG00000101292	*PROKR2*	0	0	0	Not expressed	Not expressed	Not expressed
ENSG00000107187	*LHX3*	0	0	0	Not expressed	Not expressed	Not expressed
ENSG00000175325	*PROP1*	0	0	0	Not expressed	Not expressed	Not expressed
ENSG00000070193	*FGF10*	0	0	0	Not expressed	Not expressed	Not expressed
ENSG00000120903	*CHRNA2*	0	0	0	Not expressed	Not expressed	Not expressed
ENSG00000135902	*CHRND*	0	0	0	Not expressed	Not expressed	Not expressed
ENSG00000036473	*OTC*	0	0	0	Not expressed	Not expressed	Not expressed
ENSG00000070748	*CHAT*	0	0	0	Not expressed	Not expressed	Not expressed
ENSG00000134438	*RAX*	0	0	0	Not expressed	Not expressed	Not expressed
ENSG00000109906	*ZBTB16*	0	31	7	Not expressed	Well expressed	Low expressed
ENSG00000101204	*CHRNA4*	0	0	0	Not expressed	Not expressed	Not expressed
ENSG00000119614	*VSX2*	0	0	0	Not expressed	Not expressed	Not expressed
ENSG00000179270	*PCARE*	0	0	0	Not expressed	Not expressed	Not expressed
ENSG00000157119	*KLHL40*	0	0	0	Not expressed	Not expressed	Not expressed
ENSG00000164588	*HCN1*	0	0	0	Not expressed	Not expressed	Not expressed
ENSG00000134240	*HMGCS2*	0	0	0	Not expressed	Not expressed	Not expressed
ENSG00000234438	*KBTBD13*	0	0	0	Not expressed	Not expressed	Not expressed
ENSG00000170484	*KRT74*	0	0	0	Not expressed	Not expressed	Not expressed
ENSG00000184302	*SIX6*	0	0	0	Not expressed	Not expressed	Not expressed
ENSG00000198216	*CACNA1E*	0	0	2	Not expressed	Not expressed	Low expressed
ENSG00000165588	*OTX2*	0	0	0	Not expressed	Not expressed	Not expressed
ENSG00000113327	*GABRG2*	0	0	0	Not expressed	Not expressed	Not expressed
ENSG00000006071	*ABCC8*	0	0	0	Not expressed	Not expressed	Not expressed
ENSG00000112280	*COL9A1*	0	0	0	Not expressed	Not expressed	Not expressed
ENSG00000165970	*SLC6A5*	0	0	0	Not expressed	Not expressed	Not expressed
ENSG00000186895	*FGF3*	0	0	0	Not expressed	Not expressed	Not expressed
ENSG00000207695	*MIR184*	0	0	0	Not expressed	Not expressed	Not expressed
ENSG00000122406	*RPL5*	651	2399	308	Well expressed	Well expressed	Well expressed
ENSG00000215612	*HMX1*	0	0	0	Not expressed	Not expressed	Not expressed
ENSG00000237412	*PRSS56*	0	0	0	Not expressed	Not expressed	Not expressed
ENSG00000167104	*BPIFB6*	0	0	0	Not expressed	Not expressed	Not expressed
ENSG00000142173	*COL6A2*	216	2275	7	Well expressed	Well expressed	Low expressed
ENSG00000142156	*COL6A1*	151	2264	2	Well expressed	Well expressed	Low expressed
ENSG00000109132	*PHOX2B*	0	0	0	Not expressed	Not expressed	Not expressed
ENSG00000185002	*RFX6*	0	0	0	Not expressed	Not expressed	Not expressed
ENSG00000115665	*SLC5A7*	0	0	0	Not expressed	Not expressed	Not expressed
ENSG00000180772	*AGTR2*	0	0	0	Not expressed	Not expressed	Not expressed
ENSG00000184344	*GDF3*	0	0	0	Not expressed	Not expressed	Not expressed
ENSG00000113140	*SPARC*	1026	1598	33	Well expressed	Well expressed	Well expressed
ENSG00000128610	*FEZF1*	0	0	0	Not expressed	Not expressed	Not expressed
ENSG00000185231	*MC2R*	0	0	0	Not expressed	Not expressed	Not expressed
ENSG00000166794	*PPIB*	921	1306	205	Well expressed	Well expressed	Well expressed
ENSG00000185624	*P4HB*	615	1253	160	Well expressed	Well expressed	Well expressed
ENSG00000181163	*NPM1*	697	1222	55	Well expressed	Well expressed	Well expressed
ENSG00000124614	*RPS10*	950	1208	235	Well expressed	Well expressed	Well expressed
ENSG00000163359	*COL6A3*	23	1201	0	Well expressed	Well expressed	Not expressed
ENSG00000142676	*RPL11*	667	1193	150	Well expressed	Well expressed	Well expressed
ENSG00000114942	*EEF1B2*	661	1148	72	Well expressed	Well expressed	Well expressed
ENSG00000197746	*PSAP*	1089	1126	1316	Well expressed	Well expressed	Well expressed
ENSG00000168267	*PTF1A*	0	0	0	Not expressed	Not expressed	Not expressed
ENSG00000182774	*RPS17*	767	1111	81	Well expressed	Well expressed	Well expressed
ENSG00000204020	*LIPN*	0	0	7	Not expressed	Not expressed	Low expressed
ENSG00000240505	*TNFRSF13B*	0	0	1	Not expressed	Not expressed	Low expressed
ENSG00000136698	*CFC1*	0	0	0	Not expressed	Not expressed	Not expressed
ENSG00000172482	*AGXT*	0	0	0	Not expressed	Not expressed	Not expressed
ENSG00000197616	*MYH6*	0	0	0	Not expressed	Not expressed	Not expressed
ENSG00000196091	*MYBPC1*	0	0	0	Not expressed	Not expressed	Not expressed
ENSG00000160882	*CYP11B1*	0	0	0	Not expressed	Not expressed	Not expressed

Supplementary Table 6. Results of the functional enrichment analysis of the 81 genes that were well-expressed (TPM values ≥ 10) in AF cells but not expressed (TPM values < 1) or lowly expressed (1 ≤ TMP < 10) in the whole blood and skin fibroblasts.

GroupID	Category	Term	Description	p-value	q-value	In Term / In List	Genes
1_Summary	GO Biological Processes	GO:0048598	embryonic morphogenesis	2.28E-08	1.76E-06	21/576	ALDH1A3 CDKN1C EDN1 FOXC1 GATA6 ITGB4 PAX2 SALL1 SMO SOX4 SOX9 SOX11 ST14 HNF1B TGFB2 PAX8 GDF5 NOG B9D1 DACT1 SHROOM3 BMP2 DSP RIPK4 ADGRG1 ADAMTS9 CDH3 IRF6 TDRD7 TUBB2B GPC4
1_Member	GO Biological Processes	GO:0048598	embryonic morphogenesis	2.28E-08	1.76E-06	21/576	ALDH1A3 CDKN1C EDN1 FOXC1 GATA6 ITGB4 PAX2 SALL1 SMO SOX4 SOX9 SOX11 ST14 HNF1B TGFB2 PAX8 GDF5 NOG B9D1 DACT1 SHROOM3
1_Member	GO Biological Processes	GO:0048729	tissue morphogenesis	2.04E-07	1.16E-05	19/554	ALDH1A3 BMP2 DSP EDN1 FOXC1 ITGB4 PAX2 SALL1 SMO SOX4 SOX9 SOX11 ST14 HNF1B TGFB2 PAX8 NOG RIPK4 SHROOM3
1_Member	GO Biological Processes	GO:0035239	tube morphogenesis	8.16E-07	3.91E-05	19/661	BMP2 EDN1 FOXC1 GATA6 PAX2 SALL1 SMO SOX4 SOX9 SOX11 ST14 HNF1B TGFB2 PAX8 NOG ADGRG1 DACT1 ADAMTS9 SHROOM3
1_Member	GO Biological Processes	GO:0002009	morphogenesis of an epithelium	1.66E-06	6.36E-05	16/443	ALDH1A3 BMP2 EDN1 PAX2 SALL1 SMO SOX4 SOX9 SOX11 ST14 HNF1B TGFB2 PAX8 NOG RIPK4 SHROOM3
1_Member	GO Biological Processes	GO:0030855	epithelial cell differentiation	2.14E-06	6.82E-05	17/549	BMP2 CDH3 DSP GATA6 IRF6 PAX2 SALL1 SMO SOX4 SOX9 SOX11 ST14 HNF1B PAX8 TDRD7 B9D1 SHROOM3
1_Member	GO Biological Processes	GO:0060562	epithelial tube morphogenesis	2.18E-06	6.82E-05	14/307	BMP2 EDN1 PAX2 SALL1 SMO SOX4 SOX9 SOX11 ST14 HNF1B TGFB2 PAX8 NOG SHROOM3
1_Member	GO Biological Processes	GO:0016331	morphogenesis of embryonic epithelium	2.81E-06	8.35E-05	11/143	ALDH1A3 PAX2 SOX4 SOX9 SOX11 ST14 HNF1B TGFB2 PAX8 NOG SHROOM3
1_Member	GO Biological Processes	GO:0043009	chordate embryonic development	5.52E-06	1.55E-04	17/629	BMP2 CDKN1C EDN1 FOXC1 GATA6 PAX2 SMO SOX4 SOX9 SOX11 ST14 HNF1B TGFB2 NOG B9D1 DACT1 SHROOM3
1_Member	GO Biological Processes	GO:0001822	kidney development	5.76E-06	1.55E-04	13/288	BMP2 CDKN1C FOXC1 PAX2 SALL1 SMO SOX4 SOX9 SOX11 HNF1B TGFB2 PAX8 NOG
1_Member	GO Biological Processes	GO:0048568	embryonic organ development	5.91E-06	1.55E-04	15/446	ALDH1A3 CDKN1C EDN1 FOXC1 PAX2 SALL1 SMO SOX9 SOX11 ST14 HNF1B TGFB2 PAX8 NOG TUBB2B
1_Member	GO Biological Processes	GO:0001838	embryonic epithelial tube formation	6.57E-06	1.59E-04	10/122	PAX2 SOX4 SOX9 SOX11 ST14 HNF1B TGFB2 PAX8 NOG SHROOM3
1_Member	GO Biological Processes	GO:0060993	kidney morphogenesis	6.81E-06	1.59E-04	9/83	BMP2 PAX2 SALL1 SMO SOX4 SOX9 HNF1B PAX8 NOG
1_Member	GO Biological Processes	GO:0072001	renal system development	6.82E-06	1.59E-04	13/297	BMP2 CDKN1C FOXC1 PAX2 SALL1 SMO SOX4 SOX9 SOX11 HNF1B TGFB2 PAX8 NOG
1_Member	GO Biological Processes	GO:0009792	embryo development ending in birth or egg hatching	6.93E-06	1.59E-04	17/650	BMP2 CDKN1C EDN1 FOXC1 GATA6 PAX2 SMO SOX4 SOX9 SOX11 ST14 HNF1B TGFB2 NOG B9D1 DACT1 SHROOM3
1_Member	GO Biological Processes	GO:0035270	endocrine system development	7.06E-06	1.59E-04	10/124	BMP2 CDKN1C EDN1 GATA6 SALL1 SMO SOX4 SOX9 PAX8 NOG
1_Member	GO Biological Processes	GO:0072175	epithelial tube formation	8.69E-06	1.79E-04	10/130	PAX2 SOX4 SOX9 SOX11 ST14 HNF1B TGFB2 PAX8 NOG SHROOM3
1_Member	GO Biological Processes	GO:0048732	gland development	1.05E-05	2.07E-04	14/401	ALDH1A3 BMP2 CDKN1C EDN1 FOXC1 GATA6 IRF6 SALL1 SMO SOX9 HNF1B TGFB2 PAX8 NOG
1_Member	GO Biological Processes	GO:0001655	urogenital system development	1.30E-05	2.52E-04	13/334	BMP2 CDKN1C FOXC1 PAX2 SALL1 SMO SOX4 SOX9 SOX11 HNF1B TGFB2 PAX8 NOG
1_Member	GO Biological Processes	GO:0072009	nephron epithelium development	1.38E-05	2.63E-04	9/99	BMP2 FOXC1 PAX2 SALL1 SMO SOX9 HNF1B PAX8 NOG
1_Member	GO Biological Processes	GO:0035148	tube formation	1.44E-05	2.70E-04	10/146	PAX2 SOX4 SOX9 SOX11 ST14 HNF1B TGFB2 PAX8 NOG SHROOM3
1_Member	GO Biological Processes	GO:0072088	nephron epithelium morphogenesis	1.54E-05	2.85E-04	8/65	BMP2 PAX2 SALL1 SMO SOX9 HNF1B PAX8 NOG
1_Member	GO Biological Processes	GO:0072028	nephron morphogenesis	1.72E-05	3.13E-04	8/67	BMP2 PAX2 SALL1 SMO SOX9 HNF1B PAX8 NOG
1_Member	GO Biological Processes	GO:2001054	negative regulation of mesenchymal cell apoptotic process	2.71E-05	4.77E-04	5/10	PAX2 SOX9 HNF1B PAX8 GDF5
1_Member	GO Biological Processes	GO:0001657	ureteric bud development	3.55E-05	6.13E-04	8/82	BMP2 FOXC1 PAX2 SALL1 SOX9 HNF1B PAX8 NOG
1_Member	GO Biological Processes	GO:2000027	regulation of animal organ morphogenesis	3.58E-05	6.13E-04	9/126	BMP2 EDN1 GPC4 PAX2 SMO SOX9 PAX8 NOG DACT1
1_Member	GO Biological Processes	GO:0072163	mesonephric epithelium development	3.71E-05	6.19E-04	8/83	BMP2 FOXC1 PAX2 SALL1 SOX9 HNF1B PAX8 NOG
1_Member	GO Biological Processes	GO:0072164	mesonephric tubule development	3.71E-05	6.19E-04	8/83	BMP2 FOXC1 PAX2 SALL1 SOX9 HNF1B PAX8 NOG
1_Member	GO Biological Processes	GO:0072006	nephron development	3.81E-05	6.20E-04	9/128	BMP2 FOXC1 PAX2 SALL1 SMO SOX9 HNF1B PAX8 NOG
1_Member	GO Biological Processes	GO:0072073	kidney epithelium development	3.81E-05	6.20E-04	9/128	BMP2 FOXC1 PAX2 SALL1 SMO SOX9 HNF1B PAX8 NOG
1_Member	GO Biological Processes	GO:0001823	mesonephros development	4.38E-05	6.98E-04	8/87	BMP2 FOXC1 PAX2 SALL1 SOX9 HNF1B PAX8 NOG
1_Member	GO Biological Processes	GO:0060675	ureteric bud morphogenesis	4.39E-05	6.98E-04	7/52	BMP2 PAX2 SALL1 SOX9 HNF1B PAX8 NOG
1_Member	GO Biological Processes	GO:1902337	regulation of apoptotic process involved in morphogenesis	4.45E-05	6.99E-04	5/12	FOXC1 PAX2 HNF1B TGFB2 PAX8
1_Member	GO Biological Processes	GO:0072171	mesonephric tubule morphogenesis	4.67E-05	7.26E-04	7/53	BMP2 PAX2 SALL1 SOX9 HNF1B PAX8 NOG
1_Member	GO Biological Processes	GO:1904748	regulation of apoptotic process involved in development	5.49E-05	8.35E-04	5/13	FOXC1 PAX2 HNF1B TGFB2 PAX8
1_Member	GO Biological Processes	GO:2001053	regulation of mesenchymal cell apoptotic process	5.49E-05	8.35E-04	5/13	PAX2 SOX9 HNF1B PAX8 GDF5
1_Member	GO Biological Processes	GO:0090183	regulation of kidney development	6.92E-05	1.03E-03	6/32	PAX2 SMO SOX9 HNF1B PAX8 NOG
1_Member	GO Biological Processes	GO:0021915	neural tube development	7.25E-05	1.07E-03	9/151	PAX2 SMO SOX4 SOX11 ST14 TGFB2 NOG DACT1 SHROOM3
1_Member	GO Biological Processes	GO:0072078	nephron tubule morphogenesis	8.06E-05	1.17E-03	7/63	BMP2 PAX2 SALL1 SOX9 HNF1B PAX8 NOG
1_Member	GO Biological Processes	GO:0061138	morphogenesis of a branching epithelium	9.52E-05	1.35E-03	9/162	BMP2 EDN1 PAX2 SALL1 SOX9 ST14 HNF1B PAX8 NOG
1_Member	GO Biological Processes	GO:0061333	renal tubule morphogenesis	9.77E-05	1.38E-03	7/67	BMP2 PAX2 SALL1 SOX9 HNF1B PAX8 NOG
1_Member	GO Biological Processes	GO:1902338	negative regulation of apoptotic process involved in morphogenesis	1.07E-04	1.47E-03	4/6	FOXC1 PAX2 HNF1B PAX8
1_Member	GO Biological Processes	GO:1904746	negative regulation of apoptotic process involved in development	1.07E-04	1.47E-03	4/6	FOXC1 PAX2 HNF1B PAX8
1_Member	GO Biological Processes	GO:0001763	morphogenesis of a branching structure	1.17E-04	1.57E-03	9/171	BMP2 EDN1 PAX2 SALL1 SOX9 ST14 HNF1B PAX8 NOG
1_Member	GO Biological Processes	GO:0072079	nephron tubule formation	1.24E-04	1.65E-03	5/18	PAX2 SOX9 HNF1B PAX8 NOG
1_Member	GO Biological Processes	GO:0090190	positive regulation of branching involved in ureteric bud morphogenesis	1.24E-04	1.65E-03	5/18	PAX2 SMO SOX9 PAX8 NOG
1_Member	GO Biological Processes	GO:0072080	nephron tubule development	1.45E-04	1.90E-03	7/76	BMP2 PAX2 SALL1 SOX9 HNF1B PAX8 NOG
1_Member	GO Biological Processes	GO:0061326	renal tubule development	1.69E-04	2.19E-03	7/80	BMP2 PAX2 SALL1 SOX9 HNF1B PAX8 NOG
1_Member	GO Biological Processes	GO:0072273	metanephric nephron morphogenesis	1.81E-04	2.29E-03	5/21	PAX2 SALL1 SMO SOX9 PAX8
1_Member	GO Biological Processes	GO:0090189	regulation of branching involved in ureteric bud morphogenesis	1.81E-04	2.29E-03	5/21	PAX2 SMO SOX9 PAX8 NOG
1_Member	GO Biological Processes	GO:0003337	mesenchymal to epithelial transition involved in metanephros morphogenesis	2.08E-04	2.62E-03	4/8	PAX2 SALL1 SMO PAX8
1_Member	GO Biological Processes	GO:0003338	metanephros morphogenesis	3.56E-04	4.23E-03	5/28	PAX2 SALL1 SMO SOX9 PAX8
1_Member	GO Biological Processes	GO:0001841	neural tube formation	3.67E-04	4.32E-03	7/103	PAX2 SOX4 SOX11 ST14 TGFB2 NOG SHROOM3
1_Member	GO Biological Processes	GO:0072283	metanephric renal vesicle morphogenesis	4.07E-04	4.68E-03	4/11	PAX2 SALL1 SMO PAX8
1_Member	GO Biological Processes	GO:1900211	regulation of mesenchymal cell apoptotic process involved in metanephros development	4.39E-04	4.97E-03	3/3	PAX2 HNF1B PAX8
1_Member	GO Biological Processes	GO:1900212	negative regulation of mesenchymal cell apoptotic process involved in metanephros development	4.39E-04	4.97E-03	3/3	PAX2 HNF1B PAX8
1_Member	GO Biological Processes	GO:0072210	metanephric nephron development	4.84E-04	5.39E-03	5/32	PAX2 SALL1 SMO SOX9 PAX8
1_Member	GO Biological Processes	GO:0072077	renal vesicle morphogenesis	5.68E-04	6.27E-03	4/13	PAX2 SALL1 SMO PAX8
1_Member	GO Biological Processes	GO:1905332	positive regulation of morphogenesis of an epithelium	6.33E-04	6.89E-03	5/36	PAX2 SMO SOX9 PAX8 NOG
1_Member	GO Biological Processes	GO:0072087	renal vesicle development	6.57E-04	7.08E-03	4/14	PAX2 SALL1 SMO PAX8
1_Member	GO Biological Processes	GO:0048754	branching morphogenesis of an epithelial tube	7.93E-04	8.32E-03	7/133	BMP2 EDN1 PAX2 SALL1 SOX9 HNF1B PAX8
1_Member	GO Biological Processes	GO:0072039	regulation of mesenchymal cell apoptotic process involved in nephron morphogenesis	8.01E-04	8.32E-03	3/4	PAX2 HNF1B PAX8
1_Member	GO Biological Processes	GO:0072040	negative regulation of mesenchymal cell apoptotic process involved in nephron morphogenesis	8.01E-04	8.32E-03	3/4	PAX2 HNF1B PAX8
1_Member	GO Biological Processes	GO:0072289	metanephric nephron tubule formation	8.01E-04	8.32E-03	3/4	PAX2 SOX9 PAX8
1_Member	GO Biological Processes	GO:0001701	in utero embryonic development	8.13E-04	8.41E-03	10/381	BMP2 CDKN1C EDN1 FOXC1 GATA6 SMO ST14 HNF1B NOG B9D1
1_Member	GO Biological Processes	GO:0060231	mesenchymal to epithelial transition	8.50E-04	8.76E-03	4/16	PAX2 SALL1 SMO PAX8
1_Member	GO Biological Processes	GO:0048562	embryonic organ morphogenesis	8.81E-04	9.01E-03	9/292	ALDH1A3 EDN1 PAX2 SMO SOX9 SOX11 HNF1B PAX8 NOG
1_Member	GO Biological Processes	GO:0001658	branching involved in ureteric bud morphogenesis	8.97E-04	9.13E-03	5/42	BMP2 PAX2 SALL1 SOX9 PAX8
1_Member	GO Biological Processes	GO:0043583	ear development	1.01E-03	1.03E-02	8/217	ALDH1A3 BMP2 EDN1 PAX2 SOX9 TGFB2 PAX8 NOG
1_Member	GO Biological Processes	GO:0072202	cell differentiation involved in metanephros development	1.06E-03	1.07E-02	4/18	PAX2 SALL1 SMO PAX8
1_Member	GO Biological Processes	GO:1904673	negative regulation of somatic stem cell population maintenance	1.19E-03	1.19E-02	3/5	PAX2 HNF1B PAX8
1_Member	GO Biological Processes	GO:0060688	regulation of morphogenesis of a branching structure	1.38E-03	1.34E-02	5/51	PAX2 SMO SOX9 PAX8 NOG
1_Member	GO Biological Processes	GO:0072174	metanephric tubule formation	1.61E-03	1.54E-02	3/6	PAX2 SOX9 PAX8
1_Member	GO Biological Processes	GO:0090596	sensory organ morphogenesis	1.87E-03	1.76E-02	8/261	ALDH1A3 EDN1 PAX2 SOX9 SOX11 PAX8 NOG TDRD7
1_Member	GO Biological Processes	GO:1905330	regulation of morphogenesis of an epithelium	2.36E-03	2.14E-02	5/65	PAX2 SMO SOX9 PAX8 NOG
1_Member	GO Biological Processes	GO:0042471	ear morphogenesis	2.45E-03	2.21E-02	6/122	ALDH1A3 EDN1 PAX2 SOX9 PAX8 NOG
1_Member	GO Biological Processes	GO:0048793	pronephros development	2.51E-03	2.25E-02	3/8	PAX2 HNF1B PAX8
1_Member	GO Biological Processes	GO:0072282	metanephric nephron tubule morphogenesis	2.51E-03	2.25E-02	3/8	PAX2 SOX9 PAX8
1_Member	GO Biological Processes	GO:1902455	negative regulation of stem cell population maintenance	2.51E-03	2.25E-02	3/8	PAX2 HNF1B PAX8
1_Member	GO Biological Processes	GO:0072173	metanephric tubule morphogenesis	3.49E-03	2.99E-02	3/10	PAX2 SOX9 PAX8
1_Member	GO Biological Processes	GO:0090184	positive regulation of kidney development	3.49E-03	2.99E-02	3/10	PAX2 SOX9 NOG
1_Member	GO Biological Processes	GO:1904672	regulation of somatic stem cell population maintenance	3.49E-03	2.99E-02	3/10	PAX2 HNF1B PAX8
1_Member	GO Biological Processes	GO:2000036	regulation of stem cell population maintenance	3.60E-03	3.06E-02	4/35	PAX2 SMO HNF1B PAX8
1_Member	GO Biological Processes	GO:0035850	epithelial cell differentiation involved in kidney development	3.78E-03	3.18E-02	4/36	PAX2 SALL1 SMO PAX8
1_Member	GO Biological Processes	GO:0001656	metanephros development	3.80E-03	3.18E-02	5/81	PAX2 SALL1 SMO SOX9 PAX8
1_Member	GO Biological Processes	GO:0030856	regulation of epithelial cell differentiation	4.63E-03	3.79E-02	6/157	CDKN1C FOXC1 PAX2 SMO SOX9 PAX8
1_Member	GO Biological Processes	GO:0001843	neural tube closure	4.65E-03	3.80E-02	5/89	PAX2 ST14 TGFB2 NOG SHROOM3
1_Member	GO Biological Processes	GO:0060606	tube closure	4.76E-03	3.87E-02	5/90	PAX2 ST14 TGFB2 NOG SHROOM3
1_Member	GO Biological Processes	GO:0014020	primary neural tube formation	5.34E-03	4.29E-02	5/95	PAX2 ST14 TGFB2 NOG SHROOM3
1_Member	GO Biological Processes	GO:0072234	metanephric nephron tubule development	5.63E-03	4.50E-02	3/14	PAX2 SOX9 PAX8
1_Member	GO Biological Processes	GO:0061005	cell differentiation involved in kidney development	6.06E-03	4.78E-02	4/47	PAX2 SALL1 SMO PAX8
1_Member	GO Biological Processes	GO:0072170	metanephric tubule development	6.78E-03	5.26E-02	3/16	PAX2 SOX9 PAX8
1_Member	GO Biological Processes	GO:0072243	metanephric nephron epithelium development	6.78E-03	5.26E-02	3/16	PAX2 SOX9 PAX8
1_Member	GO Biological Processes	GO:0048839	inner ear development	7.43E-03	5.71E-02	6/190	ALDH1A3 BMP2 PAX2 SOX9 TGFB2 PAX8
1_Member	GO Biological Processes	GO:0072207	metanephric epithelium development	8.58E-03	6.47E-02	3/19	PAX2 SOX9 PAX8
1_Member	GO Biological Processes	GO:0030858	positive regulation of epithelial cell differentiation	1.06E-02	7.83E-02	4/65	FOXC1 PAX2 SOX9 PAX8
2_Summary	GO Biological Processes	GO:0003205	cardiac chamber development	1.30E-06	5.47E-05	12/167	BMP2 DSP FOXC1 GATA6 SALL1 SMO SOX4 SOX11 TGFB2 PAX8 NOG ADGRG6 EDN1 SOX9 KDM6A ADAMTS9 SIK1 ITGA6 IRF6 ITGB4 GDF5 B9D1 CDH3 CDKN1C PAX2 CASK TNNT1 MATN3 PPM1D ADGRG1 CHRDL1 NEDD4L IL11 H4C11 CCND2 RUBCN
2_Member	GO Biological Processes	GO:0003205	cardiac chamber development	1.30E-06	5.47E-05	12/167	BMP2 DSP FOXC1 GATA6 SALL1 SMO SOX4 SOX11 TGFB2 PAX8 NOG ADGRG6
2_Member	GO Biological Processes	GO:0007507	heart development	1.96E-06	6.82E-05	17/542	BMP2 DSP EDN1 FOXC1 GATA6 SALL1 SMO SOX4 SOX9 SOX11 TGFB2 KDM6A PAX8 NOG ADAMTS9 ADGRG6 SIK1
2_Member	GO Biological Processes	GO:0003206	cardiac chamber morphogenesis	7.58E-06	1.64E-04	10/126	BMP2 DSP FOXC1 GATA6 SMO SOX4 SOX11 TGFB2 NOG ADGRG6
2_Member	GO Biological Processes	GO:0048736	appendage development	7.80E-06	1.64E-04	11/177	ITGA6 IRF6 ITGB4 SALL1 SOX4 SOX9 SOX11 TGFB2 GDF5 NOG B9D1
2_Member	GO Biological Processes	GO:0060173	limb development	7.80E-06	1.64E-04	11/177	ITGA6 IRF6 ITGB4 SALL1 SOX4 SOX9 SOX11 TGFB2 GDF5 NOG B9D1
2_Member	GO Biological Processes	GO:0003007	heart morphogenesis	1.04E-05	2.07E-04	12/250	BMP2 DSP EDN1 FOXC1 GATA6 SMO SOX4 SOX9 SOX11 TGFB2 NOG ADGRG6
2_Member	GO Biological Processes	GO:0003279	cardiac septum development	8.78E-05	1.26E-03	8/106	GATA6 SALL1 SMO SOX4 SOX11 TGFB2 PAX8 NOG
2_Member	GO Biological Processes	GO:0060537	muscle tissue development	1.00E-04	1.40E-03	11/306	BMP2 DSP FOXC1 GATA6 SMO SOX9 SOX11 TGFB2 NOG ADAMTS9 SIK1
2_Member	GO Biological Processes	GO:0003231	cardiac ventricle development	1.60E-04	2.09E-03	8/126	DSP FOXC1 SALL1 SOX4 SOX11 TGFB2 PAX8 NOG
2_Member	GO Biological Processes	GO:0035107	appendage morphogenesis	2.42E-04	2.95E-03	8/142	SALL1 SOX4 SOX9 SOX11 TGFB2 GDF5 NOG B9D1
2_Member	GO Biological Processes	GO:0035108	limb morphogenesis	2.42E-04	2.95E-03	8/142	SALL1 SOX4 SOX9 SOX11 TGFB2 GDF5 NOG B9D1
2_Member	GO Biological Processes	GO:0014706	striated muscle tissue development	2.55E-04	3.07E-03	10/287	BMP2 DSP FOXC1 GATA6 SMO SOX11 TGFB2 NOG ADAMTS9 SIK1
2_Member	GO Biological Processes	GO:0050678	regulation of epithelial cell proliferation	2.65E-04	3.17E-03	11/379	BMP2 CDH3 CDKN1C PAX2 SMO SOX9 SOX11 TGFB2 GDF5 CASK NOG
2_Member	GO Biological Processes	GO:0048738	cardiac muscle tissue development	4.74E-04	5.31E-03	8/173	BMP2 DSP FOXC1 GATA6 TGFB2 NOG ADAMTS9 SIK1
2_Member	GO Biological Processes	GO:0048844	artery morphogenesis	5.58E-04	6.19E-03	6/69	EDN1 FOXC1 SOX4 TGFB2 NOG ADAMTS9
2_Member	GO Biological Processes	GO:0060411	cardiac septum morphogenesis	6.01E-04	6.60E-03	6/71	GATA6 SMO SOX4 SOX11 TGFB2 NOG
2_Member	GO Biological Processes	GO:0003208	cardiac ventricle morphogenesis	6.24E-04	6.82E-03	6/72	DSP FOXC1 SOX4 SOX11 TGFB2 NOG
2_Member	GO Biological Processes	GO:0003281	ventricular septum development	6.47E-04	7.00E-03	6/73	SALL1 SOX4 SOX11 TGFB2 PAX8 NOG
2_Member	GO Biological Processes	GO:0061061	muscle structure development	7.55E-04	8.02E-03	11/480	BMP2 DSP FOXC1 GATA6 SMO SOX9 SOX11 TGFB2 TNNT1 NOG SIK1
2_Member	GO Biological Processes	GO:0001501	skeletal system development	8.64E-04	8.87E-03	11/495	BMP2 CDKN1C EDN1 FOXC1 MATN3 SOX4 SOX9 SOX11 TGFB2 GDF5 NOG
2_Member	GO Biological Processes	GO:0008285	negative regulation of cell population proliferation	1.12E-03	1.13E-02	13/784	BMP2 CDKN1C GATA6 IRF6 SOX4 SOX9 SOX11 TGFB2 GDF5 PPM1D CASK NOG ADGRG1
2_Member	GO Biological Processes	GO:0071772	response to BMP	1.25E-03	1.24E-02	6/94	BMP2 GATA6 SOX9 TGFB2 GDF5 NOG
2_Member	GO Biological Processes	GO:0071773	cellular response to BMP stimulus	1.25E-03	1.24E-02	6/94	BMP2 GATA6 SOX9 TGFB2 GDF5 NOG
2_Member	GO Biological Processes	GO:0060840	artery development	1.32E-03	1.29E-02	6/96	EDN1 FOXC1 SOX4 TGFB2 NOG ADAMTS9
2_Member	GO Biological Processes	GO:0072132	mesenchyme morphogenesis	1.38E-03	1.34E-02	5/51	BMP2 FOXC1 SOX9 TGFB2 NOG
2_Member	GO Biological Processes	GO:0003229	ventricular cardiac muscle tissue development	1.70E-03	1.61E-02	5/56	DSP FOXC1 TGFB2 NOG ADAMTS9
2_Member	GO Biological Processes	GO:0055008	cardiac muscle tissue morphogenesis	1.98E-03	1.86E-02	5/60	BMP2 DSP FOXC1 TGFB2 NOG
2_Member	GO Biological Processes	GO:0090092	regulation of transmembrane receptor protein serine/threonine kinase signaling pathway	2.01E-03	1.88E-02	8/267	BMP2 CDKN1C GATA6 SOX11 TGFB2 GDF5 NOG CHRDL1
2_Member	GO Biological Processes	GO:0090100	positive regulation of transmembrane receptor protein serine/threonine kinase signaling pathway	2.35E-03	2.14E-02	6/120	BMP2 CDKN1C GATA6 SOX11 TGFB2 GDF5
2_Member	GO Biological Processes	GO:0001944	vasculature development	2.92E-03	2.61E-02	10/526	EDN1 FOXC1 GATA6 SMO SOX4 TGFB2 NOG ADGRG1 B9D1 ADAMTS9
2_Member	GO Biological Processes	GO:0050679	positive regulation of epithelial cell proliferation	2.99E-03	2.65E-02	7/209	BMP2 CDH3 PAX2 SMO SOX9 SOX11 NOG
2_Member	GO Biological Processes	GO:0060415	muscle tissue morphogenesis	3.03E-03	2.68E-02	5/73	BMP2 DSP FOXC1 TGFB2 NOG
2_Member	GO Biological Processes	GO:0003151	outflow tract morphogenesis	3.31E-03	2.88E-02	5/76	EDN1 GATA6 SOX11 TGFB2 NOG
2_Member	GO Biological Processes	GO:0090287	regulation of cellular response to growth factor stimulus	3.40E-03	2.95E-02	8/314	BMP2 CDKN1C GATA6 SOX11 GDF5 CASK NOG CHRDL1
2_Member	GO Biological Processes	GO:0061626	pharyngeal arch artery morphogenesis	3.49E-03	2.99E-02	3/10	EDN1 TGFB2 NOG
2_Member	GO Biological Processes	GO:0001837	epithelial to mesenchymal transition	3.50E-03	2.99E-02	5/78	BMP2 EDN1 SOX9 TGFB2 NOG
2_Member	GO Biological Processes	GO:0048644	muscle organ morphogenesis	3.70E-03	3.14E-02	5/80	BMP2 DSP FOXC1 TGFB2 NOG
2_Member	GO Biological Processes	GO:0003203	endocardial cushion morphogenesis	3.78E-03	3.18E-02	4/36	BMP2 SOX9 TGFB2 NOG
2_Member	GO Biological Processes	GO:0051216	cartilage development	4.63E-03	3.79E-02	6/157	BMP2 EDN1 MATN3 SOX9 GDF5 NOG
2_Member	GO Biological Processes	GO:0071363	cellular response to growth factor stimulus	4.68E-03	3.81E-02	9/464	BMP2 EDN1 FOXC1 GATA6 PAX2 SOX9 TGFB2 GDF5 NOG
2_Member	GO Biological Processes	GO:0060412	ventricular septum morphogenesis	4.97E-03	4.02E-02	4/42	SOX4 SOX11 TGFB2 NOG
2_Member	GO Biological Processes	GO:0070848	response to growth factor	5.78E-03	4.61E-02	9/493	BMP2 EDN1 FOXC1 GATA6 PAX2 SOX9 TGFB2 GDF5 NOG
2_Member	GO Biological Processes	GO:0003197	endocardial cushion development	5.84E-03	4.63E-02	4/46	BMP2 SOX9 TGFB2 NOG
2_Member	GO Biological Processes	GO:0055010	ventricular cardiac muscle tissue morphogenesis	6.06E-03	4.78E-02	4/47	DSP FOXC1 TGFB2 NOG
2_Member	GO Biological Processes	GO:0001568	blood vessel development	6.20E-03	4.85E-02	9/503	EDN1 FOXC1 GATA6 SMO SOX4 TGFB2 NOG ADGRG1 ADAMTS9
2_Member	GO Biological Processes	GO:0001503	ossification	6.29E-03	4.91E-02	7/271	BMP2 FOXC1 SMO SOX9 GDF5 NOG CHRDL1
2_Member	GO Biological Processes	GO:0030510	regulation of BMP signaling pathway	6.61E-03	5.15E-02	5/105	GATA6 SOX11 GDF5 NOG CHRDL1
2_Member	GO Biological Processes	GO:0007517	muscle organ development	7.25E-03	5.59E-02	7/285	BMP2 DSP FOXC1 SMO SOX11 TGFB2 NOG
2_Member	GO Biological Processes	GO:0048514	blood vessel morphogenesis	8.49E-03	6.43E-02	8/419	EDN1 FOXC1 SMO SOX4 TGFB2 NOG ADGRG1 ADAMTS9
2_Member	GO Biological Processes	GO:0030326	embryonic limb morphogenesis	8.61E-03	6.47E-02	5/119	SALL1 TGFB2 GDF5 NOG B9D1
2_Member	GO Biological Processes	GO:0035113	embryonic appendage morphogenesis	8.61E-03	6.47E-02	5/119	SALL1 TGFB2 GDF5 NOG B9D1
2_Member	GO Biological Processes	GO:0061448	connective tissue development	9.29E-03	6.91E-02	6/208	BMP2 EDN1 MATN3 SOX9 GDF5 NOG
2_Member	GO Biological Processes	GO:0060393	regulation of pathway-restricted SMAD protein phosphorylation	9.81E-03	7.26E-02	4/62	BMP2 TGFB2 GDF5 NOG
2_Member	GO Biological Processes	GO:0061035	regulation of cartilage development	1.21E-02	8.71E-02	4/70	BMP2 SOX9 GDF5 NOG
2_Member	GO Biological Processes	GO:0021536	diencephalon development	1.36E-02	9.68E-02	4/75	BMP2 SALL1 SMO NOG
3_Summary	GO Biological Processes	GO:0007423	sensory organ development	2.17E-05	3.88E-04	15/551	ALDH1A3 BMP2 CDKN1C EDN1 FOXC1 MITF PAX2 SOX9 SOX11 TGFB2 PAX8 NOG TDRD7 B9D1 CHRDL1 SALL1
3_Member	GO Biological Processes	GO:0007423	sensory organ development	2.17E-05	3.88E-04	15/551	ALDH1A3 BMP2 CDKN1C EDN1 FOXC1 MITF PAX2 SOX9 SOX11 TGFB2 PAX8 NOG TDRD7 B9D1 CHRDL1
3_Member	GO Biological Processes	GO:0001654	eye development	2.21E-04	2.76E-03	11/364	ALDH1A3 CDKN1C FOXC1 MITF PAX2 SOX9 SOX11 TGFB2 TDRD7 B9D1 CHRDL1
3_Member	GO Biological Processes	GO:0150063	visual system development	2.32E-04	2.86E-03	11/368	ALDH1A3 CDKN1C FOXC1 MITF PAX2 SOX9 SOX11 TGFB2 TDRD7 B9D1 CHRDL1
3_Member	GO Biological Processes	GO:0048880	sensory system development	2.49E-04	3.02E-03	11/374	ALDH1A3 CDKN1C FOXC1 MITF PAX2 SOX9 SOX11 TGFB2 TDRD7 B9D1 CHRDL1
3_Member	GO Biological Processes	GO:0043010	camera-type eye development	3.89E-04	4.52E-03	10/318	ALDH1A3 CDKN1C FOXC1 MITF PAX2 SOX9 SOX11 TGFB2 TDRD7 B9D1
3_Member	WikiPathways	WP4830	GDNF/RET signaling axis	1.68E-03	1.60E-02	4/23	FOXC1 PAX2 SALL1 SOX11
3_Member	GO Biological Processes	GO:0002072	optic cup morphogenesis involved in camera-type eye development	2.05E-03	1.91E-02	3/7	ALDH1A3 PAX2 SOX11
3_Member	GO Biological Processes	GO:0060900	embryonic camera-type eye formation	4.00E-03	3.33E-02	3/11	ALDH1A3 PAX2 SOX11
3_Member	GO Biological Processes	GO:0048593	camera-type eye morphogenesis	9.07E-03	6.79E-02	5/122	ALDH1A3 PAX2 SOX9 SOX11 TDRD7
3_Member	GO Biological Processes	GO:0048596	embryonic camera-type eye morphogenesis	1.31E-02	9.36E-02	3/26	ALDH1A3 PAX2 SOX11
3_Member	GO Biological Processes	GO:0048592	eye morphogenesis	1.39E-02	9.83E-02	5/150	ALDH1A3 PAX2 SOX9 SOX11 TDRD7
4_Summary	WikiPathways	WP5053	Development of ureteric collection system	6.91E-05	1.03E-03	7/60	BMP2 FOXC1 PAX2 SALL1 SMO HNF1B TGFB2 EDN1 MID1 PAX8 NOG ADGRG1 TDRD7 SHROOM3 ALDH1A3 SOX9 SPTBN2 TRAPPC9 POMK
4_Member	WikiPathways	WP5053	Development of ureteric collection system	6.91E-05	1.03E-03	7/60	BMP2 FOXC1 PAX2 SALL1 SMO HNF1B TGFB2
4_Member	GO Biological Processes	GO:0007389	pattern specification process	1.81E-04	2.29E-03	12/443	BMP2 EDN1 FOXC1 MID1 PAX2 SMO HNF1B PAX8 NOG ADGRG1 TDRD7 SHROOM3
4_Member	GO Biological Processes	GO:0003002	regionalization	4.64E-04	5.23E-03	10/332	BMP2 EDN1 FOXC1 PAX2 SMO HNF1B PAX8 NOG ADGRG1 TDRD7
4_Member	GO Biological Processes	GO:0060322	head development	1.15E-03	1.15E-02	13/787	ALDH1A3 BMP2 FOXC1 PAX2 SALL1 SMO SOX9 SPTBN2 HNF1B NOG ADGRG1 TRAPPC9 POMK
4_Member	GO Biological Processes	GO:0007420	brain development	2.07E-03	1.91E-02	12/742	ALDH1A3 BMP2 FOXC1 PAX2 SALL1 SMO SPTBN2 HNF1B NOG ADGRG1 TRAPPC9 POMK
4_Member	GO Biological Processes	GO:0009952	anterior/posterior pattern specification	9.51E-03	7.06E-02	6/210	BMP2 FOXC1 SMO HNF1B NOG TDRD7
4_Member	GO Biological Processes	GO:0021537	telencephalon development	1.41E-02	9.91E-02	6/247	ALDH1A3 BMP2 SALL1 SMO ADGRG1 TRAPPC9
5_Summary	GO Biological Processes	GO:0042063	gliogenesis	7.44E-05	1.09E-03	10/214	MYRF ITGB4 PAX2 SMO SOX4 SOX9 SOX11 TGFB2 ADGRG1 ADGRG6
5_Member	GO Biological Processes	GO:0042063	gliogenesis	7.44E-05	1.09E-03	10/214	MYRF ITGB4 PAX2 SMO SOX4 SOX9 SOX11 TGFB2 ADGRG1 ADGRG6
5_Member	GO Biological Processes	GO:0010001	glial cell differentiation	4.20E-04	4.81E-03	8/167	MYRF ITGB4 PAX2 SMO SOX4 SOX9 SOX11 ADGRG6
5_Member	GO Biological Processes	GO:0021782	glial cell development	1.79E-03	1.69E-02	6/108	MYRF ITGB4 SMO SOX4 SOX11 ADGRG6
6_Summary	GO Biological Processes	GO:0048762	mesenchymal cell differentiation	1.09E-04	1.49E-03	9/168	BMP2 EDN1 FOXC1 PAX2 SMO SOX9 SOX11 TGFB2 NOG HNF1B SOX4 VLDLR SPTBN2 DACT1 CDKN1C
6_Member	GO Biological Processes	GO:0048762	mesenchymal cell differentiation	1.09E-04	1.49E-03	9/168	BMP2 EDN1 FOXC1 PAX2 SMO SOX9 SOX11 TGFB2 NOG
6_Member	GO Biological Processes	GO:0060485	mesenchyme development	3.62E-04	4.28E-03	9/230	BMP2 EDN1 FOXC1 PAX2 SMO SOX9 SOX11 TGFB2 NOG
6_Member	GO Biological Processes	GO:0048863	stem cell differentiation	3.95E-04	4.57E-03	8/164	EDN1 FOXC1 PAX2 SMO SOX9 SOX11 HNF1B TGFB2
6_Member	GO Biological Processes	GO:0014032	neural crest cell development	3.80E-03	3.18E-02	5/81	EDN1 FOXC1 SMO SOX9 SOX11
6_Member	GO Biological Processes	GO:0014031	mesenchymal cell development	4.32E-03	3.55E-02	5/86	EDN1 FOXC1 SMO SOX9 SOX11
6_Member	GO Biological Processes	GO:0048864	stem cell development	4.32E-03	3.55E-02	5/86	EDN1 FOXC1 SMO SOX9 SOX11
6_Member	GO Biological Processes	GO:0014033	neural crest cell differentiation	5.11E-03	4.11E-02	5/93	EDN1 FOXC1 SMO SOX9 SOX11
6_Member	GO Biological Processes	GO:0021510	spinal cord development	5.83E-03	4.63E-02	5/99	SMO SOX4 SOX11 VLDLR NOG
6_Member	GO Biological Processes	GO:0030902	hindbrain development	1.32E-02	9.41E-02	5/146	FOXC1 SMO SPTBN2 HNF1B NOG
7_Summary	GO Biological Processes	GO:0043588	skin development	1.11E-04	1.50E-03	10/235	CDH3 DSP GATA6 ITGA6 IRF6 ITGB4 SMO SOX9 ST14 TGFB2
7_Member	GO Biological Processes	GO:0043588	skin development	1.11E-04	1.50E-03	10/235	CDH3 DSP GATA6 ITGA6 IRF6 ITGB4 SMO SOX9 ST14 TGFB2
7_Member	GO Biological Processes	GO:0008544	epidermis development	2.35E-03	2.14E-02	8/280	CDH3 DSP GATA6 IRF6 SMO SOX9 ST14 TGFB2
8_Summary	GO Biological Processes	GO:0055123	digestive system development	2.25E-04	2.79E-03	8/139	CDKN1C GATA6 SALL1 SMO SOX11 HNF1B TGFB2 DACT1 IRF6
8_Member	GO Biological Processes	GO:0055123	digestive system development	2.25E-04	2.79E-03	8/139	CDKN1C GATA6 SALL1 SMO SOX11 HNF1B TGFB2 DACT1
8_Member	GO Biological Processes	GO:0048565	digestive tract development	6.91E-04	7.37E-03	7/127	GATA6 SALL1 SMO SOX11 HNF1B TGFB2 DACT1
8_Member	GO Biological Processes	GO:0048566	embryonic digestive tract development	3.24E-03	2.83E-02	4/33	SALL1 SOX11 HNF1B TGFB2
9_Summary	GO Biological Processes	GO:0003209	cardiac atrium morphogenesis	3.86E-04	4.51E-03	5/29	BMP2 SMO SOX4 TGFB2 NOG SOX9 ADAMTS9 CCND2 EDN1 ITGA6 MITF PAX8 IKBKG SOX11 PPM1D CASK RIPK4 POMK SIK1 IL11 VLDLR GDF5 CDKN1C EEF1A2 RUBCN TUBB2B MID1 DACT1
9_Member	GO Biological Processes	GO:0003209	cardiac atrium morphogenesis	3.86E-04	4.51E-03	5/29	BMP2 SMO SOX4 TGFB2 NOG
9_Member	GO Biological Processes	GO:0003230	cardiac atrium development	6.74E-04	7.23E-03	5/37	BMP2 SMO SOX4 TGFB2 NOG
9_Member	GO Biological Processes	GO:0003179	heart valve morphogenesis	1.63E-03	1.56E-02	5/55	BMP2 SOX4 SOX9 TGFB2 ADAMTS9
9_Member	GO Biological Processes	GO:0003170	heart valve development	2.36E-03	2.14E-02	5/65	BMP2 SOX4 SOX9 TGFB2 ADAMTS9
9_Member	GO Biological Processes	GO:0060413	atrial septum morphogenesis	6.20E-03	4.85E-02	3/15	SMO SOX4 TGFB2
9_Member	KEGG Pathway	hsa05200	Pathways in cancer	7.48E-03	5.73E-02	9/531	BMP2 CCND2 EDN1 ITGA6 MITF SMO TGFB2 PAX8 IKBKG
9_Member	GO Biological Processes	GO:0003283	atrial septum development	1.11E-02	8.11E-02	3/23	SMO SOX4 TGFB2
9_Member	GO Biological Processes	GO:0003181	atrioventricular valve morphogenesis	1.18E-02	8.51E-02	3/24	BMP2 SOX4 TGFB2
9_Member	GO Biological Processes	GO:0003171	atrioventricular valve development	1.38E-02	9.72E-02	3/27	BMP2 SOX4 TGFB2
10_Summary	GO Biological Processes	GO:0002065	columnar/cuboidal epithelial cell differentiation	7.96E-04	8.32E-03	6/79	BMP2 GATA6 SMO SOX4 SOX11 B9D1 EDN1 ALDH1A3 IL11 HNF1B PAX8 TGFB2 CASK MYRF L1CAM NOG CDKN1C
10_Member	GO Biological Processes	GO:0002065	columnar/cuboidal epithelial cell differentiation	7.96E-04	8.32E-03	6/79	BMP2 GATA6 SMO SOX4 SOX11 B9D1
10_Member	GO Biological Processes	GO:0060563	neuroepithelial cell differentiation	1.30E-03	1.27E-02	4/20	BMP2 SOX4 SOX11 B9D1
10_Member	GO Biological Processes	GO:0003357	noradrenergic neuron differentiation	4.00E-03	3.33E-02	3/11	EDN1 SOX4 SOX11
10_Member	GO Biological Processes	GO:0003207	cardiac chamber formation	5.08E-03	4.10E-02	3/13	BMP2 SOX4 SOX11
11_Summary	GO Biological Processes	GO:2000738	positive regulation of stem cell differentiation	1.30E-03	1.27E-02	4/20	FOXC1 GATA6 SOX9 TGFB2 CDKN1C EDN1 SALL1 SMO SPTBN2 HNF1B ST14 NOG ADGRG1 BMP2 IRF6 SOX4 SOX11 GDF5 CASK ADAMTS9
11_Member	GO Biological Processes	GO:2000738	positive regulation of stem cell differentiation	1.30E-03	1.27E-02	4/20	FOXC1 GATA6 SOX9 TGFB2
11_Member	GO Biological Processes	GO:0048589	developmental growth	2.07E-03	1.91E-02	9/369	CDKN1C EDN1 FOXC1 SALL1 SMO SOX9 SPTBN2 HNF1B TGFB2
11_Member	GO Biological Processes	GO:0040007	growth	2.11E-03	1.94E-02	9/371	CDKN1C EDN1 FOXC1 SALL1 SMO SOX9 SPTBN2 HNF1B TGFB2
11_Member	GO Biological Processes	GO:0048608	reproductive structure development	3.26E-03	2.85E-02	9/419	CDKN1C FOXC1 GATA6 SALL1 SOX9 ST14 TGFB2 NOG ADGRG1
11_Member	GO Biological Processes	GO:0061458	reproductive system development	3.35E-03	2.90E-02	9/422	CDKN1C FOXC1 GATA6 SALL1 SOX9 ST14 TGFB2 NOG ADGRG1
11_Member	GO Biological Processes	GO:2000736	regulation of stem cell differentiation	9.81E-03	7.26E-02	4/62	FOXC1 GATA6 SOX9 TGFB2
11_Member	GO Biological Processes	GO:0008406	gonad development	1.03E-02	7.59E-02	6/217	FOXC1 GATA6 SALL1 SOX9 TGFB2 ADGRG1
11_Member	GO Biological Processes	GO:0045137	development of primary sexual characteristics	1.09E-02	7.97E-02	6/222	FOXC1 GATA6 SALL1 SOX9 TGFB2 ADGRG1
11_Member	GO Biological Processes	GO:0008283	cell population proliferation	1.37E-02	9.70E-02	8/489	BMP2 FOXC1 IRF6 SOX4 SOX9 SOX11 HNF1B TGFB2
12_Summary	GO Biological Processes	GO:0048638	regulation of developmental growth	1.37E-03	1.33E-02	9/329	EDN1 FOXC1 GATA6 L1CAM SMO HNF1B GDF5 NOG NEDD4L PAX2 ST14 TGFB2 VLDLR DCDC2 SHROOM3 ROBO3 ITGB4 TNNT1 PAX8
12_Member	GO Biological Processes	GO:0048638	regulation of developmental growth	1.37E-03	1.33E-02	9/329	EDN1 FOXC1 GATA6 L1CAM SMO HNF1B GDF5 NOG NEDD4L
12_Member	GO Biological Processes	GO:0000902	cell morphogenesis	3.22E-03	2.82E-02	11/673	EDN1 L1CAM PAX2 SMO ST14 TGFB2 VLDLR NOG DCDC2 SHROOM3 ROBO3
12_Member	GO Biological Processes	GO:0032989	cellular component morphogenesis	4.07E-03	3.37E-02	10/573	EDN1 ITGB4 L1CAM PAX2 SMO TNNT1 VLDLR NOG DCDC2 ROBO3
12_Member	GO Biological Processes	GO:0046620	regulation of organ growth	6.09E-03	4.79E-02	5/101	EDN1 FOXC1 GATA6 SMO NOG
12_Member	GO Biological Processes	GO:0040008	regulation of growth	6.96E-03	5.39E-02	10/660	EDN1 FOXC1 GATA6 L1CAM SMO HNF1B TGFB2 GDF5 NOG NEDD4L
12_Member	GO Biological Processes	GO:0000904	cell morphogenesis involved in differentiation	7.05E-03	5.45E-02	9/522	EDN1 L1CAM PAX2 SMO ST14 VLDLR NOG DCDC2 ROBO3
12_Member	GO Biological Processes	GO:0048667	cell morphogenesis involved in neuron differentiation	7.99E-03	6.11E-02	8/411	EDN1 L1CAM PAX2 SMO VLDLR NOG DCDC2 ROBO3
12_Member	GO Biological Processes	GO:0071371	cellular response to gonadotropin stimulus	9.20E-03	6.87E-02	3/20	EDN1 GATA6 PAX8
12_Member	GO Biological Processes	GO:0048812	neuron projection morphogenesis	1.10E-02	8.01E-02	8/455	EDN1 L1CAM PAX2 SMO VLDLR NOG DCDC2 ROBO3
12_Member	GO Biological Processes	GO:0120039	plasma membrane bounded cell projection morphogenesis	1.13E-02	8.20E-02	8/459	EDN1 L1CAM PAX2 SMO VLDLR NOG DCDC2 ROBO3

12_Member	GO Biological Processes	GO:0048858	cell projection morphogenesis	1.16E-02	8.39E-02	8/463	EDN1	L1CAM	PAX2	SMO	VLDLR	NOG	DCDC2	ROBO3											
12_Member	GO Biological Processes	GO:0032990	cell part morphogenesis	1.31E-02	9.36E-02	8/482	EDN1	L1CAM	PAX2	SMO	VLDLR	NOG	DCDC2	ROBO3											
13_Summary	GO Biological Processes	GO:0030111	regulation of Wnt signaling pathway	1.47E-03	1.42E-02	9/336	BMP2	CDH3	SALL1	SOX4	SOX9	HNF1B	NOG	DACT1	DCDC2	SMO	NEDD4L	H4C11							
13_Member	GO Biological Processes	GO:0030111	regulation of Wnt signaling pathway	1.47E-03	1.42E-02	9/336	BMP2	CDH3	SALL1	SOX4	SOX9	HNF1B	NOG	DACT1	DCDC2										
13_Member	GO Biological Processes	GO:0030177	positive regulation of Wnt signaling pathway	1.28E-02	9.20E-02	5/144	BMP2	CDH3	SALL1	SOX4	DACT1														
14_Summary	GO Biological Processes	GO:0045165	cell fate commitment	1.50E-03	1.44E-02	8/244	BMP2	EDN1	GATA6	MITF	PAX2	SMO	SOX9	HNF1B	SOX4	CDH3	TGFB2	FOXC1	MYO5B	CIB2	IRF6	ST14	SHROOM3	ADGRG1	
14_Member	GO Biological Processes	GO:0045165	cell fate commitment	1.50E-03	1.44E-02	8/244	BMP2	EDN1	GATA6	MITF	PAX2	SMO	SOX9	HNF1B											
14_Member	GO Biological Processes	GO:0031016	pancreas development	3.12E-03	2.75E-02	5/74	GATA6	SMO	SOX4	SOX9	HNF1B														
14_Member	GO Biological Processes	GO:0031018	endocrine pancreas development	4.16E-03	3.44E-02	4/38	GATA6	SMO	SOX4	SOX9															
14_Member	GO Biological Processes	GO:0051797	regulation of hair follicle development	8.58E-03	6.47E-02	3/19	CDH3	SMO	TGFB2																
15_Summary	GO Biological Processes	GO:0001704	formation of primary germ layer	2.06E-03	1.91E-02	6/114	FOXC1	GATA6	ITGB4	PAX2	HNF1B	NOG	PAX8	SMO	DNMT3B	KDM6A	SOX9								
15_Member	GO Biological Processes	GO:0001704	formation of primary germ layer	2.06E-03	1.91E-02	6/114	FOXC1	GATA6	ITGB4	PAX2	HNF1B	NOG													
15_Member	GO Biological Processes	GO:0048645	animal organ formation	3.78E-03	3.18E-02	4/36	GATA6	PAX2	PAX8	NOG															
15_Member	GO Biological Processes	GO:0007369	gastrulation	4.93E-03	4.00E-02	6/161	FOXC1	GATA6	ITGB4	PAX2	HNF1B	NOG													
15_Member	GO Biological Processes	GO:0007498	mesoderm development	9.23E-03	6.88E-02	5/123	FOXC1	ITGB4	PAX2	SMO	NOG														
15_Member	GO Biological Processes	GO:0001707	mesoderm formation	1.15E-02	8.36E-02	4/68	FOXC1	ITGB4	PAX2	NOG															
15_Member	GO Biological Processes	GO:0048332	mesoderm morphogenesis	1.21E-02	8.71E-02	4/70	FOXC1	ITGB4	PAX2	NOG															
15_Member	GO Biological Processes	GO:0060795	cell fate commitment involved in formation of primary germ layer	1.38E-02	9.72E-02	3/27	GATA6	PAX2	HNF1B																
16_Summary	Reactome Gene Sets	R-HSA-1474244	Extracellular matrix organization	2.97E-03	2.65E-02	8/301	BMP2	ITGA6	ITGB4	MATN3	TGFB2	GDF5	CASK	ADAMTS9	GPC4	IL11	ST14	CHRDL1							
16_Member	Reactome Gene Sets	R-HSA-1474244	Extracellular matrix organization	2.97E-03	2.65E-02	8/301	BMP2	ITGA6	ITGB4	MATN3	TGFB2	GDF5	CASK	ADAMTS9											
17_Summary	GO Biological Processes	GO:0032909	regulation of transforming growth factor beta2 production	2.99E-03	2.65E-02	3/9	CDH3	GATA6	TGFB2	ALDH1A3	BMP2	EEF1A2	ITGA6	KCNMA1	SOX4	SIK1	EDN1	FOXC1	SMO	SOX9	TNNT1	L1CAM	TUBB4A	TUBB2B	HNF1B
17_Member	GO Biological Processes	GO:0032909	regulation of transforming growth factor beta2 production	2.99E-03	2.65E-02	3/9	CDH3	GATA6	TGFB2																
17_Member	GO Biological Processes	GO:0043065	positive regulation of apoptotic process	7.38E-03	5.68E-02	9/529	ALDH1A3	BMP2	EEF1A2	GATA6	ITGA6	KCNMA1	SOX4	TGFB2	SIK1										
17_Member	GO Biological Processes	GO:0035051	cardiocyte differentiation	8.01E-03	6.11E-02	5/115	BMP2	EDN1	GATA6	TGFB2	SIK1														
17_Member	GO Biological Processes	GO:0043068	positive regulation of programmed cell death	8.07E-03	6.14E-02	9/543	ALDH1A3	BMP2	EEF1A2	GATA6	ITGA6	KCNMA1	SOX4	TGFB2	SIK1										
17_Member	GO Biological Processes	GO:0042476	odontogenesis	8.46E-03	6.42E-02	5/118	BMP2	FOXC1	GATA6	SMO	TGFB2														
17_Member	GO Biological Processes	GO:1902893	regulation of pri-miRNA transcription by RNA polymerase II	9.01E-03	6.76E-02	4/59	BMP2	GATA6	SOX9	TGFB2															
17_Member	GO Biological Processes	GO:0010942	positive regulation of cell death	1.16E-02	8.40E-02	9/604	ALDH1A3	BMP2	EEF1A2	GATA6	ITGA6	KCNMA1	SOX4	TGFB2	SIK1										
18_Summary	GO Biological Processes	GO:0060174	limb bud formation	4.00E-03	3.33E-02	3/11	SOX4	SOX9	SOX11	NOG	ALDH1A3	GATA6	BMP2	MYRF	FOXC1										
18_Member	GO Biological Processes	GO:0060174	limb bud formation	4.00E-03	3.33E-02	3/11	SOX4	SOX9	SOX11																
19_Summary	GO Biological Processes	GO:0009071	serine family amino acid catabolic process	5.63E-03	4.50E-02	3/14	CBS	GCSH	GLDC																
19_Member	GO Biological Processes	GO:0009071	serine family amino acid catabolic process	5.63E-03	4.50E-02	3/14	CBS	GCSH	GLDC																
20_Summary	GO Biological Processes	GO:0051091	positive regulation of DNA-binding transcription factor activity	5.84E-03	4.63E-02	7/264	BMP2	EDN1	SMO	IKBKG	DDX58	RIPK4	TRAPPC9	SIK1											
20_Member	GO Biological Processes	GO:0051091	positive regulation of DNA-binding transcription factor activity	5.84E-03	4.63E-02	7/264	BMP2	EDN1	SMO	IKBKG	DDX58	RIPK4	TRAPPC9												
20_Member	GO Biological Processes	GO:0051090	regulation of DNA-binding transcription factor activity	1.07E-02	7.83E-02	8/451	BMP2	EDN1	SMO	IKBKG	DDX58	RIPK4	TRAPPC9	SIK1											

Supplementary Table 7. Number of candidate splicing outliers and the corresponding ranks per proband for each described family.

sampleID	seqnames	start	end	hgncSymbol	type	pValue	padjust	zScore	psiValue	deltaPsi	meanCounts	meanTotalCounts	counts	totalCounts
Family 1 - twin 1	chr19	682145	682146	*FSTL3*	theta	1.95E-11	4.6163E-06	-4.05	0.51	-0.48	1337.62	1374.43	648	1266
Family 1 - twin 1	chr2	133403853	133403854	*GPR39*	theta	2.88E-10	5.68E-05	3.98	0.72	0.64	38.05	1141.82	34	47
Family 1 - twin 1	chr5	83525703	83549901	*EDIL3*	psi3	4.83E-10	9.00E-05	-4	0.57	-0.41	399.91	411.96	419	732
Family 1 - twin 1	chr12	109018089	109046060	*SELPLG*	psi3	1.34E-09	0.00015702	5.19	0.67	0.61	2.77	35.89	155	230
Family 1 - twin 1	chr21	44324387	44326949	*NDUFV3*	psi5	6.44E-09	0.00050256	2.32	0.98	0.75	28.54	283.86	238	244
Family 1 - twin 1	chr11	62293201	62297352	*AHNAK*	psi3	7.44E-09	0.00059404	-1.18	0.11	-0.72	291.14	557.05	16	146
**Family 1 - twin 1**	**chr8**	**61768762**	**61769003**	***CHD7***	**psi3**	**3.50E-08**	**0.0020554**	**-4.35**	**0.29**	**-0.62**	**37.2**	**37.75**	**6**	**21**
Family 1 - twin 1	chr14	105408807	105414251	*AHNAK2*	psi3	1.63E-07	0.0072648	3.49	0.97	0.54	38.27	198.05	65	67
Family 1 - twin 1	chr11	62334977	62338436	*EEF1G*	psi3	2.46E-07	0.01017	-2.25	0	-0.46	37.02	37.55	0	26
Family 1 - twin 1	chr17	41151436	41151437	*RPL27*	theta	2.84E-07	0.024169	-4.58	0.01	-0.55	6.93	8.25	1	75
Family 1 - twin 1	chr1	9304991	9304992	*H6PD*	theta	4.08E-07	0.033015	-3.82	0.32	-0.63	55.96	58	12	38
Family 1 - twin 2	chr5	83525703	83549901	*EDIL3*	psi3	2.89E-10	8.06E-05	-3.68	0.56	-0.42	399.91	411.96	328	588
Family 1 - twin 2	chr11	62293201	62297352	*AHNAK*	psi3	4.32E-09	0.00053592	-1.26	0.01	-0.32	291.14	557.05	2	315
Family 1 - twin 2	chr22	39410385	39445429	*APOBEC3C*	psi3	9.78E-09	0.001093	4.43	0.54	0.48	0.68	36	38	70
Family 1 - twin 2	chr22	39441548	39445429	*APOBEC3F*	psi3	9.78E-09	0.001093	-3.1	0.31	-0.56	34.29	36	22	70
Family 1 - twin 2	chr5	69517843	69851044	*AC138866.1*	psi5	1.09E-08	0.0013729	4.37	0.46	0.43	0.77	103.64	13	28
Family 1 - twin 2	chr2	242512269	242512348	*BOK*	psi5	2.03E-08	0.0020876	3.18	1	0.92	9.3	179.64	37	37
**Family 1 - twin 2**	**chr8**	**61768762**	**61769003**	***CHD7***	**psi3**	**3.57E-08**	**0.0036225**	**-4.22**	**0.2**	**-0.66**	**37.2**	**37.75**	**4**	**20**
Family 1 - twin 2	chr20	17639830	17640339	*RRBP1*	psi5	1.83E-07	0.013778	2.9	0.93	0.82	3.77	69.43	67	72
Family 1 - twin 2	chr1	1398089	1444584	*ATAD3C*	psi5	5.10E-07	0.02741	3.96	1	0.64	0.46	13.23	22	22
Family 1 - twin 2	chr13	30110340	30169552	*SLC7A1*	psi5	7.55E-07	0.037084	3.26	0.51	0.47	7.73	207.96	61	120
Family 1 - twin 2	chr7	102129046	102131432	*RASA4B*	psi3	9.31E-07	0.047433	3.44	0.95	0.87	15.43	65.61	244	256
Family 2	chr1	40747193	40747194	*ZMPSTE24*	theta	2.14E-18	2.54E-12	-6.53	0.38	-0.61	211.12	214.68	121	320
Family 2	chr14	105413757	105414251	*AHNAK2*	psi5	9.32E-17	1.05E-10	5.16	1	0.94	1.96	179.95	21	21
**Family 2**	**chr11**	**61546734**	**61546735**	***MYRF***	**theta**	**1.19E-09**	**0.00020211**	**2.63**	**0.43**	**0.31**	**5.82**	**360.64**	**60**	**141**
Family 2	chr22	29686401	29686402	*EWSR1*	theta	1.02E-08	0.001288	-5.38	0.59	-0.39	45.48	47.95	185	312
Family 2	chr17	45566322	45567531	*MRPL45P2*	psi3	2.85E-08	0.001871	-3.78	0.13	-0.71	22.93	23.64	3	23
Family 2	chr12	121840544	121840545	*RNF34*	theta	6.47E-08	0.0059299	4.7	0.68	0.59	0.41	20.59	13	19
Family 2	chr12	112306666	112308074	*MAPKAPK5*	psi5	3.30E-07	0.011645	-3.28	0.48	-0.43	105.57	113.3	65	136
Family 3	chr10	120801787	120801788	*EIF3A*	theta	8.12E-19	1.92E-12	-4.73	0.18	-0.8	700.14	723.95	242	1325
**Family 3**	**chr7**	**94048864**	**94049702**	***COL1A2***	**psi3**	**2.63E-18**	**2.94E-12**	**3.51**	**0.6**	**0.59**	**8.41**	**801.39**	**471**	**785**
Family 3	chr1	144520954	144520955	*AL592284.1*	theta	2.28E-13	7.73E-08	5.97	0.87	0.71	44.64	642.38	27	31
Family 3	chr11	62289220	62289603	*AHNAK*	psi3	8.24E-13	3.07E-07	1.18	0.42	0.4	77.07	898.73	201	473
Family 3	chr16	2014470	2014544	*RPS2*	psi5	4.06E-12	4.17E-07	3.24	1	0.92	22.82	372.34	126	126
Family 3	chr9	135526504	135527834	*DDX31*	psi3	4.79E-11	8.91E-06	-6.44	0.68	-0.31	93.09	93.54	52	76
Family 3	chr1	143744381	143744382	*RP6-206I17.1*	theta	1.10E-10	2.18E-05	6.34	0.99	0.55	111.14	380.71	92	93
Family 3	chr2	97864229	97864322	*ANKRD36*	psi5	5.70E-09	0.00027229	-2.68	0.09	-0.62	13.34	18.93	8	91
Family 3	chr11	82905771	82909520	*ANKRD42*	psi5	4.18E-08	0.0016265	-5.04	0.53	-0.44	32.23	32.59	17	32
Family 3	chr19	54710942	54710943	*RPS9*	theta	2.83E-07	0.02486	-5.07	0.46	-0.49	15.71	16.36	31	67
Family 3	chr3	118955918	118959611	*B4GALT4*	psi3	3.56E-07	0.012036	3.9	0.67	0.63	2.36	40.68	41	61
Family 3	chr7	39664027	39726229	*RALA*	psi5	4.24E-07	0.01212	-3.53	0.22	-0.64	11.64	13.02	6	27
Family 3	chr13	45914256	45914300	*TPT1*	psi3	4.30E-07	0.01414	-1.91	0.56	-0.43	410.41	423.29	426	757
Family 3	chr19	35169719	35173681	*ZNF302*	psi3	1.21E-06	0.031461	-3.77	0.64	-0.33	40.5	41.41	38	59
Family 3	chr1	3655972	3656115	*TP73-AS1*	psi3	1.32E-06	0.03279	3.68	0.44	0.41	0.93	41.66	32	72

Supplementary Table 8. Number of candidate expression outliers and the corresponding ranks per proband for each described family.

hgncSymbol	geneID	sampleID	pValue	padjust	zScore	l2fc	rawcounts	normcounts	meanCorrected	theta	aberrant	AberrantBySample	AberrantByGene	padj_rank	foldChange
MIR4458HG	ENSG00000247516.6_5	Family 1 - twin 1	5.60E-09	0.000920448	-6.64	-5.91	1	1.5	178.42	7.83	TRUE	2	1	1	0.02
SOX11	ENSG00000176887.6_3	Family 1 - twin 1	4.24E-07	0.034816995	-5.59	-2.16	95	378.04	1707.41	19.78	TRUE	2	1	2	0.22
WDR74	ENSG00000133316.15_4	Family 2	8.54E-09	0.001403196	5.3	1.25	3787	3999.15	1678.74	32.14	TRUE	1	1	1	2.38

Supplementary Figures:

Supplementary Figure 1. Number of expressed genes across 56 AF cells.

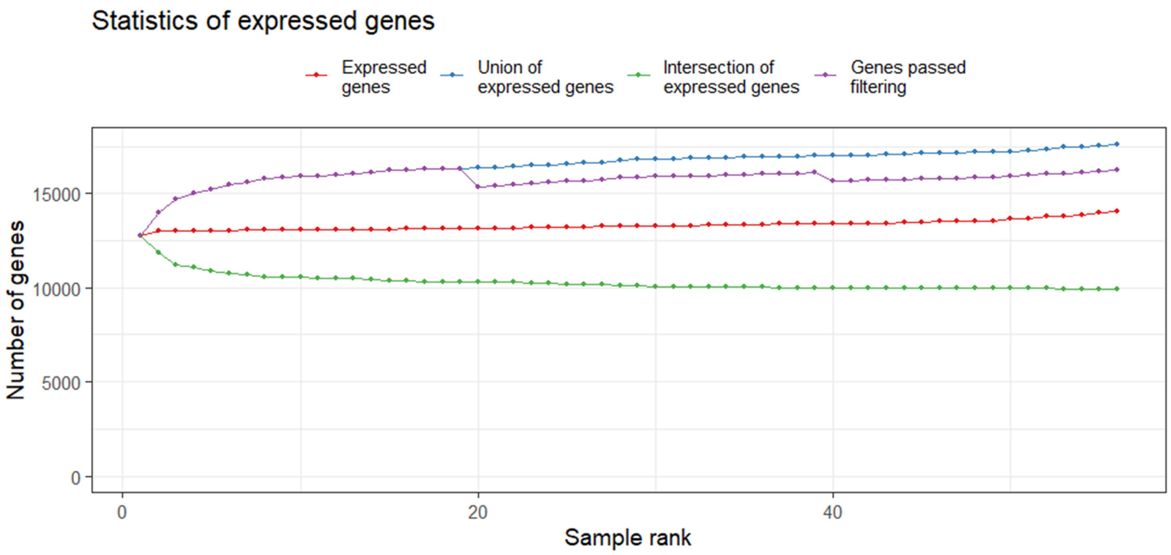

Genes expressed in each sample (red), union of all detected genes (blue), intersection of expressed genes (green), and genes that passed the minimum fragments per kilobase of transcript per million mapped fragments (FPKM) filter as a group (violet). Genes with a 95th percentile FPKM <1 were not considered to be sufficiently expressed and were therefore filtered out. Over 12,500 genes passed the quality filter.

Supplementary Figure 2. Correlation of the median transcript per million (TPM) per gene between the GTEx fibroblasts and 76 control fibroblast datasets processed by the in-house adaptation of DROP.

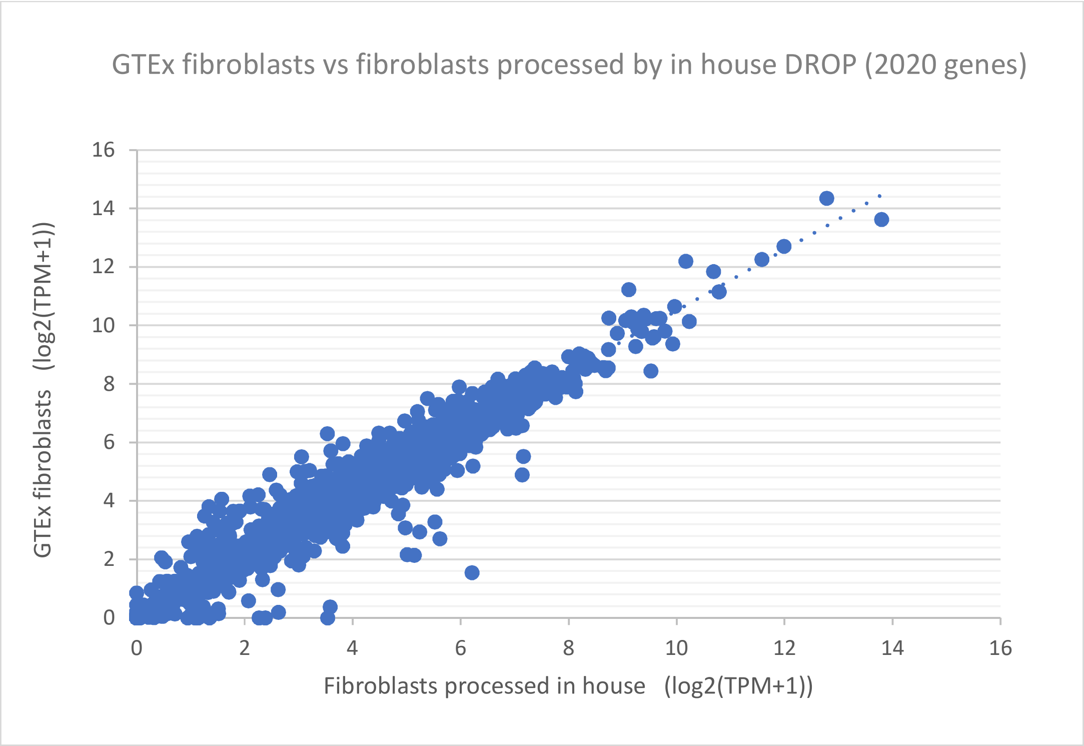

BAM files for 76 control fibroblast datasets were downloaded from the NCBI dbGaP and processed by the adapted DROP. Median TPMs were extracted for comparison with GTEx control fibroblast data. The comparison of the TPM values between GTEx control fibroblasts and in-house fibroblasts demonstrated a high correlation with $R^2 = 94.9\%$ and $p<0.001$.

Supplementary Figure 3. Number of expressed genes in GTEx whole blood, GTEx fibroblasts, and AF cells for (A) cardiac genes (n=33), and (B) skeletal genes (n=423).

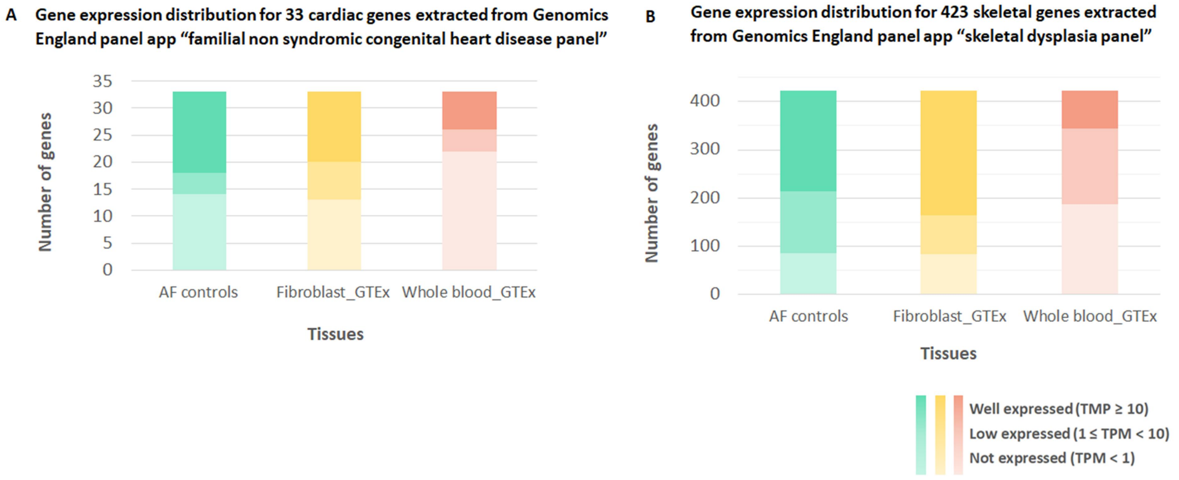

Supplementary Figure 4. Venn diagram of well-expressed genes (TPM≥10) in GTEx whole blood, GTEx fibroblasts, and AF cells for (A) 33 cardiac genes, and (B) 423 skeletal genes.

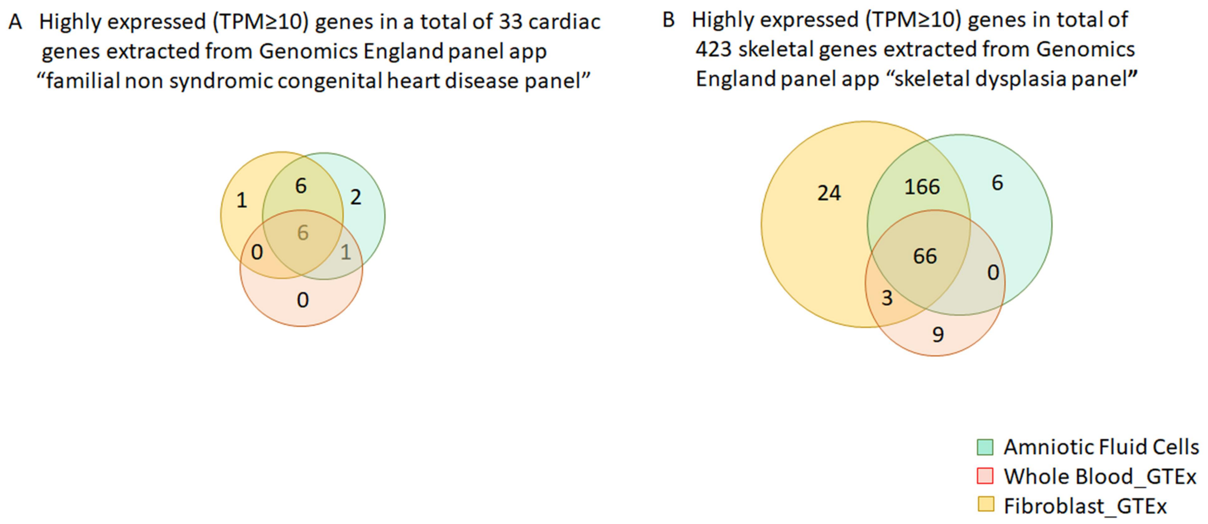

Supplementary Figure 5. Gene expression across all GTEx whole blood (n=504), GTEx fibroblasts (n=755), and AF cells (n=52) for the three prenatal cases including that of (A) *CHD7*, (B) *MYRF*, and (C) *COL1A2*. Centre lines represent the median, bounds of box represent interquartile range and whiskers represent 95% confidence interval.

A Expression level of *CHD7* across all three tissues

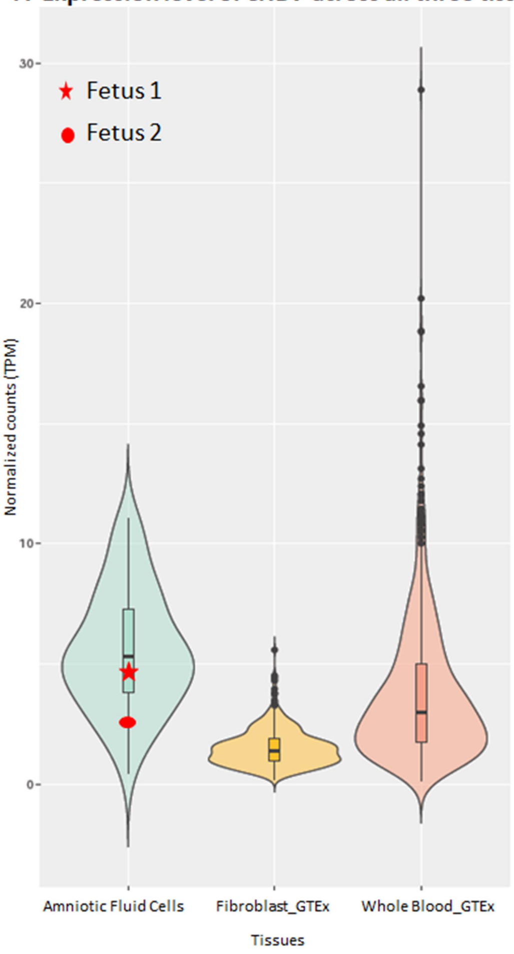

B Expression level of *MYRF* across all three tissues

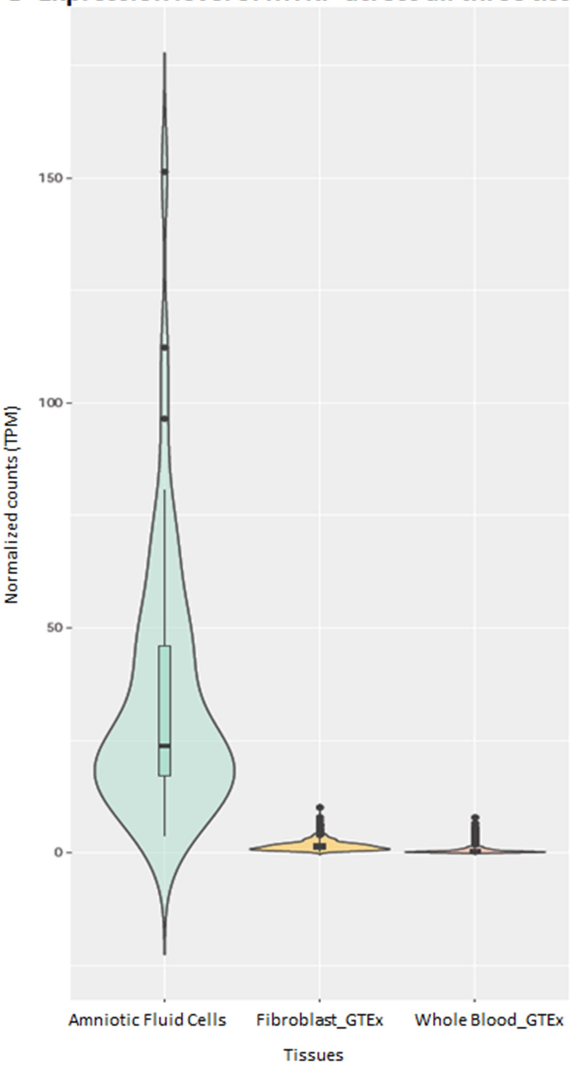

C Expression level of *COL1A2* across all three tissues

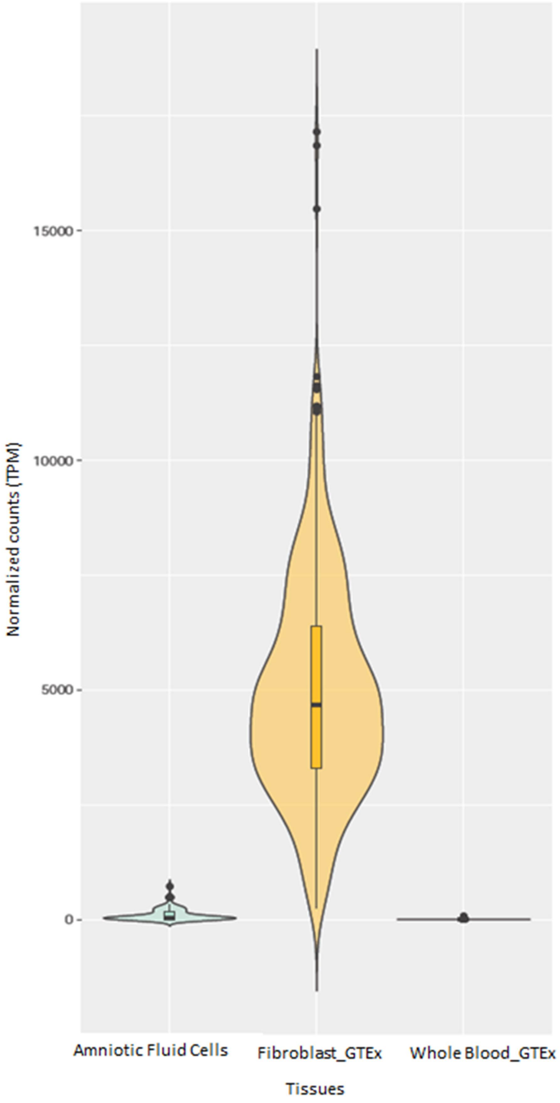

Supplementary Figure 6. Principal component analysis (PCA) plot based on the gene expression profiles of AF cells (n=48), GTEx fibroblasts (n= 755), GTEx muscle (n=803), GTEx whole blood (n=504), whole blood from live participants[1] (n=125) and iPSCs[2] (n=330).

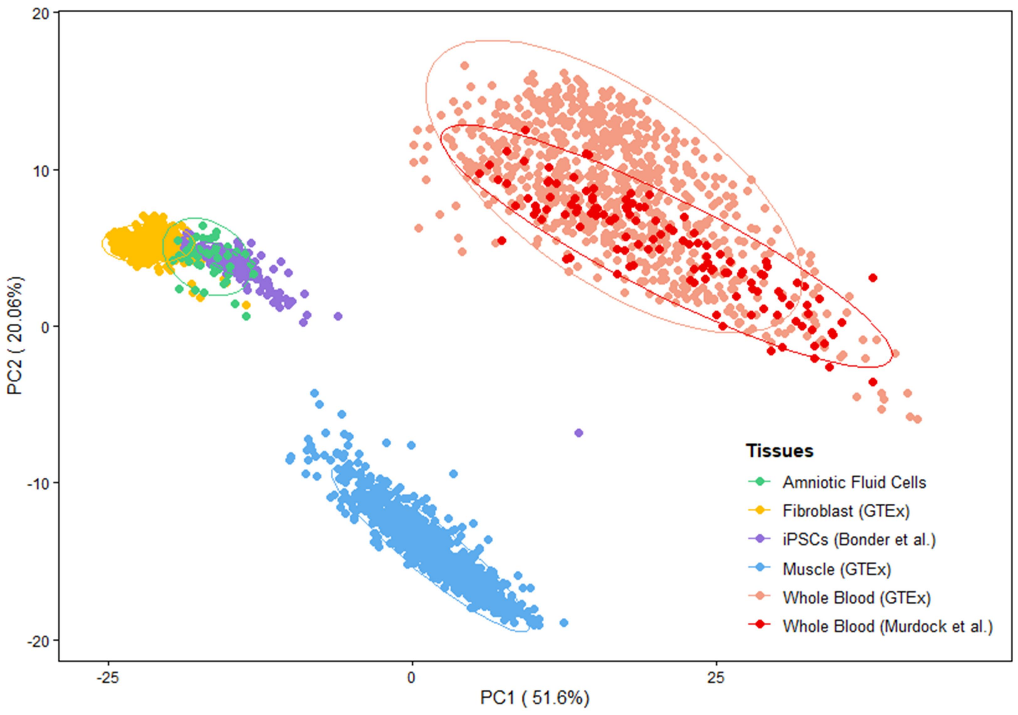

Supplementary References

1. Murdock DR, Dai H, Burrage LC, et al. Transcriptome-directed analysis for Mendelian disease diagnosis overcomes limitations of conventional genomic testing. *J Clin Invest*. Jan 4 2021;131(1)doi:10.1172/JCI141500
2. Bonder MJ, Smail C, Gloudemans MJ, et al. Identification of rare and common regulatory variants in pluripotent cells using population-scale transcriptomics. *Nat Genet*. Mar 2021;53(3):313-321. doi:10.1038/s41588-021-00800-7
