# Supplementary material for: Diagnostic potential of the amniotic fluid cells transcriptome in deciphering mendelian disease: a proof-of-concept
Source: NPJ Genom Med. 2022 Dec 28;7:74. doi: 10.1038/s41525-022-00347-4 (PMC9797484; doi:10.1038/s41525-022-00347-4)
Supplement: Supplementary file 2 — Reporting Summary Checklist [file 41525_2022_347_MOESM2_ESM.pdf]

nature portfolio

Corresponding author(s): Brian Hon Yin Chung and Anita Sik Yau Kan

Last updated by author(s): Sep 24, 2022

Reporting Summary

Nature Portfolio wishes to improve the reproducibility of the work that we publish. This form provides structure for consistency and transparency in reporting. For further information on Nature Portfolio policies, see our Editorial Policies and the Editorial Policy Checklist.

Statistics

For all statistical analyses, confirm that the following items are present in the figure legend, table legend, main text, or Methods section.

n/a	Confirmed	
☐	☒	The exact sample size (*n*) for each experimental group/condition, given as a discrete number and unit of measurement
☐	☒	A statement on whether measurements were taken from distinct samples or whether the same sample was measured repeatedly
☐	☒	The statistical test(s) used AND whether they are one- or two-sided<br>*Only common tests should be described solely by name; describe more complex techniques in the Methods section.*
☐	☒	A description of all covariates tested
☐	☒	A description of any assumptions or corrections, such as tests of normality and adjustment for multiple comparisons
☐	☒	A full description of the statistical parameters including central tendency (e.g. means) or other basic estimates (e.g. regression coefficient) AND variation (e.g. standard deviation) or associated estimates of uncertainty (e.g. confidence intervals)
☐	☒	For null hypothesis testing, the test statistic (e.g. *F*, *t*, *r*) with confidence intervals, effect sizes, degrees of freedom and *P* value noted<br>*Give P values as exact values whenever suitable.*
☒	☐	For Bayesian analysis, information on the choice of priors and Markov chain Monte Carlo settings
☒	☐	For hierarchical and complex designs, identification of the appropriate level for tests and full reporting of outcomes
☒	☐	Estimates of effect sizes (e.g. Cohen's *d*, Pearson's *r*), indicating how they were calculated

Our web collection on statistics for biologists contains articles on many of the points above.

Software and code

Policy information about availability of computer code

Data collection	no software was used in the data collection
Data analysis	DROP v1.1.0 and R (v4.1.2), details are provided in the Method section.

For manuscripts utilizing custom algorithms or software that are central to the research but not yet described in published literature, software must be made available to editors and reviewers. We strongly encourage code deposition in a community repository (e.g. GitHub). See the Nature Portfolio guidelines for submitting code & software for further information.

Data

Policy information about availability of data

All manuscripts must include a data availability statement. This statement should provide the following information, where applicable:

- Accession codes, unique identifiers, or web links for publicly available datasets
- A description of any restrictions on data availability
- For clinical datasets or third party data, please ensure that the statement adheres to our policy

Gene expression count matrices, as well as the privacy preserving count matrices of split and unsplit reads overlapping annotated splice sites from RNA-seq, are available for download without restriction from the Zenodo repository (https://zenodo.org/record/7079684#.YyLGoXZByUl). The codes used to process the data in this study were deposited in GitHub (https://github.com/gagneurlab/drop) with no access restrictions.

Human research participants

Policy information about studies involving human research participants and Sex and Gender in Research.

Reporting on sex and gender	We recruited 52 fetuses, comprising 23 females and 29 males.
Population characteristics	We recruited 52 fetuses, comprising 23 females and 29 males. All 52 AF samples were obtained from pregnant women with a gestational age ranging from 16 weeks and 0 days to 21 weeks and 3 days between August 2020 and May 2021, except for one AF sample retrieved from a pregnancy in 2017. Forty-eight AF samples serve as the non-diseased controls and four AF samples were used for validation.
Recruitment	AF was collected from pregnant women under the care of Tsan Yuk Hospital, Hong Kong, who opted for amniocenteses during their second trimester based on either high-risk Down syndrome screening test results or abnormal ultrasound findings. For non-diseased samples, fetuses with abnormal ultrasound findings were excluded. Chromosomal microarray confirmed whether the fetus was euploid and a healthy pregnancy was expected to continue.<br>For validation samples, we studied four pregnancies from three families (Family 1 had two affected pregnancies) with known splicing variants detected by WES. AF cells from these affected pregnancies were either retrieved from the stored sample (n = 1 from a previous pregnancy in 2017) or were freshly obtained during prenatal testing (n = 3).
Ethics oversight	The study was approved by the institutional review board of the University of Hong Kong/Hospital Authority Hong Kong West Cluster (UW11-190 and UW12-211).

Note that full information on the approval of the study protocol must also be provided in the manuscript.

Field-specific reporting

Please select the one below that is the best fit for your research. If you are not sure, read the appropriate sections before making your selection.

- [x] Life sciences
- [ ] Behavioural & social sciences
- [ ] Ecological, evolutionary & environmental sciences

For a reference copy of the document with all sections, see nature.com/documents/nr-reporting-summary-flat.pdf

Life sciences study design

All studies must disclose on these points even when the disclosure is negative.

Sample size	52
Data exclusions	All 52 samples were used
Replication	48 controls were used, RNA-seq results were verified by alternative methods (i.e. WES and RT-PCR)
Randomization	Not relevant to this study. All non diseased control and case samples were run together with the outlier approach, DROP. The rationale of this approach is based on the comparison of one patient sample against the rest of the samples in the cohort as internal controls. This comparison allows for the identification of significant aberrant outliers
Blinding	Not relevant to this study. All non diseased control and case samples were run together with the outlier approach, DROP. The rationale of this approach is based on the comparison of one patient sample against the rest of the samples in the cohort as internal controls. This comparison allows for the identification of significant aberrant outliers

Reporting for specific materials, systems and methods

We require information from authors about some types of materials, experimental systems and methods used in many studies. Here, indicate whether each material, system or method listed is relevant to your study. If you are not sure if a list item applies to your research, read the appropriate section before selecting a response.

Materials & experimental systems

n/a	Involved in the study
☒	☐ Antibodies
☒	☐ Eukaryotic cell lines
☒	☐ Palaeontology and archaeology
☒	☐ Animals and other organisms
☒	☐ Clinical data
☒	☐ Dual use research of concern

Methods

n/a	Involved in the study
☒	☐ ChIP-seq
☒	☐ Flow cytometry
☒	☐ MRI-based neuroimaging
